# A Boolean network model of hypoxia, mechanosensing and TGF-β signaling captures the role of phenotypic plasticity and mutations in tumor metastasis

**DOI:** 10.1371/journal.pcbi.1012735

**Published:** 2025-04-16

**Authors:** Grant Greene, Ian Zonfa, Erzsébet Ravasz Regan

**Affiliations:** Biochemistry and Molecular Biology, College of Wooster, Wooster, Ohio, United States of America; Inria Saclay: Inria Centre de Recherche Saclay-Ile-de-France, FRANCE

## Abstract

The tumor microenvironment aids cancer progression by promoting several cancer hallmarks, independent of cancer-related mutations. Biophysical properties of this environment, such as the stiffness of the matrix cells adhere to and local cell density, impact proliferation, apoptosis, and the epithelial to mesenchymal transition (EMT). The latter is a rate-limiting step for invasion and metastasis, enhanced in hypoxic tumor environments but hindered by soft matrices and/or high cell densities. As these influences are often studied in isolation, the crosstalk between hypoxia, biomechanical signals, and the classic EMT driver TGF-β is not well mapped, limiting our ability to predict and anticipate cancer cell behaviors in changing tumor environments. To address this, we built a Boolean regulatory network model that integrates hypoxic signaling with a mechanosensitive model of EMT, which includes the EMT-promoting crosstalk of mitogens and biomechanical signals, cell cycle control, and apoptosis. Our model reproduces the requirement of Hif-1α for proliferation, the anti-proliferative effects of strong Hif-1α stabilization during hypoxia, hypoxic protection from anoikis, and hypoxia-driven mechanosensitive EMT. We offer experimentally testable predictions about the effect of VHL loss on cancer hallmarks, with or without secondary oncogene activation. Taken together, our model serves as a predictive framework to synthesize the signaling responses associated with tumor progression and metastasis in healthy vs. mutant cells. Our single-cell model is a key step towards more extensive regulatory network models that cover damage-response and senescence, integrating most cell-autonomous cancer hallmarks into a single model that can, in turn, control the behavior of in silico cells within a tissue model of epithelial homeostasis and carcinoma.

## Author summary

The cellular environment in and around a tumor can aid cancer progression by promoting several cancer hallmarks. This environment can affect growth and cell death, as well as a phenotype change that renders cells migratory and invasive: the epithelial to mesenchymal transition. Hypoxia (low oxygen availability) promotes this transition, while the attachment of cells to soft matrices or high cell density environments hinders it. These influences are often studied in isolation. As a result, their crosstalk is poorly understood. To address this, we have built a network model of cellular regulation that integrates a cell’s responses to hypoxia, the biophysical environment, and growth signals to model cell division, death, and the epithelial-to-mesenchymal transition in environments cells encounter during metastatic tumor progression. Our model reproduces a wide range of experimental cell responses and offers experimentally testable predictions about the emergence of cancer hallmarks, driven by mutations that affect the hypoxic response. Our single-cell model is a key step towards more extensive cell-scale models that also include cell aging and damage response. These, in turn, can serve as building blocks of a larger tissue model of healthy vs. cancerous epithelia.

## Introduction

Tumor formation is marked by the development of mutations leading to dysregulated cell growth and death. Accumulation of cancer hallmarks is further aided by the tumor microenvironment (TME) – the biochemical composition, biophysical structure of the tissue, and non-cancer cells residing inside and around a tumor, all of which exert a major influence on the development and progression of cancer [1,2]. The TME is both variable and dynamic, with primary and secondary metastatic sites found to have differing environments that further vary by cancer origin [3–5]. Interestingly, the TME can contribute to the initial formation of tumors, rather than simply relying on stochastic genetic change as originally believed for carcinogenesis [6]. As cancer remains the second leading cause of death globally, understanding the unique conditions that promote metastasis contributing to 70–90% of cancer mortality is paramount to identifying therapeutics to both prevent and treat it [7].

A rate-limiting step for metastasis is epithelial to mesenchymal transition (EMT), a process where cells gain mesenchymal characteristics and lose their apical-basal polarity; aiding their migration, tissue invasion, and dissemination to secondary sites [8]. Yet, the reverse process (MET) is also crucial for tumor growth at secondary sites [9,10]. EMT and MET often do not require mutations to arise; rather they emerge due to the phenotypic plasticity of cancer cells responding to the TME. The stepwise process of EMT is controlled by two central double-negative feedback loops [11,12]. The first switch flips when an epithelial cell transitions to a hybrid E/M state, requiring the activation of SNAI1 by extracellular signals, accompanied by repression of the epithelial microRNA miR-34 [13]. In this hybrid E/M state, cells lose their apical-basal polarity, become migratory, yet maintain adherens junctions with neighbors to facilitate collective migration. To complete full EMT, loss of the second epithelial microRNA, miR-200, must occur in conjunction with high expression of the EMT factor ZEB1 [14]. This leads to the loss of E-cadherin and all adherens junctions, prompting a full mesenchymal phenotype. While these two feedback loops are but a small part of the regulatory network driving EMT, their central role is illustrated by the fact that hybrid E/M states were first predicted by a computational model of this circuit [11,12], later confirmed *in vitro* [15–18] and *in vivo* [19–22]. A series of computational models of increasing complexity followed, exploring the role of phenotypic stability factors [23,24], the connection between hybrid E/M cells and cancer stemness [18], spatial patterning in tumors [25], and the role of biological noise in EMT [26]. Most relevant for this study are models that capture the crosstalk between multiple EMT-driving extracellular signals [27]. Among these, the influential large Boolean model by Steinway et al. integrated TGFβ, Wnt, Notch, Hedgehog, and serval growth signaling pathways, predicting and verifying several interventions capable of controlling EMT *in vitro* [28,29].

In addition to well-characterized transforming signals such as TGFβ secreted by nearby cells, EMT is influenced by two key aspects of the TME. First, the concentration of oxygen is known to decrease in response to tumor growth [30]. The increased size of solid tumors coupled with poor vascularization and vessel integrity lead to hypoxia – defined as an oxygen supply of < 40 mmHg or < 6% O_2_ [31,32]. Tumor cells respond to hypoxic conditions with the secretion of Vascular Endothelial Growth Factor (VEGF) to induce angiogenesis and meet their oxygen demand [33,34]. Yet, blood vessel formation within tumors is highly dysregulated and ineffective at relieving hypoxia [32,35]. Acute, moderate hypoxia, in turn, promotes invasion and metastasis – an alternative pathway for tumor cells to resolve the oxygen demand by migrating to oxygen-rich tissue. Second, EMT is highly dependent on the biophysical environment. Namely, high cell density hinders EMT [36,37], while the increased matrix stuffiness characteristic of fibrotic tissue promotes it [38,39]. Both hypoxia and the biophysical environment were shown to contribute to immuno-, radio- and chemotherapy resistance, furthering cancer mortality [40–44]. However, the context-dependent crosstalk of hypoxia and the biophysical environment in driving EMT and MET are not well understood.

**Fig 1:**
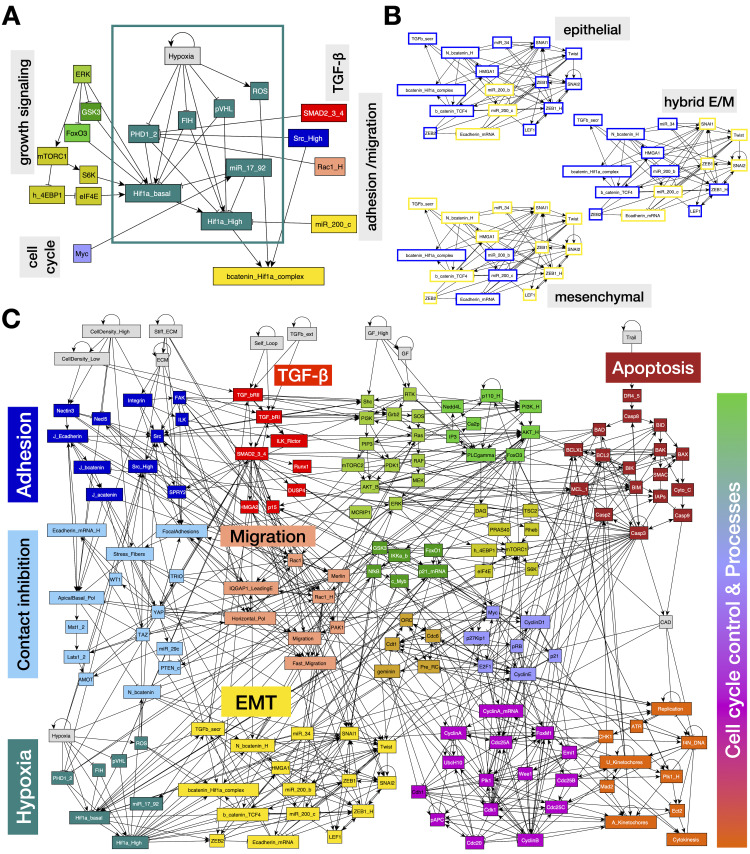
The hypoxic response affects proliferative signaling and EMT. A) Regulatory module depicting the canonical and non-canonical hypoxic response, along with influences from other signaling pathways and cell states. B) Three stable attractors of the isolated EMT regulatory switch (S2 File). *Orange/blue node borders:* ON/OFF. C) Modular network representation of our extended Boolean model. *Gray:* inputs representing environmental factors; *dark blue:* Adhesion signals; *red: TGFβ* signaling; *green*: Growth Signaling (*lime green*: basal AKT & MAPK, *bright green:* PI3K/AKT oscillations, *mustard*: mTORC1, *dark green: NF-κB, GSK3, FoxO1*); *dark red:* Apoptotic Switch*; light blue:* Contact Inhibition; *pink/light orange:* Migration; *light brown:* Origin of Replication Licensing; *lilac:* Restriction Switch; *purple:* Phase Switch; *dark orange:* cell cycle processes; *yellow:* EMT switch; *teal:* hypoxic response; → : activation; ⊣ : inhibition.

The hypoxic response occurs through two mechanisms: a canonical response mediated transcriptionally by Hypoxia Inducible Factor (HIF) proteins, and a non-canonical loss of oxygen-dependent degradation enzyme function, increasing the stability of target proteins [31,45–47]. HIF proteins are heterodimeric transcription factors comprised of an oxygen-sensitive Hif-α domain paired with a constitutively stable Hif-β domain. Regulation of the Hif-α subunit occurs through the oxygen-dependent hydroxylation of its proline residues, performed by prolyl hydroxylase domain (PHD) enzymes (Fig 1A) [48]. This in turn allows for recognition by E3 ubiquitin ligase Von Hippel Lindau protein (pVHL), leading to rapid Hif-1α degradation [49]. Additionally, the Factor Inhibiting HIF (FIH) hydroxylase enzyme uses O_2_ as a substrate for asparagine hydroxylation, inhibiting HIF binding to transcription co-activator p300 and thus blocking its transcriptional activity [50]. Most higher eukaryotes have three oxygen-sensitive HIFα isoforms; Hif-1α, Hif-2α, and Hif-3α [51]. The first discovered and best understood subunit is Hif-1α, stabilized in response to acute hypoxic stress [52]. In contrast, Hif-2α is preferentially stabilized during long-term hypoxic reprogramming, offering temporal control over HIF regulation [53,54]. Finally, Hif-3α forms a negative feedback loop with Hif-1α to fine-tune long-term hypoxia, while maintaining its unique transcriptional activity [55,56]. In comparison to the ubiquitously expressed Hif-1α, Hif-2α and -3α are more temporal and cell-specific [51,57,58].

Since the discovery of Hif-1α, several hundred genes have been discovered to be differentially expressed under hypoxia as a result of HIF transcription, affecting a wide array of signaling pathways [59–61]. Critically, both mesenchymal factors SNAI1 and ZEB1 are transcriptional targets of Hif-1⍺ [62,63]. Additionally, several other mesenchymal markers are shared targets Hif-1⍺ and the classical EMT-inducing TGF-β pathway [64,65]. Independent of HIF transcriptional regulation, non-canonical hypoxia responses involving the loss of oxygen-dependent PHD hydroxylation and the accumulation of reactive oxygen species (ROS) through the electron transport chain were also shown to promote EMT. For example, PHD/pVHL has been implicated in repressing Transforming Growth Factor-β (TGF-β), contributing to increased TGF-β expression under hypoxia [66]. Moreover, ROS accumulation caused by increased glycolysis under hypoxia contributes to Src activation and PHD inhibition, furthering invasion, metastasis, apoptosis resistance, and treatment resistance [67–69]. Src tyrosine kinase activity upregulates ERK [70] and is required for the loss of cadherin-dependent cell-cell contacts under hypoxia [71].

Despite the delineation of molecular pathways responsible for canonical and non-canonical hypoxic responses, downstream effects on the cell can appear paradoxical. For example, the Hif-1⍺-driven Warburg effect is required for proliferative metabolism [72], yet extreme hypoxia can inhibit proliferation [73,74]. While metabolic reprogramming under chronic hypoxia aids proliferation [75], acute stabilization of Hif-1α under hypoxia has been shown to prevent proliferation through strong Myc antagonization [74]. Similarly, while hypoxia induces apoptosis, hypoxia-driven EMT confers resistance to anoikis (apoptosis triggered by loss of adhesion) [76,77]. The pro- and anti-apoptotic effects of hypoxia are suggested to be distinguished by severity and length, governed by two competing cellular compartments: the cytosol vs. mitochondria. On one hand, hypoxia-driven loss of mitochondria membrane potential and decreased ATP production can increase mitochondrial membrane permeability, resulting in cytochrome C release and downstream caspase cleavage under severe hypoxia [78,79]. Under moderate hypoxia, localization of Hif-1⍺ to the mitochondria prevents oxidative-stress-induced apoptosis by reducing mitochondrial transcription and ROS production [80]. On the other hand, Inhibitor of Apoptotic Protein-2 (IAP-2) expression increases under hypoxia, providing cytosolic prevention of apoptosis independently of Hif-1α activity [81]. Loss of HIF function attenuated these behaviors, demonstrating the necessity of the canonical pathway. These differing outcomes highlight the need to map the precise combinations of environmental conditions leading to each response. Furthermore, the effect of the biophysical environment on these diverging responses remains largely unexplored.

The tumor microenvironment is heterogeneous, both in terms of the spatial distribution of different tumor-associated cells and temporal differences in its molecular composition. Thus it is important to understand how the biophysical environment modulates the phenotypic diversity of tumor cell populations [82,83]. For example, single-cell sequencing experiments have found heterogeneous expression of epithelial and mesenchymal markers emerging from the same tumor site, confirming a broad range of states within the EMT spectrum [22,84,85]. As oxygen availability in tumors is also not static, it is important to understand the interplay between these two highly dynamic pathways: the hypoxic response and the induction of EMT in response to the biophysical environment.

The goal of this study is to map the way hypoxia alters these responses in a biophysical milieu-dependent manner. In a previously published Boolean model of mechanosensitive EMT, we reproduced known effects of the biophysical environment on EMT and TGFβ signaling. We predicted that in the absence of TGFβ, the decision between no response, partial EMT, and full EMT in mitogen-stimulated epithelial cells on a stiff ECM is dictated by cell density [86]. Here we expanded this model by including key regulators of the canonical and non-canonical hypoxic response and integrated their effect on growth factor signaling, proliferation, EMT, and apoptosis. Our model reproduces the requirement of Hif-1α for normal proliferation [87], as well as the antiproliferative effects of strong Hif-1α stabilization during hypoxia [73,74]. Additionally, we capture the molecular mechanism of Hif-1α mediated SNAI1/2 and ZEB1 upregulation [62,63,88] and reproduce hypoxia-driven EMT [63,89]; independent of TGF-β(66) but dependent on a stiff ECM [90]. Our model also reproduces hypoxic protection from anoikis [76]. We offer experimentally testable predictions about the context-dependent effects of TGF-β signaling on hypoxia-mediated EMT, as well as VHL loss on cancer hallmarks with or without secondary oncogene activation. Taken together, we offer a mechanistic, predictive framework to synthesize the signaling responses responsible for hypoxic behaviors associated with tumor progression and metastasis in healthy vs. mutant cells.

## Results

### Expression of Hif-1α is required for proliferation

To model the way hypoxia contributes to EMT and proliferation, we expanded our previous mechanosensitive Boolean network, which includes a detailed cell cycle control circuit driven by growth signaling (MAPK, PI3K/Akt, mTORC) integrated with cell-cell and cell-ECM adhesion, contact inhibition, control of EMT, and apoptosis [86]. Here we incorporated a hypoxia module with canonical and non-canonical response mechanisms [86] (Fig 1A; model in S1 File). This module captures the acute, moderate hypoxic response, focused on Hif-1⍺ and its downstream transcriptional activity [91]. In cells, Hif-1⍺ activation and signaling have two functionally distinct regimes, requiring two Boolean nodes to properly capture its effects. Moderate Hif-1⍺ is activated in response to growth factor signaling under normal O_2_ conditions to boost glycolysis and proliferative metabolism; we encode this as *Hif1a_basal *= ON. A stronger activation occurs in response to hypoxic conditions or TGF-β signaling, typically in combination with deactivation of Hif-1⍺ inhibitors such as pVHL (modeled as *Hif1a_High* = ON). Next, we included Hif-1⍺ mediated transcription of mesenchymal markers including SNAI1/2, ZEB1, LEF1, β-catenin (and its subsequent nuclear localization/ complex formation with Hif-1⍺) [46,62,63,92]. Fig 1B shows the three stable states (fixed-point attractors) of the isolated EMT module, matching the expected activity of EMT regulators in three distinct phenotypic cell states along the EMT spectrum; namely epithelial (top), hybrid E/M (middle), and mesenchymal (bottom). The full network is shown in Fig 1C, while the Boolean rules and detailed biological justification for each of the 165 nodes and 763 links are included in S1 Text.

First, we validated our model by comparing its behavior to 150 experimental assays curated from 13 primary literature papers focused on EMT and hypoxia signaling. The 150 assays covered cell behaviors in response to 19 distinct protein or microRNA perturbations (knockdown/ overexpression) and involved 229 unique statistical tests, listed in S1 Table. Our model reproduced 92% [138] of the 150 assays (207 of 229 statistical tests; auto-generated figures in S12 Fig). Of the 12 failed assays marked in S1 Table, 7 involved experiments that directly contradict others on the list; the others pointed to model limitations detailed in our *Discussion*. Below, we showcase key validation experiments related to hypoxic signaling, along with a series of model predictions.

**Fig 2:**
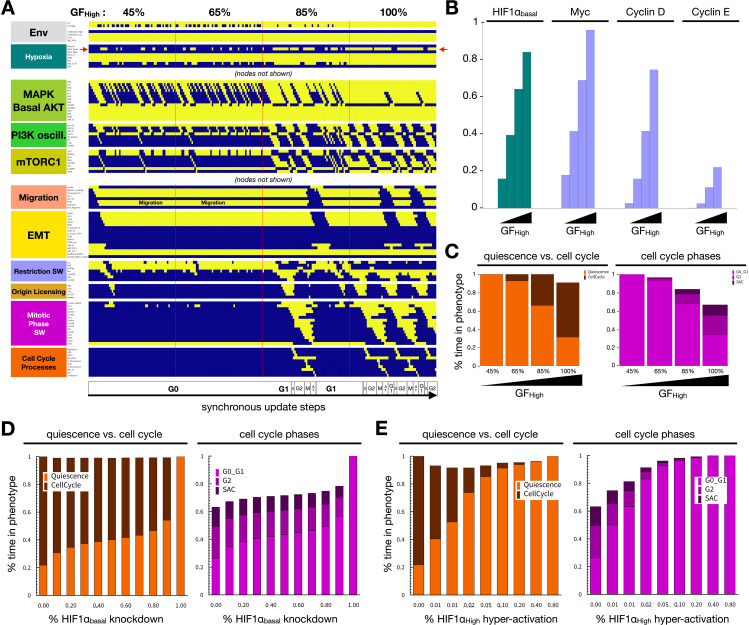
Hif-1α is required for proliferation under normoxia. A) Dynamics of relevant regulatory molecule expression/activity during exposure of a quiescent cell to increasing mitogen signaling (60 update-steps of 45%, 65%, 85%, and 100% high GF; full network dynamics in S1A Fig). *X-axis:* update steps annotated by cell cycle phase (G0: quiescence; G1: start of cell cycle entry; S: DNA synthesis; G2: growth phase 2; M: metaphase; A T: anaphase, telophase, cytokinesis); *y-axis*: nodes organized by regulatory module; *yellow/blue*: ON/OFF; *red arrow:* Hif1a_basal node. B) Average expression of *Hif1a_basal* (*teal*), Myc, CyclinD1, and CyclinE (*lilac*) in the four consecutive time windows with increasing GF_High input (45%, 65%, 85%, 100%) in an ensemble of 10,000 simulations representing individual cells. C) Fraction of time cells spend in quiescent (*orange*) vs. proliferative (*dark red*) states (*left*) and fraction of time a cell’s state matched the G0_G1, G2, or Spindle Assembly Checkpoint (SAC) state of the phase switch (*right*) in subsequent time windows with increasing growth factor (45%, 65%, 85%, 100%) in an ensemble of 10,000 cells. D) Fraction of time cells spend quiescent (*orange*) vs. proliferative (*dark red*) states (*left*) and fraction of time cell states match the G0_G1, G2, or SAC state of the phase switch (*right*) at increasing levels of *Hif1a_basal* knockdown (*autocrine TGF-β:* 5% TGFb_secr knockdown). E) Fraction of time cells spend in quiescence (*orange*) vs. in cell cycle (*dark red*, *left*) and fraction of time cell states match the G0_G1, G2 or SAC state of the phase switch (*right*) at increasing levels of *Hif1a_High* hyper-activation (*log2 scale:* 0, 0.625, 1.25, 2.5, 5, 10, 20, 40 and 80% Hif1a_High = ON; *autocrine TGF-β:* 5% TGFb_secr knockdown). *Length of time-window for continuous runs:* 500 steps (~24 wild-type cell cycle lengths); *total sampled live cell time:* 100,000 steps; *update*: synchronous; *initial condition for all sampling runs*: epithelial cells in GF:1, CellDensity_High:1, Stiff_ECM:1, Trail:0, Self_Loop:1, TGFb_ext:0, Hypoxia:0; *environment of sampling runs:* saturating growth signals (GF_High:1), moderate density (CellDensity_Low:1).

To test whether the updated model can reproduce the context-dependent influence of Hif-1⍺ activation on proliferation, we used synchronous Boolean update to map the model’s attractors in each cellular environment (*Computational Methods IV*). As our starting cell state, we chose a quiescent epithelial phenotype under normoxia, low mitogen exposure sufficient for quiescence, plated at moderate density (e.g., at a monolayer’s edge). We then exposed this cell to increasing concentrations of mitogen (Fig 2A, *full time-course:* S1 Fig). As Hif-1⍺ is a transcriptional inducer of glycolytic genes, we expected a moderate Hif-1⍺ activity increase despite the presence of oxygen, required for maintaining the energy demand associated with proliferation [93–95]. Indeed, our model showed intermittent, moderate Hif-1⍺ activation preceding each division (Fig 2A, *arrows*). The average activity of the *Hif1a_basal* node in a population under increasing mitogen exposure went up in tandem with Myc, Cyclin D1, and Cyclin E (Fig 2B), parallelling the increase in cell cycle progression (Fig 2C). Indeed, the loss of Hif-1⍺ under normoxia reduced proliferation in our model (Fig 2D), as demonstrated *in vitro* [87]. Yet, high levels of Hif-1⍺ of accumulation were also associated with cell cycle arrest due to Myc inhibition [73,74], reproduced by our model by overexpression of the *Hif1a_High* node in strongly mitogen-stimulated cells (Fig 2E). Strong Hif-1⍺ activation blocks the cell cycle via Myc repression, as demonstrated by partial cell cycle rescue in cells with hyper-active Myc (S2 Fig). In summary, both over and under-expression of Hif-1⍺ severely limit proliferation in our model, as described in the experimental literature.

### Hypoxia arrests proliferation and induces EMT

**Fig 3:**
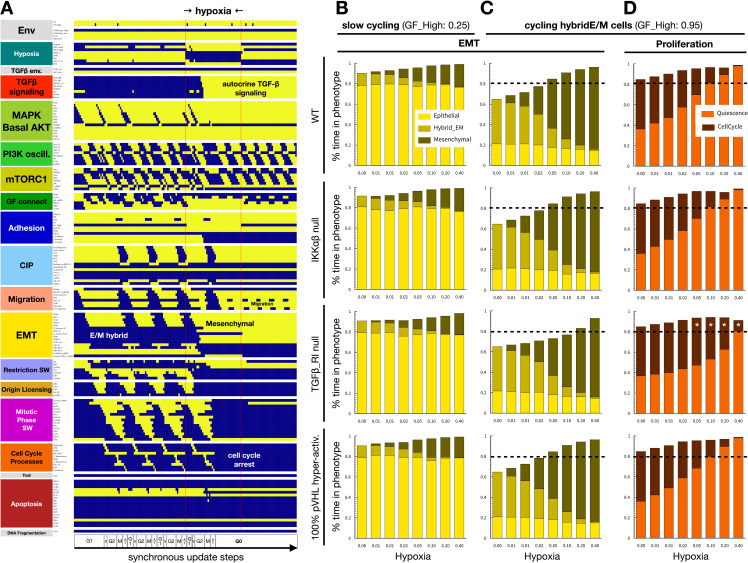
Hypoxia induces EMT and blocks the cell cycle independently of TFG-β signaling. A) Dynamics of regulatory molecule expression in a proliferating cell in response to hypoxia (80/ 40/ 40 update-steps of normoxia/hypoxia/normoxia at 95% saturating mitogen exposure and moderate cell density). *X-axis:* update steps annotated by cell cycle phase (G0: quiescence; G1: start of cell cycle entry; S: DNA synthesis; G2: growth phase 2; M: metaphase; A/T: anaphase, telophase, cytokinesis); *y-axis*: nodes organized by regulatory module; *yellow/blue*: ON/OFF; *black/white labels:* relevant phenotypes; *update*: synchronous. B-C) Fraction of time cells at moderate density exposed to weak (B) or strong mitogens (C) spend in epithelial (*yellow*), hybrid E/M (*dark yellow*), and mesenchymal (*mustard*) states at increasing hypoxia exposure. D) Fraction of time strong mitogen-exposed cells spend in quiescence (*orange*) vs. in cell cycle (*dark red*) at increasing hypoxia exposure. B-D) *1*^*st*^ *row:* wild-type; *2*^*nd*^ *row*: 100% IKKɑ/β knockdown; *3*^*rd*^ *row*: 100% TGFβ-RI knockdown; *4*^*th*^ *row:* 100% pVHL hyper-activation. *X axis (log*_*2*_ *scale):* 0, 0.625, 01.25, 2.5, 5, 10, 20 and 40% hypoxia; *black dashed line:* 80% time; *white stars:* altered from wild-type; *length of time-window for continuous runs:* 100 steps (~5 wild-type cell cycle lengths); *total sampled live cell time:* 100,000 steps; *update*: synchronous; *initial condition for all sampling runs*: epithelial cells in GF:1, CellDensity_Low:1, Stiff_ECM:1, Trail:0, Self_Loop:1, TGFb_ext:0, Hypoxia:0; *environment of sampling runs:* GF_High = 0.25 (B) or GF_High = 0.95 (C) and moderate density (CellDensity_Low:1); *autocrine TGF-β loop:* 5% TGFb_secr knockdown.

Having demonstrated the role of Hif-1α expression in proliferation under normoxia, we next assessed the effect of the hypoxic response on proliferation and EMT. As described above, Hif-1⍺ overexpression in the presence of oxygen leads to cell cycle arrest due to Myc repression (S2 Fig) [74]. Hypoxia induced a similar cell cycle arrest in our model, as lack of oxygen blocked PHD2 hydroxylation and downstream VHL-mediated Hif-1⍺ degradation [92] (Fig 3A). In addition to cell cycle arrest, our simulation showed hypoxia-induced EMT in cycling cells (Fig 3A), as expected from experimental literature [87,90,96]. Namely, hypoxia resulted in Hif-1⍺ mediated transcription of mesenchymal transcription factors SNAI1/2, ZEB1, LEF1, and nuclear β-catenin, generating a mesenchymal phenotype and complete loss of epithelial markers (S3A Fig) [63,92,97]. In addition, non-canonical (Hif-1⍺ independent) stabilization of IKK⍺ due to a lack of oxygen-dependent hydroxylation by PHD led to NF-κB activation (S3A Fig) [98].

As seen in Fig 3A, hypoxia triggers autocrine TGF-β signaling due to a combination of oxygen-dependent loss of VHL activity [66], Hif-1⍺ mediated activation of TGF-β converting enzyme furin [99], and critically, secretion of TGF-β induced by EMT transcription factors [46,100]. Yet, this effect is a consequence rather than a driver of EMT under hypoxia, as TGF-β receptor I knockdown does not block hypoxia-induced EMT – though it slows it in mild hypoxia (Fig 3B-3C). That said, lack of TGF-β signaling does weaken cell cycle arrest in the presence of mitogens (Fig 3D). Moreover, hypoxic EMT in VHL independent (Fig 3B-3C), as PHD2-mediated hydroxylation and degradation of Hif-1⍺ requires oxygen even in VHL-overexpressing cells (S3B Fig) [48,101]. In contrast, TGF-β can only stabilize Hif-1⍺ in the absence of VHL (S3C Fig) [101]. As EMT transcription factor induction occurs independently through either Hif-1⍺ or SMAD2/3/4, the two pathways do not rely on each other to promote EMT (S3D Fig).

### The biophysical environment and oxygen availability modulate EMT and its reversal

**Fig 4:**
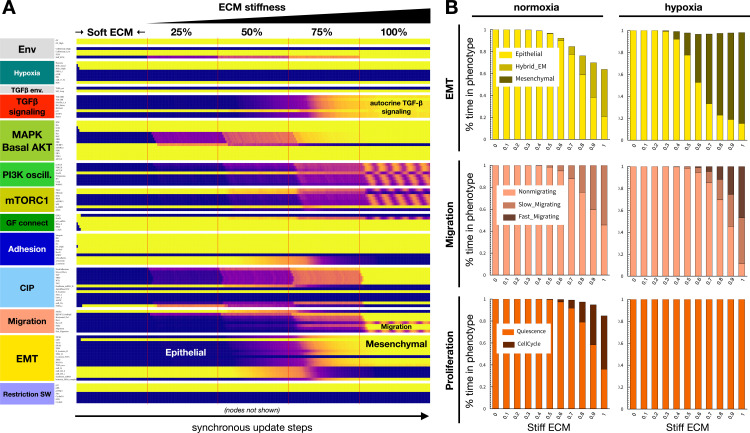
A stiff ECM aids hypoxia-induced EMT. A) Dynamics of relevant regulatory molecule expression in a quiescent cell on a soft ECM under hypoxia, in response to increasing ECM stiffness (50 update-steps of 0, 25, 50, 75, and 100% Stiff_ECM = ON at saturating mitogen exposure, moderate cell density, and 5% TGFb_secr knockdown). *X-axis:* update steps; *y-axis*: nodes organized by regulatory module; *yellow/purple/blue scale*: 100% ON/ 50% on/ 100% OFF; *black/white labels:* relevant phenotypes; *update*: synchronous; *full time-course:* S4A Fig. B) Fraction of time normoxic (*left*) vs. hypoxic (*right*) cells at moderate density and saturating mitogen exposure, plated on ECM of increasing stiffness, spend in: *top:* epithelial (*yellow*), hybrid E/M (*dark yellow*) and mesenchymal (*mustard*) states; *middle*: non-migrating (*light pink*), slow-migrating (*dark pink*) and fast-migrating (*brow-pink*) states; *bottom:* quiescence (*orange*) vs. in cell cycle (*dark red*). *Length of time-window for continuous runs:* 100 steps (~5 wild-type cell cycle lengths); *total sampled live cell time:* 100,000 steps; *update*: synchronous; *initial condition for all sampling runs*: epithelial cells in GF:1, CellDensity_Low:1, Stiff_ECM:1, Trail:0, Self_Loop:1, TGFb_ext:0, Hypoxia:0; *environment of sampling runs:* Hypoxia:0(*left*)/1(*right*), GF_High:1, CellDensity_Low:1; *autocrine TGF-β loop:* 95% (5% TGFb_secr knockdown).

Experimental literature on the crosstalk between the hypoxic response and ECM primarily focuses on hypoxia-induced remodeling by fibroblasts, or induction of the Warburg effect [102–106], leaving the interplay of hypoxia and ECM stiffness or cell density underexplored. One of the few direct studies on the dependence of EMT on ECM stiffness under hypoxia demonstrated that mesenchymal marker induction under hypoxia positively correlates with matrix stiffness in vitro^12^. To test whether our model can reproduce this, we started our next simulation with a quiescent epithelial cell on a soft ECM, at low density and under hypoxia (arrested due to an inability to stretch, as well as hypoxia). We then gradually increased ECM stiffness by stochastically turning ON the *Stiff_ECM* node, until its saturation (*Stiff_ECM* = ON). As shown in Fig 4A, mesenchymal markers gradually turned on starting from *Stiff_ECM = 0.5*, and cells maintained a fully mesenchymal state by *Stiff_ECM = 0.75* (complete time-course: S4A Fig). In contrast, cells under normoxia underwent partial EMT and entered the cell cycle, never reaching high levels of ZEB1 to repress miR-200c and E-cadherin expression (Figs 4B and S4B). The average SNAI1 activity of a heterogeneous population of *in silico* cells at increasing ECM stiffness closely resembled its relative expression *in vitro* (S5A Fig). Additionally, the effect of matrix stiffness on migration was stronger under hypoxia, aligning with the study’s wound-healing results (Fig 4B, *middle row*). Lastly, our model predicts that matrix stiffness affects hypoxia-induced EMT more profoundly than TGF-β induced EMT (S5B Fig). The EMT-induction threshold of hypoxia increases rapidly on softer ECMs, while the effect on TGF-β-induced EMT is significantly milder. We further predict that on soft ECMs that cannot support stress fiber formation and migration, only a combination of strong hypoxia and near-saturating TGF-β can induce EMT (S5B Fig, *left*). In contrast, high cell density blocks EMT in response to either signal, as well as their combination, while isolated mitogen-stimulated cells undergo biomechanically induced EMT even in the absence of hypoxia or external TGF-β (S5C Fig) [86].

### Loss of the Hif-1α repressor VHL aids metastatic cell behaviors

The development of clear cell renal cell carcinoma (ccRCC) is marked by mutations to Von Hippel Lindau gene, resulting in reduced expression or inactive forms of VHL protein and aberrant Hif-1⍺ expression under normoxia as well as hypoxia [107]. This mutation contributes to ccRCC being the 13^th^ most common malignancy throughout the world [108], as well as its reduced treatment efficacy and markedly high mortality rate [109]. While VHL loss is a hallmark of ccRCC, this mutation alone is unable to consistently induce tumorigenesis in mice [110,111]. Modeling VHL-deficient cells, we predict that the loss of VHL can drive metastatic cell behaviors under biophysical microenvironments that do not induce EMT in wild-type cells. To this end, we simulated wild type vs. 90% VHL knockdown in cells on soft to stiff ECM, at densities ranging from isolated cells to dense fully confluent monolayers under normoxia (S6A Fig, *top left*). In the presence of a near-saturating growth stimulus, only cells with some access spreading space and a very stiff ECM underwent full EMT. In contrast, the combination of ECM and density values leading to EMT were significantly expanded in VHL-deficient cells. Loss of VHL boosted full EMT rates, particularly at moderate ECM stiffness and in cells that had only intermittent access to space to establish horizontal polarity (S6A Fig, *middle/bottom left*). While hypoxia blunted its effects, the boost to EMT was still significant at moderate hypoxia (S6A Fig, *middle/right columns*). As the loss of VHL stabilized Hif-1⍺, it also resulted in a full cell cycle arrest (S6B Fig), indicating that a boost to EMT in VHL-null cells would be counterbalanced by arrested growth.

Overall, our VHL-deficient simulations indicate that a combination of VHL loss and cell cycle-promoting oncogene activation is required for cancer progression, breaking Hif-1⍺ and/or EMT-induced cell cycle arrest. Indeed, ccRCC tumors with a VHL missense mutation often harbor Cdk4/6 and/or Cyclin D amplification and are highly proliferative [112]. Moreover, the most proliferative subtype of ccRCC is both VHL deficient and has enhanced MYC activity [113]. To test whether our model could reproduce proliferation rescue in VHL-null cells, we re-ran the above simulations at varying ECM stiffness and cell density in VHL-deficient cells with or without Myc and/or Cyclin D hyper-activation (S7A Fig). Intriguingly, only joint hyper-activation of Myc and Cyclin D could significantly boost the proliferation of VHL-deficient cells (S7B Fig*, top*). This co-occurred with a mild reduction in EMT compared to VHL deficiency alone (S7B Fig*, bottom*). In contrast, pairing Cyclin D with ccRCC-associated mutations upstream in the PI3K or MAPK pathway, such as PI3K/mTORC1/ERK hyper-activation or p21/PTEN loss [114], only led to weak cell cycle rescue (S7C Fig, *top*); though most of these combinations also mildly boosted EMT (S7C Fig, *bottom*). Pairing Myc with the same cancer mutations only yielded weak cell cycle rescue with p21 loss (S7D Fig).

### Hypoxia prevents anoikis and epithelial apoptosis in response to TGF-β

**Fig 5:**
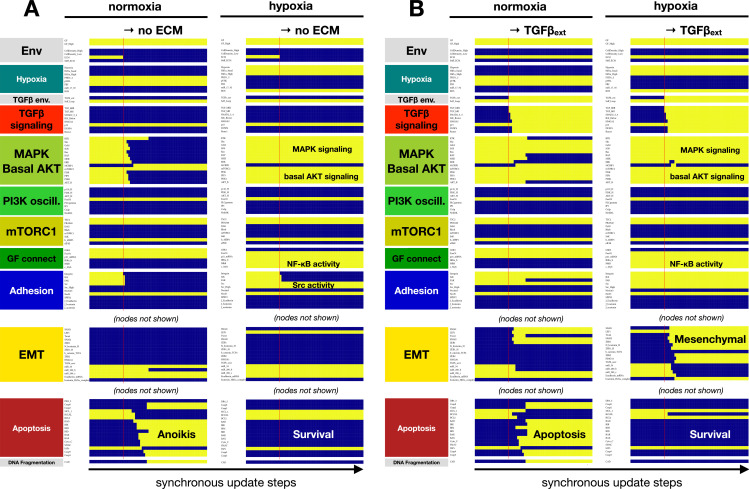
Hypoxia confers anoikis resistance and protection from TGF-β induced epithelial cell apoptosis on a soft ECM. A) Dynamics of relevant regulatory molecule expression in a quiescent cell detached from a soft ECM in normoxia (*left*) vs. hypoxia (*right*) (20 update-steps on soft ECM, 50 update-steps detached, 100% high GF). B) Dynamics of relevant regulatory molecule expression in a quiescent cell on a soft ECM, exposed to exogenous TGF-β in normoxia (*left*) vs. hypoxia (*right*) (20 update-steps no TGF-β, 50 update-steps 100% TGF-β). *X-axis:* update steps; *y-axis*: nodes organized by regulatory module; *yellow/blue*: ON/OFF; *black/white labels:* relevant phenotypes; *update*: synchronous; *full time-course:* S8 Fig.

In addition to inducing a mesenchymal state, the lack of oxygen has been shown to block anoikis and apoptosis; further contributing to the mortality rate of hypoxic tumor formation [79,115]. As previously shown in Sullivan et al. [86], our mechanosensitive model can reproduce anoikis-mediated epithelial cell death in response to detachment from the extracellular matrix. To test if our model could also replicate hypoxia-mediated evasion of anoikis, we simulated detachment of a quiescent, isolated epithelial cell from a soft ECM exposed to saturating growth factors (Fig 5A; *full time-course:* S8 Fig). In contrast to normoxia, hypoxia repressed pro-apoptotic factor activation and conferred anoikis resistance [76,116]. This was due to the ability of cells suspended under hypoxia to maintain high levels of MAPK signaling and basal AKT activity. This was supported by hypoxia- and ROS-mediated Src upregulation, an effect that did not require integrin-mediated attachment and thus prevented anoikis (Fig 5A*, right*) [76]. The non-canonical hypoxic response also contributed to anoikis resistance through NF-κB nuclear translocation, reinforcing BCL-2 and BCL-X_L_ expression [117,118]. Along similar lines, our model reproduces TGF-β induced apoptosis of epithelial cells on a soft matrix (Fig 5B*, left*) [119]. Specifically, the model depicts an increase in BAX/BIM signaling along with BCL-X_L_ loss, followed by apoptosis. In comparison, cells exposed to TGF-β in a hypoxic environment are predicted to resist apoptosis through increased Src and MAPK activity (Fig 5B*, right*).

### Hypoxia and reoxygenation can drive the metastatic cascade in the absence of exogenous transforming signals

**Fig 6:**
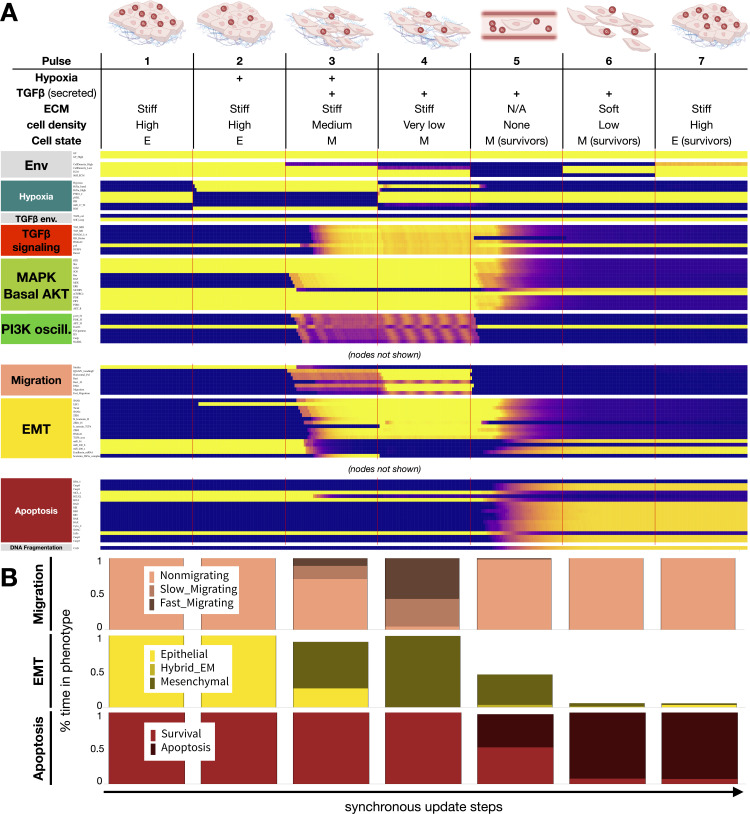
As cells follow the metastatic cascade, hypoxia-induced EMT protects cells in circulation, reversed at the secondary site by high cell density. A) Dynamics of relevant regulatory molecule expression in an ensemble of 1000 quiescent epithelial cells exposed to a sequence of microenvironments along the metastatic cascade (100 update steps per pulse). *Pulse 1:* normoxia, stiff ECM, high density; *Pulse 2:* hypoxia, stiff ECM, high density; *Pulse 3:* hypoxia, stiff ECM, medium density; *Pulse 4* (invasion): normoxia, stiff ECM, very low density; *Pulse 5* (intravasation): normoxia, no ECM, no neighbors; *Pulse 6* (extravasation): normoxia, soft ECM, low density; *Pulse 7* (MET): normoxia, stiff ECM, high density; *X-axis:* update steps; *y-axis*: nodes organized by regulatory module; *yellow/blue*: ON/OFF; *black/white labels:* relevant phenotypes; *update*: synchronous; *autocrine TGF-β:* 5% TGFb_secr knockdown; *full time-course:* S8 Fig. B) Average fraction of time an ensemble of 1000 cells spend in *top:* non-migrating (*light pink*), slow-migrating (*dark pink*) and fast-migrating (*brow-pink*) states; *middle*: epithelial (*yellow*), hybrid E/M (*dark yellow*) and mesenchymal (*mustard*) states; *bottom:* survival (*red*) vs. in apoptotic (*dark red*) states for each pulse along the metastatic cascade in panel A. *Image credits:* metastatic cascade adapted from https://commons.wikimedia.org/wiki/File:Contribution_of_EMT_to_cancer_progression.jpg.

Next, we aimed to identify the biophysical microenvironments required for MET following reoxygenation, a crucial step in secondary tumor formation [120]. To simulate a metastatic cascade, allowing for both intravasation and extravasation from a solid tumor, we simulated a sequence of microenvironments designed to mimic the signals metastatic cells experience en route from a solid tumor toward a new metastatic site. To do this, we started with an epithelial cell at high cell density on a stiff ECM, such as the basement membrane [121] (Fig 6A, *pulse 1*). In a growing solid tumor, this cell is likely to experience hypoxia (Fig 6A, *pulse 2*). Yet, due to density-dependent contact inhibition of proliferation as well as migration, this epithelial cell is protected from hypoxia-driven EMT. For EMT to start, a decrease in local density must occur (Fig 6A, *pulse 3*). In a tumor setting this may be due to cell death in the neighborhood, potentially due to nearby anoxic areas of necrosis (not modeled) [79] or changes to constraining structures such as the basement membrane [122]. Migrating away from the solid tumor, a mesenchymal cell can now reach areas of lower density and adequate oxygen, while maintaining its mesenchymal state (Fig 6A, *pulse 4*).

As hypoxia induces self-sustaining autocrine TGF-β signaling [14], transitioning back to an epithelial state requires breaking the autocrine loop. This can happen due to non-saturating TGF-β secretion in transit through the bloodstream, where cells have no access to a stiff ECM to keep mechanosensitive EMT signals active (Fig 6A, *pulse 5*). As a result, upon entering the vasculature, detachment from the ECM and other cells is a crossroad with two phenotypic outcomes: cells that maintain a mesenchymal state avoid anoikis, whereas cells that revert to an epithelial state undergo apoptosis (Fig 6A, *pulse 5*). Surviving cells then transition to an epithelial state upon reaching high cell density in the new tissue (Fig 6A, *pulse 7*). From here, they can re-enter a proliferative hybrid E/M state once they escape contact inhibition [123]. This completes the metastatic cascade, leading to new tumor growth. In contrast to cells with non-saturating autocrine TGF-β signaling, saturating TGF-β secretion prevents apoptosis in nearly all cells undergoing the metastatic cascade (S9 Fig). That said, even slight perturbations of the autocrine loop, modeled by reducing *TGFb_*secr, are predicted to have a major impact on the rate of apoptosis in the bloodstream, as well as upon transitioning onto a soft ECM (S10 Fig).

Another way to visualize the environment changes driving EMT and MET is to highlight the cell state sequence the above steps push our model through, shown on a comprehensive map of all relevant stable model phenotypes (attractors) organized by microenvironment (S11 Fig). Taken together, our mechanosensitive model can reproduce hypoxia-driven EMT, and its reversal is based on a realistic sequence of its biophysical environments, depicting molecular signaling which occurs throughout the metastatic cascade.

## Discussion

Despite the prevalence of hypoxic tumor microenvironments during solid tumor progression, the combined effect of hypoxia and the biophysical environment on cancer hallmarks remains underexplored. Here, we have built a dynamic regulatory network model focused on depicting the molecular signaling that occurs in response to oxygen deprivation (Fig 1). To do this, we incorporated a hypoxic response module into our previously published mechanosensitive EMT model [86]. Unlike published models exploring individual hypoxia-induced phenotypes [124–127], our model captures Hif-1α mediated transcription responsible for i) the induction as well as inhibition of proliferation (Fig 2), ii) propelling EMT in a mechanosensitive manner (Figs 3 and 4), and iii) preventing cell death in response to a changing TME (Fig 5). Taken together, our model can reproduce cell state changes along a full metastatic cascade (Fig 6), providing insight into the effects of a continuously changing biophysical environment during secondary tumor formation. The resulting cell behaviors cover cell-autonomous aspects of several cancer hallmarks, including *sustaining proliferative signaling*, *evading growth suppressors*, *activating invasion and metastasis*, *resisting cell death*, and *unlocking phenotypic plasticity* [128].

1. *Sustaining proliferative signaling* & *evading growth suppressors.* Our model captures the paradoxical relationship between Hif-1α and proliferation. On one hand, Hif-1α is required for proliferation through its role in the upregulation of the glycolytic machinery, including GLUT1, LDH-A, ALDA, and more (modeled indirectly; Fig 2D) [129]. On the other hand, the increased stability of Hif-1α under hypoxic conditions is known to induce cell cycle arrest through functionally repressing Myc, a key driver of proliferation (Fig 2E) [73,74]. Indeed, our model predicts that Myc hyperactivation can rescue the cell cycle under hypoxia (S2 Fig). In comparison to the effects of *acute* oxygen deprivation captured by our model, *chronic* hypoxia increases proliferation through Hif-2α-induced Myc transcription, increased MAPK/ERK signaling, and YAP1 activation [130–132]. Capturing this temporal shift in our model is an area of future interest.
2. *Activating invasion and metastasis.* While the effects of hypoxia on EMT are well-documented, the mechanosensitive nature of this response, as well as its crosstalk with TGF-β signaling, have not been modeled individually or collectively. Our model shows that hypoxia can induce EMT in cycling cells (Fig 3) [87], as long as they have access to a stiff ECM and some space to spread (Figs 4 and S5) [90]. In contrast, normoxic cells only undergo hybrid E/M in all but the most EMT-promoting biophysical environments (S5 Fig). Once established, a mesenchymal state is maintained regardless of hypoxia due to its ability to induce autocrine TGF-β signaling (Fig 3A), even in the presence of hyper-active VHL [101]. Yet, this autocrine signal is not required for EMT in hypoxic cells, as loss of TGF-β Receptor I does not block the transition (Fig 3B) [101]. In contrast, TGF-β can only boost Hif-1α stabilization in VHL-deficient cells. These nuances of crosstalk highlight the importance of modeling the biophysical environments and genetic interactions that compound the loss of VHL, a tumor suppressor pivotal to clear cell renal cell carcinoma (ccRCC) tumorigenesis [109]. These cancers have among the highest rates of metastasis and mortality [133]. Our model predicts that VHL loss alone, known to predispose patients to cancer [134], can push cells at moderate density and/or on a softer ECM into full EMT. These cells undergo EMT instead of maintaining an epithelial state or a reversible hybrid E/M phenotype required for repairing epithelial damage, as expected in healthy cells (S6 Fig) [135]. Furthermore, we highlight the importance of secondary driver mutations that directly impact cell cycle entry, such as joint cyclin D and Myc amplification/hyperactivation [112,113]. These mutations overcome the cell cycle arrest mediated by hyperactive Hif-1α and/or autocrine TGF-β signaling (S7 Fig), helping cancer cells combine sustained proliferation with an invasive mesenchymal state.
3. *Resisting cell death.* Our model not only reproduces anoikis resistance of epithelial cells under hypoxia [76] but also predicts that hypoxia can protect epithelial cells on a soft ECM from TGF-β-induced apoptosis (Fig 5).
4. *Unlocking phenotypic plasticity.* Our simulation of the metastatic cascade, from hypoxia-induced EMT in a primary tumor through anoikis resistance in the bloodstream to cell density-induced MET at a secondary site, our model captures the non-genetic phenotypic plasticity cancer cells tap into as they navigate different combinations of chemical and biomechanical environments (Fig 6). Interestingly, our model predicts that the ability to reach and sustain a fully mesenchymal state, with no break in autocrine TGF-β signaling, serves as an environmental bottleneck for circulating tumor cells (S9 and S10 Figs). Moreover, intermittent (non-saturating) TGF-β signaling can also render the transition back onto a soft ECM dangerous, as a weaker mesenchymal program is less apt at warding off the apoptotic effects TGF-β has on epithelial cells on soft ECM [86].

A particular strength of our model is its ability to reproduce a wide array of experimental results in wild-type, as well as mutant cells. Namely, the model matched the direction and significance of molecular or phenotypic changes in 138 of 150 independently conducted experimental assays from 13 primary papers focused on hypoxia and EMT (S1 Table), and 7 of its 12 failures were due to contradictory experiments in our list. This validation lends weight to our predictions, summarized below:

a) We predict that the loss of TGF-β signaling can weaken hypoxia-mediated cell cycle arrest (Fig 3D), and slow down hypoxia-induced EMT in cells with weak mitogenic signaling (Fig 3B-3C).
b) We predict that soft ECMs drastically raise the hypoxia threshold of EMT, while they impact TGF-β more mildly. On very soft ECMs unable to support stress fiber formation and migration, only a combination of strong hypoxia and near-saturating TGF-β can induce EMT (S5B Fig).
c) We predict that breaking the cell cycle inhibitory effects of VHL loss requires combined hyper-activation of Myc *and* Cyclin D (S7 Fig; less potent 2-gene combinations involve either p21 loss or Cyclin D hyper-activation). In contrast, upstream mutations that boost PI3K or MAPK signaling (alone or in combination with Myc) cannot overcome cell cycle arrest mediated by Hif-1α and/or TGF-β.
d) We predict that epithelial cells on a soft ECM exposed to TGF-β are protected from apoptosis under hypoxic conditions (Fig 5B).
e) Finally, we predict that circulating mesenchymal cells are highly sensitive to intermittent weakening of autocrine TGF-β signaling, and thus only a fraction of them survives anoikis (S9 and S10 Figs). This could be tested with cell suspension experiments on cells that first undergo EMT, treated with increasing doses of TGF-β or TGF-β RI inhibitors.

Our use of a Boolean modeling framework, despite its advantages in allowing us to build a 165-node regulatory network with 763 links, presents two technical limitations of particular interest here (also discussed in [86]). *First,* modeling autocrine signaling in a Boolean framework means that a positive feedback loop can lock in between a signaling pathway and the transcription/secretion of the signal itself, such as TGF-β. This loop implicitly assumes that a single mesenchymal cell produces sufficient levels of bio-available TGF-β to maintain saturating signaling on its own receptors, a likely problematic assumption. To get around the resulting artifacts, we intentionally disrupted autocrine signaling using a 5% reduction in secreted TGF-β in most simulations. This confers some reversibility to EMT, permitting a fluid coexistence of mesenchymal and hybrid E/M cells, the latter of which can divide. Yet, it is possible that *in vitro* the slow division of mostly mesenchymal cells is not due to temporary loss of TGF-β signaling (as modeled), but to the fact that even saturating levels of TGF-β do not fully block cell cycle entry in mesenchymal cells.

A second technical limitation, detailed in [86], relates to our Boolean approximation of ECM stiffness and cell density. There are three qualitatively distinct regimes for these inputs: no ECM/ soft ECM not capable of supporting stress fibers and growth/ stiff ECM that does. Similarly for density: no neighbors capable of making junctions/ some neighbors but also some space/ full confluency with no room to expand. Our Boolean model uses two linked nodes for each input, generating a 0/ low/ high state. This setup results in distinct signals in the 0 to low vs. the low to high regimes without overlap, and more rigid model responses near these boundaries than expected *in vitro.*

A different class of model limitations relates to the signaling biology we choose to include or ignore. *First,* the five experiments our models failed to match that did not contradict other data highlighted that at present our model is missing the molecular mechanism responsible for Hif-1⍺-mediated G2 arrest (our model only arrests in G1), as well as hypoxia-induced apoptosis (not modeled by choice). *Second*, we restricted our focus to the *acute* hypoxic response governed primarily by Hif-1α stabilization. Chronic hypoxia leads to a different set of responses our model does not cover, as they are induced by Hif-2α stabilization [136]. Despite some conflicting data, it is generally accepted that the third HIF subunit, Hif-3α, prevents Hif-1α transcriptional activity through sequestration of Hif-1β in a cell-type-specific manner [137,138]. Expansion of the model to include these Hif-α subunits is a promising future direction, required for modeling the temporal control of cell fates under hypoxia.

*Third,* Hif-1α is a metabolic regulator, yet we only included its effects on energy metabolism indirectly. The inclusion of a nutrient stimulus such as glucose, and the energy-sensing pathways that intersect with cell cycle entry modeled, in part, in [139] would allow for a more precise accounting of the hypoxic response. For example, apoptosis is differentially regulated based on nutrient loss in the presence or absence of Hif-1α, as Hif-1α prevents metabolic stress-induced apoptosis [95]. Glucose transporters GLUT1/3 are also upregulated in mesenchymal cells, demonstrating a shift towards glycolysis during EMT and suggesting a dependence of EMT on nutrient levels under hypoxia and/or normoxia [140]. A future expansion of our model could cover these responses, further mapping the environment combinations most conducive to EMT and MET.

*Fourth,* our model excludes several signaling pathways known to be differentially regulated under hypoxia, such as Wnt, Sonic Hedgehog (Shh), Notch, Interleukin-6, or TNF-α [141–148]. Thus, we operate under the implicit assumption that these signals are induced at equal levels in response to all stimuli tested within our model – even though their regulation may also be mechanosensitive. These signals are also implicated in mediating EMT, preventing apoptosis, and contributing to the spatial and temporal regulation of EMT in response to a changing TME [149]. Overall, the integration of these pathways may offer further contextual insight into cancer-associated behaviors increased by hypoxic tumor microenvironments.

The challenges of applying a cell-type agnostic model of hypoxia-induced EMT to specific carcinomas are at least three-fold. First, the most prevalent driver mutations and pathway alterations driving carcinoma vary substantially by tissue (e.g., VHL loss in ccRCC vs. BRAF^V600E^ mutations in melanoma). Moreover, our current model does not cover pathways that regulate genomic instability or immune evasion [150]. Second, there is substantial genetic diversity within tumors of the same type; not only among patients but also among cells from a single tumor [151,152]. Third, tumor growth is a cell population-level phenomenon, where cell-cell interactions go beyond contact inhibition or EMT-promoting signaling (modeled here), extending to signals from tumor-associated fibroblasts and immune cells (not modeled). Given these challenges, it is important to address: *i)* which, if any, tumor cell type do we expect our predictions to apply to, and *ii)* how can our model answer questions about specific cancers? *i)* Our predictions address mechano-sensitive responses such as EMT or proliferation under hypoxia (b,d,e) or the effects of specific mutations (a,c) in controlled conditions. Thus, we expect our predicted trends to hold *in vitro* in several carcinoma cell lines, with substantial variation in effect size and/or the environments the effects are strongest in (note that most mutation combinations only change a subset of model behaviors, if any; S7 Fig). *ii)* Given the challenges of predicting cancer cell behaviors *in vivo*, we expect our model to be of use in predicting the *range* of cell behaviors that mutations associated with a specific carcinoma can generate, in combination, in different microenvironments (e.g., S7 Fig for ccRCC), in response to chemotherapy/targeted therapy. These predicted responses can then be compared to single-cell RNA sequencing from tumors [153], analyzed with a focus on cell-behavior signatures rather than individual genes (e.g., EMT, cell cycle, apoptosis). This can help us pinpoint enriched behaviors compared to healthy tissues and predict mutation combinations that co-occur in single cells to drive tissue behavior (testable with DNA sequencing).

Single-cell transcriptomics can also identify key tissue-specific cancer cell signaling pathways missing from our model, pointing to cell-cell interactions we most need to account for. As an example, our *in silico* metastatic cascade is a simplification of the highly complex tumor and blood microenvironments experienced by circulating tumor cells. Extensive research has yielded both increasing and decreasing metastatic potential through interactions with neutrophils, cancer-associated fibroblasts (CAFs), macrophages, platelets, and other cell types, all of which are missing from our single-cell modeling approach [154–157]. Critically, interactions with platelets can upregulate TGF-β signaling and EMT, which help prevent cell death during the circulating phase [158].

The model boundaries highlighted above stem, in part, from our single-cell approach. We find this single cell focus valuable for mapping the rules that govern cell behavior in different microenvironments, a critical step towards building multiscale models of cell communities and tissue architectures needed to approach the complexity of tumor formation, tumor evolution, and metastatic disease. Long-term, our goal is two-fold. On the *single-cell* modeling side, we plan to integrate the model from this study with our Boolean model of mitochondrial dysfunction-associated senescence, which shares this model’s growth signaling, cell cycle, and apoptosis modules [139]. We will follow this with a detailed model of damage-induced cell cycle arrest mechanisms and a model of deep senescence, which can reverse mitochondrial dysfunction [159], but it is at least partially blocked in cells that underwent EMT [160,161]. The resulting single-cell model would be able to address most cell-autonomous cancer hallmarks. On the *tissue* modeling side, the above single-cell model can serve as the engine inside agents of the multicellular, spatial model of epithelial homeostasis and carcinoma development.

### Computational methods

I. ***Boolean model building.*** To build our model we extended our previously published mechanosensitive EMT model [86] with a hypoxia-sensing signaling module. The model synthesizes experimental data from 540 papers into a 165-node Boolean network with 763 regulatory links. All links and regulatory functions are experimentally justified in S1 Text. Our model construction approach, the rationale for using synchronous update, and storing our model in *Dynamically Modular Model Specification* (.*dmms*) format are detailed in the *Supplementary Methods* of [139] and STAR Methods of [86]. All model files and other files required to run the model and reproduce all results are packaged into S1-S3 Files.
II. ***Model availability.*** Model files in *SBML* format (used by BioModels [162], *GinSim* [163], and *The Cell Collective* [164]), *dmms* format (used by our discrete-state modeling software *dynmod*), *BooleanNet* format (used by the BooleanNet Python library [165]), and editable network visualization in *graphml* format (read by yED [166]) are included in S1 File.
III. **Dynmod *Boolean modeling software.*** Boolean simulations and analysis were performed with the discrete-state modeling software *dynmod* [139], available on GitHub at *https://github.com/Ravasz-Regan-Group/dynmod* (our justification for using in-house software, along with instructions to install Haskell and compile *dynmod* are detailed in the *Suppl. Methods* of [139]). Briefly, our code can: **a)** automatically map each attractor to a combinatorial phenotype profile (e.g., quiescent, alive, MiDAS) based on user-defined signatures attached to regulatory switches; **b)** visualize and filter attractors of interest via their phenotype profiles, organizing them within a coordinate system of independent environmental input-combinations; **c)** set up simulations by specifying the initial environment and phenotypic state of the cell, rather than each node state; **d)** collect phenotype statistics on large ensembles of non-interacting cells (independent simulation runs) in non-saturating environments and/or non-saturating perturbations (e.g., 10% Hypoxia, 50% VHL inhibition); **e)** generate simulation-series that explore the model’s behavior across a range of environmental inputs, combinations of 2–3 inputs, and/or the effects of increasing node knockdown/ hyper-activation (alone or in combination with changing environmental input levels; with/without additional background mutations); and **f)** use metadata from *dmms* files to generate a formatted table with all biological documentation (S1 Text).
  To run simulations (including attractor sampling), *dynmod* parses user-generated experiment files (*.vex* format). The S1 File package includes two*.vex* our readers can use to reproduce all simulations used in our figures (*Hypoxia_EMT_Main_Fig.vex, Hypoxia_EMT_SM_Fig.vex*). Similarly, the S2 File package contains the isolated EMT module model (*EMT_Module_Hypoxia.dmms*), the *EMT_Module.vex* file, and time courses that showcase the model attractors visualized in Fig 1B. Finally, S3 File contains the*.vex* file and script required for rerunning our validation experiments (see below). Additional information to run the simulations is available upon request from E.R.R.

IV. ***Attractor detection with* dynmod.** *Dynmod* uses synchronous update to find stable phenotypes and/or oscillations (attractors) via a stochastic sampling procedure [167] detailed in [86,168–170]. Briefly, we typically find attractors by running noisy time courses of length *T = 25* with noise *p = 0.02* from *N = 100* different random initial conditions for each unique combination of environmental inputs (*N*_total_ = 100*2^7^ = 128,000 random initial conditions). For each observed state along these time courses, the synchronous attractor basin is determined, recorded, and exported as a*.csv* file (*Greene_Hypoxia_Model_attractors_a100_25_2.0e-2.csv* in the S1 File package). To re-sample the model’s attractors, edit the *Sampling*{…} command block near the top of *Hypoxia_EMT_Main_Fig.vex* to switch from the currently active *Read:* directive to the commented-out *Sample:* line.
V. ***Running simulations with* dynmod.** Precise use of each command is described in *Hypoxia_EMT_Main_Fig.vex* and *Hypoxia_EMT_SM_Fig.vex* (in S1 File), which are executed by *dynmod* using the **-e** command-line tag:

dynmod Hypoxia_EMT_Model.dmms -e Hypoxia_EMT_Main_Fig.vex
dynmod Hypoxia_EMT_Model.dmms -e Hypoxia_EMT_SM_Fig.vex
  These files include instructions to simulate and visualize: a) synchronous time courses from a subset of cell states in a given initial environment, exposed to reversible changes in a single environmental signal (e.g., Fig 2A); b) non-saturating environments where an input is stochastically tuned between 0 and 1 (e.g., Fig 4A); c) partial or full knockdown/hyper-activation of arbitrary sets of nodes (e.g., Fig 3; justification and limitations in [86]); d) combine these in an arbitrary sequence of perturbations and environments defined in distinct time windows (e.g., Fig 6); e) average activity of all nodes and/or all module phenotypes in an ensemble of independent cells (e.g., Fig 2B); f) bar charts of the activity of user-specified nodes and/or module phenotypes, averaged over an ensemble of independent runs and across each distinct time window of an experiment (e.g., S3 Fig); g) bar charts and heatmaps showing the model’s behavior (module phenotype states) across a range of environments and/or node perturbations (e.g., S6 Fig).

VI. **Model validation.** To test the model’s ability to reproduce experimentally observed cell behaviors and/or molecular changes related to EMT in general and hypoxic responses in particular, we curated a list of 150 distinct observations from 13 relevant primary articles (S1 Table). These observations cover knockdown or overexpression of 19 different proteins or microRNAs. For each *in vitro* experiment, we created matching *in silico* experiments or experiment pairs that mimicked the extracellular environment and perturbations such as knockdowns or overexpression. This extensive validation protocol is included in *Hypoxia_EMT_Validation.vex* (in S3 File). Running

dynmod Hypoxia_EMT_Model.dmms -e Hypoxia_EMT_ Validation.vex
  generates simulation data on a population of cells for each experimental condition. To automate the 229 comparisons, each *in vitro* observation in S1 Table is accompanied by information about the name and location of its matching *in silico* data (experiment names, relevant time windows, code pointing to the correct attractor file). The Python script *Hypoxia Model Validation Script.py* reads a*.csv* version of S1 Table, as well as the simulation data, to perform unpaired t-tests, list, save, and summarize the validation results as the percentage of observations our model can reproduce (script and*.csv* in S3 File). Simulation results are considered a match for the experiment if either of the following conditions are met: *i)* there is a significant change in the same direction as the *in vitro* result, and the change is at least 5% of the average of the two values compared, or *ii)* no change, no significant change, or smaller than 5% difference, matching an *in vitro* result showing no significant change. The script also generates figures for each comparison (S12 Fig); the 17 failed comparisons identified by the script were manually marked with red/orange “*Mismatch*” labels (*red*: model does not match biological outcome; *orange*: different experiments give conflicting results; model matches the other outcome).

VII. **Metadata table, 2D network visualization, and model conversion.**

a) To generate a formatted table with all model metadata included in the.dmms file, run
  dynmod Hypoxia_EMT_Model.dmms -w -s
  This generates a text file with metadata warning (e.g., missing descriptions, citations, unrecognized node/link types), and a folder containing the LaTeX and BibTeX reference files used to render S1 Text. To generate the pdf, use LaTeX or *https://www.overleaf.com*.

b) To generate a.gml file read by the network visualization software yED run
  dynmod Hypoxia_EMT_Model.dmms -g
  The resulting visualization can be modified in yED to alter node coordinates and node colors. As long as none of the links or groupings are altered in the editing process, and the changes are saved in.gml format, running

dynmod Hypoxia_EMT_Model.dmms -u Hypoxia_EMT_Model.gml
  will read the altered coordinates and colors, then generate an updated.dmms file (with a new name). **Fig 1** was generated in yED by manual post-professing of the auto-generated.gml file (i.e., removal of groups, altered label colors for better contrast, creation of sub-networks).

c) To generate a.BooleanNet version of the model, run
  dynmod Hypoxia_EMT_Model.dmms -t
d) To generate the.sbml version, use *bioLQM* (http://colomoto.org/biolqm/) with the command:
  java -jar bioLQM-0.8-SNAPSHOT.jar Hypoxia_EMT_Model_Fine.booleannet Hypoxia_EMT_Model.sbml

VIII. **Previously introduced regulatory modules.** Detailed descriptions of *Growth factor signaling*, *Replication origin licensing*, *Restriction switch*, *Mitotic phase switch*, *Apoptotic switch*, *Cell cycle processes, Adhesion, Contact Inhibition, EMT* and TGF-β signaling are detailed in [86].

## Supporting information

S1 Fig Hif-1ɑ is required for proliferation under normoxia (full version of Fig 2A). (PDF)

S2 Fig Hif-1ɑ hyper-activation blocks the cell cycle by repressing Myc. (PDF)

S3 Fig Hypoxia induces EMT independently of TFG-β. (PDF)

S4 Fig A stiff ECM aids hypoxia-induced EMT, compared to hybrid E/M under normoxia. (PDF)

S5 Fig ECM stiffness boosts both hypoxia- and TGF-β-induced EMT; high levels of both are required to drive EMT on very soft ECMs. (PDF)

S6 Fig VHL deficiency boosts bio mechanically induced EMT in normoxia as well as hypoxia at moderate ECM stiffness and medium-high density, but abolishes cell cycle entry. (PDF)

S7 Fig VHL deficiency-induced cell cycle arrest is broken by cooperative hyper-activation of Myc and Cyclin D. (PDF)

S8 Fig Hypoxia confers anoikis resistance and protection from TGF-β induced epithelial cell apoptosis on a soft ECM (full version of Fig 5). (PDF)

S9 Fig Saturating autocrine TGF-β signaling protects all mesenchymal cells from anoikis, but also blocks MET on a stiff ECM. (PDF)

S10 Fig Slight breaks in the autocrine TGF-β signaling loop are potent inducers of anoikis and apoptosis on soft ECM. (PDF)

S11 Fig A changing mix of model cell phenotypes as a function of mitogen, cell density, ECM stiffness and hypoxia allows us to track TME-driven EMT and MET during the metastatic cascade. (PDF)

S12 Fig Auto-generated validation figures (150 panels with statistical tests, marked for match/ mismatch with expediting data). (PDF)

S1 Text Description and experimental support for all nodes and links of the Boolean Regulatory network model in Fig 1C. (PDF)

S1 Table List of 150 published experimental assays used for model validation, marked to indicate success/failure of the model in replicating each assay. (XLSX)

S1 File Files read by *dynmod* to simulate the Boolean model’s behaviors. These include the model in.dmms format (as well as.BooleanNet and.SBML formats, namely *Hypoxia_EMT_Model.dmms*, *Hypoxia_EMT_Model.booleannet, Hypoxia_EMT_Model.sbml*), the “in silico protocol” files for reproducing all figures (*Hypoxia_EMT_Main_Fig.vex, Hypoxia_EMT_SM_Fig.vex*) and the model network in.graphml format (*Hypoxia_EMT_Model.graphml*).
(ZIP)

S2 File Files read by *dynmod* to simulate the isolated EMT module. These include the EMT module model in.dmms format (*EMT_module_Hypoxia.dmms*) and an “in silico protocol” file for generating time courses to check the module’s attractors (*EMT_Module.vex*).
(ZIP)

S3 File Files read by *dynmod* to rerun all model validation experiments (*Hypoxia_EMT_Validation.vex*), the Pyhton3 script and.csv file that performs statistical tests on the simulation results, summarizes them, and generates the figures in S12 Fig (*Hypoxia Model Validation Script.py, ST2 - Hypoxia model validation list.csv*). (ZIP)

## References

[1] de Visser KE, Joyce JA. The evolving tumor microenvironment: From cancer initiation to metastatic outgrowth. Cancer Cell. 2023 Mar 13;41(3):374–403. 36917948 10.1016/j.ccell.2023.02.016

[2] Anderson N, Simon M. The tumor microenvironment. Current Biology. 2020 Aug 17; 30(16):R921–5. 10.1016/j.cub.2020.06.081 PMC8194051 32810447

[3] Cacho-Díaz B, García-Botello DR, Wegman-Ostrosky T, Reyes-Soto G, Ortiz-Sánchez E, Herrera-Montalvo LA. Tumor microenvironment differences between primary tumor and brain metastases. J Transl Med. 2020 Jan 3; 18(1):1. 31900168 10.1186/s12967-019-02189-8 PMC6941297

[4] Zeppellini A, Galimberti S, Leone BE, Pacifico C, Riva F, Cicchiello F, et al. Comparison of tumor microenvironment in primary and paired metastatic ER+/HER2- breast cancers: results of a pilot study. BMC Cancer. 2021 Mar 10;21(1):260. doi: 10.1186/s12885-021-07960-z 33691674 PMC7944604

[5] Kim R, Keam B, Kim S, Kim M, Kim SH, Kim JW, et al. Differences in tumor microenvironments between primary lung tumors and brain metastases in lung cancer patients: therapeutic implications for immune checkpoint inhibitors. BMC Cancer. 2019 Jan 7;19(1):19. 30616523 10.1186/s12885-018-5214-8 PMC6322302

[6] Neophytou CM, Panagi M, Stylianopoulos T, Papageorgis P. The Role of Tumor Microenvironment in Cancer Metastasis: Molecular Mechanisms and Therapeutic Opportunities. Cancers (Basel). 2021;13(9):2053. doi: 10.3390/cancers13092053 33922795 PMC8122975

[7] Dillekås H, Rogers MS, Straume O. Are 90% of deaths from cancer caused by metastases? Cancer Med. 2019 Aug 8;8(12):5574–6. 31397113 10.1002/cam4.2474 PMC6745820

[8] Son H, Moon A. Epithelial-mesenchymal Transition and Cell Invasion. Toxicol Res. 2010 Dec;26(4):245–52. doi: 10.5487/TR.2010.26.4.245 24278531 PMC3834497

[9] Dongre A, Weinberg RA. New insights into the mechanisms of epithelial-mesenchymal transition and implications for cancer. Nat Rev Mol Cell Biol. 2019;20(2):69–84. doi: 10.1038/s41580-018-0080-4 30459476

[10] Dudás J, Ladányi A, Ingruber J, Steinbichler TB, Riechelmann H. Epithelial to Mesenchymal Transition: A Mechanism that Fuels Cancer Radio/Chemoresistance. Cells. 2020 Feb 12; 9(2):428. 32059478 10.3390/cells9020428 PMC7072371

[11] Lu M, Jolly MK, Levine H, Onuchic JN, Ben-Jacob E. MicroRNA-based regulation of epithelial–hybrid–mesenchymal fate determination. Proc Natl Acad Sci USA. 2013 Nov 5;110(45):18144–9. 24154725 10.1073/pnas.1318192110 PMC3831488

[12] Tian X-J, Zhang H, Xing J. Coupled reversible and irreversible bistable switches underlying TGFβ-induced epithelial to mesenchymal transition. Biophys J. 2013 Aug;105(4):1079–89. doi: 10.1016/j.bpj.2013.07.011 23972859 PMC3752104

[13] Siemens H, Jackstadt R, Hünten S, Kaller M, Menssen A, Götz U, et al. miR-34 and SNAIL form a double-negative feedback loop to regulate epithelial-mesenchymal transitions. Cell Cycle. 2011 Dec 15;10(24):4256–71. doi: 10.4161/cc.10.24.18552 22134354

[14] Gregory PA, Bracken CP, Smith E, Bert AG, Wright JA, Roslan S, et al. An autocrine TGF-beta/ZEB/miR-200 signaling network regulates establishment and maintenance of epithelial-mesenchymal transition. Mol Biol Cell. 2011 May 15; 22(10):1686–98. 21411626 10.1091/mbc.E11-02-0103 PMC3093321

[15] Zhang J, Tian XJ, Zhang H, Teng Y, Li R, Bai F, et al. TGF-β-induced epithelial-to-mesenchymal transition proceeds through stepwise activation of multiple feedback loops. Science Signaling. 2014 Sep;7(345):ra91. 10.1126/scisignal.2005304 25270257

[16] Grosse-Wilde A, Fouquier d’Hérouël A, McIntosh E, Ertaylan G, Skupin A, Kuestner RE, et al. Stemness of the hybrid Epithelial/Mesenchymal State in Breast Cancer and Its Association with Poor Survival. PLoS One. 2015;10(5):e0126522. doi: 10.1371/journal.pone.0126522 26020648 PMC4447403

[17] Huang RYJ, Wong MK, Tan TZ, Kuay KT, Ng AHC, Chung VY, et al. An EMT spectrum defines an anoikis-resistant and spheroidogenic intermediate mesenchymal state that is sensitive to e-cadherin restoration by a src-kinase inhibitor, saracatinib (AZD0530). Cell Death Dis. 2013 Nov 7;4:e915. 10.1038/cddis.2013.442 PMC3847320 24201814

[18] Jolly MK, Tripathi SC, Jia D, Mooney SM, Celiktas M, Hanash SM, et al. Stability of the hybrid epithelial/mesenchymal phenotype. Oncotarget. 2016 Mar 17;7(19):27067–84. 27008704 10.18632/oncotarget.8166 PMC5053633

[19] Dong J, Hu Y, Fan X, Wu X, Mao Y, Hu B, et al. Single-cell RNA-seq analysis unveils a prevalent epithelial/mesenchymal hybrid state during mouse organogenesis. Genome Biol. 2018 Mar 14;19(1):31. doi: 10.1186/s13059-018-1416-2 29540203 PMC5853091

[20] Gonzalez VD, Samusik N, Chen TJ, Savig ES, Aghaeepour N, Quigley DA, et al. Commonly Occurring Cell Subsets in High-Grade Serous Ovarian Tumors Identified by Single-Cell Mass Cytometry. Cell Rep. 2018 Feb 13;22(7):1875–88. 29444438 10.1016/j.celrep.2018.01.053 PMC8556706

[21] Karacosta LG, Anchang B, Ignatiadis N, Kimmey SC, Benson JA, Shrager JB, et al. Mapping lung cancer epithelial-mesenchymal transition states and trajectories with single-cell resolution. Nat Commun. 2019 Dec 6;10(1):5587. doi: 10.1038/s41467-019-13441-6 31811131 PMC6898514

[22] Puram S, Tirosh I, Parikh A, Patel A, Yizhak K, Gillespie S, et al. Single-cell transcriptomic analysis of primary and metastatic tumor ecosystems in head and neck cancer. Cell. 2017 Dec 14; 171(7):1611–24.e24 29198524 10.1016/j.cell.2017.10.044 PMC5878932

[23] Biswas K, Jolly MK, Ghosh A. Stability and mean residence times for hybrid epithelial/mesenchymal phenotype. Phys Biol. 2019 Jan 29;16(2):025003. doi: 10.1088/1478-3975/aaf7b7 30537698

[24] Hong T, Watanabe K, Ta CH, Villarreal-Ponce A, Nie Q, Dai X. An Ovol2-Zeb1 Mutual Inhibitory Circuit Governs Bidirectional and Multi-step Transition between Epithelial and Mesenchymal States. PLoS Comput Biol. 2015 Nov 10;11(11):e1004569. 10.1371/journal.pcbi.1004569 PMC4640575 26554584

[25] Bocci F, Gearhart-Serna L, Boareto M, Ribeiro M, Ben-Jacob E, Devi GR, et al. Toward understanding cancer stem cell heterogeneity in the tumor microenvironment Proc Natl Acad Sci U S A. 2019 Jan 2;116(1:)148–57. 30587589 10.1073/pnas.1815345116 PMC6320545

[26] Tripathi S, Chakraborty P, Levine H, Jolly MK. A mechanism for epithelial-mesenchymal heterogeneity in a population of cancer cells. PLoS Comput Biol. 2020 Feb;16(2):e1007619. doi: 10.1371/journal.pcbi.1007619 32040502 PMC7034928

[27] Steinway SN, Zañudo JGT, Ding W, Rountree CB, Feith DJ, Loughran TP, et al. Network modeling of TGFβ signaling in hepatocellular carcinoma epithelial-to-mesenchymal transition reveals joint sonic hedgehog and Wnt pathway activation. Cancer Research. 2014 Nov;74(21):5963–77. 25189528 10.1158/0008-5472.CAN-14-0225 PMC4851164

[28] Gómez Tejeda Zañudo J, Guinn MT, Farquhar K, Szenk M, Steinway SN, Balázsi G, et al. Towards control of cellular decision-making networks in the epithelial-to-mesenchymal transition. Phys Biol. 2019;16(3):031002. doi: 10.1088/1478-3975/aaffa1 30654341 PMC6405305

[29] Steinway SN, Zañudo JGT, Michel PJ, Feith DJ, Loughran TP, Albert R. Combinatorial interventions inhibit TGFβ-driven epithelial-to-mesenchymal transition and support hybrid cellular phenotypes. NPJ Syst Biol Appl. 2015;1:15014. doi: 10.1038/npjsba.2015.14 28725463 PMC5516807

[30] Li Y, Zhao L, Li XF. Hypoxia and the Tumor Microenvironment. Technol Cancer Res Treat. 2021 Aug 5;20:15330338211036304. 34350796 10.1177/15330338211036304 PMC8358492

[31] Jiang BH, Semenza GL, Bauer C, Marti HH. Hypoxia-inducible factor 1 levels vary exponentially over a physiologically relevant range of O2 tension. American Journal of Physiology-Cell Physiology. 1996 Oct;271(4):C1172–80. doi: 10.1152/ajpcell.1996.271.4.C1172 8897823

[32] Krock BL, Skuli N, Simon MC. Hypoxia-Induced Angiogenesis. Genes Cancer. 2011 Dec;2(12):1117–33. doi: 10.1177/1947601911423654 22866203 PMC3411127

[33] Saxena K, Jolly MK, Balamurugan K. Hypoxia, partial EMT and collective migration: Emerging culprits in metastasis. Transl Oncol. 2020 Aug 8; 13(11):100845. 32781367 10.1016/j.tranon.2020.100845 PMC7419667

[34] Jeltsch M, Leppänen V-M, Saharinen P, Alitalo K. Receptor tyrosine kinase-mediated angiogenesis. Cold Spring Harb Perspect Biol. 2013 Sep;5(9):a009183. doi: 10.1101/cshperspect.a009183 24003209 PMC3753715

[35] Jiang X, Wang J, Deng X, Xiong F, Zhang S, Gong Z, et al. The role of microenvironment in tumor angiogenesis. J Exp Clin Cancer Res. 2020 Sep 30;39(1):204. doi: 10.1186/s13046-020-01709-5 32993787 PMC7526376

[36] Cichon MA, Nelson CM, Radisky DC. Regulation of Epithelial-Mesenchymal Transition in Breast Cancer Cells by Cell Contact and Adhesion. Cancer Inform. 2015 Feb 9; 14(Suppl 3):1–13. 10.4137/CIN.S18965 PMC4325704 25698877

[37] O’Connor JW, Mistry K, Detweiler D, Wang C, Gomez EW. Cell-cell contact and matrix adhesion promote αSMA expression during TGFβ1-induced epithelial-myofibroblast transition via Notch and MRTF-A. Sci Rep. 2016 May 19; 6(1):26226. 27194451 10.1038/srep26226 PMC4872162

[38] Rice AJ, Cortes E, Lachowski D, Cheung BCH, Karim SA, Morton JP, et al. Matrix stiffness induces epithelial-mesenchymal transition and promotes chemoresistance in pancreatic cancer cells. Oncogenesis. 2017 Jul 3;6(7):e352. 10.1038/oncsis.2017.54 PMC5541706 28671675

[39] Tian H, Shi H, Yu J, Ge S, Ruan J. Biophysics Role and Biomimetic Culture Systems of ECM Stiffness in Cancer EMT. Glob Chall. 2022 Mar 20; 6(6):2100094. 35712024 10.1002/gch2.202100094 PMC9189138

[40] Aldea M, Andre F, Marabelle A, Dogan S, Barlesi F, Soria J-C. Overcoming Resistance to Tumor-Targeted and Immune-Targeted Therapies. Cancer Discovery. 2021 Apr 1;11(4):874–99. doi: 10.1158/2159-8290.CD-20-1638 33811122

[41] Taeb S, Ashrafizadeh M, Zarrabi A, Rezapoor S, Musa AE, Farhood B, et al. Role of Tumor Microenvironment in Cancer Stem Cells Resistance to Radiotherapy. Current Cancer Drug Targets. 2022 Jan 1;22(1):18–30. 34951575 10.2174/1568009622666211224154952

[42] Zhao D, Mo Y, Neganova ME, Aleksandrova Y, Tse E, Chubarev VN, et al. Dual effects of radiotherapy on tumor microenvironment and its contribution towards the development of resistance to immunotherapy in gastrointestinal and thoracic cancers. Front Cell Dev Biol. 2023 Oct 3; 11:1266537. 37849740 10.3389/fcell.2023.1266537 PMC10577389

[43] Luqmani YA Mechanisms of Drug Resistance in Cancer Chemotherapy. Medical Principles and Practice. 2008 Jul 9;14(Suppl. 1):35–48. 16103712 10.1159/000086183

[44] Fletcher NM, Belotte J, Saed MG, Memaj I, Diamond MP, Morris RT, et al. Specific point mutations in key redox enzymes are associated with chemoresistance in epithelial ovarian cancer. Free Radic Biol Med. 2017;102:122–32. doi: 10.1016/j.freeradbiomed.2016.11.028 27890641

[45] Appelhoff RJ, Tian YM, Raval RR, Turley H, Harris AL, Pugh CW, et al. Differential Function of the Prolyl Hydroxylases PHD1, PHD2, and PHD3 in the Regulation of Hypoxia-inducible Factor *. Journal of Biological Chemistry. 2004 Sep 10;279(37):38458–65. 15247232 10.1074/jbc.M406026200

[46] Peng J, Wang X, Ran L, Song J, Luo R, Wang Y. Hypoxia-Inducible Factor 1α Regulates the Transforming Growth Factor β1/SMAD Family Member 3 Pathway to Promote Breast Cancer Progression. J Breast Cancer. 2018 Sep;21(3):259–66. 10.4048/jbc.2018.21.e42 PMC6158164 30275854

[47] Ananth S, Knebelmann B, Grüning W, Dhanabal M, Walz G, Stillman IE, et al. Transforming growth factor beta1 is a target for the von Hippel-Lindau tumor suppressor and a critical growth factor for clear cell renal carcinoma. Cancer Res. 1999 May 1;59(9):2210–6. 10232610

[48] Fong G-H, Takeda K. Role and regulation of prolyl hydroxylase domain proteins. Cell Death Differ. 2008;15(4):635–41. doi: 10.1038/cdd.2008.10 18259202

[49] Kamura T, Sato S, Iwai K, Czyzyk-Krzeska M, Conaway RC, Conaway JW. Activation of HIF1α ubiquitination by a reconstituted von Hippel-Lindau (VHL) tumor suppressor complex. Proc Natl Acad Sci U S A. 2000 Sep 12; 97(19):10430–5. 10973499 10.1073/pnas.190332597 PMC27041

[50] Lando D, Peet DJ, Gorman JJ, Whelan DA, Whitelaw ML, Bruick RK. FIH-1 is an asparaginyl hydroxylase enzyme that regulates the transcriptional activity of hypoxia-inducible factor. Genes Dev. 2002;16(12):1466–71. doi: 10.1101/gad.991402 12080085 PMC186346

[51] Prabhakar NR, Semenza GL. Adaptive and maladaptive cardiorespiratory responses to continuous and intermittent hypoxia mediated by hypoxia-inducible factors 1 and 2. Physiol Rev. 2012 Jul;92(3):967–1003. 22811423 10.1152/physrev.00030.2011 PMC3893888

[52] Semenza GL. HIF-1 and mechanisms of hypoxia sensing. Current Opinion in Cell Biology. 2001 Apr 1;13(2):167–71. 11248550 10.1016/s0955-0674(00)00194-0

[53] Prabhakar NR, Semenza GL. Adaptive and maladaptive cardiorespiratory responses to continuous and intermittent hypoxia mediated by hypoxia-inducible factors 1 and 2. Physiol Rev. 2012 Jul;92(3):967–1003. doi: 10.1152/physrev.00030.2011 22811423 PMC3893888

[54] Koh MY, Lemos R, Liu X, Powis G. The hypoxia-associated factor switches cells from HIF-1α to HIF-2α dependent signaling promoting stem cell characteristics aggressive tumor growth and invasion. Cancer Res. 2011;71(11):4015–27. doi: 10.1158/0008-5472.CAN-10-4142 21512133 PMC3268651

[55] Makino Y, Uenishi R, Okamoto K, Isoe T, Hosono O, Tanaka H, et al. Transcriptional Up-regulation of Inhibitory PAS Domain Protein Gene Expression by Hypoxia-inducible Factor 1 (HIF-1): a negative feedback regulatory circuit in HIF-1-mediated signaling in hypoxic cells. Journal of Biological Chemistry. 2007 May 11;282(19):14073–82. 17355974 10.1074/jbc.M700732200

[56] Duan C. Hypoxia-inducible factor 3 biology: complexities and emerging themes. Am J Physiol Cell Physiol. 2016 Feb 15;310(4):C260-269. 10.1152/ajpcell.00315.2015 26561641

[57] Li QF, Wang XR, Yang YW, Lin H. Hypoxia upregulates hypoxia inducible factor (HIF)-3alpha expression in lung epithelial cells: characterization and comparison with HIF-1alpha. Cell Res. 2006;16(6):548–58. doi: 10.1038/sj.cr.7310072 16775626

[58] Main AM, Gillberg L, Jacobsen AL, Nilsson E, Gjesing AP, Hansen T, et al. DNA methylation and gene expression of HIF3A: cross-tissue validation and associations with BMI and insulin resistance. Clin Epigenetics. 2016 Sep 2;8(1):89. doi: 10.1186/s13148-016-0258-6 27594926 PMC5010678

[59] Benita Y, Kikuchi H, Smith AD, Zhang MQ, Chung DC, Xavier RJ. An integrative genomics approach identifies Hypoxia Inducible Factor-1 (HIF-1)-target genes that form the core response to hypoxia. Nucleic Acids Res. 2009;37(14):4587–602. doi: 10.1093/nar/gkp425 19491311 PMC2724271

[60] Wang V, Davis DA, Haque M, Huang LE, Yarchoan R. Differential gene up-regulation by hypoxia-inducible factor-1α and hypoxia-inducible factor-2α in HEK293T cells. Cancer Research. 2005 Apr 15;65(8):3299–306. doi: 10.1158/0008-5472.CAN-04-4130 15833863

[61] Manalo DJ, Rowan A, Lavoie T, Natarajan L, Kelly BD, Ye SQ, et al. Transcriptional regulation of vascular endothelial cell responses to hypoxia by HIF-1. Blood. 2005 Jan 15;105(2):659–69. 15374877 10.1182/blood-2004-07-2958

[62] Zhang L, Huang G, Li X, Zhang Y, Jiang Y, Shen J, et al. Hypoxia induces epithelial-mesenchymal transition via activation of SNAI1 by hypoxia-inducible factor -1α in hepatocellular carcinoma. BMC Cancer. 2013 Mar 9; 13:108. 23496980 10.1186/1471-2407-13-108 PMC3614870

[63] Zhang W, Shi X, Peng Y, Wu M, Zhang P, Xie R, et al. HIF-1α Promotes Epithelial-Mesenchymal Transition and Metastasis through Direct Regulation of ZEB1 in Colorectal Cancer. PLOS ONE. 2015 Jun 9;10(6):e0129603. doi: 10.1371/journal.pone.0129603 26057751 PMC4461314

[64] Schödel J, Oikonomopoulos S, Ragoussis J, Pugh CW, Ratcliffe PJ, Mole DR. High-resolution genome-wide mapping of HIF-binding sites by ChIP-seq. Blood. 2011 Jun 9;117(23):e207–17. 10.1182/blood-2010-10-314427 PMC3374576 21447827

[65] Morikawa M, Koinuma D, Miyazono K, Heldin C-H. Genome-wide mechanisms of Smad binding. Oncogene. 2013;32(13):1609–15. doi: 10.1038/onc.2012.191 22614010 PMC3615190

[66] Mallikarjuna P, Sitaram RT, Landström M, Ljungberg B. VHL status regulates transforming growth factor-β signaling pathways in renal cell carcinoma. Oncotarget. 2018 Mar 27;9(23):16297–310. 29662646 10.18632/oncotarget.24631 PMC5893241

[67] Dai Y, Siemann D. c-Src is required for hypoxia-induced metastasis-associated functions in prostate cancer cells. Onco Targets Ther. 2019 May 8;12:3519–29. 31190858 10.2147/OTT.S201320 PMC6512571

[68] Guo Q, Lu L, Liao Y, Wang X, Zhang Y, Liu Y, et al. Influence of c-Src on hypoxic resistance to paclitaxel in human ovarian cancer cells and reversal of FV-429. Cell Death Dis. 2018 Jan;8(1):e3178. doi: 10.1038/cddis.2017.367 29324735 PMC5827169

[69] Lee G, Won HS, Lee YM, Choi JW, Oh TI, Jang JH, et al. Oxidative Dimerization of PHD2 is Responsible for its Inactivation and Contributes to Metabolic Reprogramming via HIF-1α Activation. Sci Rep. 2016 Jan 7; 6(1):18928. 26740011 10.1038/srep18928 PMC4703963

[70] Hu X, Wu X, Xu J, Zhou J, Han X, Guo J. Src kinase up-regulates the ERK cascade through inactivation of protein phosphatase 2A following cerebral ischemia. BMC Neurosci. 2009 Jul 14; 10:74. 19602257 10.1186/1471-2202-10-74 PMC2714518

[71] Owens DW, McLean GW, Wyke AW, Paraskeva C, Parkinson EK, Frame MC, et al. The catalytic activity of the Src family kinases is required to disrupt cadherin-dependent cell-cell contacts. Mol Biol Cell. 2000;11(1):51–64. doi: 10.1091/mbc.11.1.51 10637290 PMC14756

[72] Zhang X, Guo J, Jabbarzadeh Kaboli P, Zhao Q, Xiang S, Shen J, et al. Analysis of Key Genes Regulating the Warburg Effect in Patients with Gastrointestinal Cancers and Selective Inhibition of This Metabolic Pathway in Liver Cancer Cells. Onco Targets Ther. 2020 Jul 27;13:7295–304. 32801756 10.2147/OTT.S257944 PMC7394593

[73] Goda N, Ryan HE, Khadivi B, McNulty W, Rickert RC, Johnson RS. Hypoxia-inducible factor 1alpha is essential for cell cycle arrest during hypoxia. Mol Cell Biol. 2003;23(1):359–69. doi: 10.1128/MCB.23.1.359-369.2003 12482987 PMC140666

[74] Koshiji M, Kageyama Y, Pete EA, Horikawa I, Barrett JC, Huang LE. HIF-1α induces cell cycle arrest by functionally counteracting Myc. EMBO J. 2004 May 5; 23(9):1949–56. 15071503 10.1038/sj.emboj.7600196 PMC404317

[75] Chen Y, Yan H, Yan L, Wang X, Che X, Hou K, et al. Hypoxia-induced ALDH3A1 promotes the proliferation of non-small-cell lung cancer by regulating energy metabolism reprogramming. Cell Death Dis. 2023 Sep 20; 14(9):1–11. 37730658 10.1038/s41419-023-06142-y PMC10511739

[76] Whelan KA, Caldwell SA, Shahriari KS, Jackson SR, Franchetti LD, Johannes GJ, et al. Hypoxia Suppression of Bim and Bmf Blocks Anoikis and Luminal Clearing during Mammary Morphogenesis. Mol Biol Cell. 2010 Nov 15;21(22):3829–37. 20861305 10.1091/mbc.E10-04-0353 PMC2982135

[77] Rohwer N, Welzel M, Daskalow K, Pfander D, Wiedenmann B, Detjen K, et al. Hypoxia-Inducible Factor 1α Mediates Anoikis Resistance via Suppression of α5 Integrin. Cancer Research. 2008 Dec 15; 68(24):10113–20. 19074877 10.1158/0008-5472.CAN-08-1839

[78] Saikumar P, Dong Z, Patel Y, Hall K, Hopfer U, Weinberg JM, et al. Role of hypoxia-induced Bax translocation and cytochrome c release in reoxygenation injury. Oncogene. 1998;17(26):3401–15. doi: 10.1038/sj.onc.1202590 10030664

[79] Greijer AE, van der Wall E. The role of hypoxia inducible factor 1 (HIF-1) in hypoxia induced apoptosis. J Clin Pathol. 2004;57(10):1009–14. doi: 10.1136/jcp.2003.015032 15452150 PMC1770458

[80] Li H-S, Zhou Y-N, Li L, Li S-F, Long D, Chen X-L, et al. HIF-1α protects against oxidative stress by directly targeting mitochondria. Redox Biol. 2019 Jul;25:101109. doi: 10.1016/j.redox.2019.101109 30686776 PMC6859547

[81] Dong Z, Venkatachalam MA, Wang J, Patel Y, Saikumar P, Semenza GL, et al. Up-regulation of apoptosis inhibitory protein IAP-2 by hypoxia. Hif-1-independent mechanisms. J Biol Chem. 2001 Jun 1; 276(22):18702–9. 11278985 10.1074/jbc.M011774200 PMC2854569

[82] Dagogo-Jack I, Shaw AT. Tumour heterogeneity and resistance to cancer therapies. Nat Rev Clin Oncol. 2018 Feb;15(2):81–94. doi: 10.1038/nrclinonc.2017.166 29115304

[83] Jacquemin V, Antoine M, Dom G, Detours V, Maenhaut C, Dumont JE. Dynamic Cancer Cell Heterogeneity: Diagnostic and Therapeutic Implications. Cancers (Basel). 2022 Jan 7;14(2):280. 35053446 10.3390/cancers14020280 PMC8773841

[84] Simeonov KP, Byrns CN, Clark ML, Norgard RJ, Martin B, Stanger BZ, et al. Single-cell lineage tracing of metastatic cancer reveals selection of hybrid EMT states. Cancer Cell. 2021 Aug 9;39(8):1150–62.e9. 34115987 10.1016/j.ccell.2021.05.005 PMC8782207

[85] Zhang Q, Fei L, Han R, Huang R, Wang Y, Chen H, et al. Single-cell transcriptome reveals cellular hierarchies and guides p-EMT-targeted trial in skull base chordoma. Cell Discov. 2022 Sep 20;8(1):94. doi: 10.1038/s41421-022-00459-2 36127333 PMC9489773

[86] Sullivan E, Harris M, Bhatnagar A, Guberman E, Zonfa I, Ravasz Regan E. Boolean modeling of mechanosensitive epithelial to mesenchymal transition and its reversal. iScience. 2023 Apr 21;26(4):106321. doi: 10.1016/j.isci.2023.106321 36968076 PMC10030917

[87] Schwab LP, Peacock DL, Majumdar D, Ingels JF, Jensen LC, Smith KD, et al. Hypoxia-inducible factor 1α promotes primary tumor growth and tumor-initiating cell activity in breast cancer. Breast Cancer Res. 2012;14(1):R6. doi: 10.1186/bcr3087 22225988 PMC3496121

[88] D’Uva G, Bertoni S, Lauriola M, De Carolis S, Pacilli A, D’Anello L, et al. Beta-catenin/HuR post-transcriptional machinery governs cancer stem cell features in response to hypoxia. PLoS One. 2013 Nov 15;8(11):e80742. doi: 10.1371/journal.pone.0080742 24260469 PMC3829939

[89] Yang MH, Wu MZ, Chiou SH, Chen PM, Chang SY, Liu CJ, et al. Direct regulation of TWIST by HIF-1alpha promotes metastasis. Nat Cell Biol. 2008 Mar;10(3):295–305. 18297062 10.1038/ncb1691

[90] Lv Y, Chen C, Zhao B, Zhang X. Regulation of matrix stiffness on the epithelial-mesenchymal transition of breast cancer cells under hypoxia environment. Naturwissenschaften. 2017 Jun;104(5–6):38. 28382476 10.1007/s00114-017-1461-9

[91] Uchida T, Rossignol F, Matthay MA, Mounier R, Couette S, Clottes E, et al. Prolonged hypoxia differentially regulates hypoxia-inducible factor (HIF)-1alpha and HIF-2alpha expression in lung epithelial cells: implication of natural antisense HIF-1alpha. J Biol Chem. 2004 Apr 9;279(15):14871–8. doi: 10.1074/jbc.M400461200 14744852

[92] Kaidi A, Williams AC, Paraskeva C. Interaction between beta-catenin and HIF-1 promotes cellular adaptation to hypoxia. Nat Cell Biol. 2007;9(2):210–7. doi: 10.1038/ncb1534 17220880

[93] Yeung SJ, Pan J, Lee MH. Roles of p53, Myc and HIF-1 in Regulating Glycolysis — the Seventh Hallmark of Cancer. Cell Mol Life Sci. 2008 Sep 3; 65(24):3981. 18766298 10.1007/s00018-008-8224-x PMC11131737

[94] Horváthová J, Moravčík R, Matúšková M, Šišovský V, Boháč A, Zeman M. Inhibition of Glycolysis Suppresses Cell Proliferation and Tumor Progression In Vivo: Perspectives for Chronotherapy. Int J Mol Sci. 2021 Apr 22; 22(9):4390. 33922320 10.3390/ijms22094390 PMC8122821

[95] Belapurkar R, Pfisterer M, Dreute J, Werner S, Zukunft S, Fleming I, et al. A transient increase of HIF-1α during the G1 phase (G1-HIF) ensures cell survival under nutritional stress. Cell Death Dis. 2023 Jul 27;14(7):1–15. 37500648 10.1038/s41419-023-06012-7 PMC10374543

[96] Green SL, Freiberg RA, Giaccia AJ. p21(Cip1) and p27(Kip1) regulate cell cycle reentry after hypoxic stress but are not necessary for hypoxia-induced arrest. Mol Cell Biol. 2001 Feb;21(4):1196–206. 11158306 10.1128/MCB.21.4.1196-1206.2001 PMC99573

[97] Luo Y, Li M, Zuo X, Basourakos SP, Zhang J, Zhao J, et al. β‑catenin nuclear translocation induced by HIF‑1α overexpression leads to the radioresistance of prostate cancer. Int J Oncol. 2018 Jun;52(6):1827–40. 29658569 10.3892/ijo.2018.4368 PMC5919719

[98] Cummins E, Berra E, Comerford K, Ginouves A, Fitzgerald K, Seeballuck F, et al. Prolyl hydroxylase-1 negatively regulates IκB kinase-β, giving insight into hypoxia-induced NFκB activity Proc Natl Acad Sci U S A. 2006 Nov 28;103(48):18154–9. 17114296 10.1073/pnas.0602235103 PMC1643842

[99] McMahon S, Grondin F, McDonald PP, Richard DE, Dubois CM. Hypoxia-enhanced expression of the proprotein convertase furin is mediated by hypoxia-inducible factor-1: impact on the bioactivation of proproteins. J Biol Chem. 2005;280(8):6561–9. doi: 10.1074/jbc.M413248200 15611046

[100] Matsuoka J, Yashiro M, Doi Y, Fuyuhiro Y, Kato Y, Shinto O, et al. Hypoxia stimulates the EMT of gastric cancer cells through autocrine TGFβ signaling. PLOS ONE. 2013 May 17;8(5):e62310. doi: 10.1371/journal.pone.0062310 23690936 PMC3656884

[101] Mallikarjuna P, Raviprakash TS, Aripaka K, Ljungberg B, Landström M. Interactions between TGF-β type I receptor and hypoxia-inducible factor-α mediates a synergistic crosstalk leading to poor prognosis for patients with clear cell renal cell carcinoma. Cell Cycle. 2019;18(17):2141–56. doi: 10.1080/15384101.2019.1642069 31339433 PMC6986558

[102] Brereton CJ, Yao L, Davies ER, Zhou Y, Vukmirovic M, Bell JA, et al. Pseudohypoxic HIF pathway activation dysregulates collagen structure-function in human lung fibrosis. eLife. 11:e69348. 10.7554/eLife.69348 PMC8860444 35188460

[103] Muñoz-Nájar UM, Neurath KM, Vumbaca F, Claffey KP. Hypoxia stimulates breast carcinoma cell invasion through MT1-MMP and MMP-2 activation. Oncogene. 2006 Apr 13;25(16):2379–92. 16369494 10.1038/sj.onc.1209273

[104] Choi JY, Jang YS, Min SY, Song JY. Overexpression of MMP-9 and HIF-1α in Breast Cancer Cells under Hypoxic Conditions. J Breast Cancer. 2011 Jun;14(2):88–95. doi: 10.4048/jbc.2011.14.2.88 21847402 PMC3148536

[105] Park JS, Burckhardt CJ, Lazcano R, Solis LM, Isogai T, Li L, et al. Mechanical regulation of glycolysis via cytoskeleton architecture. Nature. 2020;578(7796):621–6. doi: 10.1038/s41586-020-1998-1 32051585 PMC7210009

[106] Gizak A, Wiśniewski J, Heron P, Mamczur P, Sygusch J, Rakus D. Targeting a moonlighting function of aldolase induces apoptosis in cancer cells. Cell Death Dis. 2019 Sep 26;10(10):712. 31558701 10.1038/s41419-019-1968-4 PMC6763475

[107] van Leeuwaarde RS, Ahmad S, van Nesselrooij B, Zandee W, Giles RH. Von Hippel-Lindau Syndrome. In: Adam MP, Feldman J, Mirzaa GM, Pagon RA, Wallace SE, Bean LJ, et al., Editors. GeneReviews. Seattle (WA): University of Washington; 1993. Available from: http://www.ncbi.nlm.nih.gov/books/NBK1463/. 20301636

[108] Ferlay J, Colombet M, Soerjomataram I, Dyba T, Randi G, Bettio M, et al. Cancer incidence and mortality patterns in Europe: Estimates for 40 countries and 25 major cancers in 2018. Eur J Cancer. 2018 Nov;103:356–87. doi: 10.1016/j.ejca.2018.07.005 30100160

[109] Clark PE. The role of VHL in clear-cell renal cell carcinoma and its relation to targeted therapy. Kidney International. 2009 Nov 1;76(9):939–45. 19657325 10.1038/ki.2009.296 PMC2963106

[110] Pritchett TL, Bader HL, Henderson J, Hsu T. Conditional inactivation of the mouse von Hippel-Lindau tumor suppressor gene results in wide-spread hyperplastic, inflammatory and fibrotic lesions in the kidney. Oncogene. 2015;34(20):2631–9. doi: 10.1038/onc.2014.197 25023703

[111] Wang S-S, Gu Y-F, Wolff N, Stefanius K, Christie A, Dey A, et al. Bap1 is essential for kidney function and cooperates with Vhl in renal tumorigenesis. Proceedings of the National Academy of Sciences. 2014 Nov 18;111(46):16538–43. doi: 10.1073/pnas.1414789111 25359211 PMC4246264

[112] Xu Z, Liu L, Jiang W, Qiu Y, Zhang B, Cheng J, et al. VHL missense mutation delineate aggressive clear cell renal cell carcinoma subtype with favorable immunotherapeutic response. J Immunother Cancer. 2024 Oct 23;12(10):e009963. doi: 10.1136/jitc-2024-009963 39448203 PMC11499804

[113] Gordan JD, Lal P, Dondeti VR, Letrero R, Parekh KN, Oquendo CE, et al. HIF-alpha effects on c-Myc distinguish two subtypes of sporadic VHL-deficient clear cell renal carcinoma. Cancer Cell. 2008;14(6):435–46. doi: 10.1016/j.ccr.2008.10.016 19061835 PMC2621440

[114] Lin P-H, Huang C-Y, Yu K-J, Kan H-C, Liu C-Y, Chuang C-K, et al. Genomic characterization of clear cell renal cell carcinoma using targeted gene sequencing. Oncol Lett. 2021;21(2):169. doi: 10.3892/ol.2021.12430 33456545 PMC7802514

[115] Adeshakin F, Adeshakin A, Afolabi L, Yan D, Zhang G, Wan X. Mechanisms for Modulating Anoikis Resistance in Cancer and the Relevance of Metabolic Reprogramming. Front Oncol. 2021 Mar 29;11:626577. 33854965 10.3389/fonc.2021.626577 PMC8039382

[116] Dong Z, Wang JZ, Yu F, Venkatachalam MA. Apoptosis-Resistance of Hypoxic Cells. Am J Pathol. 2003 Aug;163(2):663–71. 12875985 10.1016/S0002-9440(10)63693-0 PMC1868200

[117] Park SH, Riley P, Frisch SM. Regulation of anoikis by deleted in breast cancer-1 (DBC1) through NF-κB. Apoptosis. 2013 Aug;18(8):949–62. doi: 10.1007/s10495-013-0847-1 23588592 PMC3691317

[118] Wang CY, Guttridge DC, Mayo MW, Baldwin AS. NF-κB Induces Expression of the Bcl-2 Homologue A1/Bfl-1 To Preferentially Suppress Chemotherapy-Induced Apoptosis. Mol Cell Biol. 1999 Sep;19(9):5923–9. 10454539 10.1128/mcb.19.9.5923 PMC84448

[119] Tilghman RW, Cowan CR, Mih JD, Koryakina Y, Gioeli D, Slack-Davis JK, et al. Matrix rigidity regulates cancer cell growth and cellular phenotype. PLOS ONE. 2010 Sep 23;5(9):e12905. doi: 10.1371/journal.pone.0012905 20886123 PMC2944843

[120] Mittal V Epithelial Mesenchymal Transition in Tumor Metastasis. Annu Rev Pathol. 2018 Jan 24;13:395–412. 29414248 10.1146/annurev-pathol-020117-043854

[121] Deritei D, Anamika WJ, Zhou X, Silverman EK, Regan ER, Glass K. HHIP’s Dynamic Role in Epithelial Wound Healing Reveals a Potential Mechanism of COPD Susceptibility. bioRxiv; 2024 [cited 2024 Dec 18]. p. 2024.09.05.611545. https://www.biorxiv.org/content/10.1101/2024.09.05.611545v2 10.1073/pnas.2424377123 PMC13225619 42190016

[122] Chang J, Chaudhuri O. Beyond proteases: Basement membrane mechanics and cancer invasion. J Cell Biol. 2019 Aug 5;218(8):2456–69. 31315943 10.1083/jcb.201903066 PMC6683740

[123] F Kai, Drain AP, Weaver VM. The extracellular matrix modulates the metastatic journey. Dev Cell. 2019 May 6;49(3):332–46. 31063753 10.1016/j.devcel.2019.03.026 PMC6527347

[124] Nguyen LK, Cavadas MAS, Scholz CC, Fitzpatrick SF, Bruning U, Cummins EP, et al. A dynamic model of the hypoxia-inducible factor 1α (HIF-1α) network. Journal of Cell Science. 2013 Mar 15;126(6):1454–63. 23390316 10.1242/jcs.119974

[125] Qutub AA, Popel AS. A computational model of intracellular oxygen sensing by hypoxia-inducible factor HIF1α. J Cell Sci. 2006 Aug 15;119(Pt 16):3467–80. 16899821 10.1242/jcs.03087 PMC2129128

[126] Hashemzadeh S, Shahmorad S, Rafii-Tabar H, Omidi Y. Computational modeling to determine key regulators of hypoxia effects on the lactate production in the glycolysis pathway. Sci Rep. 2020 10(1):9163. 32514127 10.1038/s41598-020-66059-w PMC7280308

[127] Oliveira de RHM, Annex BH, Popel AS. Endothelial cells signaling and patterning under hypoxia: a mechanistic integrative computational model including the Notch-Dll4 pathway. Front Physiol. 2024 Feb 22 [cited 2024 Dec 18];15. Available from: https://www.frontiersin.org/journals/physiology/articles/10.3389/fphys.2024.1351753/full 10.3389/fphys.2024.1351753 PMC10917925 38455844

[128] Hanahan D. Hallmarks of Cancer: New Dimensions. Cancer Discov. 2022;12(1):31–46. doi: 10.1158/2159-8290.CD-21-1059 35022204

[129] Kierans SJ, Taylor CT. Regulation of glycolysis by the hypoxia-inducible factor (HIF): implications for cellular physiology. The Journal of Physiology. 2021;599(1):23–37. 33006160 10.1113/JP280572

[130] Gordan JD, Bertout JA, Hu C-J, Diehl JA, Simon MC. HIF-2α promotes hypoxic cell proliferation by enhancing c-Myc transcriptional activity. Cancer Cell. 2007;11(4):335–47. doi: 10.1016/j.ccr.2007.02.006 17418410 PMC3145406

[131] Zhu C, Yu J, Pan Q, Yang J, Hao G, Wang Y, et al. Hypoxia-inducible factor-2 alpha promotes the proliferation of human placenta-derived mesenchymal stem cells through the MAPK/ERK signaling pathway. Sci Rep. 2016 Oct 21;6(1):35489. 27765951 10.1038/srep35489 PMC5073233

[132] Ma X, Zhang H, Xue X, Shah YM. Hypoxia-inducible factor 2α (HIF-2α) promotes colon cancer growth by potentiating Yes-associated protein 1 (YAP1) activity. J Biol Chem. 2017 Oct 13; 292(41):17046–56. doi: 10.1074/jbc.M117.805655 28848049 PMC5641885

[133] Chen S-H, Xu L-Y, Wu Y-P, Ke Z-B, Huang P, Lin F, et al. Tumor volume: a new prognostic factor of oncological outcome of localized clear cell renal cell carcinoma. BMC Cancer. 2021 Jan 19;21(1):79. doi: 10.1186/s12885-021-07795-8 33468079 PMC7816334

[134] Chrabańska M, Szweda-Gandor N, Drozdzowska B. Two Single Nucleotide Polymorphisms in the Von Hippel-Lindau Tumor Suppressor Gene in Patients with Clear Cell Renal Cell Carcinoma. Int J Mol Sci. 2023;24(4):3778. doi: 10.3390/ijms24043778 36835190 PMC9959571

[135] Stone RC, Pastar I, Ojeh N, Chen V, Liu S, Garzon KI, et al. Epithelial-mesenchymal transition in tissue repair and fibrosis. Cell Tissue Res. 2016 Sep;365(3):495–506. doi: 10.1007/s00441-016-2464-0 27461257 PMC5011038

[136] Lin Q, Cong X, Yun Z. Differential hypoxic regulation of hypoxia-inducible factors 1alpha and 2alpha. Mol Cancer Res. 2011;9(6):757–65. doi: 10.1158/1541-7786.MCR-11-0053 21571835 PMC3117969

[137] Zhang P, Yao Q, Lu L, Li Y, Chen P-J, Duan C. Hypoxia-inducible factor 3 is an oxygen-dependent transcription activator and regulates a distinct transcriptional response to hypoxia. Cell Rep. 2014 Mar 27;6(6):1110–21. doi: 10.1016/j.celrep.2014.02.011 24613356

[138] Augstein A, Poitz DM, Braun-Dullaeus RC, Strasser RH, Schmeisser A. Cell-specific and hypoxia-dependent regulation of human HIF-3α: inhibition of the expression of HIF target genes in vascular cells. Cell Mol Life Sci. 2011 Aug 1;68(15):2627–42. 21069422 10.1007/s00018-010-0575-4 PMC11115058

[139] Sizek H, Deritei D, Fleig K, Harris M, Regan PL, Glass K, et al. Unlocking Mitochondrial Dysfunction-Associated Senescence (MiDAS) with NAD – a Boolean Model of Mitochondrial Dynamics and Cell Cycle Control. Tansl Onc. 2023 Dec 1 (submitted). 10.1016/j.tranon.2024.102084 PMC11380032 39163758

[140] Kang H,Kim H,Lee S,Youn H,Youn B.Role of Metabolic Reprogramming in Epithelial–Mesenchymal Transition (EMT).Int J Mol Sci.2019 Apr 25;20(8):2042. 10.3390/ijms20082042 PMC6514888 31027222

[141] Hong C-F, Chen W-Y, Wu C-W. Upregulation of Wnt signaling under hypoxia promotes lung cancer progression. Oncol Rep. 2017 Sep;38(3):1706–14. doi: 10.3892/or.2017.5807 28713928

[142] Shen X, Li M, Wang C, Liu Z, Wu K, Wang A, et al. Hypoxia is fine-tuned by Hif-1α and regulates mesendoderm differentiation through the Wnt/β-Catenin pathway. BMC Biol. 2022 Oct 5;20(1):219. 36199093 10.1186/s12915-022-01423-y PMC9536055

[143] Guo M, Niu Y, Xie M, Liu X, Li X. Notch signaling, hypoxia, and cancer. Front Oncol. 2023 Jan 31;13:1078768. 36798826 10.3389/fonc.2023.1078768 PMC9927648

[144] Gyamfi J, Lee YH, Eom M, Choi J. Interleukin-6/STAT3 signalling regulates adipocyte induced epithelial-mesenchymal transition in breast cancer cells. Sci Rep 2018 Jun 11;8:8859. 29891854 10.1038/s41598-018-27184-9 PMC5995871

[145] Zhang T, Yang J, Sun Y, Song J, Gao D, Huang S, et al. Interleukin-6 and Hypoxia Synergistically Promote EMT-Mediated Invasion in Epithelial Ovarian Cancer via the IL-6/STAT3/HIF-1α Feedback Loop. Anal Cell Pathol (Amst). 2023;2023:8334881. doi: 10.1155/2023/8334881 36814597 PMC9940980

[146] Li X, Kimura H, Hirota K, Kasuno K, Torii K, Okada T, et al. Synergistic effect of hypoxia and TNF-α on production of PAI-1 in human proximal renal tubular cells. Kidney International. 2005 Aug;68(2):569–83. 16014034 10.1111/j.1523-1755.2005.00435.x

[147] Basu S, Cheriyamundath S, Ben-Ze’ev A. Cell-cell adhesion: linking Wnt/β-catenin signaling with partial EMT and stemness traits in tumorigenesis. F1000Res. 2018 Sep 18;7:F1000 Faculty Rev-1488. doi: 10.12688/f1000research.15782.1 30271576 PMC6144947

[148] Katoh Y, Katoh M. Hedgehog signaling, epithelial-to-mesenchymal transition and miRNA. Int J Mol Med. 2008 Sep 22(3):271–5. 18698484

[149] Deshmukh AP, Vasaikar SV, Tomczak K, Tripathi S, den Hollander P, Arslan E, et al. Identification of EMT signaling cross-talk and gene regulatory networks by single-cell RNA sequencing. Proceedings of the National Academy of Sciences. 2021 May 11;118(19):e2102050118. 10.1073/pnas.2102050118 PMC8126782 33941680

[150] Bianchi JJ, Zhao X, Mays JC, Davoli T. Not all cancers are created equal: Tissue specificity in cancer genes and pathways. Curr Opin Cell Biol. 2020 Apr;63:135–43. doi: 10.1016/j.ceb.2020.01.005 32092639 PMC7247947

[151] Marusyk A, Janiszewska M, Polyak K. Intratumor Heterogeneity: The Rosetta Stone of Therapy Resistance. Cancer Cell. 2020;37(4):471–84. doi: 10.1016/j.ccell.2020.03.007 32289271 PMC7181408

[152] Reiter JG, Baretti M, Gerold JM, Makohon-Moore AP, Daud A, Iacobuzio-Donahue CA, et al. An analysis of genetic heterogeneity in untreated cancers. Nat Rev Cancer. 2019 Nov;19(11):639–50. doi: 10.1038/s41568-019-0185-x 31455892 PMC6816333

[153] Sun G, Li Z, Rong D, Zhang H, Shi X, Yang W, et al. Single-cell RNA sequencing in cancer: Applications, advances, and emerging challenges. Mol Ther Oncolytics. 2021 Jun 25;21:183–206. 34027052 10.1016/j.omto.2021.04.001 PMC8131398

[154] Shaul ME, Fridlender ZG. Tumour-associated neutrophils in patients with cancer. Nat Rev Clin Oncol. 2019 Oct;16(10):601–20. doi: 10.1038/s41571-019-0222-4 31160735

[155] Doak GR, Schwertfeger KL, Wood DK. Distant Relations: Macrophage Functions in the Metastatic Niche. Trends in Cancer. 2018 Jun 1;4(6):445–59. 29860988 10.1016/j.trecan.2018.03.011 PMC5990045

[156] Schlesinger M. Role of platelets and platelet receptors in cancer metastasis. J Hematol Oncol. 2018;11(1):125. doi: 10.1186/s13045-018-0669-2 30305116 PMC6180572

[157] Gaggioli C, Hooper S, Hidalgo-Carcedo C, Grosse R, Marshall JF, Harrington K, et al. Fibroblast-led collective invasion of carcinoma cells with differing roles for RhoGTPases in leading and following cells. Nat Cell Biol. 2007;9(12):1392–400. doi: 10.1038/ncb1658 18037882

[158] Guo Y, Cui W, Pei Y, Xu D. Platelets promote invasion and induce epithelial to mesenchymal transition in ovarian cancer cells by TGF-β signaling pathway. Gynecol Oncol. 2019 Jun;153(3):639–50. doi: 10.1016/j.ygyno.2019.02.026 30928020

[159] Nacarelli T, Lau L, Fukumoto T, Zundell J, Fatkhutdinov N, Wu S, et al. NAD+ metabolism governs the proinflammatory senescence-associated secretome. Nat Cell Biol. 2019 Mar;21(3):397–407. doi: 10.1038/s41556-019-0287-4 30778219 PMC6448588

[160] Smit MA, Peeper DS. Epithelial-mesenchymal transition and senescence: two cancer-related processes are crossing paths. Aging (Albany NY). 2010 Oct 17; 2(10):735–41. 10.18632/aging.100209 PMC2993803 20975209

[161] Liu Y, El-Naggar S, Darling DS, Higashi Y, Dean DC. ZEB1 links epithelial-mesenchymal transition and cellular senescence. Development. 2008 Feb;135(3):579–88. doi: 10.1242/dev.007047 18192284 PMC2507753

[162] Malik-Sheriff R, Glont M, Nguyen T Tiwari K, Roberts M, Xavier A, et al. BioModels-15 years of sharing computational models in life science. Nucleic Acids Res. 2020 Jan 8;48(D1):D407–15. 10.1093/nar/gkz1055 PMC7145643 31701150

[163] Chaouiya C, Naldi A, Thieffry D. Logical modelling of gene regulatory networks with GINsim. Methods Mol Biol. 2012;804:463–79. doi: 10.1007/978-1-61779-361-5_23 22144167

[164] Helikar T, Kowal B, McClenathan S, Bruckner M, Rowley T, Madrahimov A, et al. The Cell Collective: toward an open and collaborative approach to systems biology. BMC Syst Biol. 2012;6:96. doi: 10.1186/1752-0509-6-96 22871178 PMC3443426

[165] Albert I, Thakar J, Li S, Zhang R, Albert R. Boolean network simulations for life scientists. Source Code Biol Med. 2008 Nov 14;3:16. doi: 10.1186/1751-0473-3-16 19014577 PMC2603008

[166] yWorks GmbH. yEd. 2019. Available from: https://www.yworks.com/products/yed

[167] Mann M, Klemm K. Efficient exploration of discrete energy landscapes. Physical Review E. 2009 Oct; 83(1):011113. 10.1103/PhysRevE.83.011113 21405667

[168] Deritei D, Aird WC, Ercsey-Ravasz M, Regan ER. Principles of dynamical modularity in biological regulatory networks. Sci Rep. 2016 Mar 16;6:21957. 26979940 10.1038/srep21957 PMC4793241

[169] Sizek H, Hamel A, Deritei D, Campbell S, Ravasz Regan E. Boolean model of growth signaling, cell cycle and apoptosis predicts the molecular mechanism of aberrant cell cycle progression driven by hyperactive PI3K. PLoS Comput Biol. 2019;15(3):e1006402. doi: 10.1371/journal.pcbi.1006402 30875364 PMC6436762

[170] Guberman E, Sherief H, Regan ER. Boolean model of anchorage dependence and contact inhibition points to coordinated inhibition but semi-independent induction of proliferation and migration. Comput Struct Biotechnol J. 2020;18:2145–65. doi: 10.1016/j.csbj.2020.07.016 32913583 PMC7451872

