# Supplementary material for: A Boolean network model of hypoxia, mechanosensing and TGF-β signaling captures the role of phenotypic plasticity and mutations in tumor metastasis
Source: PLoS Comput Biol. 2025 Apr 16;21(4):e1012735. doi: 10.1371/journal.pcbi.1012735 (PMC12061430; doi:10.1371/journal.pcbi.1012735)
Supplement: S2 Fig — (PDF) [file pcbi.1012735.s002.pdf]

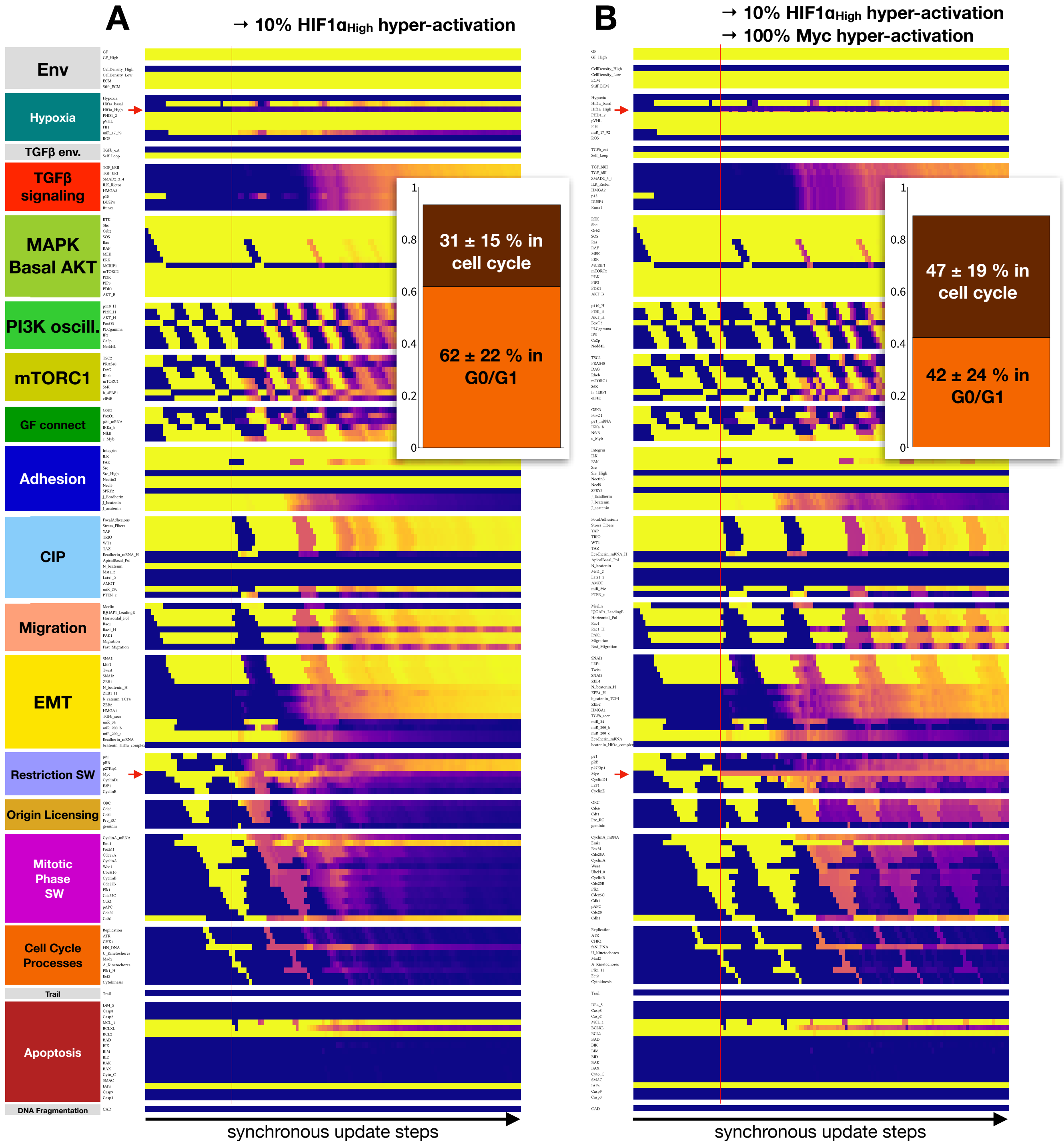

S2 Fig. Hif-1α hyper-activation blocks the cell cycle by repressing Myc. **A)** Dynamics of average expression/activity of all regulatory molecules across 1000 cycling cells (*left side of vertical red line*) exposed to 10% Hif-1α hyper-activation (*right side of vertical red line*). *Inset:* fraction of time cells spend in quiescence (*orange*) vs. in cell cycle (*dark red*) following Hif-1α hyper-activation. **B)** Dynamics of average expression/activity of all regulatory molecules across 1000 cycling cells (*left side of vertical red line*) simultaneously exposed to 10% Hif-1α and 100% Myc hyper-activation (*right side of vertical red line*). *Inset:* fraction of time cells spend in quiescence (*orange*) vs. in cell cycle (*dark red*) following Hif-1α/Myc hyper-activation. *X-axis:* synchronous update steps; *y-axis*: nodes organized by regulatory module; *yellow/purple/blue color scale*: ON/50%/OFF; *red arrows:* Hif1a_High / Myc node. *Initial condition*: epithelial cells in GF:1, CellDensity_Low:1, GF_High:1, Stiff_ECM:1, Trail:0, Self_Loop:1, TGFb_ext:0, Hypoxia:0; *autocrine TGF-β*: 5% TGFb_secr knockdown.
