# Supplementary material for: A Boolean network model of hypoxia, mechanosensing and TGF-β signaling captures the role of phenotypic plasticity and mutations in tumor metastasis
Source: PLoS Comput Biol. 2025 Apr 16;21(4):e1012735. doi: 10.1371/journal.pcbi.1012735 (PMC12061430; doi:10.1371/journal.pcbi.1012735)
Supplement: S3 Fig — (PDF) [file pcbi.1012735.s003.pdf]

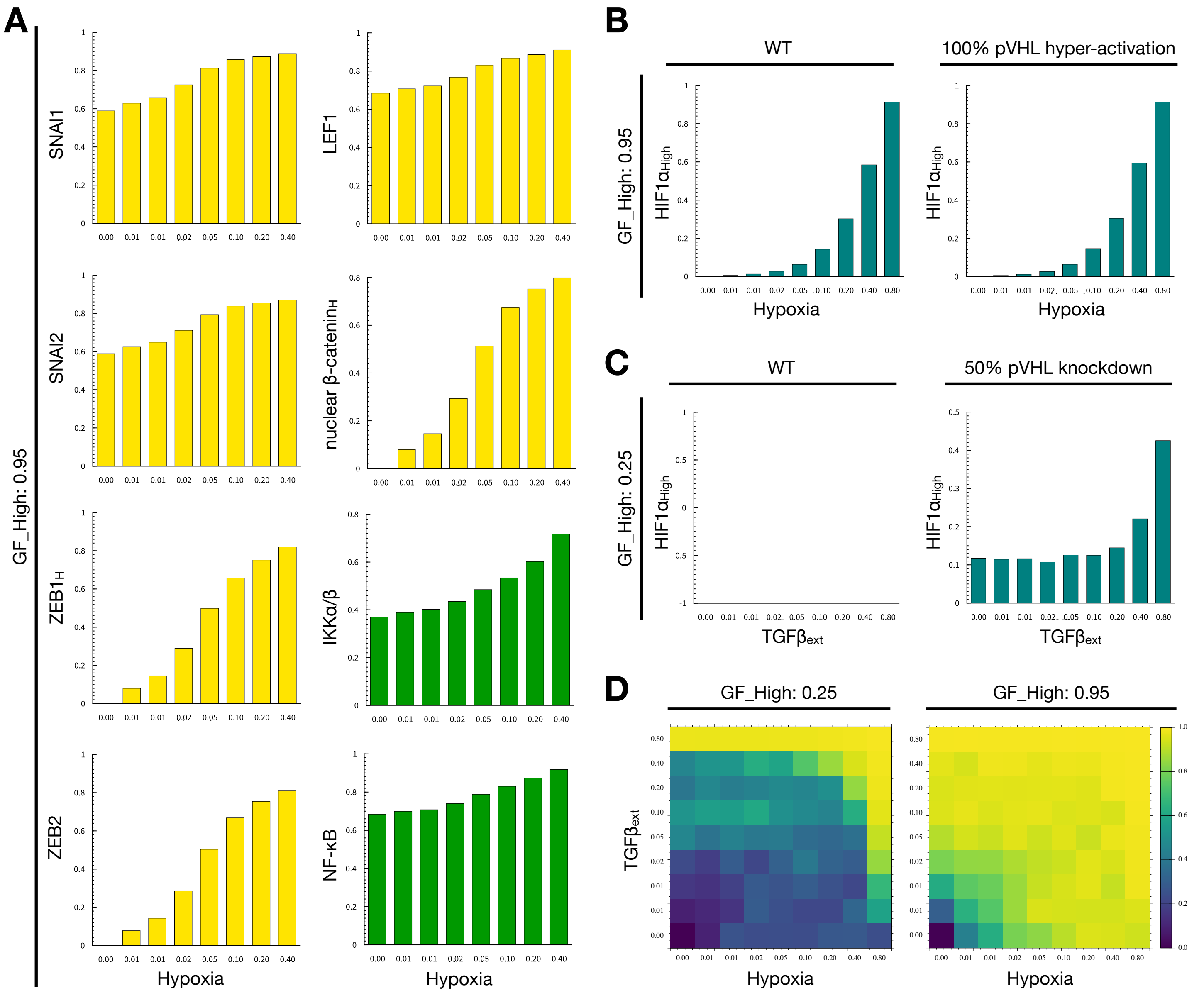

S3 Fig. Hypoxia induces EMT independently of TFG-β. A) Average activation of mesenchymal factors SNAI1, SNAI2, ZEB1 (high expression node *ZEB1_H*), ZEB2, LEF1, nuclear nuclear β-catenin (high expression node *N_bcatenin_H*), IKKα/β and NF-κB in an ensemble of initially epithelial cells in response to increasing hypoxia exposure (log 2 hypoxia scale; *environment:* GF_High:0.95, TGFb_ext:0). **B)** Average Hif-$1\alpha_{high}$ activation in an ensemble of initially epithelial cells in response to increasing hypoxia exposure (log 2 hypoxia scale; *environment:* GF_High:0.95, TGFb_ext:0). *Left:* wild-type; *right:* 100% pVHL hyper-activation. **C)** Average Hif-$1\alpha_{high}$ activation in an ensemble of initially epithelial cells in response to increasing hypoxia exposure (log 2 hypoxia scale; *environment:* GF_High:0.25, Hypoxia:0). *Left:* wild-type; *right:* 50% pVHL knockdown. **D)** Fraction of time initially epithelial cells spend a mesenchymal state, as a function of hypoxia (*x axis*, log2 scale) and external TFG-β (*y axis*, log2 scale). *Left:* 25% GF_High; *right:* 95% GF_High. *Length of time-window for continuous runs:* 100 steps (~5 wild-type cell cycle lengths); *total sampled live cell time:* 100,000 steps; *update*: synchronous; *condition for all sampling runs*: CellDensity_Low:1, Stiff_ECM:1, Trail:0, Self_Loop:1; *autocrine TGF-β*: 5% TGFb_secr knockdown.
