# Supplementary material for: A Boolean network model of hypoxia, mechanosensing and TGF-β signaling captures the role of phenotypic plasticity and mutations in tumor metastasis
Source: PLoS Comput Biol. 2025 Apr 16;21(4):e1012735. doi: 10.1371/journal.pcbi.1012735 (PMC12061430; doi:10.1371/journal.pcbi.1012735)
Supplement: S4 Fig — (PDF) [file pcbi.1012735.s004.pdf]

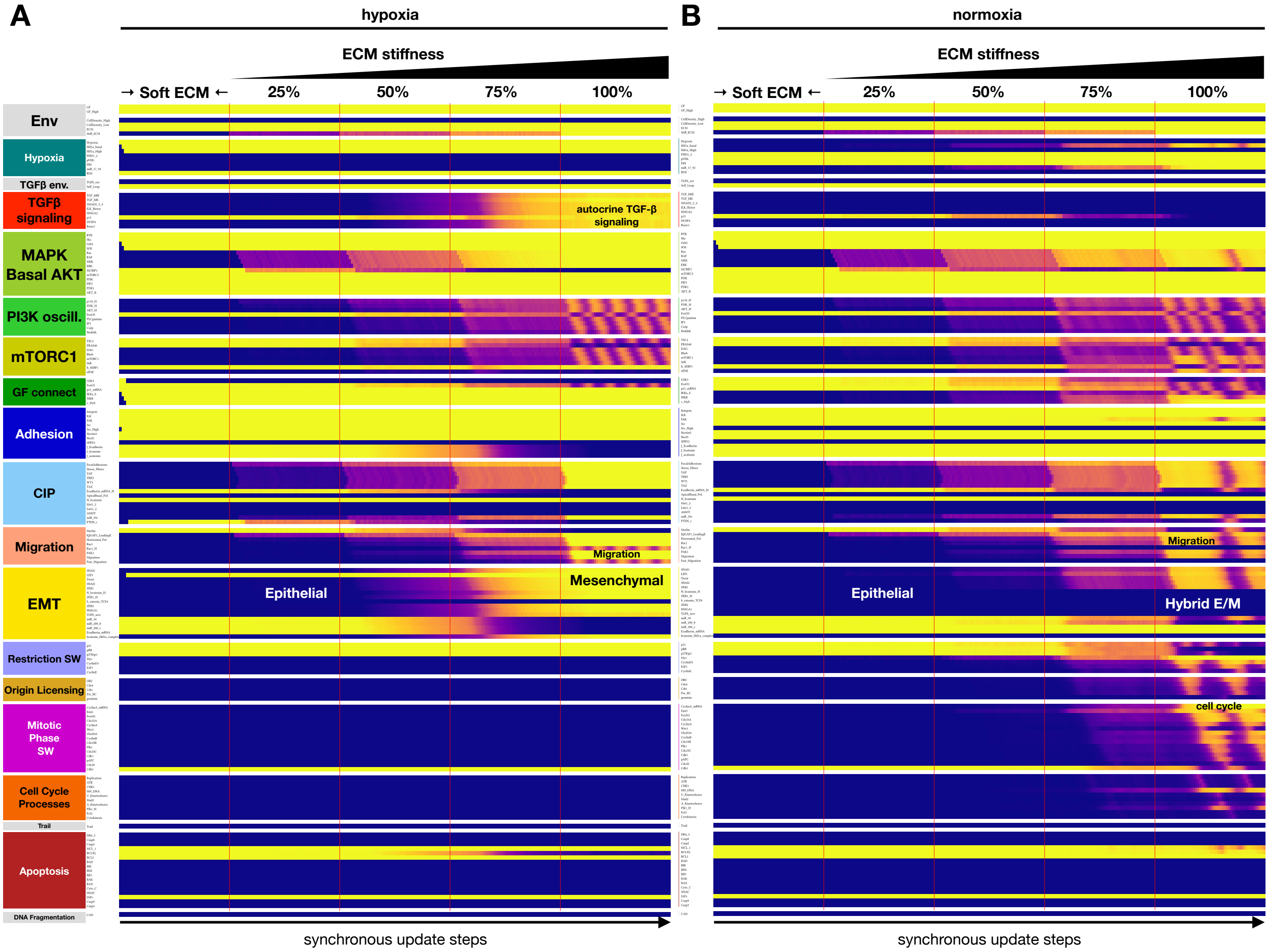

S4 Fig. A stiff ECM aids hypoxia-induced EMT, compared to hybrid E/M under normoxia. A) Dynamics of regulatory molecule expression in a quiescent cell on a soft ECM under hypoxia, in response to increasing ECM stiffness (50 update-steps of 0,25,50,75 ans 100% Stiff_ECM = ON at saturating mitogen exposure and moderate cell density). Full time-course version of Fig. 4A. **B)** Dynamics of regulatory molecule expression in a quiescent cell on a soft ECM under normoxia, in response to increasing ECM stiffness (50 update-steps of 0,25,50,75 ans 100% Stiff_ECM = ON at saturating mitogen exposure and moderate cell density). *X-axis:* update steps; *y-axis*: nodes organized by regulatory module; *yellow/purple/ blue scale*: 100% ON/ 50% on / 100% OFF; *black/white labels:* relevant phenotypes; *update*: synchronous; *autocrine TGF-β*: 5% TGFb_secr knockdown.
