# Supplementary material for: A Boolean network model of hypoxia, mechanosensing and TGF-β signaling captures the role of phenotypic plasticity and mutations in tumor metastasis
Source: PLoS Comput Biol. 2025 Apr 16;21(4):e1012735. doi: 10.1371/journal.pcbi.1012735 (PMC12061430; doi:10.1371/journal.pcbi.1012735)
Supplement: S5 Fig — (PDF) [file pcbi.1012735.s005.pdf]

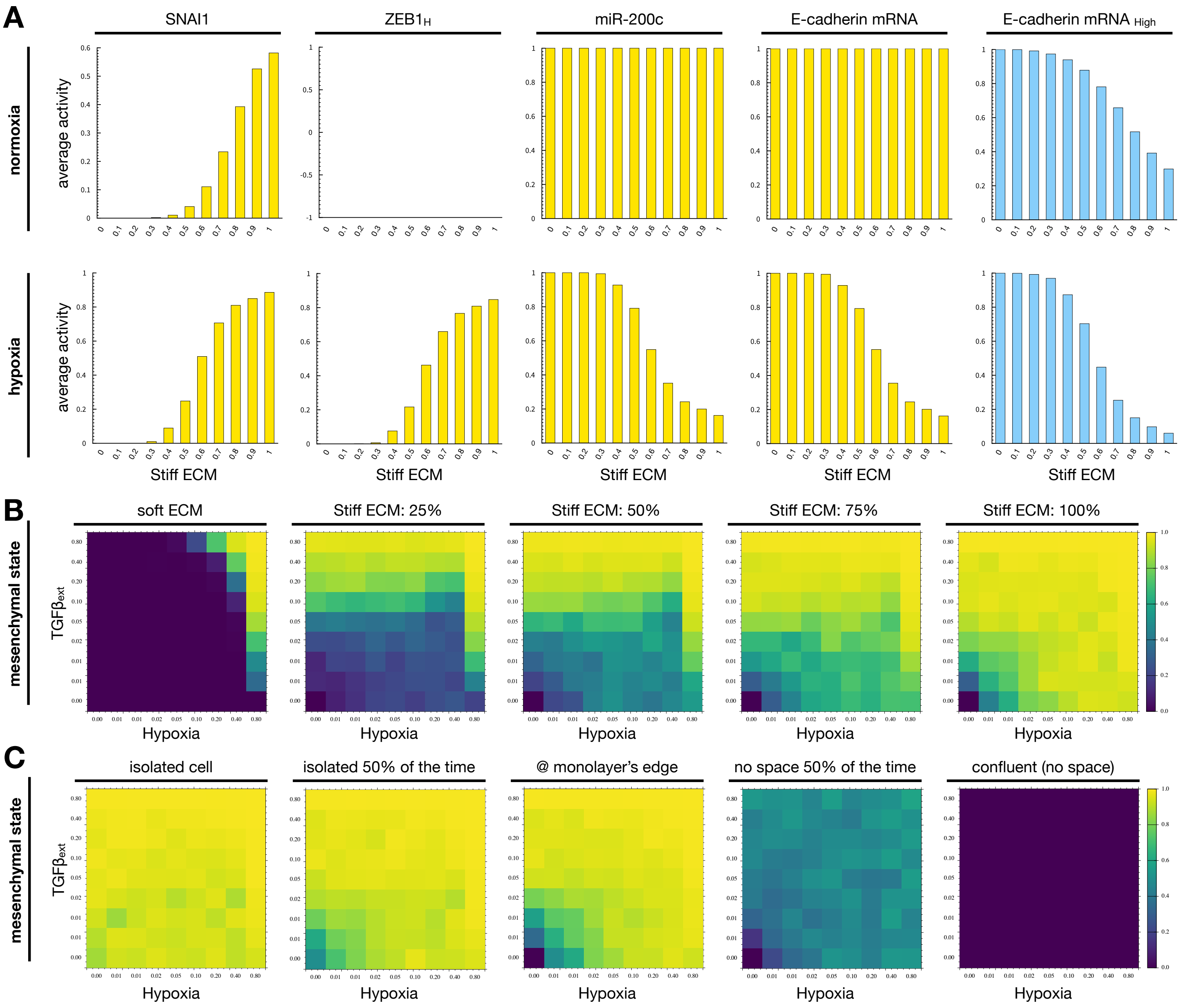

S5 Fig. ECM stiffness boosts both hypoxia- and TGF-β-induced EMT; high levels of both are required to drive EMT on very soft ECMs. A) Average expression of SNAI1, high ZEB1, miR-200c and E-cadherin mRNA (*yellow*: baseline expression; *light blue:* high expression) in normoxic (*top*) vs. hypoxic (*bottom*) cells at moderate density and saturating mitogen exposure, plated on ECM of increasing stiffness (CellDensity_Low:1). **B)** Fraction of time initially epithelial cells spend a mesenchymal state, as a function of hypoxia (*x axis*) and external TFG-β (*y axis*) plated on ECMs of increasing stiffness (soft ECM, 25%, 50%, 75% and 100% Stiff_ECM; CellDensity_Low:1). **C)** Fraction of time initially epithelial cells spend a mesenchymal state, as a function of hypoxia (*x axis*) and external TFG-β (*y axis*) plated on ECMs of increasing cell density (isolated, 50% CellDensity_Low, 100% CellDensity_Low, 50% CellDensity_High, 100% CellDensity_High; Stiff_ECM:1). *Length of time-window for continuous runs:* 100 steps (~5 wild-type cell cycle lengths); *total sampled live cell time:* 100,000 steps; *update*: synchronous; *initial condition for all sampling runs*: epithelial cells in GF:1, CellDensity_Low:1, Stiff_ECM:1, Trail:0, Self_Loop:1, TGFb_ext:0, Hypoxia:0; *environment of sampling runs:* GF_High:0.95, *autocrine TGF-β loop:* 95% (5% TGFb_secr knockdown).
