# Supplementary material for: A Boolean network model of hypoxia, mechanosensing and TGF-β signaling captures the role of phenotypic plasticity and mutations in tumor metastasis
Source: PLoS Comput Biol. 2025 Apr 16;21(4):e1012735. doi: 10.1371/journal.pcbi.1012735 (PMC12061430; doi:10.1371/journal.pcbi.1012735)
Supplement: S6 Fig — (PDF) [file pcbi.1012735.s006.pdf]

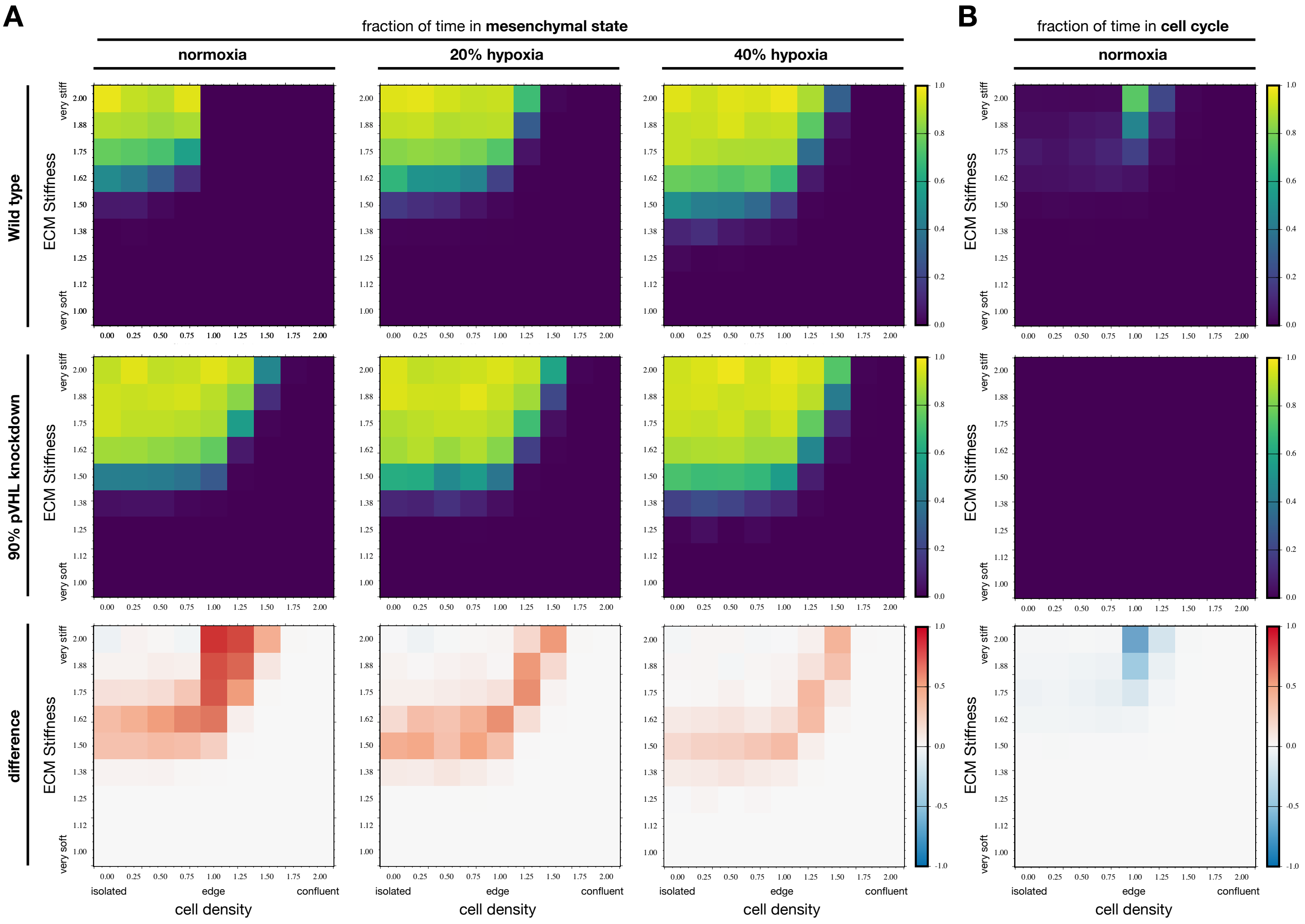

S6 Fig. VHL deficiency boosts bio mechanically induced EMT in normoxia as well as hypoxia at moderate ECM stiffness and medium-high density, but abolishes cell cycle entry. A) Fraction of time initially epithelial cells spend in a mesenchymal state as a function of cell density (*x axis*) and ECM stiffness (*y axis*) in normoxia (*left column*), 20% hypoxia (*middle column*) and 40% hypoxia (*right column*). **B)** Fraction of time cells spend in the cell cycle under normoxic conditions, as a function of cell density (*x axis*) and ECM stiffness (*y axis*). (A-B) *Top row*: wild-type cells; *middle row*: VHL deficient cells (90% pVHL knockdown); *bottom row:* difference (pVHL - wild-type). *Length of time-window for continuous runs:* 100 steps (~5 wild-type cell cycle lengths); *total sampled live cell time:* 100,000 steps; *update*: synchronous; *condition for all sampling runs*: GF_High:0.95, TGF_ext:0, Trail:0, Self_Loop:1; *autocrine TGF-β*: 5% TGFb_secr knockdown.
