# Supplementary material for: A Boolean network model of hypoxia, mechanosensing and TGF-β signaling captures the role of phenotypic plasticity and mutations in tumor metastasis
Source: PLoS Comput Biol. 2025 Apr 16;21(4):e1012735. doi: 10.1371/journal.pcbi.1012735 (PMC12061430; doi:10.1371/journal.pcbi.1012735)
Supplement: S7 Fig — (PDF) [file pcbi.1012735.s007.pdf]

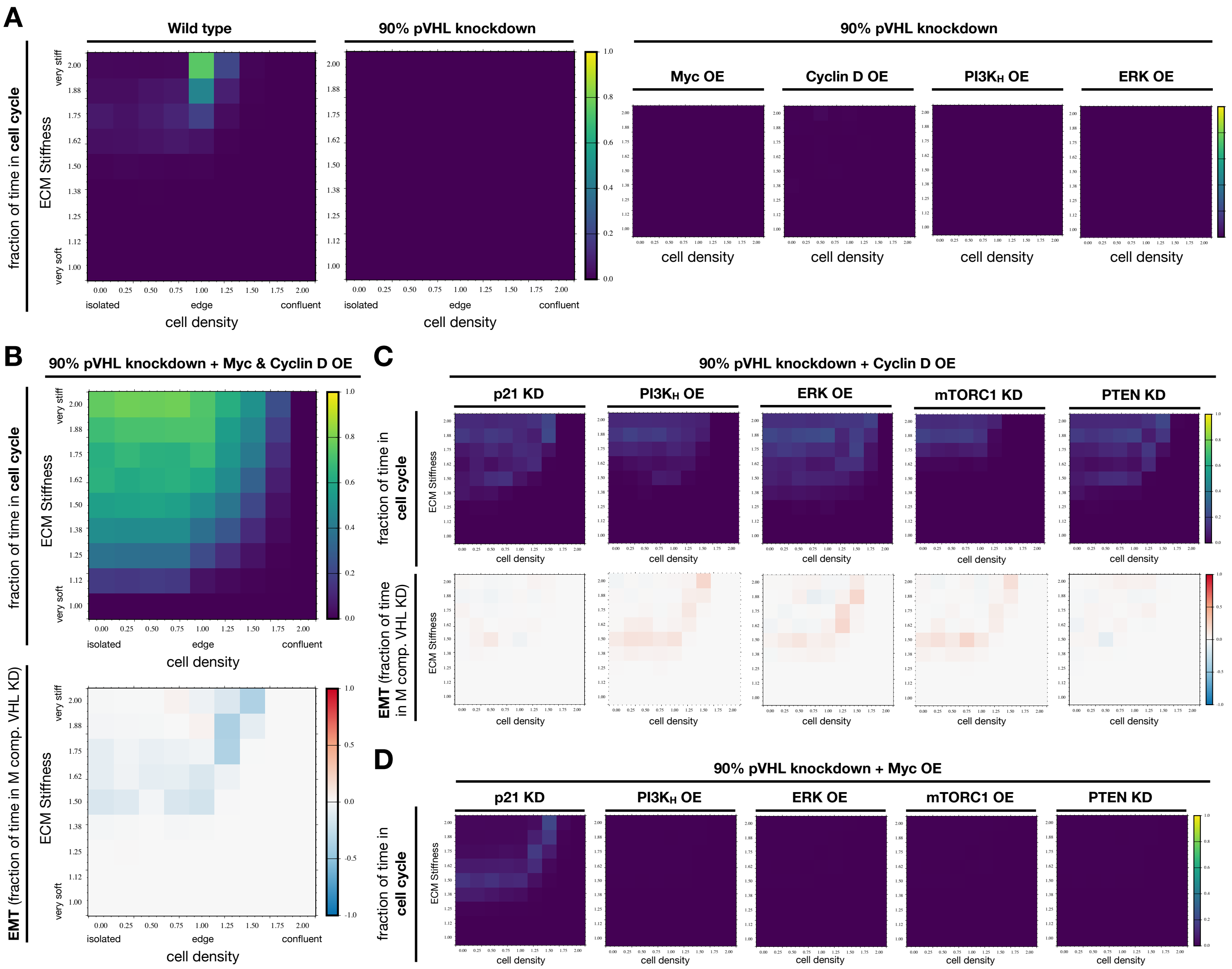

S7 Fig. VHL deficiency-induced cell cycle arrest is broken by cooperative hyper-activation of Myc and Cyclin D. A-B) Fraction of time normoxic cells spend in cell cycle as a function of density (*x axis*), ECM stiffness (*y axis*), and genetic background (wild-type, 90% pVHL knockdown, and 90% pVHL knockdown plus 100% ON lock of Myc, CyclinD1, PI3K_H or ERK). **B)** *Top:* Fraction of time normoxic cells with a 90% VHL deficiency and dual Myc/CyclinD1 hyper-activation (100% ON) spend in cell cycle as a function of density (*x axis*), ECM stiffness (*y axis*). *Bottom:* Difference in the fraction of time normoxic cells with a 90% VHL deficiency and dual Myc/CyclinD1 hyper-activation (100% ON) spend in a mesenchymal state, compared to cells with VHL deficiency deficiency only, as a function of density (*x axis*), ECM stiffness (*y axis*). *Red/blue:* more/less time in M state in triple-mutant cells. **C)** *Top:* Fraction of time normoxic cells with a 90% VHL deficiency and 100% CyclinD1 hyper-activation spend in cell cycle as a function of density (*x axis*), ECM stiffness (*y axis*), and additional genetic alterations (100% p21_mRNA:0, PI3K_H:1, ERK:1, mTORC1:1 or PTEN_c:0). *Bottom:* Difference in the fraction of time normoxic cells with a 90% VHL deficiency and 100% CyclinD1 hyper-activation spend in a mesenchymal state, compared to VHL deficiency deficiency only, as a function of density (*x axis*), ECM stiffness (*y axis*), and additional genetic alterations. **D)** Fraction of time normoxic cells with a 90% VHL deficiency and 100% Myc hyper-activation spend in cell cycle as a function of density (*x axis*), ECM stiffness (*y axis*), and additional genetic alterations. (A-D) *Length of time-window for continuous runs:* 100 steps (~5 wild-type cell cycle lengths); *total sampled live cell time:* 100,000 steps; *update*: synchronous; *condition for all sampling runs*: GF_High:0.95, TGF_ext:0, Hypoxia:0, Trail:0, Self_Loop:1; *autocrine TGF-β*: 5% TGFb_secr knockdown.
