# Supplementary material for: A Boolean network model of hypoxia, mechanosensing and TGF-β signaling captures the role of phenotypic plasticity and mutations in tumor metastasis
Source: PLoS Comput Biol. 2025 Apr 16;21(4):e1012735. doi: 10.1371/journal.pcbi.1012735 (PMC12061430; doi:10.1371/journal.pcbi.1012735)
Supplement: S8 Fig — (PDF) [file pcbi.1012735.s008.pdf]

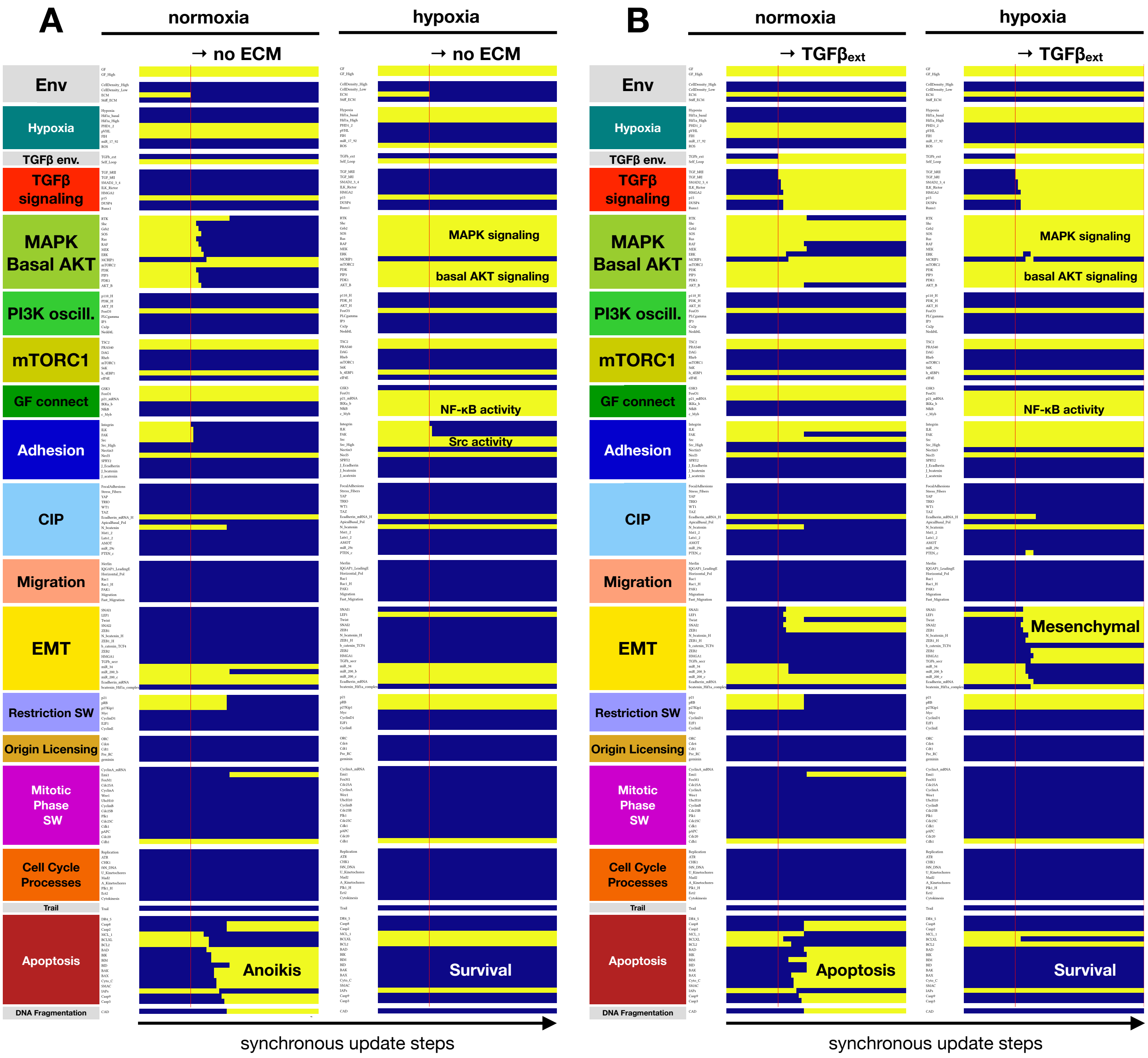

S8 Fig. Hypoxia confers anoikis resistance and protection from TGF-β induced epithelial cell apoptosis on a soft ECM (full version of Fig. 5). A) Dynamics of regulatory molecule expression in a quiescent cell detached from a soft ECM in normoxia (left) vs. hypoxia (right) (20 update-steps on soft ECM, 50 update-steps detached, 100% high GF). **B)** Dynamics of regulatory molecule expression in a quiescent cell on a soft ECM, exposed to exogenous TGF-β in normoxia (left) vs. hypoxia (right) (20 update-steps no TGF-β, 50 update-steps 100% TGF-β). X-axis: update steps; y-axis: nodes organized by regulatory module; yellow/blue: ON/OFF; black/white labels: relevant phenotypes; update: synchronous.
