# Supplementary material for: A Boolean network model of hypoxia, mechanosensing and TGF-β signaling captures the role of phenotypic plasticity and mutations in tumor metastasis
Source: PLoS Comput Biol. 2025 Apr 16;21(4):e1012735. doi: 10.1371/journal.pcbi.1012735 (PMC12061430; doi:10.1371/journal.pcbi.1012735)
Supplement: S9 Fig — (PDF) [file pcbi.1012735.s009.pdf]

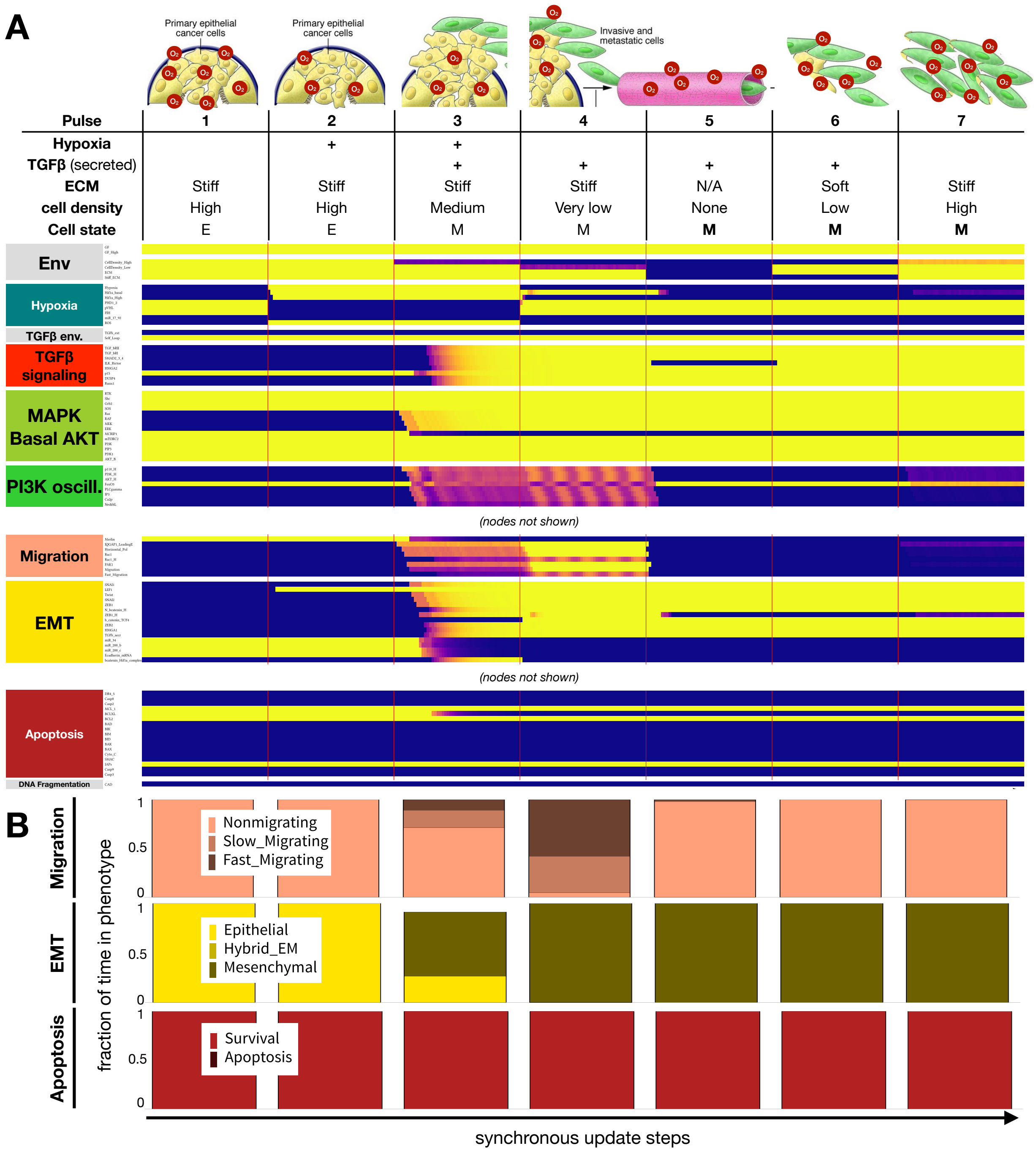

S9 Fig. Saturating autocrine TGF-β signaling protects all mesenchymal cells from anoikis, but also blocks MET on a stiff ECM. A) Dynamics of relevant regulatory molecule expression in an ensemble of 1000 quiescent epithelial cell exposed to a sequence of microenvironments along the metastatic cascade (100 update-steps/pulse). *Pulse 1:* normoxia, stiff ECM, high density; *Pulse 2:* hypoxia, stiff ECM, high density; *Pulse 3:* hypoxia, stiff ECM, medium density; *Pulse 4* (invasion): normoxia, stiff ECM, very low density; *Pulse 5* (intravasation): normoxia, no ECM, no neighbors; *Pulse 6* (extravasation): normoxia, soft ECM, low density; *Pulse 7* (MET): normoxia, stiff ECM, high density. *X-axis:* update steps; *y-axis:* nodes organized by regulatory module; *yellow/blue:* ON/OFF; *black/white labels:* relevant phenotypes; *update:* synchronous; *autocrine TGF-β:* saturating. **B)** Average fraction of time an ensemble of 1000 cells spend in *top*: non-migrating (*light pink*), slow-migrating (*dark pink*) and fast-migrating (*brow-pink*) states; *middle*: epithelial (*yellow*), hybrid E/M (*dark yellow*) and mesenchymal (*mustard*) states; *bottom*: survival (*red*) vs. in apoptotic (*dark red*) states for each pulse along the metastatic cascade in panel A. *Image credits:* metastatic cascade adapted from https://commons.wikimedia.org/wiki/File:Contribution_of_EMT_to_cancer_progression.jpg.
