# Supplementary material for: A Boolean network model of hypoxia, mechanosensing and TGF-β signaling captures the role of phenotypic plasticity and mutations in tumor metastasis
Source: PLoS Comput Biol. 2025 Apr 16;21(4):e1012735. doi: 10.1371/journal.pcbi.1012735 (PMC12061430; doi:10.1371/journal.pcbi.1012735)
Supplement: S10 Fig — (PDF) [file pcbi.1012735.s010.pdf]

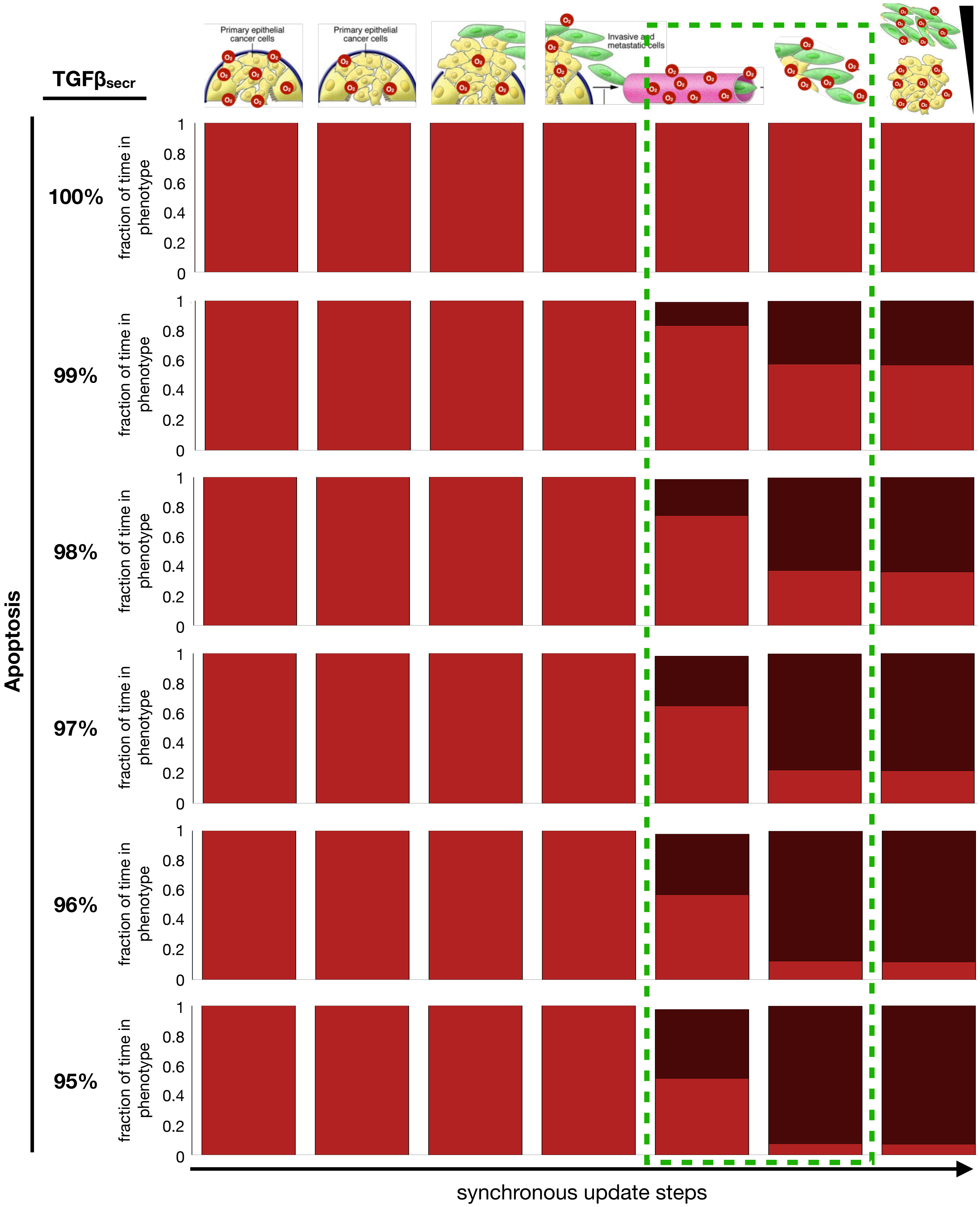

S10 Fig. Slight breaks in the autocrine TGF-β signaling loop are potent inducers of anoikis and apoptosis on soft ECM. Average fraction of time an ensemble of 1000 cells spend in survival (*red*) vs. in apoptotic (*dark red*) states for each pulse along the metastatic cascade in Fig. 6 / SM Fig. 9, as a function of a weakening autocrine loop (1 to 5% TGFb_secr knockdown). *Image credits:* metastatic cascade adapted from *https://commons.wikimedia.org/wiki/File:Contribution_of_EMT_to_cancer_progression.jpg*.
