# Supplementary material for: A Boolean network model of hypoxia, mechanosensing and TGF-β signaling captures the role of phenotypic plasticity and mutations in tumor metastasis
Source: PLoS Comput Biol. 2025 Apr 16;21(4):e1012735. doi: 10.1371/journal.pcbi.1012735 (PMC12061430; doi:10.1371/journal.pcbi.1012735)
Supplement: S11 Fig — (PDF) [file pcbi.1012735.s011.pdf]

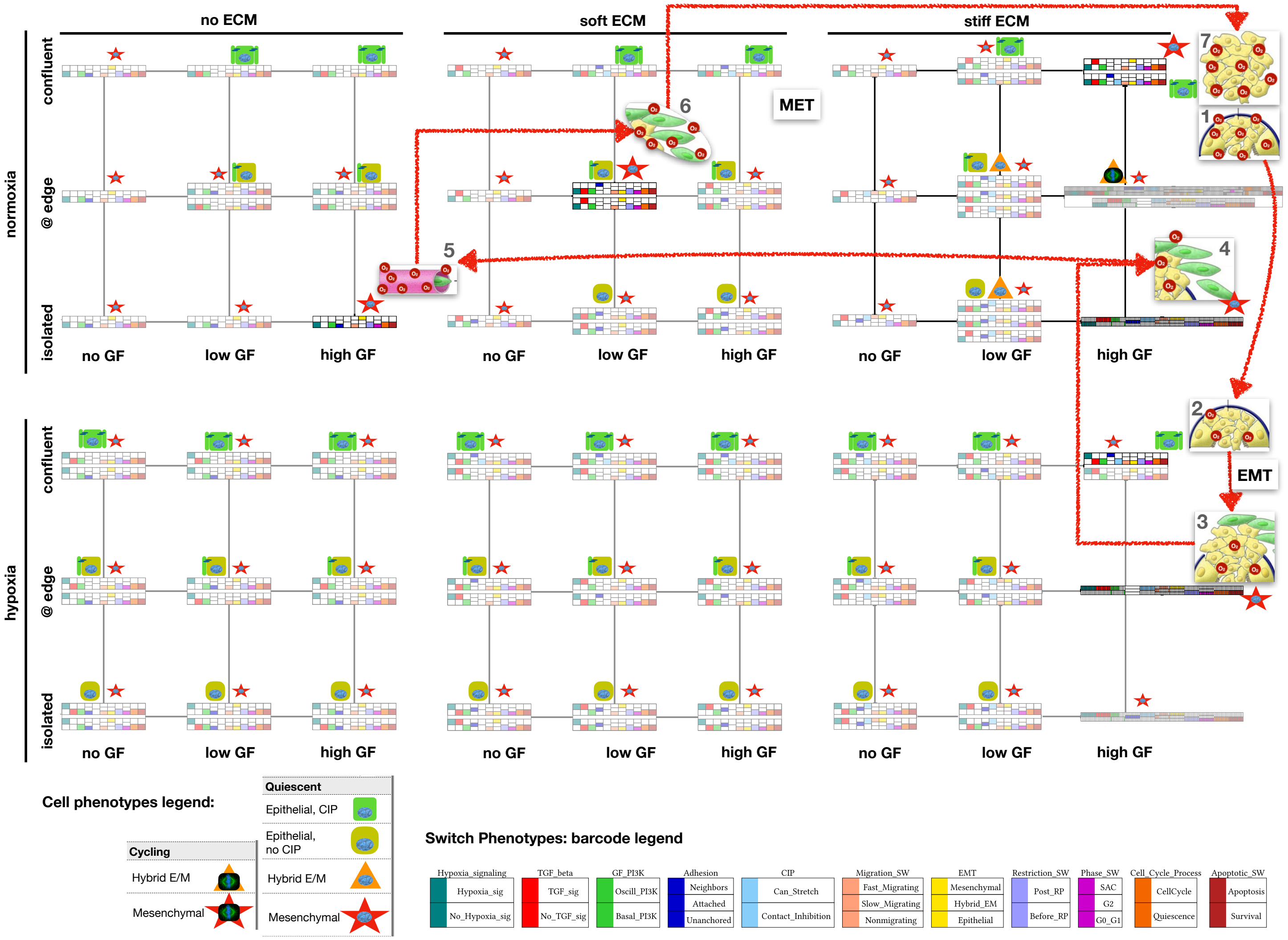

S11 Fig. A changing mix of model cell phenotypes as a function of mitogen, cell density, ECM stiffness and hypoxia allows us to track TME-driven EMT and MET during the metastatic cascade. Summary of model cell states detected in every combination of low/ high growth-factor (*x axis*), isolated cells / moderate density / high cell density (*y axis*), no ECM / very soft ECM / very stiff ECM (*left to right*) in normoxia vs. hypoxia (*top vs. bottom*). Apoptotic and tetraploid quiescent cell states were omitted for clarity. Visual summaries of epithelial (*green/mustard sybmols*) / hybrid EM (*orange triangle*) / mesenchymal (*red star*) cell phenotypes (see cell phenotype legend, *bottom left*). Barcodes provide detailed phenotypic descriptions for each cell state (attractor); derived by comparing the expression of nodes in relevant modules to predetermined molecular signatures (e.g., apoptosis vs. survival), encoded in the *dmms* model file (barcode legend, *bottom right*). Oscillatory phenotypes have expanded barcodes that mark the transitions their regulatory switches undergo during the cycle (*high GF, stiff ECM*). *State transition arrows:* environment and cell phenotype (attractor) changes along the steps of the metastatic cascade shown in Fig. 6. *Saturated barcodes:* cell states along the cascade; *transparent barcodes:* all other diploid live cell states. *Image credits:* metastatic cascade adapted from *https://commons.wikimedia.org/wiki/File:Contribution_of_EMT_to_cancer_progression.jpg*; mitotic spindle image in cycling cell icons: *https://commons.wikimedia.org/wiki/File:Kinetochore.jpg*.
