# Supplementary material for: A Boolean network model of hypoxia, mechanosensing and TGF-β signaling captures the role of phenotypic plasticity and mutations in tumor metastasis
Source: PLoS Comput Biol. 2025 Apr 16;21(4):e1012735. doi: 10.1371/journal.pcbi.1012735 (PMC12061430; doi:10.1371/journal.pcbi.1012735)
Supplement: S1 Text — (PDF) [file pcbi.1012735.s013.pdf]

S1 Description & experimental support for the modules of Hypoxia_EMT_Model_Fine.

Table S1a: GrowthFactor_Env module

Target Node	Node Gate		
	Node Type	Node Description	
	Link Type	Input Node	Link Description
GF	**GF** = **GF** or **GF_High**		
	Env	The *GF* node represents an extracellular environment with low levels of growth factors capable of sustaining survival signaling. Thus, the *GF* input node is self-sustaining in the absence of *in silico* perturbation.	
	← Env	GF	The *GF* input node is self-sustaining in the absence of *in silico* perturbation.
	← Env	GF_High	The *GF* node represents an extracellular environment with low levels of growth factors capable of sustaining survival signaling. Thus, this node is ON in high growth factor as well.
GF_High	**GF_High** = **GF_High**		
	Env	The GF_{High} node in our model represents an extracellular environment with saturating levels of growth factors; this input node is self-sustaining in the absence of *in silico* perturbation.	
	← Env	GF_High	The GF_{High} input node is self-sustaining in the absence of *in silico* perturbation.

Table S1b: PhysEnv module

Target Node	Node Gate		
	Node Type	Node Description	
	Link Type	Input Node	Link Description
CellDensity _High	**CellDensity_High** = **CellDensity_High**		
	Env	The *CellDensity_High* node in our model represents an extracellular environment with high enough cell density to block cell spreading. This input node is self-sustaining in the absence of *in silico* perturbation.	
	← Env	CellDensity _High	*CellDensity_High* is self-sustaining in the absence of *in silico* perturbation.
CellDensity _Low	**CellDensity_Low** = **CellDensity_Low** or **CellDensity_High**		
	Env	The *CellDensity_Low* node represents an environment with cell density comparable to the edge of a monolayer, where cells can maintain strong adhesions with each other but are also able to spread and polarize horizontally.	

Table S1b: PhysEnv module

	← Env	CellDensity_High	*CellDensity_Low* is automatically ON at very high cell density.
	← Env	CellDensity_Low	*CellDensity_Low* is self-sustaining in the absence of *in silico* perturbation.
ECM	**ECM** = **ECM** or **Stiff_ECM**		
	Env	The *ECM* input node represents access to a very soft extracellular matrix that does not support cell spreading or stress fiber formation ($<$ 0.5 kPa), but does support anchorage-dependent survival signaling [1]. This input node is self-sustaining in the absence of a stiff *ECM* (or *in silico* perturbation), and overridden to an ON state otherwise by *Stiff_ECM*.	
	← Env	Stiff_ECM	*ECM* is automatically ON when cells have access to stiff *ECM*.
	← Env	ECM	*ECM* is automatically ON when cells have access to stiff *ECM*.
Stiff_ECM	**Stiff_ECM** = **Stiff_ECM**		
	Env	The *Sfiff_ECM* input node represents access to a very stiff extracellular matrix that promotes / supports stress fiber formation sufficiently to place no limitation on a cell's capacity to proliferate ($>$ 100 kPa) [1]. This input node is self-sustaining in the absence of *in silico* perturbation.	
	← Env	Stiff_ECM	*Sfiff_ECM* is self-sustaining in the absence of *in silico* perturbation.

Table S1c: Hypoxia_signaling module

Target Node	Node Gate<br>Node Type<br>Link Type	 Node Description<br>Input Node	 <br>Link Description
Hypoxia	**Hypoxia** = **Hypoxia**		
	Env	The *Hypoxia* input node represents an environment with less than 1% cellular oxygen level, or $CoCl_2$ treatment mimicking hypoxia. [2].	
	← Env	Hypoxia	*Hypoxia* is self-sustaining in the absence of *in silico* perturbation.
Hif1a_basal	**Hif1a_basal** = not((**PHD1_2** and **pVHL**) or **miR_17_92** or (**FIH** and not **Hypoxia**)) or (**mTORC1** and **eIF4E** and **S6K**) or (**ERK** and not(**GSK3** and **FoxO3**))		
	Prot	This node represents basal expression of *Hif-1α*, observed under normoxic conditions in dividing cells, part of the Warburg effect (aerobic glycolysis) [3]. It plays a similar role to the *HIF1* node in our previously published model of mitochondrial dysfunction-induced senescence [4]. Links in turquoise font are copied from this model.	

Table S1c: Hypoxia_signaling module

	← P	ERK	*ERK*-mediated phosphorylation of *HIF-1α* regulates its physical interaction with *NPM1*, a histone chaperone and chromatin remodeler. This interaction increases *HIF-1α* targeting to hypoxia target genes and their transcriptional activation [5].
	⊢ IBind	FoxO3	*FoxO3* interferes with and reduces *p300*-dependent *HIF-1α* transcription [6].
	← Ind	mTORC1	*mTORC1* activation downtream of *AKT* increases *HIF-1α* protein expression [7] protein accumulation through enhanced *STAT3* dependent transcription [8].
	← TL	S6K	*mTORC1*-induced *S6K* activation increases *HIF-1α* protein translation [8].
	← TL	eIF4E	*mTORC1*-induced *eIF4E* activation increases *HIF-1α* protein translation [8].
	⊢ P	GSK3	*GSK3β* phosphorylates and destabilizes *HIF-1α*, independently of thye hypoxia sensor *VHL* [9].
	← ComplProc	Hypoxia	*Hif-1α* binding to *FIH* is oxygen dependent, preventing *Hif-1α* transactivation [10].
	⊢ Ubiq	pVHL	Ubiquitination of *Hif-1α* on its oxygen dependent degradation domain results in degradation by *VHL*, the substrate recognition domain of an E3 ubiquitin ligase [11]
	⊢ -OH	PHD1_2	PDH proteins hydroxylate the oxygen-dependent domain of *Hif-1α* in an oxygen dependent mechanism, allowing for recognition by *VHL*, and subsequent degredation [12].
	⊢ RNAi	miR_17_92	*Hif-1α* protein levels were significantly downregulated in cell lines overexpressing the *miR-17-92* microRNA cluster [13].
	⊢ IBind	FIH	*Hif-1α* is shown to directly bind *FIH* in an oxygen dependent manner, preventing *Hif-1α* transactivation. This provides a unifying mechanism to repress *Hif-1α* function under normoxic, but not hypoxic conditions [10]
Hif1a_High	**Hif1a_High** = **Hif1a_basal** and (not(**PHD1_2** and **pVHL**) or (**Hypoxia** and not(**FIH** and **miR_200_c** and **miR_17_92**)))		
	Prot		This model uses *Hif1a_High* to indicate a strong but non-lethal level of *Hif-1α* accumulation, induced by hypoxia or complete knockdown of *Hif-1α* repressors.
	⊢ RNAi	miR_200_c	Expression of *miR-200c* decreases the ability of *Hif-1α* to bind to a know HRE, and reduces the mRNA expression of *Hif-1α* targets[14].
	← ComplProc	Hypoxia	*Hif-1α* binding to *FIH* is oxygen dependent, preventing *Hif-1α* transactivation [10].
	← Per	Hif1a_basal	In our model, high *Hif-1α* activity is contingent on the ON-state of the basal *Hif-1α* node.

Table S1c: Hypoxia_signaling module

	⊣ Ubiq	pVHL	Ubiquitination of *Hif-1α* on its oxygen dependent degredation domain by *VHL* results in degradation [11].
	⊣ -OH	PHD1_2	PDH proteins hydroxylate the oxygen-dependent domain of *Hif-1α* in an oxygen dependent mechanism, allowing for recognition by VHL for degradation [12].
	⊣ RNAi	miR_17_92	*Hif-1α* was found to be significantly downregulated in cell lines overexpressing the miR-17-92 microRNA cluster [13].
	⊣ IBind	FIH	*Hif-1α* binds FIH in an oxygen dependent manner, preventing *Hif-1α* transactivation. This provides a unifying mechanism to repress *Hif-1α* function under normoxic, but not hypoxic conditions [10].
PHD1_2	**PHD1_2** = not(**Hypoxia** or (**SMAD2_3_4** and **Rac1_H** and **Src_High**))		
	Enz	*PHD1/2* are prolyl hydroxylases responsible for hydroxylating the oxygen-dependent degredation domain of *Hif-1α*, leading to ubiqitination by VHL protein under normoxic conditions. *PHD1/2* require molecular oxygen for hydroxylation [11].	
	⊣ Ind	Src_High	High levels of active *Src* inhibit *PHD2* activity and thus stabilize *Hif-1α* by an *NADPH oxidase / Rac1*-dependent mechanism that generates cellular ROS, which in turn reduces vitamin C levels required for the activity of *PHD1/2* [15].
	⊣ Ind	Rac1_H	*Src*-mediated *PHD2* inhibition requires *Rac1* [15]. Here we assume only high levels of active *Rac1* mediate this effect; moderate *Rac1* activation in migrating epithelial cells do not.
	⊣ TR	SMAD2_3_4	Exogenous *TGF-β* has been demonstrated to decrease the amount of *PHD* mRNA within 4-8 hours of a 10uM dose through SMAD signaling, thus leading to decreased *PHD2* hydroxylated targets and increased *Hif-1α* expression. By adding SMAD inhibitors, this effect was abrogated [16].
	⊣ ComplProc	Hypoxia	Hydroxylation by *PHD1/2* is oxygen dependent [11].
pVHL	**pVHL** = not **Hypoxia**		
	UbL	Von Hippel Lindau protein is the primary subunit of a ubiquitin ligase responsible for recognizing *Hif-1α*, leading to its oxygen-dependent degredation [11].	
	⊣ ComplProc	Hypoxia	Under normoxia, hydroxylated *Hif-1α* is recognized by *VHL*, causing its ubiqination. Under hypoxia, pVHL is unable to recognize *Hif-1α*, as it cannot be hydroxylated by oxygen dependent PHD [11]. Moreover, there is evidence that at least some of *VHL*'s non-cannonical effects on *TGFβ* receptor I expression are also blocked by hypoxia [17]. Thus, we assume that hypoxia blocks *VHL* protein function.
FIH	**FIH** = not **Hypoxia**		

Table S1c: Hypoxia_signaling module

	PTase	Factor Inhibiting HIF1, of *FIH*, is an asparaginyl hydroxylase that depends on Fe(II) and uses uses molecular O2 to modify its substrates. Under normoxia, it represses *Hif-1*α through inhibition of its transactivation domain [10] by hydroxylation of asparagine residues that mediate recruitment of the p300 cofactor [18].	
	⊣ ComplProc	Hypoxia	*FIH* is an asparaginyl hydroxylase that uses uses molecular O2 to modify its substrates, and is blocked by hypoxia [18].
miR_17_92	**miR_17_92 = Myc**		
	mRNA	miR-17-92 is a microRNA responsible for negatively regulating the hypoxic response pathway [13]. It is induced in dividing cells by *Myc*.	
	← TR	Myc	Increased levels of miR-17-92 are directly linked to *Myc* binding at the microRNA gene cluster under normoxic conditions [13].
ROS	**ROS = Hypoxia**		
	Env	ROS accumulation is associated with hypoxic conditions due to metabolic disruption in the mitochondria. [19]	
	← ComplProc	Hypoxia	Under hypoxia (but not anoxia), the ratio of ROS production from the mitochondrial increased significantly; an effect that requires ETC Complex III [19].

Table S1d: TGF_beta_env module

Target Node	Node Gate<br>Node Type<br>Link Type	Node Description<br>Input Node	Link Description
TGFb_ext	**TGFb_ext = TGFb_ext**		
	Env	The *TGFb_ext* node in our model represents an extracellular environment with saturating levels of *TGF-*β. This input node is self-sustaining in the absence of *in silico* perturbation.	
	← Env	TGFb_ext	The *TGFb_ext* input node is self-sustaining in the absence of *in silico* perturbation.
Self_Loop	**Self_Loop = Self_Loop**		
	Env	The *Self_Loop* input node qualtifies the extent to which *TGF-*β secreted by a single isolated cell can drive its own autocrine signaling to saturating levels. When tuned stochastically between 0 and 1, its level represents the fraction of saturating *TGF-*β signal driven by the autocrine loop. We expect the fraction that accurately characterises a given cell to be a funciton of both cell type (intrinsic ability to secrete *TGF-*β) and microenvironment, as the latter can influence diffusion and bio-availability of the secreted ligand.	

← Env	Self_Loop	The *Self_Loop* input node is self-sustaining in the absence of *in silico* perturbation.

Table S1d: TGF_beta_env module

Table S1e: TGF_beta module

Target Node	Node Gate / Node Type / Link Type	Node Description / Input Node	Link Description
TGF_bRII	**TGF_bRII** = **TGFb_ext** or (**TGFb_secr** and (**CellDensity_Low** or **Self_Loop**))		
	Rec	The ON state of the *TGF_bRII* node represents a *TGF-β*-bound *TβRII* receptor, and thus requires external *TGF-β* [20], or secreted *TGF-β* along with either strong autocrine signaling (*Self_Loop*=ON) or at least medium cell density (*CellDensity_Low*=ON).	
	← ComplProc	CellDensity_Low	Though direct experimental evidence is hard to find, our model makes the assumption that autocrine signaling among cells that have at least medium density is strong enough to support autocrine *TGF-β* signaling, even if a single isolated cell's secretion is not sufficient. The assumption is indirecty supported by evidence that autocrine signaling is required for, and can indeed support the maintenance of a mesencymal state even after external *TGF-β* is no longer supplied [21, 22].
	← Ligand	TGFb_ext	Externally supplied *TGF-β* binds to and activates *TβRII* (*TGFBR2* gene) [20].
	← ComplProc	Self_Loop	In cells / environments where a single cell can secrete sufficient *TGF-β* to saturate its own *TGF-β* signaling, this input removes the requirement of neighboring cells to boost the availability of secreted *TGF-β*.
	← Ligand	TGFb_secr	Mesenhymal cells secrete *TGF-β*, creating an autocrine signaling loop required to maintian their mesenchymal state [21, 22].
TGF_bRI	**TGF_bRI** = **TGF_bRII** or (**TGFb_ext** or (**TGFb_secr** and ((**CellDensity_Low** or **Self_Loop**) and not **pVHL**)))		
	Rec	The ON state of *TβRI* represents an active, ligand-bound receptor complex of *TGF-β*, *TβRII*, and *TβRI* [20]. Thus it requires *TGF-β*-bound *TβRII*, where *TGF-β* is either externally supplied or secreted *TGF-β* (the latter requiring strong autocrine signaling or medium cell density).	
	← ComplProc	CellDensity_Low	Our model assumes that autocrine signaling among cells that have at least medium density is strong enough to support autocrine *TGF-β* signaling, even if a single isolated cell's secretion is not [21, 22].
	← Ligand	TGFb_ext	Externally supplied *TGF-β* binds to and activates *TβRII*, which in turn recruits and phosphorylates *TβRI* [20].

Table S1e: TGF_beta module

	← ComplProc	Self_Loop	In cells / environments where a single cell can secrete sufficient *TGF-β* to saturate its own *TGF-β* signaling, this input removes the requirement of neighboring cells to boost the availability of secreted *TGF-β*.
	← P	TGF_bRII	When *TGF-β* binds to its type II receptor (*TβRII*), it recruits the type I receptor *TβRI* and activates it by phosphorylation [20].
	← Ligand	TGFb_secr	Mesenhymal cells secrete *TGF-β*, creating an autocrine signaling loop required to maintian their mesenchymal state [21, 22].
	⊢ Ubiq	pVHL	*TGF-β receptor I* ubiquination is mediated by VHL protein, attenuating *TGF-β* signaling [23].
SMAD2_3_4	**SMAD2_3_4** = **TGF_bRI** and **TGF_bRII** and not(**SPRY2** and **pVHL**) and((**YAP** and **TAZ**) or not **ApicalBasal_Pol**)		
	PC		The *SMAD2_3_4* node represents transcriptionally active *Smad2/Smad3/Smad4* complexes. *Smad2* and *Smad3* are activated via phosphorylation by *TβRI*, which releases them from the receptor complex to aid their nuclear translocation in partnership with *Smad4* [20]. *SPRY2* reduces *Smad* phosphorylation in dense monolayers [24], which also sequester *TGF-β* receptors to their baso-lateral surface, hiding them from apically applied *TGF-β* [25]. In contrast, *YAP* and *TAZ* bind to *Smads*, aid their nuclear localization, and potentiate *Smad*-induced transcription [26]. Our model assumes that in order to respond to *TGF-β*, cells either need active *YAP/TAZ*, or no apical-basal polarizaiton.
	⊢ DP	SPRY2	*SPRY2* supresses *Smad2* phosphorylation and can block *TGF-β* induced EMT [24].
	← Loc	YAP	*YAP/TAZ* bind to *Smad2* and aid their nuclear accumulation and *Smad*-induced transcription. Fibroblast growth on soft gels with cytosolic *YAP/TAZ* or treated with chemical *YAP/TAZ* inhibitors show impaired *TGF-β*–induced *Smad2/3*-driven transcription [26].
	← Loc	TAZ	*YAP/TAZ* bind to *Smads* and aid their nuclear accumulation and *Smad*-induced transcription [26].
	⊢ Ind	ApicalBasal_Pol	Epithelial cell polarization was shown to block *TGF-β* signaling upstream and independently of cytoplasmic *YAP/TAZ* sequestration. In cells polarized along their apical-basal axis (represented in our model by *ApicalBasal_Pol* = ON), *TGF-β* receptors I and II are sequestered to the basolateral surface of the cell, depriving apically delivered *TGF-β* of access to its receptors and thus weakening the *TGF-β* response [25].
	← P	TGF_bRII	The actions of *TβRI* on *Smad2* and *Smad3* require an active receptor/ligand complex, and thus *TGF_bRII* [20].

Table S1e: TGF_beta module

	← P	TGF_bRI	The type I *TGF-β* receptor (*TβRI*) phosphorylates receptor-bound *Smad2* and *Smad3* at their carboxy-terminal, which releases them from the receptor complex and triggers their nuclear translocation. *Smad4* partners with activated *Smads* to help carry out their function [20].
	⊢ Ubiq	pVHL	VHL protein recognizes the MH2 domain of SMAD3, leading to its ubiqitination, supressing *TGF-β* signaling [27].
ILK_Rictor	**ILK_Rictor** = (**ILK** and **TGF_bRI**) and **TGF_bRII**		
	PC		*TGFβ-1* induces expression of the *mTORC2* component *Rictor*, *ILK* binding to *Rictor*, and *ILK*-dependent *Rictor* phosphorylation [28]. The complex, in turn, is known to activate *AKT1* [29].
	← Compl	ILK	*TGFβ-1* induces *ILK* binding and phosphorylation of *Rictor* [28]; we assume this requres an active *ILK* kinase.
	← Ind	TGF_bRII	*ILK/Rictor* binding is mediated by *TGFβ* receptors I and II, responsive to *TGFβ-1* [28].
	← Ind	TGF_bRI	*ILK/Rictor* binding is mediated by *TGFβ* receptors I and II, responsive to *TGFβ-1* [28].
HMGA2	**HMGA2** = **SMAD2_3_4**		
	TF		*HMGA2* is a transcription factor induced by *SMAD 3/4* in response to *TGF-β*. Once induced, it drives transcription of the master EMT switch by inducing *SNAI1/2* and *Twist* [30].
	← TR	SMAD2_3_4	*HMGA2* is a direct transcriptonal target of *TGF-β* induced *SMAD 3/4* [30].
p15	**p15** = **SMAD2_3_4** or ((**FoxO1** or **FoxO3**) and not **Myc**)		
	CDKI		*p15* (*Ink4B*) is a cyclin-dependent kinase inhibitor that binds *cdk4* / *cdk6*, displacing *Cyclin D* and thus blocking G1/S progression [31]. *TGFβ* induces both transcription [32] and stabilization of *p15* [33], while *Myc* represses its transcription [34]. In addition, *FoxO1/3* can also induce *p15* [35].
	← TR	FoxO3	*FoxO3* is a direct transctiptional activator of *p15* [35].
	← TR	FoxO1	*FoxO1* is a direct transctiptional activator of *p15* [35].
	← TR	SMAD2_3_4	*Smad2*, *Smad3* and *Smad4* induce *p15* transcription and increase its protein stability in response to *TGFβ* [32].
	⊢ TR	Myc	*Myc* is a direct transctiptional repressor of *p15* [34].
DUSP4	**DUSP4** = **SMAD2_3_4**		
	Ph		*TGFβ* induces the transcription of the mitogen-activated protein kinase phosphatase *MKP2*, or *DUSP4*, through a *SMAD3*-dependent mechanism [36]. *DUSP4*, in turn, attenuates *ERK* and allows *BIM* accumulation, aiding *TGFβ*-mediated apoptosis [36, 37].

Table S1e: TGF_beta module

	← TR	SMAD2_3_4	*TGFβ* induces *DUSP4* transcription through *SMAD3* [36].
Runx1	**Runx1** = **SMAD2_3_4**		
	TF	The *Runx1* transcription factor is transcriptionally induced by *TGFβ*, leading to elevated mRNA and protein levels [38]. *Runx1* in turns binds to *FoxO3* to induce *BIM* and promote *TGFβ*-mediated apoptosis [38].	
	← TR	SMAD2_3_4	Either *Smad2* or *Smad3*, plus *Smad4*, are required for induction of *Runx* transcription factors by *TGFβ*1 [39].

Table S1f: GF_Basal_MAPK module

Target Node	Node Gate<br>Node Type<br>Link Type	Node Description<br>Input Node	Link Description
RTK	**RTK** = not **CAD** and (**GF_High** or **GF**)		
	Rec	The ON state of the *RTK* node in our model represents basal growth receptor activation (required to keep a normal cell alive). Thus it requires the absence of *CAD* and at least low growth levels of growth factors in the extracellular environment [40].	
	← Ligand	GF	The ON state of the *RTK* node in our model represents basal growth receptor activation by low / medium growth factor availability, encoded by the *GF* node (required to keep a normal cell alive).
	← Ligand	GF_High	Similarly, high growth factor availability also keeps *RTK* on.
	⊣ Per	CAD	Caspase-activated DNase (*CAD*) inhibition of receptor tyrosine kinases ensures that apoptotic cells no longer maintain even basal levels of growth signaling.
Shc	**Shc** = ((**RTK** and **GF_High**) or (**TGF_bRI** and **TGF_bRII**)) and (**FAK** or **Src**)		
	Adap	*Shc* is ON when *RTK*s are activated by high levels of extracellular growth factors (capable of driving proliferation) [41] or *TGFβ* activates *TβRI-TβRII* [42], and its recruitment is aided by active *Src* kinase [43] or focal adhesion kinase (*FAK*) [43]. The two mediators of integrin signaling appears to be able to act independently, forming two parallel links between integrin signaling and full *RTK* activation [43].	
	← Compl	GF_High	The ON state of *Shc* in our model encode the change from basal *Shc* recruitment to weakly stimulated *RTKs* to the level of recruitment seen in high growth factor environments, capable of mediating *Ras* activation.
	← Compl	RTK	*Shc* proteins are adaptors that binds to phosphor-tyrosine motifs, facilitating their recruitment to activated receptors such as receptor tyrosine kinases *RTKs* [41].

Table S1f: GF_Basal_MAPK module

	← Compl	FAK	*FAK* activated at sites of integrin-ECM attachments directly phosphorylates *Shc Tyr-317*, promoting its ability to assemble MAPK-inducing signaling scaffolds, including *Grb2* binding [43].
	← Compl	Src	*c-Src* recruited to and activated by integrin-ECM attachments directly phosphorylate *Shc*, promoting its ability to assemble MAPK-inducing signaling scaffolds, including *Grb2* binding [43].
	← Compl	TGF_bRII	*TGFβ* receptor II (*TβRII*) can be phosphorylated by *Src* on Y284, which provides a docking site for *Grb2* and *Shc*, and subsequent MAPK activation [42].
	← Compl	TGF_bRI	*TβRII* activation requires complex formation with *TβRI* upon ligand binding [42].
Grb2	**Grb2** = (**RTK** or (**TGF_bRI** and **TGF_bRII**)) and **Shc**		
	Adap	*Grb2* is recruited to *RTKs* or *TGFβ* receptors upon ligand binding and subsequent recruitment of *Shc* adaptors [44, 42].	
	← Compl	RTK	The SH2 domain of *Grb2* binds to a phosphotyrosine residue in the activated *RTK*, where it functions as an adaptor protein [44].
	← Compl	Shc	*Shc* proteins phosphorylated by tyrosine kinases represent binding sites for *Grb2*, aiding its recruitment to active RTKs [41].
	← Compl	TGF_bRII	*TβRII* phosphorylated by *Src* on Y284, provides a docking site for *Grb2* and *Shc* [42].
	← Compl	TGF_bRI	*TβRII* works in complex with *TβRI* and their ligand [42].
SOS	**SOS** = **Grb2**		
	GEF	*RTK*-bound *Grb2* recruits *SOS*, a guanine nucleotide–exchange protein (GEF) that converts inactive *Ras* to its active GTP-bound form [44].	
	← Compl	Grb2	*Grb2* recruits *SOS* to activated *RTK*s [44].
Ras	**Ras** = **Grb2** and **SOS** and **Src** and (**IQGAP1_LeadingE** or not **Merlin** or **N_bcatenin_H**)		
	GTPa	*Ras* activation requires the GEF activity of *SOS* and the *RTK*-linked (active) adaptor protein *Grb2* [44] and *Src* [45, 46]. In addition to aiding sustained *Ras/Raf-1* signaling, *Src* may also physically link *IQGAP1* to *RTKs* such as *VEGFR2* [47]. *IQGAP1*, in turn, serves a scaffold for *MAPK* and *PI3K* signaling [48], leading us to link its activation at the leading edge. In contrast, *Merlin* blocks *Ras* activation at sites that link focal adhesions and actin filaments to *MAPK* signaling [49]. Here we assume that concentrated *IQGAP1* at the leading edge can override remaining *Merlin* activity in the rest of the cell. Alternatively, high levels of *β-catenin* can also sustain *Ras* by protecting it from lysosomal degradation [50].	

Table S1f: GF_Basal_MAPK module

	$\underset{\text{Compl}}{\leftarrow}$	Grb2	*RTK*-bound *Grb2* is required to recruits *SOS*, the GEF responsible for converting inactive *Ras* to its GTP-bound active form [44].
	$\underset{\text{GEF}}{\leftarrow}$	SOS	*SOS* is a GEF that is recruited to activate *Ras* near ligand-bound, active *RTKs* [44].
	$\underset{\text{ComplProc}}{\leftarrow}$	Src	Cellular Src (*c-Src*) is required for mitogenesis initiated by multiple growth factor receptors, including epidermal growth factor (*EGF*), platelet-derived growth factor (*PDGF*), colony stimulating factor-1 (*CSF-1*), and basic fibroblast growth factor (*bFGF*) [45]. In addition to aiding the formation of Shc/Grb2/SOS/Ras/Raf-1 cascade, *Src* may also increase the rate of receptor internalization and aid sustained *MAPK* signaling by internalized *Ras* on endosomes and Golgi [45, 46].
	$\underset{\text{ComplProc}}{\vdash}$	Merlin	*Merlin* uncouples *Ras* from growth factor signals by counteracting the ERM (ezrin, radixin, moesin)–dependent activation of *Ras*, which aids *Grb2*, *SOS*, *Ras* complex formation linked to filamentous actin [49].
	$\underset{\text{Compl}}{\leftarrow}$	IQGAP1 _LeadingE	*IQGAP1* acts as a scaffold for the *MAPK* cascade, binding directly to *B-Raf*, *MEK*, and *ERK* and regulating their activation [51]. The *IQGAP1-Leading-E* node in our model specifically links the availability of active *IQGAP1* recruited to lamellipodia and enhanced *MAPK* / *AKT* signaling.
	$\underset{\text{Ind}}{\leftarrow}$	N_bcatenin _H	High levels of *β-catenin* protect *Ras* from lysosomal degradation [50]. Overall, *β-catenin* overexpression / silencing can activate / block *ERK* in a *MEK*-dependent way, like via *Ras/Raf* [52].
RAF	**RAF** = **Ras** and not **SPRY2** and not **Casp3**		
	K	*Raf* is active in response to *Ras* activity in the absence of *Caspase 3*. As active *Raf-1* is continuously dephosphorylated and bound by *14-3-3*, which translocates it to the cytoplasm from the plasma membrane (not modeled explicitly), ongoing *Ras* activity [53] and lack of *SPRY2* inhibition [54, 55] are necessary to keep *Raf* ON.	
	$\underset{\text{P}}{\leftarrow}$	Ras	Active *Ras* phosphorylates *Raf*, enhancing its kinase activity [53].
	$\underset{\text{IBind}}{\vdash}$	SPRY2	Sprouty2 (*SPRY2*) blocks Raf activity and downstream MAPK signaling [54, 55].
	$\underset{\text{Lysis}}{\vdash}$	Casp3	*Raf-1* is cleaved and inhibited by *Caspase 3* [56].
MEK	**MEK** = **RAF**		
	K	*Raf* phosphorylates and activates the *MEK* kinase [53].	
	$\underset{\text{P}}{\leftarrow}$	RAF	*Raf* phosphorylates and activates the *MEK* kinase [53].
ERK	**ERK** = **MEK** and not **BIK** and (**FocalAdhesions** or not **DUSP4** or **N_bcatenin_H**)		

Table S1f: GF_Basal_MAPK module

	K	The *ERK* kinase is active when phoisphotylated by *MEK* [53] and allowed to translocate to the nucleus in the absence of *BIK* [57]. Here we assume that in the presence of *DUSP4* [36, 37], *ERK* activity can be maintained /compensated by either strong focal adhesion assembly (*FocalAdhesions* node) [58], or high levels of *β-catenin* [52].	
	← P	MEK	*MEK* phosphorylates and activates the *ERK* kinase [53].
	← ComplProc	FocalAdhesions	Focal adhesions recruit the MAPK scaffolding protein GIT1 and locally potentiate *ERK1/2* activation [58].
	⊣ DP	DUSP4	*DUSP4* is a phosphatase that blocks *ERK* and allows *BIM* accumulation in response to *TGFβ*, aiding apoptosis [36, 37].
	← Ind	N_bcatenin_H	*β-catenin* overexpression / silencing can activate / block *ERK* in a *MEK*-dependent way [52].
	⊣ IBind	BIK	*BIK* binds to active, phosphorylated *ERK1/2* and suppresses its nuclear translocation [57].
MCRIP1	**MCRIP1** = not **ERK**		
	Prot	*MCRIP1* is a binding partner and repressor of *CtBP*, a binding partner of *Zeb1* during transcription , and thus *MCRIP1* blocks *Zeb1*-mediated repression of *E-cadherin* unless inhibited by *ERK* phosphorylation [59].	
	⊣ P	ERK	Upon *ERK* phosphorylation *MCRIP1*, an inhibitor of EMT, dissociates and releases the *ZEB1* co-activator *CtBP* [59].
mTORC2	**mTORC2** = **PIP3** or not **S6K**		
	PC	Our model assumes that *mTORC2* is active in quiescent cells with basal levels of *PI3K* activity leading to basal *PIP3* generation. Alternatively, the absence of high growth factor-stimulated *mTORC1* and *S6K1* can also increase *mTORC2* activity.	
	← PBind	PIP3	PtdIns(3,4,5)P3 (*PIP3*), interacts with the *mTORC2* component *Sin1* to release its inhibition on the *mTOR* kinase domain. Thus, *PIP3* is necessary for *mTORC2* activation [60].
	⊣ P	S6K	*Rictor*, a component of the *mTORC2 complex*, undergoes *S6K1*-mediated phosphorylation at T1135, dampening *mTORC2*-dependent phosphorylation of *Akt* [61, 62].
PI3K	**PI3K** = ((**FAK** or **Src**) and (**Ras** or **RTK**)) or (**TGF_bRI** and **TGF_bRII**)		
	K	In our model, basal *PI3K* activity can be maintained by active *RTK*s [40], active *Ras* [63, 64], or active *TβRI-TβRII* [65]. In addition, survival signalling via *PI3K* requires anchorage-dependent signals via active *FAK* [66] or via *Src*-mediated blocking of basal *PTEN* activity (not modeled explicitly) [67].	
	← BLoc	RTK	Active *RTK*s recruit *PI3K* to the signaling complex they nucleate, where *PI3K* catalyzes the production of PtdIns(3,4,5)P3 (PIP3) [40].

Table S1f: GF_Basal_MAPK module

	$\leftarrow$ Compl	Ras	*Ras* binds the catalytic subunit of *PI3K* and *Ras* knockdown / over expression decreases /increases the *PI3K*-dependent generation of PIP3 [63, 64].
	$\leftarrow$ PLoc	FAK	Attachment to the ECM activates *FAK* kinase, which promotes anchorage-dependent survival signaling via *PI3K* / *AKT* [66].
	$\leftarrow$ P	Src	*Src* kinases regulate *PI3K* signaling cascade by altering the function of the *PTEN* tumor suppressor via inhibitory phosphorylation [67].
	$\leftarrow$ Compl	TGF_bRII	*TGFβ* induces *PI3K* activation and *AKT* phosphorylatio, a pathway required for EMT [68]. This activation requires *TβRI* and *TβRII* kinase activity [65].
	$\leftarrow$ Compl	TGF_bRI	*TGFβ* induced *PI3K* activation requires *TβRI* and *TβRII* kinase activity [68, 65].
PIP3	**PIP3** = **PI3K_H** or **PI3K**		
	Met		In our model, PIP3 is ON as a result of basal or high *PI3K* activity.
	$\leftarrow$ Cat	PI3K	Active *PI3K* recruited to the membrane catalyzes the production of membrane-bound PtdIns(3,4,5)P3 (PIP3) from PtdIns(4,5)P2 (PIP2) [40].
	$\leftarrow$ Cat	PI3K_H	Active *PI3K* recruited to the membrane catalyzes the production of membrane-bound PtdIns(3,4,5)P3 (PIP3) from PtdIns(4,5)P2 (PIP2) [40].
PDK1	**PDK1** = **PI3K** and **PIP3**		
	K		*PDK1* enzyme activation requires active (at least basal) *PI3K* and *PIP3* [69].
	$\leftarrow$ BLoc	PI3K	The *PDK1* kinase is recruited to the plasma membrane by *PIP3* at the sites of active *PI3K* activity [69].
	$\leftarrow$ BLoc	PIP3	The *PDK1* kinase is recruited to the plasma membrane by *PIP3* at the sites of active *PI3K* activity [69].
AKT_B	**AKT_B** = **PIP3** and (**PDK1** or **mTORC2**) and not **Casp3**		
	K		Basal *AKT1* activity in our model requires the absence of *Caspase 3*, the availability of at least basal levels of *PIP3*, and phosphorylation by *PDK1* or *mTORC2*. In contrast, full mitogen-stimulated *AKT1* activation requires phosphorylation by both (see *AKT_H*) [69].
	$\leftarrow$ P	mTORC2	Maximal activation of *AKT1* requires phosphorylation of S473 by *mTORC2* [69].
	$\leftarrow$ BLoc	PIP3	*PIP3* recruits *AKT1* to the plasma membrane and *PIP3* binding changes the conformation of *AKT1* such that it becomes accessible for T308 phosphorylation by *PDK1* [69].
	$\leftarrow$ P	PDK1	Membrane-recruited *PDK1* phosphorylates *AKT1* at T308, a critical step in its activation [69].

	⊣ Lysis	Casp3	*AKT1* is cleaved and inhibited by *Caspase 3* [56].

Table S1g: GF_PI3K module

Target Node	Node Gate<br>Node Type<br>Link Type	Node Description<br>Input Node	Link Description
p110_H	**p110_H** = **YAP**and((**FoxO3**andnot **Nedd4L**)or(**p110_H**and(**FoxO3**ornot **Nedd4L**)))		
	Prot	As *YAP* is a transcriptional inducer of *p110* subunits [70] and their expression is low in high cell density areas where *YAP* activity is suppressed [71], here we assume that high *p110* expression requires active *YAP*. In order to capture the cyclic dynamics of *p110* protein expression, we make the assumption that high *p110* protein levels can be induced by *FoxO3* in the absence of the growth factor-activated *Nedd4L* ubiquitin ligase. Once present, high *p110* can be maintained by *FoxO3* transcription, or the absence of activated *Nedd4L*.	
	← Per	p110_H	Our model assumes that maintaining high *p110* levels is easier than driving the re-accumulation of the protein following its rapid destruction.
	← TR	FoxO3	*FoxO3* is a direct inducer *p110α* (*PIK3CA*), the catalytic subunit of *PI3K* [72].
	⊣ Ubiq	Nedd4L	*p110α* (*PIK3CA*) is polyubiquitinated by the E3 ligase *Nedd4L*, leading to its proteasomal degradation. Both free *p110α* and the regulatory subunit-bound protein is subject to ubiquitination by *Nedd4L* [73].
	← TR	YAP	*YAP* is a transcriptional inducer of both catalytic *p110* subunits of *PI3K*, *p110a* and *p110b*; *p110-H* albeit its effect on *p110a* expression requires raising *p110b* levels first. Moreover, *YAP* knockdown leads to downregulation of both subunits [70].
PI3K_H	**PI3K_H** = **p110_H** and **PI3K** and not **PTEN_c** and (**RTK** or (**TGF_bRI** and **TGF_bRII**)) and **Ras**		
	K	Full, peak-level activation of *PI3K* requires high levels of *p110* protein, basal *PI3K* activation, active *Ras*, and active *RTK*s or *TβRI-TβRII*. As the ON-state of *Ras* in our model represents strong *Ras* activation in the presence of proliferation-inducing (high) growth factors, *PI3K_H* activation can only occur in these conditions. In addition, a reduction of cytoplasmic *PTEN* levels is also required for peak *PI3K* activity.	
	← BLoc	RTK	High levels of *PI3K* activation only occur at growth factor-bound *RTK*s, which recruit and activate *PI3K* at the plasma membrane [69].
	← Compl	Ras	*Ras* binds the catalytic subunit of *PI3K* and *Ras* knockdown / over expression decreases /increases the *PI3K*-dependent generation of PIP3 [63, 64].

Table S1g: GF_PI3K module

	← Per	PI3K	In our model, high *PI3K* activation is contingent on the ON-state of the basal *PI3K* node.
	← Per	p110_H	High levels of *PI3K* activity in response to strong growth factor stimulation only occur in cells that express high levels of *p110* protein [71].
	⊢ DP	PTEN_c	Cytoplasmic *PTEN* regulates *PI3K* signaling by dephosphorylating its lipid signaling intermediate *PIP3* [74].
	← Compl	TGF_bRII	*TGFβ* induced *PI3K* activation requires *TβRI* and *TβRII* kinase activity [68, 65].
	← Compl	TGF_bRI	*TGFβ* induced *PI3K* activation requires *TβRI* and *TβRII* kinase activity [68, 65].
AKT_H	**AKT_H** = **AKT_B** and **p110_H** and (**PI3K_H** or (**PI3K** and **ILK_Rictor**)) and **PIP3** and **PDK1** and (**mTORC2** or **ILK_Rictor**) and (**Ras** or **PAK1**)		
	K		In contact to basal *AKT1*, high *AKT1* activity in our model requires basal *AKT1* (*AKT_B*), the ongoing presence of high *p110* protein levels along with active *PI3K_H* and *PIP3*. In addition this maximal *AKT1* activation requires phosphorylation by both *PDK1* and *mTORC2*, , as well as either active *Ras* [69] or *PAK1* [75]. In response to *TGFβ*, high *AKT1* activity can also be induced by *PI3K* and the *ILK-Rictor* complex; the latter capable of replacing *mTORC2* to supply S473 phosphorylation of *AKT1* [28, 29].
	← Compl	Ras	*Ras* binding to the catalytic subunit of *PI3K* is required for its full potency in *PIP3* generation [63, 64]. Active *Ras* is thus required for inducing peak *AKT_H* activity.
	← P	mTORC2	Maximal activation of *AKT1* requires phosphorylation of S473 by *mTORC2* [69].
	← P	PI3K	As maximal *AKT1* activity also requires T308 phosphprylation by *PDK1* recruited to the membrane by *PI3K* [69], we assume that *ILK/Rictor* complexes can only dirve high *AKT* activity in the presence of at least basal *PI3K* activation.
	← Compl	PIP3	*PIP3* recruits *AKT1* to the plasma membrane and *PIP3* binding changes the conformation of *AKT1* such that it becomes accessible for T308 phosphorylation by *PDK1* [69].
	← P	PDK1	Membrane-recruited *PDK1* phosphorylates *AKT1* at T308, a critical step in its activation [69].
	← Per	AKT_B	In our model, high *AKT1* activation is contingent on the ON-state of basal *AKT1* (*AKT_B*).
	← P	p110_H	Ongoing high *p110* availability and *PI3K_H* activity are required to induce maximal activation of *AKT_H* [69].
	← P	PI3K_H	Ongoing high *p110* availability and *PI3K_H* activity are required to induce maximal activation of *AKT_H* [69].

Table S1g: GF_PI3K module

	$\leftarrow$ P	PAK1	*PAK1* interacts with and directly phosphorylates *AKT1* [76]. In addition, *PAK1* provides a scaffold to facilitate *Akt* stimulation by *PDK1* and to aid *AKT's* membrane recruitment [75].
	$\leftarrow$ P	ILK_Rictor	Maximal activation of *AKT1* requires phosphorylation of S473. In response to *TGF-β*, *ILK* complexes with *Rictor* to phosphoprylate *AKT1* [28, 29].
FoxO3	**FoxO3** = not(**AKT_B** or **AKT_H** or **ERK**) or (not(**AKT_H** and (**Plk1** or **Plk1_H** or **AKT_B** or **ERK**)) and not(**Plk1** and **Plk1_H** and **ERK**))		
	TF		In order to account for all the influences on *FoxO3* activity, we used the following logic. In the absence of basal or high *AKT1* as well as *ERK*, *FoxO3* remains active. In addition, *FoxO3* can overcame peak (*AKT_H*) activation only if no other inhibitor is present and *AKT_B* is OFF (indicating that *AKT1* levels are falling). Finally, the joint activity of *ERK* and *Plk1* can also block *FoxO3*.
	$\vdash$ P	ERK	ERK downregulates *FoxO3* transcriptional activity by phosphorylating it at three Serines, inducing its *MDM2*-mediated ubiquitination and degradation [77].
	$\vdash$ PLoc	AKT_B	*AKT1* mediates the translocation of the *FoxO3* our of the nucleus through direct phosphorylation of three conserved residues. These events create a recognition site for *14-3-3* family proteins, which export and sequester *FoxO3* in the cytosol [69].
	$\vdash$ PLoc	AKT_H	*AKT1* mediates the translocation of the *FoxO3* our of the nucleus through direct phosphorylation of three conserved residues. These events create a recognition site for *14-3-3* family proteins, which export and sequester *FoxO3* in the cytosol [69].
	$\vdash$ PLoc	Plk1	*Plk1* binds *FoxO3*, induces its translocation to the cytosol, phosphorylates it and suppresses its activity through most of the the cell cycle, but most significantly during G2 and M [78].
	$\vdash$ PLoc	Plk1_H	*Plk1* binds *FoxO3*, induces its translocation to the cytosol, phosphorylates it and suppresses its activity through most of the the cell cycle, but most significantly during G2 and M [78].
PLCgamma	**PLCgamma** = **RTK** and **Grb2** and **GF_High** and **p110_H** and **PI3K_H** and **PIP3**		
	Enz		Peak activation of *PLCγ* requires active an *RTK* receptor node bound by active *Grb2*, as well as high *PI3K* activity (including high *p110* availability and the presence of PIP3).
	$\leftarrow$ Compl	GF_High	We assume that high levels of *RTK* activity is required for tyrosine phosphorylation of *PLCγ*.
	$\leftarrow$ P	RTK	The SH2 domains of *PLCγ* binds to active *RTK*s at tyrosine autophosphorylation sites, leading to tyrosine phosphorylation of *PLCγ* and stimulation its enzymatic activity [79, 80].

Table S1g: GF_PI3K module

	← BLoc	Grb2	*RTK* tyrosine autophosphorylation induces $PLC\gamma$ binding to the *Grb2* adaptor protein and likely aids the translocation of $PLC\gamma$ to the plasma membrane [81].
	← BLoc	PIP3	Membrane targeting of $PLC\gamma$ to growth receptor stimulation is mediated by *PIP3* binding of $PLC\gamma$ [82, 83].
	← P	p110_H	Membrane targeting of $PLC\gamma$ to growth receptor stimulation requires *PI3K* activity and *PIP3* generation near growth receptors [82]. Thus, peak $PLC\gamma$ activity in our model requires high *p110* protein expression [83].
	← P	PI3K_H	In addition to high *p110* protein levels, high *PI3K* activation is also required to fully activate $PLC\gamma$ [82, 83].
IP3	**IP3 = PLCgamma**		
	Met	Membrane-bound, active $PLC\gamma$ is responsible for converting phosphatidylinositol(4,5)P2 (*PIP2*) to the second messenger inositol(1,4,5)P3 (*IP3*) responsible for triggering a sudden Ca^{2+} influx from the endoplasmic reticulum, along with *DAG* (diacylglycerol, another second messenger) [84].	
	← Cat	PLCgamma	Membrane-bound, active $PLC\gamma$ is responsible for converting phosphatidylinositol(4,5)P2 (*PIP2*) to the second messenger inositol(1,4,5)P3 (*IP3*) responsible for triggering a sudden Ca^{2+} influx from the endoplasmic reticulum, along with *DAG* (diacylglycerol, another second messenger) [84].
Ca2p	**Ca2p = IP3**		
	Met	*IP3* travels from the cell membrane to the endoplasmic reticulum where it opens *IP3*-sensitive Ca^{2+} channels, releasing a sudden Ca^{2+} efflux from the ER into the cytosol [85].	
	← Loc	IP3	*IP3* travels from the cell membrane to the endoplasmic reticulum where it opens *IP3*-sensitive Ca^{2+} channels, releasing a sudden Ca^{2+} efflux from the ER into the cytosol [85].
Nedd4L	**Nedd4L = Ca2p** and **IP3**		
	UbL	Activation of *Nedd4L* requires both Ca^{2+} and *IP3* binding [86].	
	← Compl	IP3	In order to transition to its active form, the E3 ubiquitin ligase *Nedd4L* binds Ca^{2+} and inositol 1,4,5-trisphosphate (*IP3*) [86].
	← Compl	Ca2p	In order to transition to its active form, the E3 ubiquitin ligase *Nedd4L* binds Ca^{2+} and inositol 1,4,5-trisphosphate (*IP3*) [86].

Table S1h: GF_mTOR module

Target Node	Node Gate		
	Node Type	Node Description	
	Link Type	Input Node	Link Description

Table S1h: GF_mTOR module

TSC2	**TSC2** = not **AKT_H** or not(**AKT_B** or **ERK**)		
	Prot	Blocking *TSC2* requires ongoing mitogen stimulation through *AKT* and/or *ERK*. In our model, *TSC2* inhibition requires high (peak) *AKT* activity, supported by either *ERK* or basal *AKT* (assuring that complete loss of *AKT* activity is not impending) [87].	
	$\vdash$ P	ERK	*ERK* phosphorylates *TSC2* directly, causing dissociation of the complex and inhibition of its activity [88]. In addition, the *ERK* target *p90RSK* can also inactivate *TSC2* [89].
	$\vdash$ P	AKT_B	*TSC2* is phosphorylated by *AKT1*, inhibiting it by dissociating *TSC2* from lysosomal membranes [90], where it stimulates GTP hydrolysis of the small GTPase *Rheb*, this inactivating it [87].
	$\vdash$ P	AKT_H	*TSC2* is phosphorylated by *AKT1*, inhibiting it by dissociating *TSC2* from lysosomal membranes [90], where it stimulates GTP hydrolysis of the small GTPase *Rheb*, this inactivating it [87].
PRAS40	**PRAS40** = not **AKT_H** and not(**mTORC1** and **AKT_B**)		
	Prot	*PRAS40* is inhibited by peak *AKT1* activity aided by either basal *AKT1* (meaning *AKT_H* is on its way down), or ongoing *mTORC1* activation. Both *AKT1* and *mTORC1* phosphorylate *PRAS40*, leading to its dissociation from *mTORC1* [91].	
	$\vdash$ P	AKT_B	*PRAS40* is phosphorylated by *AKT*, triggering its dissociation from *mTORC1* [92].
	$\vdash$ P	AKT_H	*PRAS40* is an inhibitory component of the *mTORC1* complex. It is phosphorylated by *AKT*, triggering its dissociation from *mTORC1* and loss of *mTORC1* inhibition [92].
	$\vdash$ P	mTORC1	*PRAS40* is a substrate of the *mTORC1* kinase; its phosphorylation aids its dissociation from *mTORC1* and its sequestration by 14-3-3 proteins [93].
DAG	**DAG** = **PLCgamma**		
	Met	Membrane-bound, active $PLC\gamma$ is responsible for converting phosphatidylinositol(4,5)P2 (*PIP2*) to the second messenger diacylglycerol (*DAG*), along with *IP3* [84].	
	$\leftarrow$ Cat	PLCgamma	Membrane-bound, active $PLC\gamma$ is responsible for converting phosphatidylinositol(4,5)P2 (*PIP2*) to the second messenger diacylglycerol (*DAG*), along with *IP3* [84].
Rheb	**Rheb** = not **TSC2** and **DAG**		
	GTPa	*PKC* (and *DAG*)-dependent activation of *mTORC1* recruits *mTORC1* to the site of *Rheb* activity (to prenuclear lysosomes), while *AKT* and *ERK*-mediated *TSC2* inhibition guarantees that *Rheb* remains potent [94, 95, 96].	
	$\vdash$ GAP	TSC2	*TSC2*, a key component of the heterotrimeric *TSC* complex, is a GTPase activating protein (GAP) that induces ATP hydrolysis and deactivation of the small GTPase *Rheb* [94].

Table S1h: GF_mTOR module

	← Ind	DAG	The second messenger *DAG* activates both classical and novel *PKC*s. One of its targets, $PKC\eta$, is responsible for the translocation and accumulation of *mTORC1* to perinuclear lysosomes, where the majority of *Rheb* is anchored. Thus, *DAG* brings *Rheb* in proximity with its target, *mTORC1* [95].
mTORC1	**mTORC1** = not **Casp3** and ((not **PRAS40** and **Hif1a_basal** and **Rheb** and not **Merlin**) or (**E2F1** and **ERK**) or (**CyclinB** and **Cdk1** and **GSK3**))		
	PC		*mTORC1* is activated by mitogenic signals via *Rheb* in the absence of *PRAS40*, aided by metabolic changes mediated by proliferation-linked *Hif1-α* activation (modeled as the *Hif1a_basal* node). This, however, also requires inactivation of *Merlin*, independently of *RTK*-induced signals. In addition, *E2F1* can promote *mTORC1* activity, likely aided by *ERK*-mediated phosphorylation of it's *Raptor* component. Finally, the mitotic *Cyclin B* and its *Cdk1* kinase can also activate *mTORC1*, aided by *GSK3*.
	← P	ERK	*ERK1* and *ERK2* bind to the *mTORC1* component *Raptor* and phosphorylate it at Ser8, Ser696, and Ser863, modifications that promote *mTORC1* activity [97]. Here we assume that *ERK* can help *E2F1* maintain active *mTORC1* in the presence of *AMPK* (*E2F1* was shown to override *mTORC1* inhibition by *TSC2* [98]).
	⊣ IBind	PRAS40	*PRAS40* is an inhibitory component of the *mTORC1* complex, removed by phosphorylation by *AKT* or *mTORC1* itself [87].
	← Compl	Rheb	The *Rheb* small GTPase binds *mTORC1* directly and activates the complex [99].
	← Ind	GSK3	During mitosis, *mTORC1* is activated by the G2/M-specific phosphorylation of *Raptor*, a component of *mTORC1*, by *CyclinB / Cdk1* complexes, aided by *GSK3* [100].
	⊣ ComplProc	Merlin	*Merlin* suppresses *mTORC1* activity via an unknown mechanism that appears to be independent of *PI3K/AKT* or of *TSC2* inhibition [101], and it suppression appears to be critical for integrin-mediated *mTORC1* activation [102].
	← Ind	Hif1a_basal	*Hif1-α* was found to be transiently stabilized ahead of the G1 phase of the cell cycle [103]. This increase in *Hif1-α* is required for the metabolic reprogramming and increased glycolysis required for cell cycle entry [103]. Increased glycolysis, in turn, aids *mTORC1* activation through a sugar-derived metabolite, independently of deactivaiton of the energy sensor *AMPK* (not modeled directly) [104].
	← Loc	E2F1	*E2F1* induces *mTORC1* activity by inducing *mTORC1* translocation to late endosomes. This effect does not require *AKT* and is not blocked by high levels of *TSC2* [98].
	← Ind	CyclinB	During mitosis, *mTORC1* is activated by the G2/M-specific phosphorylation of *Raptor*, a component of *mTORC1*, by *CyclinB / Cdk1* complexes, aided by *GSK3* [100].

Table S1h: GF_mTOR module

	← Ind	Cdk1	During mitosis, *mTORC1* is activated by the G2/M-specific phosphorylation of *Raptor*, a component of *mTORC1*, by *CyclinB / Cdk1* complexes, aided by *GSK3* [100].
	⊣ Lysis	Casp3	*Raptor*, a key component of the *mTORC1* complex, is cleaved and inhibited by *Caspase 3* [105].
S6K	**S6K** = not **Casp3** and **mTORC1**		
	K	*S6K* is activated by *mTORC1* in the absence of *Caspase 3*.	
	← P	mTORC1	*mTORC1* phosphorylates and activates 40S ribosomal S6 kinases (*S6K*s) [106].
	⊣ Lysis	Casp3	*S6K* is cleaved and inhibited by *Caspase 3* [107].
h_4EBP1	**h_4EBP1** = not **mTORC1** or **SMAD2_3_4**		
	Prot	*4EBP1*, or eukaryotic translation initiation factor 4E-binding protein 1, binds and sequesters the initiation factor *eIF4F* and thus limits translation. *mTORC1* phosphorylates and deactivates *4EBP* to upregulate protein biosynthesis [87]. While in our previous models this protein was implicit along the *mTORC1* → *eIF4F* link [108, 109], we include it here because *TGFβ* signaling induces *4EBP1* via *SMAD4* to block proliferative metabolism [110].	
	⊣ P	mTORC1	*mTORC1* phosphorylates *4EBP*, triggering its dissociation from *eIF4F* [87].
	← TR	SMAD2_3_4	*4EBP1* is a transcriptional target of *SMAD4*, and its increase is critical for *TGF-β* mediated inhibition of proliferation [110].
eIF4E	**eIF4E** = not(**Casp3** or **h_4EBP1**)		
	Prot	*eIF4E* is activated by *mTORC1*-mediated repression of *4EBP1*, which normally binds to it and blocks its activity [87]. *eIF4E* is cleaved and deactivated by *Caspase 3* [111].	
	⊣ IBind	h_4EBP1	*mTORC1*-phosphorylated *4EBP* dissociates from *eIF4F*, allowing it to initiate translation [87].
	⊣ Lysis	Casp3	*eIF4E* is cleaved and inhibited by *Caspase 3* [111].

Table S1i: GF_connect module

Target Node	Node Gate<br>Node Type<br>Link Type	 Node Description<br>Input Node	 <br>Link Description
GSK3	**GSK3** = not **AKT_H** and not((**S6K** and **ERK**) or **Hif1a_High**)		
	K	*GSK3* activity can be completely blocked by peak *AKT* activation (*AKT_H*), or by the joint action of *S6K* and *ERK*.	

Table S1i: GF_connect module

	⊣ P	ERK	*ERK* binds and phosphorylates *GSK3β* at Thr-43, which primes it for subsequent phosphorylation by the *ERK* target *p90RSK* at Ser-9, which inactivates *GSK3β* [112].
	⊣ P	AKT_H	*AKT* blocks *GSK3* kinase activity via an inhibitory phosphorylation on the amino terminus, which blocks the substrate accessibility of *GSK3* [69].
	⊣ P	S6K	*GSK3* is a direct phosphorylation target of *S6K1*, resulting in its inhibition [61].
	⊣ Ind	Hif1a_High	*GSK3* is indirectly repressed by *Hif-1α*-dependent induction of miR-675, which lowers textitGSK-3/β activity by targeting the mRNA of serine/threonine-protein phosphatases *PPP2CA* for degradation. This phosphatase is responsible for *GSK-3β* activation by dephosphorylation [113].
FoxO1	**FoxO1** = not(**Plk1** or **AKT_H**)		
	TF		*FoxO1* is transcriptionally active in the absence of peak *AKT1* activation and *Plk1* activity.
	⊣ PLoc	AKT_H	*AKT1* mediates the translocation of *FoxO1* our of the nucleus through direct phosphorylation of three conserved residues. These events create a recognition site for *14-3-3* family proteins, which export and sequester *FoxO1* in the cytosol [69].
	⊣ PLoc	Plk1	*Plk1* interacts with and phosphorylates *FoxO1*, mainly at the G2/M phase of the cell cycle. *Plk1*-mediated phosphorylation leads to the impairment of *FoxO1*'s transcriptional activity in an *Akt*-independent manner. *Plk1*-induced *FoxO1* phosphorylation causes its nuclear exclusion [114].
p21_mRNA	**p21_mRNA** = ((**FoxO1** and **FoxO3**) or **SMAD2_3_4** or not **Myc** or **Hif1a_High**) and (not(**ZEB1_H** or **b_catenin_TCF4** or **N_bcatenin_H** or **HMGA1**) or (**Hif1a_High** and not **Myc**))		
	mRNA		Our model requires both FoxOs to induce $p21^{Cip1}$ if *Myc* is active and one of the two if *Myc* is OFF or *FoxO* factors are aided by *Smad* activity [115]. This is based on data showing that both *FoxO3* and *FoxO1* bind and induce the $p21^{Cip1}$ promoter and that loss of *Myc* repression alone is not sufficient to induce $p21^{Cip1}$ [116]. Finally, high levels of *ZEB1* repress $p21^{Cip1}$ mRNA expression [117].
	← TR	FoxO3	$p21^{Cip1}$ is a direct transcriptional target of *FoxO3* [116].
	← TR	FoxO1	$p21^{Cip1}$ is a direct transcriptional target of *FoxO1* [116].
	← TR	SMAD2_3_4	*Smad3–Smad4* complexes associate with *FoxO* transcription factors to induce $p21^{CIP1}$ [115].
	⊣ TR	ZEB1_H	*ZEB1* (old name δEF1) is a direct transcriptional repressor of the p21 promoter [117].
	⊣ TR	HMGA1	*HMGA1* maintains cell proliferation through direct inhibition of $p21^{Cip1}$ [118].

Table S1i: GF_connect module

	⊣ TR	N_bcatenin_H	*β-catenin/TCF4* are direct transcriptional repressors of *p21*^{Cip1} [119].
	⊣ TR	b_catenin_TCF4	*β-catenin/TCF4* are direct transcriptional repressors of *p21*^{Cip1} [119].
	← Ind	Hif1a_High	High levels of *Hif-1α* were shown to indirectly increase *p21* mRNA levels through direct repression of *Myc* transcription. Knockouts of *Hif-1α* under hypoxic conditions attenuate increases of *p21* [120].
	⊣ TR	Myc	*Myc* is a direct transcriptional repressor of the *p21*^{Cip1} promoter (it is recruited by the DNA-binding *Miz-1*) [34, 121].
IKKa_b	**IKKa_b = AKT_H** or not **PHD1_2**		
	K		*IKKα* and *IKKβ* are two catalytic subunits of the *IKK* protein complex, composed of *IKKα* and *IKKβ*, and the regulatory protein *NEMO*. Activation of the transcription factor *NF-κB* is mediated by the IKK complex, which phosphorylates and degrades the inhibitory *IκB* proteins [122].
	← P	AKT_H	*IKKα* is phosphorylated by *AKT* at T23, and as subsequent *NF-κB* activation is induced when high *AKT* activity is observed, our model requires *AKT_H = ON* for this to occur [123].
	⊣ -OH	PHD1_2	*IKKβ* levels increase under hypoxic conditions, as prolyl hydroxylases are no longer able to hydroxylate the conserved oxygen-dependent domain found in *IKKβ* [124].
NfkB	**NfkB = IKKa_b** or **PAK1**		
	TF		*NF-κB* is a transcription factor primarily known as a master regulator of inflammatory signaling and the immune system. Its role in cancer is partly due to its ability to aid EMT. It is activated via the destruction of its inhibitory binding partner *IκB*, which is phosphorylated by the *IKK* complex subsequently destroted [122].
	← Loc	PAK1	Active *PAK1* binds to with *NF-κB*-inducing kinase *NIK*, which induces degradation of *IκB* and thus activates *NF-κB* [125].
	← Ind	IKKa_b	*IKKα*, part of the *IKK* complex, phosphorylates and degrades the inhibitory *IκB* proteins [122]; an action that can be independent of the *IKKβ* subunit [126].
c_Myb	**c_Myb = NfkB** or **E2F1**		
	TF		*c-Myb* is a transcription factor that can induce the epithelial micro-RNA *miR-200* [127]. *c-Myb* is induced by *AKT*-mediated activation of *NF-κB* and/or *E2F1* [128].
	← TR	NfkB	*NF-κB* is a direct transcriptional inducer of *c-Myb* [128].
	← TR	E2F1	*E2F1* is a direct transcriptional inducer of *c-Myb* [128].

Table S1j: Adhesion module

Target Node	Node Gate<br>Node Type<br>Link Type	Node Description<br>Input Node	Link Description
Integrin	**Integrin** = **ECM**		
	Rec	Integrins are a superfamily of heterodimeric cell adhesion receptors that bind to extracellular matrix ligands, cell-surface ligands, and soluble ligands. Upon ligand binding, integrins transduce biomechanical information to the cell interior [129].	
	$\leftarrow$ Ligand	ECM	*Integrin* activation and signaling requires *integrin-ECM* attachment [130].
ILK	**ILK** = **Integrin**		
	K	Integrin-linked kinase *ILK* interacts with the cytoplasmic domain of $\beta 1$ integrins, and acrs as a scaffold connecting integrins to the actin cytoskeleton and other signalling pathways [131].	
	$\leftarrow$ BLoc	Integrin	Integrin-mediated adhesion to the ECM recruits and activates *ILK* [131].
FAK	**FAK** = not **Casp3** and not(**Cdk1** and **CyclinB**) and **Integrin**		
	K	*FAK* is activated at integrin-ECM attachment sites in the absence of *Caspase 3*-mediated cleavage and *Cyclin B/Cdk1* activity [132, 133, 134].	
	$\leftarrow$ P	Integrin	*Integrin* activation leads to recruitment and phosphorylation of the Focal Adhesion Kinase (*FAK*) [132], one of its key signaling mediators.
	$\vdash$ Ind	CyclinB	During mitosis, cells detach most of their focal adhesions and round up. This process is *Cdk1/Cyclin B* dependent [135], and it leads to the dissociation of focal adhesion complex components including focal adhesion kinase (*FAK*), *paxillin*, and *CAS*. These proteins all change their phosphorylation status by losing active tyrosine and gaining inhibitory serine/threonine phosphorylation [134].
	$\vdash$ Ind	Cdk1	During mitosis, cells detach most of their focal adhesions and round up. This process is *Cdk1/Cyclin B* dependent [135], and it leads to the dissociation of focal adhesion complex components including focal adhesion kinase (*FAK*), *paxillin*, and *CAS*. These proteins all change their phosphorylation status by losing active tyrosine and gaining inhibitory serine/threonine phosphorylation [134].
	$\vdash$ Lysis	Casp3	Caspase 3 cleaves and deactivates *FAK* during apoptosis by separating its tyrosine kinase from its focal adhesion targeting domain. These fragments further suppress phosphorylation of intact *FAK* [133].
Src	**Src** = (((**Integrin** or (**Nectin3** and **J_Ecadherin**)) and (**RTK** or **FAK**)) or (**Cdk1** and **CyclinB**)) or (**TGF_bRI** and **TGF_bRII**) or (**Hypoxia** and **ROS**)		

Table S1j: Adhesion module

	K		*Src* is activated by *FAK* or *RTKs* at sites of integrin-ECM or cell-cell attachments [136, 137], by *TGFβ* signaling [138], or hypoxia and ROS [120, 139]. In addition, *Cyclin B / Cdk1* phosphorylate *Src* during mitosis [140].
	← Loc	RTK	*RTKs* cooperate with *integrins* to recruit and activate *Src* kinases, which in turn help potentiate *RTK* signaling. Thus, in our model *Src* may be activated by basal *RTK* activity, and is, in turn, required for peak *RTK* activation [137].
	← PLoc	Integrin	*FAK* phosphorylation at Y397 at sites of *integrin-ECM* adhesion creates a high-affinity binding site for *Src*, which leads to the assembly of a *FAK–Src* signaling complex [136].
	← PLoc	FAK	*FAK* phosphorylation at Y397 at sites of *integrin-ECM* adhesion creates a high-affinity binding site for *Src*, which leads to the assembly of a *FAK–Src* signaling complex [136].
	← PLoc	Nectin3	*Nectin* was found to locally recruit and activate *Src*, leading to downstream phosphorylation of targets *Cdc42* and *Rac1*; downstream signaling was unable to be induced without *Nectins* [141].
	← PLoc	J _Ecadherin	*E_ cadherin* was found to upregulate *Src* signaling through localized phosphotryosine signaling in response to homophilic ligation; *Src* activity was also found to be increased around cell-cell contact sites [142].
	← BLoc	TGF_bRII	*TGFβ* activates *Src* via active *TβRI / TβRII* complexes [138].
	← BLoc	TGF_bRI	*TGFβ* activates *Src* via active *TβRI / TβRII* complexes [138].
	← Ind	Hypoxia	Hypoxia induces high levels of *Src* activation, required for *Src*-induced EMT and migration [120].
	← Ind	ROS	Increased activation of *Src* under hypoxia is dependent on ROS. Overexpression of ROS scavengers was shown to abate *Src* activation and inhibit *β-catenin-Hif-1α* complex formation [139].
	← P	CyclinB	Mitotic *Cyclin B/Cdk1* complexes phosphorylate and activate *c-Src* during mitosis [140].
	← P	Cdk1	Mitotic *Cyclin B/Cdk1* complexes phosphorylate and activate *c-Src* during mitosis [140].
Src_High	**Src_High** = **Src** and **Hypoxia** and **ROS**		
	K		This node represents high levels of *Src* signaling under hypoxic conditions [120].
	← Per	Src	*Src_High* = ON requires moderate activaiton of *Src*, further boosted by hypoxia and ROS.
	← Ind	Hypoxia	Hypoxia induces high levels of *Src* activation, required for *Src*-induced EMT and migration [120].

Table S1j: Adhesion module

	← Ind	ROS	Increased activation of *Src* under hypoxia is dependent on ROS. Overexpression of ROS scavengers was shown to abate *Src* activation and inhibit *β-catenin-Hif-1α* complex formation [139].
Nectin3	**Nectin3** = **CellDensity_Low** or **CellDensity_High**		
	CAM		*Nectins* form weak adhesions between adjacent cells by binding to *Nectins* on other cells and promoting local membrane ruffling that is required for adherens junction formation [143]. As downstream effects of *Nectin3* - *Nectin* binding between two cells do not require tight junction formation and high cell density, *Nectin3* activation in our model only requires the presence of some neighbors.
	← Env	CellDensity_High	Nectins avtivate by forming weak adhesions between adjacent cells [143].
	← Env	CellDensity_Low	Nectins avtivate by forming weak adhesions between adjacent cells [143].
Necl5	**Necl5** = **FocalAdhesions** or not(**Nectin3** and **CellDensity_High**)		
	Prot		*Necl-5* activity is controlled by co-localization with focal adhesions where it binds *Spry2* and aids receptor tyrosine kinase signaling [144]. We modeled this by turning the *Necl-5* node ON when the *Focal Adhesions* node is ON (indicating strong attachments that pull on the ECM and can form stress fibers), or in the absence of cell-cell adhesions at all sites of cell ECM-adhesion (i.e, fully surrounded with no free edge).
	⊢ Env	CellDensity_High	At high cell density, adherens junctions that surround the cell suppress integrin-mediated activation and recruitment of *Necl-5* to the cell surface across the entire cell, which releases its block on *Spry2* [144].
	⊢ Unbind	Nectin3	*Necl-5* interacts with *Nectin3* on neighboring cells (*Nectin 3* in our model is a proxy for this, as it is activated by cell-cell contacts), which promotes downstream reorganization of the cytoskeleton to aid adherens junction formation, which, in turn releases *Necl-5* from these adhesions [144].
	← Loc	FocalAdhesions	*Necl-5* is recruited to focal adhesions at the leading edge of cells by direct interactions with integrins [145]. This localization is important for its downstream effects.
SPRY2	**SPRY2** = not **Necl5** and **RTK** and **Src**		
	Prot		*SPRY2* (*Sprouty 2*) is a negative regulator of growth factor-induced signaling, especially *Ras/MAPK* [146].
	← Loc	RTK	*Sprouty* proteins are activated by ligand-bound RTKs to modulate / inhibit downstream MAPK signaling [147].
	← P	Src	Growth factor-induced tyrosine phosphorylation of *Spry2* is mediated by a *Src*-like kinase [147].
	⊢ IBind	Necl5	*Necl-5* localized to integrin clusters binds to and blocks the activity of *SPRY2* [146].

Table S1j: Adhesion module

J _Ecadherin	**J_Ecadherin** = (**Ecadherin_mRNA_H** or **Ecadherin_mRNA**) and **Nectin3** and not **Casp3**		
	CAM	*E-cadherin* proteins are adhesion molecules required for adherens junction formation. The presence of juncitonal *E-cadherin* (J_Ecadherin = ON) in our model requires *E-cadherin* mRNA expression, *Nectin3*-mediated sensing of contact with neighboring cells (binding to nectins) [144] and inactive Caspase 3 [148].	
	← BLoc	Nectin3	Cadherins are recruited to cell-cell adhesions formed by *Nectin3-nectin* interactions between neighboring cells, where they bind to cadherins on adjacent cells to form AJs [144].
	← TL	Ecadherin _mRNA_H	*E-cadherin* mRNA expression is required for maintenance of *E-cadherin* protein.
	← TL	Ecadherin _mRNA	*E-cadherin* mRNA expression is required for maintenance of *E-cadherin* protein.
	⊢ Lysis	Casp3	Caspase 3 cleaves junctional E-cadherin, dissociating it from the cell surface and blocking its ability to form adherens junctions [148].
J_bcatenin	**J_bcatenin** = **J_Ecadherin** and not **Casp3**		
	Prot	Junctional *E-cadherin* proteins binds to and recruits/sequesters β-*catenin* to adherens junctions. This negatively regulates β-catenin mediated transcription, but aids adherens junction formation [149]. Here we assume that β-*catenin* is localized to cell-cell junctions (*J_bcatenin* = ON) as long as there are *E-cadherin*-mediated attachments to neighboring cells (even if a cell is not fully surrounded) and *Caspase 3* is inactive [150].	
	← Per	J _Ecadherin	Junctional *E-cadherins* recruits/sequester β-*catenin* to adherens junctions [149].
	⊢ Lysis	Casp3	Active *Caspase 3* cleaves β-*catenin* into several fragments that lose their transcriptional activity and become localized to the cytoplasm [150].
J_acatenin	**J_acatenin** = **J_Ecadherin** and **J_bcatenin**		
	Adap	Junctional β-*catenin* binds to and recruitcs α-*catenin* to adherens junctions. α-*catenin* links β-*catenin* to the actin cytoskeleton to stabilize adherens junctions [149].	
	← Compl	J _Ecadherin	Junctional *E-cadherin*-bound β-*catenin* binds to and recruitcs α-*catenin* to adherens junctions [149].
	← Compl	J_bcatenin	Junctional β-*catenin* binds to and recruitcs α-*catenin* to adherens junctions [149].

Table S1k: CIP module

Target Node	Node Gate

Table S1k: CIP module

Node Type / Link Type	Node Description / Input Node	Link Description
FocalAdhesions	**FocalAdhesions** = **Integrin** and **FAK** and **ECM** and (**Stiff_ECM** or (**YAP** and (**Rac1** or **Rac1_H**) and **IQGAP1_LeadingE**))	
MSt	*Focal adhesions* form at sites of *Integrin-ECM* attachment and clustering [151]. In order to take into account the effect of stiff ECM [152] as well as positive feedback between focal adhesion formation and horizontal cell polarization that creates an active leading edge, here we assume that force-generating Focal Adhesion formation requires ECM-Integrin attachments and *FAK* [153], and either strong traction force generation supported by a stiff ECM, or the existence of a leading edge with active *Rac1* [154] and *IQGAP1* [155, 156, 157] supported by *YAP*-mediated upregulation of adhesion and focal adhesion-associated proteins [158].	
← Loc	Stiff_ECM	Adhesion to stiff ECM engages the *FAK/phosphopaxillin/vinculin* pathway, which generate a fluctuating "tugging" action on the ECM and probe ECM rigidity to aid *focal adhesion* formation and migration towards regions of stiffer ECM (durotaxis) [152].
← Loc	ECM	*Focal adhesions* form at sites of *Integrin-ECM* attachment and clustering [151].
← Loc	Integrin	*Focal adhesions* form at sites of *Integrin-ECM* attachment and clustering [151].
← Loc	FAK	*FAK* activation at sites of cell-ECM attachments (driven by force generation) can increase paxillin phosphorylation and strengthen cytoskeletal linkage and vinculin recruitment to such adhesions, resulting in *focal adhesion* maturation [153].
← TR	YAP	*YAP* is a transcriptional inducer of *f integrins* and *FA* docking proteins, and promotes *focal adhesion* formation by increasing cell spreading and *RhoA GTPase* activity [158].
← Compl	IQGAP1 _LeadingE	In migrating cells *IQGAP1* localizes to lamellipodia at the leading edge, recruited by active *RTKs* synergistically activated here by *integrin-RTK* crosstalk. Here, *IQGAP1* supports *Rac1* activation and *focal adhesion* formation; it required for migration in response to growth signals such as *PDGF*, *VEGF*, ect [155, 156, 157].
← Loc	Rac1	Active *Rac1* promotes the association of nonmuscle myosin II (MIIA) with *focal adhesions* at the leading edge during cell migration, aiding the assembly of mini- filaments in *focal adhesions*. These promote further assembly of *focal adhesions* and modulation of the traction forces cells exert on the ECM [154].
← Loc	Rac1_H	Active *Rac1* promotes the association of nonmuscle myosin II (MIIA) with *focal adhesions* at the leading edge during cell migration, aiding the assembly of mini- filaments in *focal adhesions*. These promote further assembly of *focal adhesions* and modulation of the traction forces cells exert on the ECM [154].

Table S1k: CIP module

Stress_Fibers	**Stress_Fibers** = not **CellDensity_High** and **Stiff_ECM** and **FocalAdhesions**		
	MSt	In order to model the independent effects of cell density and matrix stiffness on stress fiber formation, we assume that they require both the absence of high cell density, and the presence of focal adhesions attached to a stiff ECM.	
	⊣ Ind	CellDensity_High	*High cell density* blocks stress fiber formation by forbidding cells access to a large enough area to spread and exert force on the *ECM* [159].
	← Ind	Stiff_ECM	In the absence of a sufficiently stiff *ECM*, *Focal Adhesions* are small and stress fibers are less abundant or fail to form, as cells cannot generate sufficient traction [160].
	← ComplProc	FocalAdhesions	*Stress Fibers* are anchored to the *ECM* via strong, stable *Focal Adhesions* [160].
YAP	**YAP** = **FocalAdhesions** and **Stress_Fibers** and not(**ApicalBasal_Pol** and **J_acatenin** and **AMOT** and **Merlin** and **Lats1_2**)		
	TF	YAP is a mechanosensitive transcriptional regulator of proliferation and migration, and its activation is controlled by both the cell's ability to spread on an ECM (*FocalAdhesions* and *Stress_Fibers*), and the lack of apical-basal polarity with mature adherens junctions that can sequester *YAP* in the cytoplasm by binding and inhibitory phosphorylation. Experimental evidence indicates that in cells that maintain apical-basal polarity, several junctional proteins (*α-catenin*, *AMOT*, *Merlin*) and inhibitory kinases (*Lats1* and *Lats2* work together to sequester and block *YAP* [161, 162].	
	⊣ Per	J_acatenin	Junctional *alpha-catenin* binds *YAP* and sequesters it in the cytoplasm [163] [164] [165]. This also concentrates *YAP* close proximity to junction-localized Hippo pathway components such as *Lats1/2*, *Merlin* and *Amot.*
	← Ind	FocalAdhesions	*YAP* activation is abolished by the absence of *stress fibers* anchored to *focal adhesions*, even in the absence of inhibitory Hippo signaling [159, 166].
	← Ind	Stress_Fibers	*YAP* activation is abolished by the absence of *stress fibers* anchored to *focal adhesions*, even in the absence of inhibitory Hippo signaling [159, 166].
	⊣ Ind	ApicalBasal_Pol	Full inhibition of *YAP* by Hippo signaling linked to adherens and tight junction formation requires the establishment of *apical-basal polarity*, as cells at the edges of monolayers or in decreased cell density areas have active (nuclear) *YAP* in spite of strong remaining attachments to neighboring cells [166, 167].
	⊣ IBind	Lats1_2	The *Lats1* and *Lats2* tumor suppressor kinases bind to and phosphorylate *YAP* in vitro and in vivo [168, 162].

Table S1k: CIP module

	$\dashv$ Compl	AMOT	*AMOT* localizes to tight junctions, where it suppresses *YAP* activity by direct binding and recruitment of *YAP* inhibitory kinase *LATS2* [161]. By binding both and *YAP/TAZ*, *AMOT* works as a scaffold that connects *LATS1/2* to both its activator *MST1* and its target *YAP/TAZ* [169].
	$\dashv$ BLoc	Merlin	*Merlin* localizes to adherens junctions where it activates the *Hippo pathway* by binding to and recruiting *LATS1/2* kinases and *YAP/TAZ* to *adherens junctions* [170]. In the absence of *Merlin*, Hippo pathway components fail to block *YAP* activity [171]. *Merlin* - *YAP* binding requires active (phosphorylated) *AMOT* [172].
TRIO	**TRIO** = **YAP**		
	GEF	*TRIO* is a *Rac1*-activating GTP-exchange factor induced by *YAP* [167, 173].	
	$\leftarrow$ TR	YAP	*YAP* is a transcriptional inducer of *TRIO* [173].
WT1	**WT1** = **YAP**		
	TF	The Wilms Tumor 1 (*WT1*) transcription factor is a repressor of *E-cadherin* expression [167]. Its nuclear localization is controlled by *YAP* binding [173].	
	$\leftarrow$ BLoc	YAP	*YAP* binds to and controls nuclear localization of the Wilms Tumor 1 (*WT1*) transcription factor [173].
TAZ	**TAZ** = **Stress_Fibers** and not(**ApicalBasal_Pol** and **J_acatenin** and **AMOT** and **Merlin** and **Lats1_2**)		
	TF	*TAZ* is a mechanosensitive transcriptional regulator of cell migration, and its activation is controlled by both the cell's ability to spread on stiff ECM (*Stress_Fibers* = ON) and the lack of apical-basal polarity with mature adherens junctions that can sequestered *TAZ* in the cytoplasm by binding and inhibitory phosphorylation [166, 167]. In cells that maintain apical-basal polarity, junctional proteins (α*-catenin*, *AMOT*, *Merlin*) and inhibitory kinases (*Lats1* and *Lats2*) work together to sequester and block *TAZ* [163, 164, 165, 174, 162].	
	$\dashv$ BLoc	J_acatenin	Junctional *alpha-catenin* binds *YAP/TAZ* and sequesters them in the cytoplasm [163, 164, 165, 174]. This also concentrates *YAP/TAZ* close proximity to junction-localized Hippo pathway components such as *Lats1/2*, *Merlin* and *Amot*.
	$\leftarrow$ Ind	Stress _Fibers	*TAZ* activation is abolished by the absence of *stress fibers* anchored to *focal adhesions*, even in the absence of inhibitory Hippo signaling [159, 166].
	$\dashv$ Ind	ApicalBasal _Pol	Full inhibition of *TAZ* by Hippo signaling linked to adherens and tight junction formation requires the establishment of apical-basal polarity, as cells at the edges of monolayers or in decreased cell density areas have active (nuclear) *TAZ* in spite of strong remaining attachments to neighboring cells [166, 167].
	$\dashv$ P	Lats1_2	The *Lats1* and *Lats2* tumor suppressor kinases bind to and phosphorylate *YAP/TAZ* in vitro and in vivo [168, 162, 174].

Table S1k: CIP module

	⊢ IBind	AMOT	*AMOT* localizes to tight junctions, where it suppresses *YAP/TAZ* activity by direct binding and recruitment of *YAP* inhibitory kinase *LATS2* [161, 174]. By binding both and *YAP/TAZ*, *AMOT* works as a scaffold that connects *LATS1/2* to both its activator *MST1* and its target *YAP/TAZ* [169].
	⊢ IBind	Merlin	*Merlin* localizes to adherens junctions where it activates the Hippo pathway by binding to and recruiting *LATS1/2* kinases and *YAP/TAZ* to adherens junctions [170]. In the absence of *Merlin*, Hippo pathway components fail to block *YAP/TAZ* activity [171, 174].
Ecadherin _mRNA_H	**Ecadherin_mRNA_H** = **Ecadherin_mRNA** and not(**YAP** and **WT1**)		
	mRNA		Experiments show that *YAP* and *WT1* suppress but do not abolish *E-cadherin* protein expression in areas of lowered cell density [167]. To model this, we introduced a *Ecadherin_mRNA_H* node that is blocked by *YAP/WT1* repressor complexes (requiring their joint nuclear localization). *Ecadherin_mRNA_H*, in turn, must be ON to allow cells to establish a ring of adherens junctions sufficient for apical-basal polarity.
	⊢ Compl	YAP	Active *YAP* binds *WT1* and localizes it to the nucleus, where they form a complex a the *E-cadherin* promoter and reduce its transcription [167].
	⊢ Compl	WT1	Active *YAP* binds *WT1* and localizes it to the nucleus, where they form a complex a the *E-cadherin* promoter and reduce its transcription [167].
	← Per	Ecadherin _mRNA	High *E-cadherin* mRNA expression requires basal levels of *E-cadherin*. Our previously published epithelial model assumed this to be true [109], whereas in the current model this is only the case when *E-cadherin* expression is not fully inhibited by EMT-promoting repressors (modeled as acting on the *Ecadherin_mRNA* node).
ApicalBasal _Pol	**ApicalBasal_Pol** = **ECM** and **CellDensity_High** and **Nectin3** and **J_Ecadherin** and **J_bcatenin** and **J_acatenin** and (**Ecadherin_mRNA_H** or not **Horizontal_Pol**)		
	MSt		In addition to a need for high cell density and cell-cell adhesion proteins that help assemble adherens junctions (*Nectin3*, *J_Ecadherin*, *J_bcatenin*, *J_acatenin*), we assumed that either high (unimpeded) *E-cadherin* mRNA expression or lack of a horizontally polarized cell morphology are required for the establishment of apical-basal polarity.
	← Ind	CellDensity _High	Establishment of *apical-basal polarity* requires a ring of adherens and tight junctions that can only form in high cell density [175].
	← ComplProc	ECM	Establishment of *apical-basal polarity* requires an underlying surface such as *ECM* to define a basal side.
	← Ind	Nectin3	A key driver of *apical-basal polarization*, *Par-3*, is recruited to newly formed cell-cell adhesions by *Nectin-3* binding [175].

Table S1k: CIP module

	$\leftarrow$ Ind	J _Ecadherin	Formation of adherens junctions is a prerequisite for tight junction assembly, which is, in turn, required for *apical-basal polarity* [175].
	$\leftarrow$ Ind	J_bcatenin	Formation of adherens junctions is a prerequisite for tight junction assembly, which is, in turn, required for *apical-basal polarity* [175].
	$\leftarrow$ Ind	J_acatenin	Formation of adherens junctions is a prerequisite for tight junction assembly, which is, in turn, required for *apical-basal polarity* [175].
	$\leftarrow$ Ind	Ecadherin _mRNA_H	Formation of adherens junctions in high concentration around the cell is required for *apical-basal polarity* [175], and thus aided by high *E-cadherin* protein expression.
	$\vdash$ Ind	Horizontal _Pol	Horizontal polarization and apical-basal (vertical) polarization are mutually exclusive; cells must first lose the asymmetry between their leading and trailing edge in order to establish a ring of adherens and tight junctions.
N_bcatenin	**N_bcatenin** = not **Casp3** and (**Hif1a_High** or not(**ApicalBasal_Pol** and **GSK3**))		
	TF	When released from cell-cell junctions, *β-catenin* localizes to the nucleus and induces genes that promote proliferation and EMT [176].	
	$\vdash$ Deg	GSK3	The cytoplasmic pool of *β-catenin* not tied to junctions is highly unstable due to multiple phosphorylations promoting its proteasome-mediated degradation. This phosphorylations is maintained by the "*β-catenin* Destruction Complex" composed of the tumor suppressor *APC*, the scaffolding protein *Axin*, and the serine/threonine kinases *GSK3β* and *CK1* (casein kinase 1, which primes *GSK3*). When *GSK3* is inhibited, unphosphorylated *β-catenin* accumulates, translocates to the nucleus, and promotes transcription [176].
	$\vdash$ Loc	ApicalBasal _Pol	Junctional *E-cadherins* recruits/sequesters *β-catenin* to adherens junctions and block *β-catenin* nuclear localization [149]. Here we assume that cells able to form a ring of adherens junctions and establish apical-basal polarity lack nuclear *β-catenin*.
	$\leftarrow$ Loc	Hif1a_High	Overexpression/induction of *Hif-1α* expression has been demonstrated to increase levels of nuclear translocation of *β-catenin*, increasing mesenchymal phenotypes [177].
	$\vdash$ Lysis	Casp3	Active Caspase 3 cleaves *β-catenin* into several fragments that lose their transcriptional activity and become localized to the cytoplasm [150].
Mst1_2	**Mst1_2** = **ApicalBasal_Pol**		
	K	In cells that establish apical-basal cell polarity, *Mst1* and *2* activate the Hippo pathway by phosphorylating *Lats1/2* kinases to promote *YAP/TAZ* inhibition [178, 179, 180].	
	$\leftarrow$ Ind	ApicalBasal _Pol	Activation of the Hippo pathway by *Mst1/2* requires apical-basal cell polarity [180].

Table S1k: CIP module

Lats1_2 **Lats1_2** = **Mst1_2** and **Merlin**

K — *Mst1/2* activate *Lats1/2* kinases by phosphorylation, aided by their mutual binding to *Merlin*.

← P	Mst1_2	*Mst1/2* phosphorylate and activate *Lats 1 / 2* kinases to promote *YAP/TAZ* inhibition [178, 179].
← BLoc	Merlin	*Merlin* binds to *Lats1/2* and recruits it to the plasma membrane near adherens and tight junctions, where *Mst1/2* can phosphorylate it [170].

AMOT **AMOT** = **Lats1_2** and **Merlin**

Adap — In our model, *AMOT* = ON represents phosphorylated and tight junction localized *AMOT*, which requires *Merlin* binding and *Lats1/2* mediated phosphorylation [181, 182].

← P	Lats1_2	*Lats1/2* kinases phosphorylate the N-terminal regions of *Amot*, disrupting its interaction with F-actin [183]. As tight junction localized *AMOT* aids Hippo signaling whereas F-actin bound *AMOT* hinders it [181], *AMOT* = ON in our model represents the phosphorylated, TJ-bound protein.
← BLoc	Merlin	*Merlin* binds to *AMOT* proteins and sequesters them to tight junctions [183], shifting its activity from cytoplasmic (where it binds *F-actin* and blocks the *Rac1* inhibitor *Rish1*) to junctional (where it forms a scaffold for Hippo signaling) [182].

miR_29c **miR_29c** = **YAP**

miR — *YAP* induces the expression of microRNA *miR-29c* to target *PTEN* for degradation [184].

← TR	YAP	*YAP* is a direct inducer of *miR-29c* [184].

PTEN_c **PTEN_c** = not **miR_29c** and ((**S6K** and not(**ERK** and **GSK3**)) or not(**ERK** or **GSK3**))

Ph — Cellular *PTEN* is a tumor supressor phosphatase that reverses PIP2 → PIP3 conversion carried out by *PI3K*, and thus supresses *PI3K / AKT* signaling [185]. The combinatorial effect of the distinct *PTEN* regulators included below is not well documented. We chose to model cytoplasmic *PTEN* availability as ON in the absence of *miR-29c* [184]. In addition, we combined the positive and negative effects of *S6K*, *ERK* and *GSK3* by assuming that *PTEN* is blocked by the joint action of *ERK* and *GSK3* in the presence of *S6K*, and by either inhibitor in its absence.

⊣ TR	ERK	*ERK* activation suppresses *PTEN mRNA* and protein expression [186, 187].
← P	S6K	*S6K* phosphorylates *PTEN*, which leads to *PTEN* deubiquitination and export from the nucleus to the cytoplasm [188].
⊣ P	GSK3	*GSK3* phosphorylates *PTEN* on Thr366 which leads to destabilization of the *PTEN* protein [189].

Table S1k: CIP module

	$\dashv$ Ind	miR_29c	*YAP* induces transcription of *miR-29*, which in turn binds to *PTEN mRNA* to block its translation [184].

Table S1l: Migration_SW module

Target Node	Node Gate<br>Node Type<br>Link Type	Node Description<br>Input Node	Link Description
Merlin	**Merlin** = **J_bcatenin** and **J_acatenin** and not(**PAK1** and **ILK**) and not **AKT_H**		
	Prot	*Merlin* is functionally localized and able to mediate Hippo signaling when recruited by junctional β- and α-*catenin*, and not phosphorylated by high *AKT1* or the cooperative action of *PAK1* and *ILK*. Since the *AKT_B* node in our model represents basal *AKT* activity which co-occurs with *Merlin*-mediated contact inhibition, we choose the peak *AKT_H* node to mark the level of *AKT* signaling required to block *Merlin.*	
	$\dashv$ P	AKT_H	*Akt* directly binds to and phosphorylates *Merlin* at Thr230 and Ser315, blocking its ability to bind its regular partners and promoting its degradation [190].
	$\dashv$ Ind	ILK	*ILK* suppresses the Hippo pathway via phosphorilation and inhibition of *MYPT1-PP1*, leading to inactivation of *Merlin* [191].
	$\leftarrow$ Loc	J_bcatenin	*Merlin* binds to β-*catenin* at adherens junctions, and disruption of β-*catenin* expression recoiples cell density sensing from Hippo signaling downstream *Merlin* [192].
	$\leftarrow$ BLoc	J_acatenin	*Merlin* binds to α-*catenin* to localize to adherens junctions, where it plays a role in their maturation, links AJ formation and the *Par3* polarity complex, and orchestrates Hippo signaling [192, 193].
	$\dashv$ P	PAK1	*Pak1* can directly phosphorylate *Merlin* at Ser518 [194]. This phosphorylation prevents it from binding to /textitAMOT and *Lats1/2* [195], and thus carrying out its contact inhibitory function.
IQGAP1 _LeadingE	**IQGAP1_LeadingE** = not **CellDensity_High** and **FocalAdhesions** and (**Grb2** or (**Horizontal_Pol** and (**Rac1** or **Rac1_H**)))		
	Adap	In our model, *IQGAP1_LeadingE* = ON represents *IQGAP1* localized near the leading edge of a horizontally polarized cell, where it links focal adhesion-mediated and *RTK*-mediated signaling [156]. Its recruitment is sustained by well-established horizontal polarity, or induced by *Rac1* [196] or *RTK-Grb2* binding [51, 197, 198].	
	$\dashv$ Ind	CellDensity _High	*IQGAP1* localization to the leading edge is blocked by high cell density that leaves no free edge.

Table S1l: Migration_SW module

	← BLoc	Grb2	*Grb2* bound to active *RTK* binds and recruits *IQGAP1*, aiding its enrichment at the leading edge where the concentration of active *RTKs* is generally higher [51, 197, 198].
	← ComplProc	FocalAdhesions	*IQGAP1* interacts with focal adhesion proteins and tyrosine kinase receptors to link focal adhesion and *RTK* signaling. It is thus enriched at the leading edge by increased active FA formation [156].
	← ComplProc	Horizontal _Pol	*IQGAP1* localization to the leading edge is reinforced by horizontal polarization and the stabilization of a leading edge [156, 199].
	← Compl	Rac1	Active *Rac1* forms a complex with *IQGAP1* and *CLIP-170* and recruits both to the base of the leading edge, where their aid cytoskeletal reorganization, horizontal polarization and directed migration [196].
	← Compl	Rac1_H	Active *Rac1* forms a complex with *IQGAP1* and *CLIP-170* and recruits both to the base of the leading edge, where their aid cytoskeletal reorganization, horizontal polarization and directed migration [196].
Horizontal _Pol	**Horizontal_Pol** = not **ApicalBasal_Pol** and **ECM** and **IQGAP1_LeadingE** and **FocalAdhesions** and **TAZ** and **FAK**		
	MSt		For cells to establish and maintain horizontal polarization, our model requires the lack of apical-basal polarization, the presence of an ECM, as well as asymmetric IQGAP1 localization [200], focal adhesions and spreading (FAK [200, 201] and TAZ [158]).
	← Ind	ECM	Horizontal polarization requires a leading edge with lamellipodia and a trailing edge linked to stress fibers; both of which require adhesions to an ECM.
	← Ind	FAK	*FAK* activation at nascent adhesions at the leading edge is required for ongoing cell spreading, which is a prerequisite of ongoing *FA* maturation and maintenance of an active leading edge [200, 201].
	← Loc	FocalAdhesions	Horizontal polarization requires a leading edge with lamellipodia and a trailing edge linked to stress fibers, both of which depend on *Focal Adhesions* linked to the actin cytoskeleton.
	← Ind	TAZ	*TAZ*-null cells lose their ability to spread on the ECM, indicating that *TAZ* transcriptional activity is required for the establishment of horizontal polarization [158]. (This is in contrast to *YAP*-null cells, which cannot even form *focal adhesions*.)
	⊢ Ind	ApicalBasal _Pol	Apical-basal and horizontal polarization are mutually exclusive; cells must first lose their textitapical-basal polarity before they are able to establish horizontal polarization.

Table S1l: Migration_SW module

	← Per	IQGAP1 _LeadingE	Localization of *IQGAP1* to the leading edge is required for the establishment of horizontal polarization. Together with the adenomatous polyposis coli (*APC*) protein that is also recruited to the leading edge by active *Rac1* and *Cdc42*, *IQGAP1* links the actin cytoskeleton to microtubule dynamics to establish cell polarization [200].
Rac1	**Rac1** = not **Casp3** and **FocalAdhesions** and **Necl5** and **Horizontal_Pol** and **TRIO** and **AKT_B** and (**Stiff_ECM** or not(**Merlin** and **Nectin3** and **J_Ecadherin**))		
	GTPa		Full *Rac1* activation in our model requires the absence of *caspase 3*, as well as horizontal polarization on a stiff ECM, Focal Adhesion formation, *Necl-5* leading edge localization, *TRIO* expression and at least basal *AKT* activity. In addition, on soft ECM *Rac1* activity is inhibited by the presence of *miR-200* [202] and *miR-34* [203], as well as the cooperative action of *Merlin*, *Nectin3* and *E-cadherin* at adherens and tight junctions [204, 182].
	← Per	Stiff_ECM	*Rac1* activated at *focal adhesions* by *FAK* is enhanced by force generation supported by stiff ECM, leading to increased intracellular stiffness [205].
	← Ind	AKT_B	*AKT* phosphorylates the *Rac1* GEF *Tiam1* to facilitate its binding to 14-3-3, which increases its stability. This, in turn, boosts Rac1-GTP levels and *Rac1* activity [206]. Here we assume that at least basal *AKT* activation is required to maintain *Rac1* activity.
	⊢ Compl	Nectin3	During initial cell-cell contact and adherens junciton initiation, cadherins and nectins cooperate to briefly induce, but then rapidly suppress Rac1 [204].
	← Loc	Necl5	*Necl-5* associates with integrins at the leading edge, where it promotes the activation of *Rac1* and *Cdc42*. *Necl-5* is required for serum-and *PDGF*-induced cell motility cell motility, an effect that does not require Nectin-3 binding on neighboring cells [207].
	⊢ Compl	J _Ecadherin	During initial cell-cell contact and adherens junciton initiation, cadherins and nectins cooperate to briefly induce, but then rapidly suppress Rac1 [204].
	← Per	FocalAdhesions	*Rac1* activated at *focal adhesions* by *FAK* is enhanced by force generation supported by stiff ECM, leading to increased intracellular stiffness [205].
	← GEF	TRIO	*TRIO* is a GEF that controls *Rac1* activation during migration [167] as well as proliferation [208].
	⊢ Ind	Merlin	A protein complex that includes *Merlin* sequesters *Angiomotin* to tight junctions, releasing it from binding the *Rac1*-inhibitor *Rich1* [182].
	← Per	Horizontal _Pol	Growth of the microtubule network at leading-edge lamellipodia activates *Rac1* to drive local actin polymerization and further lamellipodial protrusions, thus supporting the maintenance of horizontal polarization [209].

Table S1l: Migration_SW module

	⊣ Lysis	Casp3	Caspase 3 cleaves *Rac1* at two sites, resulting in the inactivation of its GTPase activity and *PAK1* binding [210].
Rac1_H	**Rac1_H** = **Rac1** and not **Casp3** and **FocalAdhesions** and **Necl5** and **Horizontal_Pol** and **TRIO** and not (**miR_200_c** or **miR_200_b** or **miR_34**) and not (**Merlin** and **Nectin3** and **J_Ecadherin**) and **Stiff_ECM** and **AKT_H**		
	GTPa		Full *Rac1* activation in our model requires the absence of *caspase 3*, as well as horizontal polarization on a stiff ECM, Focal Adhesion formation, *Necl-5* leading edge localization and *TRIO* expression. In addition, high *Rac1* activity is inhibited by the presence of *miR-200b/c* [202], *miR-34* [203], as well as the cooperative action of *Merlin*, *Nectin3* and *E-cadherin* at adherens and tight junctions [204, 182]. Finally, we assume that high *Rac1* activity requires a stiff ECM [205] and peak *AKT* signaling.
	← ComplProc	Stiff_ECM	*Rac1* activated at *focal adhesions* by *FAK* is enhanced by force generation supported by stiff ECM, leading to increased intracellular stiffness [205].
	← Ind	AKT_H	*AKT* phosphorylates the *Rac1* GEF *Tiam1* to facilitate its binding to 14-3-3, which increases its stability. This, in turn, boosts Rac1-GTP levels and *Rac1* activity [206]. Here we assume that full *AKT* activation is required to maintain high levels of *Rac1* activity.
	⊣ Compl	Nectin3	During initial cell-cell contact and adherens junciton initiation, cadherins and nectins cooperate to briefly induce, but then rapidly suppress Rac1 [204].
	← Loc	Necl5	*Necl-5* associates with integrins at the leading edge, where it promotes the activation of *Rac1* and *Cdc42*. *Necl-5* is required for serum-and *PDGF*-induced cell motility cell motility, an effect that does not require Nectin-3 binding on neighboring cells [207].
	⊣ Compl	J_Ecadherin	During initial cell-cell contact and adherens junciton initiation, cadherins and nectins cooperate to briefly induce, but then rapidly suppress Rac1 [204].
	← Per	FocalAdhesions	*Rac1* activated at *focal adhesions* by *FAK* is enhanced by force generation supported by stiff ECM, leading to increased intracellular stiffness [205].
	← GEF	TRIO	*TRIO* is a GEF that controls *Rac1* activation during migration [167] as well as proliferation [208].
	⊣ Ind	Merlin	A protein complex that includes *Merlin* sequesters *Angiomotin* to tight junctions, releasing it from binding the *Rac1*-inhibitor *Rich1* [182].
	← Per	Horizontal_Pol	Growth of the microtubule network at leading-edge lamellipodia activates *Rac1* to drive local actin polymerization and further lamellipodial protrusions, thus supporting the maintenance of horizontal polarization [209].
	← Per	Rac1	*Rac1_High* = ON requires moderate activaiton of *Rac1*.

Table S1l: Migration_SW module

	⊣ Ind	miR_34	Though *miR-34* does not appear to directly target *Rac1* mRNA [211], its overexpression blocks GTP-bound (active) *Rac1* [203].
	⊣ RNAi	miR_200_b	*miR-200b/c-3p* represses *Rac1* mRNA by targeting its 3' UTR [202].
	⊣ RNAi	miR_200_c	*miR-200b/c-3p* represses *Rac1* mRNA by targeting its 3' UTR [202].
	⊣ Lysis	Casp3	Caspase 3 cleaves *Rac1* at two sites, resulting in the inactivation of its GTPase activity and *PAK1* binding [210].
PAK1	**PAK1** = **Rac1** or **Rac1_H**		
	K		The *p21*-Activated kinase 1 *PAK1*, a serine-threonine kinase interacts with the Rho GTPases *RAC1* and *CDC42* to drive migration, survival, cell cycle, EMT, stress response and inflammation [212]. It is activated by *Rac1*, which binds to *PAK1* and stimulates its kinase activity [213].
	← Compl	Rac1	*Rac1* binds to *PAK1* and stimulates its kinase activity [213].
	← Compl	Rac1_H	*Rac1* binds to *PAK1* and stimulates its kinase activity [213].
Migration	**Migration** = **Horizontal_Pol** and **Stress_Fibers** and **FocalAdhesions** and **PAK1** and **Rac1**		
	MSt		In the current model, the *Migration* node represents migration by a polarized cell, whereas *Fast_Migration* tracks rapid mesenchymal migration typical of a cell that underwent EMT. *Migration* requires horizontal polarization, stress fiber maintenance, focal adhesion formation and the activity of *Rac1* and *PAK1* kinases [214, 215].
	← Ind	FocalAdhesions	Mesenchymal style migration requires force generation via stress fibers anchored by *focal adhesions* [214].
	← Ind	Stress_Fibers	Mesenchymal style migration requires force generation via stress fibers anchored by *focal adhesions* [214].
	← Ind	Horizontal_Pol	Directed mesenchymal style migration requires *horizontal cell polarization* with a well defined leading and trailing edge [214].
	← ComplProc	Rac1	*Rac1* localizes to the leading edge of a crawling cell. It regulates adhesion and movement by promoting actin cytoskeleton remodeling [216].
	← ComplProc	PAK1	Active *PAK1* coordinates a series of cytoskeletal changes at the leading edge that are required for migration and invasion. These include: a) inhibition of myosin light chain kinase in order to decrease contractility if the leading lamellipodium and loss of established actin stress fibers and very strong *focal adhesions* at the leading edge; b) retraction of protrusions and the cell body at the sides and trailing edge with no active *PAK1*; c) suppressing actin filament turnover and promoting leading edge stabilization; d) promote membrane ruffle formation [214, 215].

Table S1l: Migration_SW module

Fast _Migration	**Fast_Migration** = **Migration** and ((**Horizontal_Pol** and **Stress_Fibers**) and **FocalAdhesions**) and **PAK1** and **Rac1_H**		
	MSt	In our model, the *Fast_ Migration* node represents sustained mesenchymal migration of a polarized cell. This requires horizontal polarization, stress fiber maintenance, focal adhesion formation, high *Rac1* actibity, as well as *PAK1* kinase [214, 215].	
	← Ind	FocalAdhesions	Mesenchymal style migration requires force generation via stress fibers anchored by *focal adhesions* [214].
	← Ind	Stress _Fibers	Mesenchymal style migration requires force generation via stress fibers anchored by *focal adhesions* [214].
	← Ind	Horizontal _Pol	Directed mesenchymal style migration requires *horizontal cell polarization* with a well defined leading and trailing edge [214].
	← ComplProc	Rac1_H	*Rac1* localizes to the leading edge of a crawling cell. It regulates adhesion and movement by promoting actin cytoskeleton remodeling [216].
	← ComplProc	PAK1	Active *PAK1* coordinates a series of cytoskeletal changes at the leading edge that are required for migration and invasion. These include: a) inhibition of myosin light chain kinase in order to decrease contractility if the leading lamellipodium and loss of established actin stress fibers and very strong *focal adhesions* at the leading edge; b) retraction of protrusions and the cell body at the sides and trailing edge with no active *PAK1*; c) suppressing actin filament turnover and promoting leading edge stabilization; d) promote membrane ruffle formation [214, 215].
	← Per	Migration	The *Fast_ Migration* node can only turn on if the more moderate *Migration* node is active.

Table S1m: EMT module

Target Node	Node Gate<br>Node Type<br>Link Type	Node Description<br>Input Node	Link Description
SNAI1	**SNAI1** = (**PAK1** and (not **GSK3** or **NfkB** or (not **miR_34** and (**ZEB1_H** or **ZEB1**) and **Hif1a_basal**))) or (**NfkB** and not(**miR_34** and **GSK3**) and (**ZEB1_H** or ((**ZEB1** or **Hif1a_High**) and **PAK1**))) or (((**ZEB1_H** and **ZEB1**) or **bcatenin_Hif1a_complex** or **N_bcatenin_H**) and (not(**miR_34** or **GSK3**) or **NfkB** or **PAK1**)) or (**SMAD2_3_4** and not(**miR_34** or **GSK3**)) or (**HMGA1** and not **miR_34**) or **HMGA2**		

Table S1m: EMT module

	TF	*SNAI1*, also known as *Snail*, is a master inducer of EMT, responsible for starting the transcriptional cascade that locks in the EMT regulatory switch [22]. In epithelial cells *SNAI1* is repressed by RNA interference by *miR-34* [217] and protein degradation by *GSK3* [218]. Transcription by *NF-κB* [219], along with loss of repression by *GSK3* and nuclear localization by *PAK1* [220], can activate *SNAI1*, starting EMT. In committed mesenhymal or hybrid E/M cells *ZEB1* and/or *Hif-1α/β-catenin*[221, 222] lower the barrier to maitnaining high levels of *SNAI1*. Downstream of *TGFβ*, *HMGA2* further aids its transcriptiopnal upregulation [223], as does*HMGA1* [224].	
	⊢ Deg	GSK3	*GSK3* both degrades and prevents the transcription of *SNAI1* [218].
	← PLoc	PAK1	*PAK1* phosphorylation of *SNAI1* activates and localizes *SNAI1* the nucleus [220].
	← TR	NfkB	The transcription factor *NF-κB* promotes the expression of *SNAI1* [219], and *NF-κB* inhibition can lower *SNAI1* expression [219].
	← TR	SMAD2_3_4	Active nuclear *Smad2/3/4*s directly bind and activate the *SNAI1* promoter [225].
	← TR	HMGA2	*HMGA2* binds to the *SNAI1* promoter to induce its expression, and cooperates with *Smads* by binding to them and increasing their affinity to the *SNAI1* promoter [223]. Thus, *TGFβ* activates the EMT transciptional program via *HMGA2* [30].
	⊢ RNAi	miR_34	*SNAI1* mRNA is a direct target of *miR-34* suppression [217].
	← Ind	ZEB1_H	*ZEB1* is an indirect transcriptional inducer of *SNAI1* [226], likely by acting as a competitive inhibitor of microRNAs such as *miR-34* [227].
	← Ind	ZEB1	*ZEB1* is an indirect transcriptional inducer of *SNAI1* [226], likely by acting as a competitive inhibitor of microRNAs such as *miR-34* [227].
	← Ind	HMGA1	siRNA against *HMGA1* strongly reduces *SNAI1* protein expression, though it is unclear whether this is the result of direct transcriptional repression similar to *HMGA2* [224].
	← TR	bcatenin_Hif1a_complex	Complex formation between *Hif-1α* and *β-catenin*, aided by active *Src* and ROS, is required for increased *SNAI1* under hypoxia; inhibitors of *Hif-1α*, *β-catenin*, *Src* or ROS abrogate *SNAI1* expression [222].
	← TR	N_bcatenin_H	Downregulation of *β-catenin* leads to significant reduction of *SNAI1* mRNA levels [228, 222].
	← TR	Hif1a_basal	*SNAI1* is a direct transcriptional target of *Hif-1α* [221].
	•– TR	Hif1a_High	*SNAI1* is a direct transcriptional target of *Hif-1α* [221].
LEF1	**LEF1** = (**ZEB1** and not **miR_200_c**) or **ZEB1_H** or **NfkB** or **SMAD2_3_4**		

Table S1m: EMT module

	TF	Lymphoid enhancer-binding factor 1, or *LEF1* is a high-mobility group transcription factor and mediator of *Wnt/β-catenin* signaling. In addition to promotong proliferation, *LEF1* helps induce EMT by activating the transcription *SNAI2* and *ZEB1* [229]. It is induced by *NF-κB* [230] or *Smad2/4* [231], its transcriptional potency is boosted by *ZEB1* [232], and it is targeted for degradation by *miR-200*.	
	← TR	NfkB	*LEF1* is a direct transcriptipnal target of *NF-κB* [230].
	← TR	SMAD2_3_4	Phosphorilated *Smad2* and *Smad4* can induce *LEF1* gene expression [231].
	⊢ Ind	miR_200_c	*miR-200a-3p* is an indirect repressor of *LEF1*, as it limiting basal *Pitx2* and *β-catenin* complexes from inducing *LEF1* transcription [233]. Here we assume that medium *ZEB1* availability can aid *LEF1*-mediated transcription if *miR-200* is repressed, while high levels of *ZEB1* can override *miR-200*.
	← Compl	ZEB1_H	*ZEB1* can bind to and significantly boost *LEF1*-mediated transcription [232].
	← Compl	ZEB1	*ZEB1* can bind to and significantly boost *LEF1*-mediated transcription [232].
Twist	**Twist** = not **Casp3** and (**HMGA2** or **HMGA1** or **bcatenin_Hif1a_complex** or (**SNAI1** and **NfkB** and (not **miR_34** or **YAP** or (**N_bcatenin** and **Hif1a_High** and **Src_High**))))		
	TF	*Twist* is a master trancriptional regulator of the EMT program, induced by *NF-κB* [234] or *HMGA2* [235], stabilized and aided by *SNAI1*, repressed by *miR-34* unless induced by *YAP*, and cleaved during apoptosis by *Caspase 3* [236]. Under hypoxia, *Twist* is induced by complex formation between *Src*-phosphorylated *β-catenin* and *Hif-1α* [222].	
	← Ind	Src_High	Under hypoxia, increased levels of Twist are dependent on complex formation between *Src*-phosphorylated *β-catenin* and *Hif-1α* [222].
	← TR	YAP	*YAP1/TEAD1* complexes bind to the *Twist1* promoter and promote *Twist1* expression [237].
	← TR	NfkB	Transcription of *Twist* is induced by *NF-κB* [234].
	← TR	N_bcatenin	Under hypoxia, increased levels of Twist are dependent on complex formation between *Src*-phosphorylated *β-catenin* and *Hif-1α* [222].
	← TR	HMGA2	*HMGA2* is a direct transcriptional inducer of *Twist*, and responsible for its increase in response to *TGFβ* [235].
	← Ind	SNAI1	*SNAI1* is required a rapid inrease in *Twist* protein levels, and aids its subsequent transcription in response to *TGFβ* [238]. In addition, *SNAI1* potentiates *Twist*-mediated enhancer activation [239].

Table S1m: EMT module

	⊣ RNAi	miR_34	*Twist*'s 3'UTR is a direct target of *miR-34* [240].
	← TR	HMGA1	*Twist1* mRNA strongly expression was repressed in *HMGA1* knock-down cells compared to controls [241].
	← TR	bcatenin _Hif1a _complex	*Twist* is a direct transcriptional target of *Hif-1α* [222]. Under hypoxia, increased levels of *Twist* are dependent on complex formation between *Src*-phosphorylated *β-catenin* and *Hif-1α* [222].
	← TR	Hif1a_High	*Twist* is a direct transcriptional target of *Hif-1α* [222]. Under hypoxia, increased levels of Twist are dependent on complex formation between *Src*-phosphorylated *β-catenin* and *Hif-1α* [222].
	⊣ Lysis	Casp3	*Twist* is a direct proteolytic target of *Caspase 3* [236].
SNAI2	**SNAI2** = (**Twist** and (**SNAI2** or **N_bcatenin** or (**N_bcatenin_H** and **LEF1**))) or **SMAD2_3_4** or **HMGA2** or **HMGA1**		
	TF		*SNAI2*, also known as *Slug*, is a prototypical EMT transcription factor that regulates tissue development and tumorigenesis [242]. It is induced / maintained in mesenhymal cells by *Twist* [243], *β-catenin* [244, 242], *LEF1* [245] as well as positive auto-regulation [246]. In response to *TGFβ* it is induced by *Smad3* [247] and *HMGA1/2* [248, 30].
	← TR	N_bcatenin	The *Wnt/β-catenin* pathway promotes *SNAI2* transcription through nuclear *β-catenin* [244, 242].
	← TR	SMAD2_3 _4	*SMAD3* bind the cis-element in the SNAI2 promoter and recruits myocardin-related transcription factors to activate its transcription [247].
	← TR	HMGA2	*HMGA2* is a direct transcriptional inducer of *SNAI2* [30].
	← TR	LEF1	*LEF1* is a direct transcriptional inducer of *SNAI2* [245].
	← TR	SNAI2	*SNAI2* is able to bind to its own promoter and induce transcription of its own mRNA [246].
	← TR	Twist	*Twist* is a direct transcriptional inducer of *SNAI2* [243].
	← Ind	HMGA1	Loss of *HMGA1* strongly reduces *SNAI2* levels, though it is unclear whether this is the result of direct transcriptional repression [248].
	← TR	N_bcatenin _H	The *Wnt/β-catenin* pathway promotes *SNAI2* transcription through nuclear *β-catenin* [244, 242].
ZEB1	**ZEB1** = **SNAI2** or (**Twist** and **SMAD2_3_4**) or ((**b_catenin_TCF4** or **Hif1a_High**) and not(**miR_200_b** or **miR_200_c**))		

Table S1m: EMT module

	TF		Zinc finger E-box binding homeobox 1 or *ZEB1* is one of the core regulators of the EMT transcriptional switch [249]. It is induced by *SNAI2* [250], nuclear β-*catenin/TCF4* [251], *Twist* aided by *SMAD2/3/4*, or high levels of *Hif-1*α. The epithelial microRNA *miR-200* targets its mRNA for destruction [252, 253]. As *ZEB1* has two distinct activation levels in hybrid E/M cells vs fully mesenchynmal ones [254, 255, 256], we modeled *ZEB1* activity with two nodes; this one represents at least medium *ZEB1* activity (characteristic of hybrid E/M cells and compatible with ongoing *miR-200* expression), and the *ZEB1_H* node representing maximal *ZEB1* activation seen in fully mesenchymal cells.
	← TR	SMAD2_3_4	The type I *TGF*-β receptor (*TβRI*) phosphorylates receptor-bound *Smad2* and *Smad3*, which triggers their nuclear translocation. Together with their activating partner *Smad4*, they aid (and are required for) *Twist*-induced transcription of *Zeb1* [20].
	← TR	SNAI2	*SNAI2* promotes *ZEB1* transcription [250].
	← TR	Twist	*Twist* works together with *Smad2/3/4* active complexes to induce *Zeb1* expression [20].
	⊣ RNAi	miR_200_b	*miR-200* inhibits mRNA expression of *ZEB1* by targeting its mRNA for destruction [257, 252, 253].
	⊣ RNAi	miR_200_c	*miR-200* inhibits mRNA expression of *ZEB1* by targeting its mRNA for destruction [257, 252, 253].
	← TR	b_catenin_TCF4	Nuclear β-*catenin/TCF4* are direct transcriptional inducers of the *ZEB1* promoter [251].
	← TR	Hif1a_High	*Hif-1*α directly promotes *ZEB1* activation through binding to its HRE-3 under hypoxic conditions [258].
N_bcatenin_H	**N_bcatenin_H** = **N_bcatenin** and not **miR_34** and (not **J_acatenin** or **SMAD2_3_4**) and (not(**miR_200_c** or **miR_200_b**) or not **GSK3** or **Hif1a_High**) and not((**CyclinE** or **CyclinA**) and **GSK3**)		
	TF		The *N_bcatenin_H* node represents maximal nuclear β-*catenin* accumulation. This requires *N_bcatenin* = ON, a lack of *miR-34*, either complete absence of junctions (*J_acatenin* = OFF) or activation by *Smads*, and the lack of joint repression *GSK3* and either *miR-200* or *CyclinE/A*-bound *Cdk2*.
	⊣ Deg	GSK3	When *GSK3* is inhibited, unphosphorylated β-*catenin* accumulates, translocates to the nucleus, and promotes transcription [176].
	⊣ Loc	J_acatenin	Junctional *E-cadherins* recruits/sequesters β-*catenin* to adherens junctions and block β-*catenin* nuclear localization [149]. We assume that cells unable to form any adherens junctions have high nuclear β-*catenin*.
	← Per	N_bcatenin	Reaching the *N_bcatenin_H* = ON state requires *N_bcatenin* = ON first.

Table S1m: EMT module

	← P	SMAD2_3_4	*TGFβ* stimulation leads to increased binding of *SMAD3/4* with *β-catenin*, which in turn increases its nuclear accumulation and *β-catenin*-mediated transcription [259, 260].
	⊢ RNAi	miR_34	*β-catenin*'s 3' UTR is a direct *miR-34* target [261].
	⊢ RNAi	miR_200_b	*β-catenin*'s 3' UTR is a direct *miR-200* target [262].
	⊢ RNAi	miR_200_c	*β-catenin*'s 3' UTR is a direct *miR-200* target [262].
	← Loc	Hif1a_High	*Hif-1α* expression increases nuclear translocation of *β-catenin* [177].
	⊢ P	CyclinE	*cyclin E/Cdk2* phosphorylate *β-catenin* on Ser33, Ser37, Thr41, and Ser45, promoting its rapid proteasomal degradation [263].
	⊢ P	CyclinA	*cyclin A/Cdk2* phosphorylate *β-catenin* , promoting its rapid degradation [263].
ZEB1_H	**ZEB1_H** = **ZEB1** and **LEF1** and (**SNAI2** or not **miR_200_c**) and ((**N_bcatenin_H** or **Hif1a_High** or **b_catenin_TCF4** or **HMGA1**)) and **Hif1a_basal**		
	TF		The *ZEB1_H* node represents maximal *ZEB1* activation seen in fully mesenchymal cells [254, 255, 256]. In our model this requires medium *ZEB1*, as well as *LEF1* [264], either *SNAI2* [250] or the absence of *miR-200* [252, 253], as well as either high nuclear *β-catenin* [251]high *Hif-1α*[258], *β-catenin/TCF4*[251], or *HMGA1*[265].
	← Ind	LEF1	*LEF1* overexpression lead to a substantial increase in *ZEB1*, indicating that elevated levels of *LEF1* can help push *ZEB1* into its mesenchymal-specific high expression range (*LEF1_H* node) [264].
	← TR	SNAI2	*SNAI2* promotes *ZEB1* transcription [250].
	⊢ RNAi	miR_200_c	*miR-200* reduces mRNA expression of *ZEB1* by targeting its mRNA for destruction [257, 252, 253].
	← Per	ZEB1	As the *ZEB1* node represents moderate levels of this trascription factor, *ZEB1_H* = ON requires *ZEB1* = ON.
	← Ind	HMGA1	*HMGA1* is a transcriptional inducer of *JMJD3*, which in turn directly demethylates the *ZEB1* promoter to increasde its transcription [265].
	← TR	N_bcatenin_H	Nuclear *β-catenin/TCF4* are direct transcriptional inducers of the *ZEB1* promoter [251]. We assume that high levels of nuclear *β-catenin* can drive *ZEB1_H* levels.
	← TR	b_catenin_TCF4	Nuclear *β-catenin/TCF4* are direct transcriptional inducers of the *ZEB1* promoter [251].
	← TR	Hif1a_basal	*Hif-1α* directly promotes *ZEB1* activation under hypoxic conditions by binding to its HRE-3 region. [258]

Table S1m: EMT module

	← TR	Hif1a_High	*Hif-1α* directly promotes *ZEB1* activation under hypoxic conditions by binding to its HRE-3 region. [258]
b_catenin_TCF4	**b_catenin_TCF4** = **N_bcatenin_H** and **SNAI1** and **SNAI2** and not **miR_200_c** and not(**Hif1a_High** and **ROS**)		
	PC		The *b_catenin_TCF4* node represents saturating levels of active nuclear *β-catenin/TCF4* transcriptional activity. In addition to the influences required to accumulate high nuclear *β-catenin*, this node's ON state also requires *SNAI1* and *SNAI2* expression (both factors promote the formation of active *β-catenin/TCF4* transcriptional complexes) [266] and low *miR-200c* levels. High levels of *Hif-1α*, aided by ROS, sequester *β-catenin* away from *TCF4* [267].
	← Ind	SNAI1	*SNAI1/2* promote the formation of active *β-catenin/TCF4* transcriptional complexes [266].
	← Ind	SNAI2	*SNAI1/2* promote the formation of active *β-catenin/TCF4* transcriptional complexes [266].
	⊢ RNAi	miR_200_c	*β-catenin*'s 3' UTR is a direct *miR-200* target [262].
	← Compl	N_bcatenin_H	Reaching saturating levels of active nuclear *β-catenin/TCF4* complex formation is aided by maximal nuclear *β-catenin* accumularion (*N_bcatenin_H* = ON).
	⊢ Compl	Hif1a_High	*Hif-1α* binds to *β-catenin* with a higher affinity than *TCF4*, diverting *β-catenin* away from *β-catenin/TCF4* complex formation [267].
	⊢ Ind	ROS	ROS activty is necessary to form *β-catenin/Hif-1α* formation, which in turn block *β-catenin-TCF4* complex formation [222]
ZEB2	**ZEB2** = not **miR_200_c** and ((**NfkB** and **Hif1a_basal**) or **SMAD2_3_4** or **Hif1a_High**)		
	TF		Zinc finger E-box binding homeobox 2 or *ZEB2* is a core regulator of the EMT transcriptional switch. *ZEB2* is transcriptionally induced by either *NF-κB* aided by moderate *Hif-1α*, *SMAD2/3/4* [268], or high levels of *Hif-1α*. It is blocked by *miR_200c*-mediated mRNA degradation [257].
	← TR	NfkB	*NF-κB* is a direct transcriptional inducer of *ZEB2* downstream of textitTNF-α or *IL1* signaling [268].
	← TR	SMAD2_3_4	*SMAD2/3/4* complexes are direct transcriptional inducers of *ZEB2* downstream of textitTGF-β signaling [268].
	⊢ RNAi	miR_200_c	*miR_200c* inhibits mRNA expression of *ZEB2* by targeting its mRNA for destruction [257].
	← TR	Hif1a_basal	*Hif-1α* is a direct transcriptional inducer of *ZEB2*[268].
	← TR	Hif1a_High	*Hif-1α* is a direct transcriptional inducer of *ZEB2*[268].
HMGA1	**HMGA1** = **ZEB2** or (**b_catenin_TCF4** and **N_bcatenin_H**)		

Table S1m: EMT module

	TF		*HMGA1* is a high mobility group (HMG) proteininvolved in transcriptional regulation via chromatin remodeling. It is directly induced by *β-catenin/TCF-4*-mediated transcription [269], and indirectly stabilized by *ZEB2* repression of the the *miR-637* mRNA (which targets its mRNA for degradation) [270].
	← Ind	ZEB2	*ZEB2* is a direct transcriptional repressor of the *miR-637* promoter, a microRNA that targets *HMGA1* mRNA for destruction [270].
	← Ind	N_bcatenin_H	*β-catenin/TCF-4* complexes are direct transcriptional inducers of *HMGA1* [269].
	← Ind	b_catenin_TCF4	*β-catenin/TCF-4* complexes are direct transcriptional inducers of *HMGA1* [269].
TGFb_secr	**TGFb_secr** = (**b_catenin_TCF4** or **Hif1a_High** or (not **pVHL** and **Hif1a_basal**)) and not(**miR_200_b** or **miR_200_c**) and not **Casp3**		
	Secr		The *TGFb_secr* node represents the production and secretion of *TGFβ* by the modeled cell. *TGFβ* transcription in mesenchymal cells is driven by nuclear *β-catenin/TCF4*-mediated transcription, while *TGFβ* mRNA is targeted for degradation by *miR-200* [21]. Alternately, hypoxia or loss of *VHL* protein allows *Hif-1α* to increase *TGFβ* secretion[271, 272]. Finally, apoptotic *Caspase 3* disrupts all ER-mediated protein secretion[273].
	⊣ RNAi	miR_200_b	Overexpression of *miR-200* significantly reduces mRNA levels of *TGFβ*, with the strongest effect on *TGFβ3* [21].
	⊣ RNAi	miR_200_c	Overexpression of *miR-200* significantly reduces mRNA levels of *TGFβ*, with the strongest effect on *TGFβ3* [21].
	← TR	b_catenin_TCF4	*TGFβ3* is directly regulated by activated *β-catenin* [274], while *SNAI1/2* promote the formation of *β-catenin/TCF4* complexes, which bind to the *TGFβ3* promoter induce its transcription [266].
	← Ind	Hif1a_basal	*Hif-1α* is implicated with increased endogenous *TGFβ* secretion, mediated by the upregulation of *Furin*, an enzyme that converts secreted but inactive *TGFβ* to its active form [271, 272].
	← Ind	Hif1a_High	*Hif-1α* is implicated with increased endogenous *TGFβ* secretion, mediated by the upregulation of *Furin*, an enzyme that converts secreted but inactive *TGFβ* to its active form [271, 272].
	⊣ Ind	pVHL	We assume that the loss of *VHL* protein can boost the effect of *Hif-1α* in boosting the levels of active secreted *TGFβ*[271, 272].
	⊣ Ind	Casp3	During apopotsis, caspases cleave key components vesicular transport between the ER and Golgi, resulting in a complete block in ER-mediated protein secretion [273].
miR_34	**miR_34** = (not **SNAI1**) or (not(**ZEB1** or **ZEB1_H**))		

Table S1m: EMT module

	miR	*miR-34* is an microRNA expressed in epithelial cells and central to blocking the accumulation of EMT-initiating transcription factors such as *SNAI1* and *β-catenin* [275]. *SNAI1*, together with *ZEB1*, feed back to repress *miR-34* expression in mesenhymal and hybrid E/M cells [276].	
	⊢ TR	SNAI1	*SNAI1* is a direct inhibitor of *miR-34* transcription [276].
	⊢ TR	ZEB1_H	*ZEB1* is a direct inhibitor of *miR-34* transcription [276].
	⊢ TR	ZEB1	*ZEB1* is a direct inhibitor of *miR-34* transcription [276].
miR_200_b	**miR_200_b** = **p21** and not((**Twist** and **SNAI1** and (**ZEB1** or not **c_Myb**)) or **ZEB1_H** or **ZEB2**)		
	miR	The *miR-200b* microRNA is expressed in epithelial cells and is central to blocking EMT transcription factors [277]. Its levels are increased by *p21* [278], and it is directly induced by *c-Myb* [127] and repressed by *ZEB1* [279, 280]. This repression is indirectly supported by *SNAI1* [281, 282] and *Twist* [238]. Here we assume that in addition to the absence of *p21* and the aid of *SNAI1* and *Twist*, either *ZEB2* or high levels of *ZEB1* (*ZEB_H* = ON) are required to silence the active *miR-200* promoter. In contrast, medium *ZEB1* can maintain repression as long as *miR-200* inducer *c-Myb* is off.	
	← TR	c_Myb	The proto-oncogene *c-Myb* induces the expression of the *miR-200* family, unless the locus is silenced by DNA methylation [127].
	⊢ Ind	SNAI1	*SNAI1* induction reduces the expression of *miR-200* [281, 282].
	⊢ Ind	Twist	*Twist* upregulation is required to maintain high levels of *ZEB1* (*Twist* is a direct enhancer of *ZEB1*) [238]. Thus, we assume that *ZEB1* and *SNAI1* cannot fully repress the *miR-200* cluster in the absence of *Twist*. In addition, overexpression of *Twist* resulted in DNA methylation of the *miR-200* locus, though this effect is likely the indirect result of *ZEB1*-mediated repression [283].
	⊢ TR	ZEB1_H	*ZEB1* is a direct transcriptional repressor of *miR-200* expression [279, 280].
	⊢ TR	ZEB1	*ZEB1* is a direct transcriptional repressor of *miR-200* expression [279, 280].
	⊢ TR	ZEB2	Overexpression of *ZEB2* suppressed the activity of the *miR-200b-200a-429* promoter [284]; *ZEB2* is a direct transcriptional repressor of the *miR-200* cluster [279, 280, 285].

Table S1m: EMT module

	← Ind	p21	*p21* knockdown downregulates several EMT-blockign miRNAs, including *miR-200a*, *miR-200b*, *miR-200c* and the *miR-183-96-182* cluster. This inhibits EMT, migration and invasion [278]. While it is unclear if *p21* is a direct repressor of the *miR-200* cluster, it was shown to bind *ZEB1* and inhibit its transcriptipnal effets, relieving its ability to repress the *miR-183-96-182* cluster [278]. As this cluster, not explicitly accounted for in our model, further inhibits *ZEB1* expression [278], it is possible that *p21* increases *miR-200* levels indirectly via this cluster, directly by blocking *ZEB1*-mediated repression, or both.
miR_200_c	**miR_200_c** = not(**Twist** and **SNAI1** and (**ZEB1_H** or **ZEB2** or (**ZEB1** and not(**miR_200_c** or **c_Myb**))))		
	miR		The *miR-200c* microRNA is expressed in epithelial cells and is central to blocking EMT transcription factors [277]. It is directly induced by *c-Myb* [127] and repressed by *ZEB1* or *ZEB2* [279, 280, 279, 280, 285]. This repression is indirectly supported by *SNAI1* [281, 282] and *Twist* [238]. Here we assume that in addition to the aid of *SNAI1* and *Twist*, high levels of *ZEB1* (*ZEB_H* = ON) or *ZEB2* are required to silence the active *miR-200* promoter. In contrast, medium *ZEB1* can maintain repression as long as *miR-200* is silenced and its inducer *c-Myb* is off.
	← TR	c_Myb	The proto-oncogene *c-Myb* induces the expression of the *miR-200* family, unless the locus is silenced by DNA methylation [127].
	⊢ Ind	SNAI1	*SNAI1* induction reduces the expression of *miR-200* [281, 282].
	⊢ Ind	Twist	*Twist* upregulation is required to maintain high levels of *ZEB1* (*Twist* is a direct enhancer of *ZEB1*) [238]. Thus, we assume that *ZEB1* and *SNAI1* cannot fully repress the *miR-200* cluster in the absence of *Twist*. In addition, overexpression of *Twist* resulted in DNA methylation of the *miR-200* locus, though this effect is likely the indirect result of *ZEB1*-mediated repression [283].
	← Epi	miR_200_c	As the *miR-200* promoter is subject of DNA methylaiton and epigenetic silencing during EMT [283], we assume that it is more difficult to turn off than to maintain its silenced state.
	⊢ TR	ZEB1_H	*ZEB1* is a direct transcriptional repressor of *miR-200* expression [279, 280].
	⊢ TR	ZEB1	*ZEB1* is a direct transcriptional repressor of *miR-200* expression [279, 280].
	⊢ TR	ZEB2	*ZEB2* is a direct transcriptional repressor of *miR-200* expression [279, 280, 285].
Ecadherin_mRNA	**Ecadherin_mRNA** = not(((**ZEB1_H** and **ZEB2**) or (not **MCRIP1** and (**ZEB1_H** or **ZEB2**))) and **ZEB1** and **SNAI1** and **SNAI2** and **Twist**)		

Table S1m: EMT module

	mRNA		The *Ecadherin_mRNA* represents basal *E-cadherin* expression, required to make at least some adherens juncitons with neighbors. This basal expression is only blocked during full EMT by the joint action of *ZEB1* [286], *SNAI1* [287, 257, 276], *SNAI2* [287, 288, 257], *Twist* [289, 276], and*ZEB2*; protected from repression by *MCRIP1* [290].
	← Ind	MCRIP1	*MCRIP1* promotes transcription of *E-cadherin* by competitively binding to *ZEB1* co-repressor *CtBP*, thus inhibiting *ZEB1*-mediated transcriptional repression [290]. During EMT, *MCRIP1* is inhibited by *ERK* phosphorylation [290].
	⊢ TR	SNAI1	*SNAI1* is a direct transcriptional inhibitor of *E-cadherin* [287, 257, 276].
	⊢ TR	SNAI2	*SNAI2* is a direct transcriptional inhibitor of *E-cadherin* transcription [287, 288, 257].
	⊢ TR	Twist	*Twist* is a direct transcriptional inhibitor of *E-cadherin* transcription [289, 276].
	⊢ TR	ZEB1_H	*ZEB1* is a direct transcriptional inhibitor of *E-cadherin* [286], either in partnership with its co-repressor *CtBP* [291], or by binding to the SWI/SNF chromatin-remodeling protein *BRG1* [292].
	⊢ TR	ZEB1	*ZEB1* is a direct transcriptional inhibitor of *E-cadherin* [286], either in partnership with its co-repressor *CtBP* [291], or by binding to the SWI/SNF chromatin-remodeling protein *BRG1* [292].
	⊢ TR	ZEB2	*ZEB2* is a direct transcriptional inhibitor of *E-cadherin* [293].
bcatenin _Hif1a _complex	**bcatenin_Hif1a_complex** = **Src_High** and **Hif1a_High** and (**N_bcatenin_H** or **b_catenin_TCF4**) and **ROS**		
	PC		The *β-catenin-Hif-1α* node represents complexes formed by nuclear *β-catenin* and *Hif-1α* under hypoxic conditions, promoting transcription of mesenchymal-associated genes [222]. This complex formation is aided by high levels of active *Src* and a hypoxic increase in ROS[222].
	← P	Src_High	*Src* induced Y654 phosphorylation of *β-catenin* is required for *β-catenin-Hif-1α* complex formation [222, 120].
	← Compl	N_bcatenin _H	Nuclear *β-catenin* binds to *Hif1α* leading to *Src* dependent complex formation [222].
	← Compl	b_catenin _TCF4	Nuclear *β-catenin* binds to *Hif1α* leading to *Src* dependent complex formation [222].
	← Compl	Hif1a_High	Accumulation of *Hif1α* under hypoxic conditions leads to ROS dependent complex formation with nuclear *β-catenin* [222].
	← Ind	ROS	ROS is required for high levels of *Src* kinase activity responsible for complex formation [222].

Table S1n: Restriction_SW module

Target Node	Node Gate / Node Type / Link Type	Node Description / Input Node	Link Description
p21	**p21** = **p21_mRNA** and not **Casp3** and (not **CyclinE** or not **Myc**)		
	Prot	In this model, the *p21* node corresponds to nuclear p21 in cells with relatively high basal *p21* activity. *p21* activity and or localization can be lowered by loss of *FoxO* mediated transcription (see $p21_{mRNA}$ node), via feedback from *Cyclin E/Cdk2* [294], or repression by *Myc*[295].	
	← TL	p21 _mRNA	*p21* protein activity requires the presence of *p21* transcription.
	⊢ TR	Myc	High *Myc* expression decreases endogenous *p21* levels by direct promoter binding, preventing transcription. *Myc* is also suspected to sequester the *p21* activator *Sp1* [295].
	⊢ Deg	CyclinE	*p21* and *Cyclin E/Cdk2* form a positive (double-negative) feedback loop in which *Cyclin E/Cdk2* activates the *SCF/Skp2* complex responsible for the degradation of *Cyclin E/Cdk2*-bound, phosphorylated *p21* [296]. *p21*, in turn, not only blocks *Cyclin E/Cdk2* activity, but it also inhibits *Cyclin D1*. Thus, *p21* interferes with the mitogen signal that turns on *Cyclin E* in the first place. In quiescent cells with high basal *p21* levels, this positive feedback renders cell cycle entry stochastic [294].
	⊢ Lysis	Casp3	*Caspase 3* cleaves and deactivates *p21* [297].
pRB	**pRB** = (((not **Casp3**) and (not **CyclinD1**)) and (not **CyclinA**)) and (**p27Kip1** or (not **CyclinE**))		
	TF	*pRB* is active in the absence of *Caspase 3*, *Cyclin D1*, *Cyclin A*, and *Cyclin E*. In addition, *pRB* maintains its activity when active $p27^{Kip1}$ counteracts the effects of *Cyclin E* [298, 299, 300, 301].	
	← ComplProc	p27Kip1	Active $p27^{Kip1}$ can counteract the inhibitory effects of active *CyclinE/Cdk2* complexes [298].
	⊢ P	CyclinD1	*Cyclin D1/Cdk4,6* complexes bind and phosphorylate *RB*, inhibiting its activity [302, 303, 300].
	⊢ P	CyclinE	*Cyclin E/Cdk2* complexes bind and phosphorylate *RB*, inhibiting its activity [304, 300].
	⊢ P	CyclinA	*Cyclin A/Cdk1,2* complexes phosphorylate and deactivate *RB* [299].
	⊢ Lysis	Casp3	*Caspase 3* cleaves *RB*, generating fragments that do not associate with *E2F1*, rendering *RB* inactive [305].
p27Kip1	**p27Kip1** = (((not **Casp3**) and (not **CyclinD1**)) and (not(**Cdk1** and **CyclinB**))) and ((((not(**CyclinA** and **Necl5** and **CyclinE**)) and (**FoxO3** and **FoxO1**)) or (((not **CyclinA**) or (not(**Necl5** or **CyclinE**))) and (**FoxO3** or **FoxO1**))) or ((not **CyclinA**) and (not(**Necl5** and **CyclinE**))))		

Table S1n: Restriction_SW module

Prot	Active $p27^{Kip1}$ is cleaved by *Caspase 3* and inhibited (sequestered) by *Cyclin D1/Cdk4,6* [298] or *Cyclin B/Cdk1* [306]. In addition, maintenance of $p27^{Kip1}$ requires one or both *FoxO* factors when sequestered by *Cyclin E/Cdk2* (one *FoxO* factor) or *Cyclin A/Cdk2* (both *FoxO* factors), but it cannot keep pace with the simultaneous activity of *Cyclin E/Cdk2* and *Cyclin A/Cdk2* [307].	
← TR	FoxO3	*FoxO* factors are direct inducers of $p27^{Kip1}$ expression [308].
← TR	FoxO1	*FoxO* factors are direct inducers of $p27^{Kip1}$ expression [308].
⊢ TR	Necl5	*Necl5* downregulates the transcription of *p27Kip1* in response to growth factor stimulation [309].
⊢ IBind	CyclinD1	Active *Cyclin D/Cdk4,6* complexes competitively bind to *p27Kip1* and progressively inhibit its ability to keep *Cyclin-E/Cdk2* inactive, thereby inducing cdk2 activity and cell-cycle progression [298].
⊢ Deg	CyclinE	Active *Cyclin-E/Cdk2* phosphorylate *p27Kip1* at threonine 187 (Thr187) [310], which marks it for degradation by the SCF^{SKP2} complex at the onset of S-phase [311]. (*Cyclin-E/Cdk2* complexes remain active in the presence of *p27Kip1* and promote its degradation when *Cyclin-A* is also active.)
⊢ Deg	CyclinA	*Cyclin A/Cdk2* complexes bind and inactivate $p27^{Kip1}$ by sequestration, phosphorylate it, and promote its degradation [312].
⊢ PLoc	CyclinB	*Cyclin B/Cdk1* complexes phosphorylate $p27^{Kip1}$ [312], and although they do not promote its degradation, phosphorylated $p27^{Kip1}$ is exported from the nuclear compartment and looses its ability to inhibit *Cdk* activity [306].
⊢ PLoc	Cdk1	*Cyclin B/Cdk1* complexes phosphorylate $p27^{Kip1}$ [312], and although they do not promote its degradation, phosphorylated $p27^{Kip1}$ is exported from the nuclear compartment and looses its ability to inhibit *Cdk* activity [306].
⊢ Lysis	Casp3	*Caspase 3* cleaves *p27Kip1* [313]; the cleaved fragments can no longer associate with *Cdk2 / Cyclin* complexes [314].

Myc	**Myc** = not **Hif1a_High** and ((**ERK** and **YAP** and not **SMAD2_3_4** and (**b_catenin_TCF4** or not **bcatenin_Hif1a_complex**)) or ((**ERK** or (**YAP** and **b_catenin_TCF4** and not **SMAD2_3_4**)) and **eIF4E** and not **GSK3**) or (**E2F1** and not **pRB** and (**eIF4E** or **ERK** or not **GSK3**)))

Table S1n: Restriction_SW module

	TF	*Myc* activity is turned on by stabilization of the protein via *ERK* phosphorylation, aided by *YAP*-mediated transcription [315, 316] in the absence of inhibitory *SMADs* [317]and aided by *TCF4/β-catenin*, the absence of inhibitory */beta-catenin-Hif-1α* complexes. To take into account the increase in translation initiated by *eIF4E* and loss of degradation-promoting phosphorylation when *GSK3β* is off [318], we assumed that they can compensate for the lack of *ERK*, absence of *YAP*, or interference from *SMADs*. Alternatively, increased transcription by *E2F1* can also promote *Myc* accumulation in the absence of active *pRB* [319], provided that the protein is stabilized by *ERK*, *eIF4E*, or the absence of *GSK3β*. Lastly, high levels of *Hif-1α* repress *Myc* activity[320].	
	← P	ERK	Ser-62 phosphorylation by *ERK* increases its half life, leading to *Myc* accumulation [318, 321].
	← TR	eIF4E	Increased translational initiation in the presence of activated *eIF4E* leads to an increase in *Myc* protein levels [322].
	⊢ P	GSK3	Thr-58 phosphorylation by *GSK-3* promotes *Myc* degradation [318, 323].
	← TR	YAP	*YAP* is a transcriptional inducer of /textitc-Myc [315, 316].
	⊢ TR	SMAD2_3_4	*TGFβ* stimulation induces *Smad* complex formation that recognizes a *TGFβ* inhibitory element in the *c-Myc* promoter [317]. This inhibition may be partially rescued by *YAP*, as *YAP/Smad7* complexes were shown to interfere with *Smad*-dependent gene expterssion control [324].
	⊢ Ind	bcatenin_Hif1a_complex	Hypoxia promotes */beta-catenin-Hif-1α* complex formation, which abrogates *Myc* expression and halts the cell cycle [325].
	← TR	b_catenin_TCF4	*TCF4/β-catenin* complexes are direct transcriptional inducers of *Myc*[326].
	⊢ IBind	Hif1a_High	*Hif-1α* binds to *Myc* coactivator *Sp1*, preventing transcription of *Myc* activated genes [320].
	⊢ TR	pRB	*E2F1*'s ability to induce *Myc* is blocked by active (hypophosphorylated *pRB*) [327].
	← TR	E2F1	*E2F1* binds and activates the *c-Myc* promoter [328, 329].
CyclinD1	**CyclinD1** = not **p15** and not **CHK1** and ((not **p21** and ((not **GSK3** and **YAP** and (**Myc** or **E2F1**)) or (**CyclinD1** and **YAP** and (**Myc** or **E2F1**)) or (**Myc** and **E2F1**))) or (not **pRB** and **E2F1** and ((**Myc** and **CyclinD1**) or (**Myc** and not **GSK3**) or (**YAP** and **CyclinD1** and not **GSK3**))))		

Table S1n: Restriction_SW module

PC	Ongoing DNA synthesis keeps the *CHK1* kinase active, which inhibits *Cyclin D1*. Similarly, the CDKI *p15* also binds and blocks active *Cdk/Cyclin D* complexes. The precise regulatory logic of *Cyclin D1* as a function of transcriptional control by *Myc* and *E2F1*, combined with the regulation of its protein stability / activity by *GSK3β* / basal *p21* is not known. Here, we assume that in the absence of *p21* (once *p21* levels drop due to growth factor signals and/or *Cdk2* activation), *Cyclin D1* can be activated by *YAP* and either *Myc* or *E2F1* – as long as *GSK3β is OFF*. In the presence of *GSK3β*, we assume that *Cyclin D1* can be induced by the combined action of both *Myc* and *E2F1* [330], but sustained in an ON state by either. In the presence of basal (normal quiescent) levels of *p21*, we assume that *Cyclin D1* transcription requires *E2F1* unencumbered by *pRB*, as well as any two of the following: *Myc*, already active *Cyclin D1*, sustained by *YAP* and not blocked by *GSK3β*.	
⊣ P	GSK3	*GSK-3β* phosphorylates *Cyclin D1* on Thr-286, promoting its ubiquitination and degradation [331].
← TR	YAP	*YAP* is a direct transcriptional inducer of *Cyclin D* [332].
⊣ IBind	p15	*p15* is a cyclin-dependent kinase inhibitor of *cdk4 / cdk6*. As it displaces *Cyclin D*, it blocks G1/S progression [31].
⊣ IBind	p21	$p21^{Cip1}$ is a Cyclin Dependent kinase inhibitor which binds to and blocks the activity of *Cdk2*, *Cdk3*, *Cdk4* and *Cdk6* kinases [333] and thus inhibits *CyclinD1/Cdk4,6* [334].
⊣ TR	pRB	*E2F1*'s ability to induce *Cyclin D1* is blocked by active (hypo-phosphorylated) *RB* protein [335].
← TR	Myc	Extracellular growth signals activate the MAPK pathway, leading to transcriptional activation of *Cyclin D1* by *Myc* [336, 337]. *Myc* overexpression leads to rapid *Cyclin D1* induction and subsequent cell cycle entry [338], while its absence halves *Cyclin D1* levels [339]. In addition, *Myc* induces *Cdk4*, aiding the assembly of active *Cyclin D1 / Cdk4,6* complexes [339, 340].
← Per	CyclinD1	In order to take into account both production and stability of *Cyclin D1*, we assumed that the presence of active *CyclinD/Cdk2,4* complexes renders transcriptional maintenance of their levels easier.
← TR	E2F1	The *Cyclin D1* promoter is bound by *E2F* factors including *E2F1* [341], and *E2F1* overexpression can increase *Cyclin D1* (though its effects are context-dependent, as *E2F1* overexpression can also lead to apoptosis) [341]. Dominant negative *E2F1* overexpression results in a 2-3 fold decrease in *Cyclin D* expression and *Cyclin D/Cdk4,6* activity [342].
⊣ P	CHK1	During replication, checkpoint kinases such as *CHK1* (active during normal DNA synthesis) suppress *Cyclin D1* [343], which has a very short half-life (∼ 24 min) [344].

Table S1n: Restriction_SW module

E2F1	**E2F1** = (not((**CAD** or **CyclinA**) or **pRB**)) and ((**YAP** and (**E2F1** or **Myc**)) or (**E2F1** and **Myc**))		
	TF	In the absence of both *CyclinA* and *pRB*, *E2F1* transcription can be induced by *YAP* and *Myc* or maintained by active *E2F1*. *CAD* deactivates *E2F1* as it destroys the cell's DNA.	
	← TR	YAP	*YAP* is a direct transcriptional inducer of *E2F1* [345].
	⊣ TR	pRB	*RB* binds to *E2F/DP1* complexes and switches their DNA binding activity from activation to repression [335, 346].
	← TR	Myc	*Myc* is required for growth-factor mediated induction of *E2F1* [347, 339]. It binds to and remodels the *E2F1* promoter, facilitating *E2F1* transcription [330]. In addition, *Myc* augments protein expression of *E2F1* [348]. Single-cell experiments show that *Myc* is a critical modulator of the amplitude of *E2F* activation [349].
	← TR	E2F1	*E2F1* binds to its own promoter and up regulates transcription (as long as *Cyclin D/E* activity blocks *RB-E2F1* binding) [350].
	⊣ P	CyclinA	The phosphorylation of the *E2F1*-binding *DP-1* protein by *Cyclin A*, which binds directly to *E2F-1* (as well as *E2F-2,3*) downregulates *E2F1* transcriptional activity in S phase [351, 352, 353].
	⊣ Deg	CAD	This link from Caspase-activated DNase (*CAD*) to *E2F1* ensures that apoptotic cells settle into an *E2F1*-negative attractor regardless of their initial state. The rationale for this is that *E2F1* cannot maintain its activity if DNA is fragmented.
CyclinE	**CyclinE** = (**Hif1a_basal** or **CyclinE**) and **E2F1** and **Cdc6** and **Pre_RC** and not(**pRB** or **p27Kip1** or **CHK1** or **Casp3**)		
	PC	In our model, the ON state of *Cyclin E* represents active *Cyclin E/Cdk2* complexes. Thus, its full activation requires transcription via *E2F1* not blocked by active *pRB*, binding to *Cdc6* and *pre-RC* complexes, and the absence of its inhibitors $p27^{Kip1}$, *CHK1* and *Caspase 3*. In additon, we assume that moderate *HIF-1α* accumulation seen in G1 is required for the mitochodrial ATP boost that aids *Cyclin E* activation and S-phase entry[354, 4]. Once active, we assume that *CyclinE* can maintain its activity without the ongoing presence of *HIF-1α*.	
	⊣ TR	pRB	*Cyclin E* transcription by *E2F1* requires the absence of active, un-phosphorylated *RB* [353].
	⊣ IBind	p27Kip1	*p27Kip1* binds to and prevents the activation of *Cyclin E/Cdk2* complexes [298].
	← TR	E2F1	*E2F1* is a potent transcriptional activator of *Cyclin E* [355].
	← Compl	Cdc6	Chromatin association and full activation of *Cyclin E/Cdk2* requires *Cdc6* [356].

Table S1n: Restriction_SW module

← Compl	Pre_RC	At the G1/S transition, *Cyclin E* is loaded onto chromatin by *pre-RC* complexes (*Cdc6* and *Cdt1* binding), where it is required for *MCM2* loading, origin firing and the start of DNA synthesis [357]. In addition, activation of its partner *Cdk2* by *Cdc6* is contingent on this localization [356].
⊢ P	CHK1	*Chk1* activation during normal S-phase progression keeps *Cdk2* activity in a physiological range by binding to both *Cdk2* and *Cdc25A*, aiding the loss of *Cyclin E/Cdk1* activity [358].
⊢ Lysis	Casp3	*Caspase 3* cleaves and deactivates *Cyclin E*, which is then rapidly degraded [359].
← Ind	Hif1a_basal	*HIF-1α* is a transcriptional inducer of nealry every step of glycolysis, including *GLUT1* to facilitate increased glucose import [360]. Transient *HIF-1α* stabilization during late G1 is required for the boost of ATP production that initiates *Cyclin E* activation and replication [354, 4].
← Lysis	CyclinE	We assume that once *Cyclin E* is activated with the aid of high levels of ATP, it then remains active to continue S-phase progression without an ongoing need for *HIF-1α* activity.

Table S1o: Origin_Licensing module

Target Node	Node Gate<br>Node Type<br>Link Type	Node Description<br>Input Node	Link Description
ORC	**ORC** = **E2F1** or ((**Pre_RC** and **Cdt1**) and **Cdc6**)		
	PC	*ORC* proteins can bind at origins of replication when transcribed by *E2F1* or as part of a fully assembled and licensed *Pre-RC* complex (including active *Cdc6* and *Cdt1*).	
	← TR	E2F1	Expression of the *ORC1* gene is regulated by *E2F1* [361].
	← Compl	Cdc6	Availability of stable (unphosphorylated) *Cdc6* in the *Pre-RC* is necessary for the maintenance of licensed origins [362].
	← Compl	Cdt1	Active (unphosphorylated and not geminin-bound) *Cdt1* bound to the *Pre-RC* is necessary for the maintenance of licensed origins [362].
	← Compl	Pre_RC	Licensed but not yet fired replication complexes (*Pre-RC*s containing *ORC*, *Cdc6*, *Cdt1* and inactive *MCM*s) remain stable at sites of replication origin until fired by the activation of the *MCM* helicase [362].
Cdc6	**Cdc6** = ((not **Casp3**) and (not(**f4N_DNA** and **CyclinA**))) and (((**E2F1** and **ORC**) and (not **Plk1**)) or (((**Pre_RC** and **ORC**) and **Cdc6**) and **Cdt1**))		

Table S1o: Origin_Licensing module

	Prot	In our model the *Cdc6* node represents nuclear, chromatin-bound *Cdc6*. Thus, the node is only active during the assembly of pre-replication complexes, or their ongoing presence during DNA replication. *Cdc6* is ON in the absence of *Caspase 3* or *CyclinA / Cdk2* phosphorylation of *Cdc6* in all origins required for the completion of DNA replication (thus, its inhibition by *Cyclin A* also requires 4N DNA). In addition, active *Cdc6* requires either transcription by *E2F1* and recruitment by origin-bound *ORC* proteins in the absence of mitotic *Plk1* or maintenance of *Pre-RCs* by the presence of all of its components.	
	← TR	E2F1	Transcription of Cdc6 is directly induced by E2F1 [363].
	← Compl	ORC	*ORC* recruits *Cdc6* to origins of replication [362].
	← Per	Cdc6	Stable (unphosphorylated) *Cdc6* in the *Pre-RC* is necessary for the maintenance of licensed origins [362].
	← Compl	Cdt1	Active (unphosphorylated and not geminin-bound) *Cdt1* bound to the *Pre-RC* is necessary for the maintenance of licensed origins [362].
	← Compl	Pre_RC	Licensed but not yet fired replication complexes (*Pre-RC*s containing *ORC*, *Cdc6*, *Cdt1* and inactive *MCM*s) remain stable and *Cdc6*-bound until fired by the activation of the *MCM* helicase [362].
	⊢ P	Plk1	*Plk1* binds, phosphorylated and strongly recruits Cdc6 to the spindle pole during metaphase, then to the central spindle in anaphase, leading to its exclusion from chromosomes until telophase, when the majority of *Plk1* is degraded by APC/C^{Cdh1} [364].
	⊢ P	CyclinA	Phosphorylation of *CDC6* by *Cyclin A/Cdk2* during DNA replication leads to its re-localization to the cytoplasm [365].
	⊢ Ind	f4N_DNA	In our model, full deactivation of Cdc6 represents the firing of all ORCs as DNA replication is completed. Thus *Cyclin A*'s inhibitory action takes full effect once the cell reaches 4N DNA content [365].
	⊢ Lysis	Casp3	*Caspase 3* cleaves and deactivates *Cdc6* [366].
Cdt1	**Cdt1** = ((((not **geminin**) and **ORC**) and **Cdc6**) and (not((**CyclinE** and **CyclinA**) and **Cdc25A**))) and ((**Pre_RC** and (**E2F1** or **Myc**)) or (**E2F1** and (**Myc** or (not **pRB**))))		
	Prot	Replication-origin bound *Cdt1* requires the absence of *geminin*, the presence of origin-bound *ORC* and *Cdc6*, and the absence of sustained *Cdk2* activity responsible for the firing of all origins during DNA synthesis (modeled as simultaneous *Cyclin E*, *Cyclin A* and *Cdc25A* activity). Bound into a licensed pre-replication complex (*Pre-RC*), *Cdt1* remains stable as long as it is transcribed by *E2F1* [367] or *Myc* [368] (this guarantees that *Pre-RC* complexes cannot persist indefinitely in the absence of de novo transcription). Alternatively, it can be turned on by *E2F1*, aided by *Myc* or the absence of *RB*, and *FoxO3* in cells with 4N DNA.	

Table S1o: Origin_Licensing module

	⊣ TR	pRB	*E2F1*-mediated transcription of *Cdt1* is blocked by hypophosphorylated (active) *pRB* [367].
	← TR	Myc	*Cdt1* is a direct transcriptional target of the *Myc-Max* complex [368], ensuring its availability for *Pre-RC* formation and maintenance.
	← TR	E2F1	*Cdt1* is a direct transcriptional target of *E2F1* [367], ensuring its availability for *Pre-RC* formation and maintenance.
	⊣ P	CyclinE	Sustained *Cdk2* activity during S-phase (modeled as simultaneous *Cyclin E*, *Cyclin A* and *Cdc25A* activity) is responsible for the firing of all origins required to complete DNA synthesis; it also leads to the phosphorylation and proteasomal degradation of *Cdt1* [362].
	← Compl	ORC	*ORC*-bound origin of replication sites are the point of pre-replication complex assembly, where *Cdt1* is recruited by *ORC*-bound *Cdc6* [362].
	← Compl	Cdc6	*ORC*-bound *Cdc6* recruits *Cdt1* to *pre-RC* complexes [362].
	← Compl	Pre_RC	Licensed but not yet fired replication complexes (*Pre-RC*) remain stable until fired during DNA replication [362].
	⊣ IBind	geminin	*Geminin* binds to *Cdt1* at pre-replication complexes, where it blocks *Cdt1* binding to DNA, sequestering it away from *Pre-RC*s [369].
	⊣ P	Cdc25A	Sustained *Cdk2* activity leads to phosphorylation and degradation of *Cdt1* [362].
	⊣ P	CyclinA	Sustained *Cdk2* activity leads to phosphorylation and degradation of *Cdt1* [362].
Pre_RC	**Pre_RC** = ((**ORC** and **Cdc6**) and **Cdt1**) and (not(**Replication** and **f4N_DNA**))		
	PC		*Pre-RC* complexes assemble when *ORC*, *Cdc6*, and *Cdt1* are all bound to sites of replication origin along the DNA. The node denoting their licensing turns OFF at the moment of transition from ongoing *Replication* to *f4N_DNA* (it is blocked in the one time-point when both of these nodes are ON).
	← Compl	ORC	*Pre-RC* complexes assemble when *ORC*, *Cdc6*, and *Cdt1* are all bound to sites of replication origin along the DNA [362].
	← Compl	Cdc6	*Pre-RC* complexes assemble when *ORC*, *Cdc6*, and *Cdt1* are all bound to sites of replication origin along the DNA [362].
	← Compl	Cdt1	*Pre-RC* complexes assemble when *ORC*, *Cdc6*, and *Cdt1* are all bound to sites of replication origin along the DNA, leading to the recruitment of the *MCM* helicase [362].
	⊣ Unbind	Replication	*Pre-RC*s fire and fall apart during DNA replication [362].
	⊣ Ind	f4N_DNA	In our model the *Pre-RC* node turns OFF when *Replication* is completed, marked by the time-point when both *Replication* and *f4N_DNA* are ON.

Table S1o: Origin_Licensing module

geminin	**geminin** = (**E2F1** and (not **Cdh1**)) and (not(**pAPC** and **Cdc20**))		
	Prot	*Geminin* is present when transcribed by *E2F1* and not targeted for degradation by APC/C^{Cdh1} or APC/C^{Cdc20} [370].	
	← TR	E2F1	*Geminin* is a direct transcriptional target of *E2F1* [367].
	⊢ Ubiq	pAPC	*Geminin* is a target of APC/C^{Cdc20} at the metaphase/ anaphase transition [370].
	⊢ Ubiq	Cdc20	*Geminin* is a target of APC/C^{Cdc20} at the metaphase/ anaphase transition [370].
	⊢ Ubiq	Cdh1	*Geminin* is a target of APC/C^{Cdh1} ubiquitin ligase [371].

Table S1p: Phase_SW module

Target Node	Node Gate<br>Node Type<br>Link Type	 Node Description<br>Input Node	 <br>Link Description
CyclinA _mRNA	**CyclinA_mRNA** = (not **CAD**) and ((**E2F1** and (not **pRB**)) or **FoxM1**)		
	mRNA	In non-apoptotic cells (no *CAD*), *Cyclin A* is transcribed by *E2F1* in the absence of active *RB* or by *FoxM1*.	
	⊢ TR	pRB	Active *RB* blocks *E2F1*'s ability to transcribe *Cyclin A* [372].
	← TR	E2F1	*Cyclin A* is transcriptionally activated by *E2F* factors [353].
	← TR	FoxM1	Depletion of *FoxM1* results in reduced *Cyclin A2* expression (it is not clear whether *FoxM1* is a direct transcriptional inducer of *Cyclin A*) [373, 374, 375, 376].
	⊢ Ind	CAD	This link from Caspase-activated DNase (*CAD*) to *Cyclin A* mRNA ensures that apoptotic cells settle into a G0-like attractor regardless of their initial state. The rationale for this is that no mRNA synthesis can be maintained if DNA is fragmented (we only use these links from *CAD* if needed).
Emi1	**Emi1** = ((**E2F1** or (not **pRB**)) or (not **p21**)) and (not(((**Plk1** and **CyclinB**) and **Cdk1**) and (**U_Kinetochores** or **A_Kinetochores**)))		
	Prot	Our model allows the sustained presence of *Emi1* protein when it is either actively transcribed by *E2F1* [377, 378] or lacks joint inhibition by *pRB* [378] and *p21* [379]. Degradation of *Emi1* is mediated by *Plk1* and *CyclinB/Cdk1* complexes; initiation of this degradation requires at least temporary co-localization of *Emi1* with *Plk1* at mitotic spindle poles [380].	

Table S1p: Phase_SW module

	$\vdash$ Ind	p21	*p21* activation during DNA damage lead to a substantial decrease of *Emi1* levels, not observed in *p21*-null cells [379].
	$\vdash$ TR	pRB	Active retinoblastoma protein can block *Emi1* transcription mediated by *E2F1* [378].
	$\leftarrow$ TR	E2F1	*Emi1* is a direct transactional target of *E2F1* [377, 378].
	$\vdash$ P	Plk1	*Plk1* phosphorylates *Emi1* at mitotic spindle poles, stimulating its $\beta TrCP$ binding and ubiquitination [380].
	$\vdash$ Ind	CyclinB	*Cyclin B/Cdk1* enhances the ability of *Plk1* to mediate *Emi1* destruction [380].
	$\vdash$ Ind	Cdk1	*Cyclin B/Cdk1* enhances the ability of *Plk1* to mediate *Emi1* destruction [380].
	$\vdash$ Ind	U _Kinetochores	As *Plk1*-mediated phosphorylation of *Emi1* occurs at mitotic spindle poles, our model requires ongoing mitosis for this interaction [380].
	$\vdash$ Ind	A _Kinetochores	*Plk1* phosphorylates *Emi1* at mitotic spindle poles, stimulating its $\beta TrCP$ binding and ubiquitination [380].
FoxM1	**FoxM1** = (((**Myc** or **YAP**) and **CyclinE**) or ((**CyclinA** and **Cdc25A**) and **Cdc25B**)) or ((**Plk1** and **CyclinB**) and **Cdk1**)		
	TF		In our model, *FoxM1* activity requires increased expression by *Myc* [381] or *YAP* [332] and activating phosphorylation by *Cyclin E/Cdk2*. Alternatively, *FoxM1* activity can be sustained by potent *Cdk2 / Cdk1* activity in G2 (supported by *Cdc25A* or *Cdc25B*), or a serial phosphorylation by *Cyclin B/Cdk1* and *Plk1* during mitosis.
	$\leftarrow$ TR	YAP	*FoxM1* is a direct transcriptional target of *YAP* [332].
	$\leftarrow$ TR	Myc	*FoxM1* is a direct transcriptional target of *c-Myc* [381].
	$\leftarrow$ P	CyclinE	*Cyclin E/Cdk2* complexes bind and phosphorylate FoxM1, potently inducing its transcriptional activity, which starts during S-phase [382].
	$\leftarrow$ Compl	Cdc25A	Active *Cdc25A* binds to and enhances the transcriptional activity of *FoxM1*, potentually by bridging FoxM1 and active cyclin-*Cdk2* complexes [383].
	$\leftarrow$ Ind	Cdc25B	*Cdc25B* overexpression can increase *FoxM1*-dependent transcription, likely via aiding *Cdk1* activity [384].
	$\leftarrow$ P	Plk1	*Plk1* binds and phosphorylates *FoxM1*, which activates *FoxM1*-mediated transcription in early mitosis [385].
	$\leftarrow$ P	CyclinA	In addition to *Cyclin E/Cdk2*, *Cyclin A/Cdk2* complexes can also keep *FoxM1* transcriptionally active by phosphorylating its autoinhibitory N-terminal region [386].

Table S1p: Phase_SW module

	$\leftarrow$ P	CyclinB	*FoxM1* binds *Plk1*, and phosphorylation of two key residues at this binding domain by *Cyclin B/Cdk1* primes it for *Plk1* binding [385].
	$\leftarrow$ P	Cdk1	*FoxM1* binds *Plk1*, and phosphorylation of two key residues at this binding domain by *Cyclin B/Cdk1* primes it for *Plk1* binding [385].
Cdc25A	**Cdc25A** = (((**FoxM1** and **E2F1**) and (not **pRB**)) or ((not **Cdh1**) and (**FoxM1** or (**E2F1** and (not **pRB**))))) and ((((not(**GSK3** or **CHK1**)) or **CyclinE**) or **CyclinA**) or (**CyclinB** and **Cdk1**))		
	Ph		As the precise combinatorial regulation of *Cdc25A* throughput the cell cycle is unknown, our model assumes that accumulation of the *Cdc25A* protein requires transcriptional activation by both *E2F1* in the absence of *pRB*, and *FoxM1* to override destruction by APC/C^{Cdh1}. Alternatively, one of the two transcription factors can drive *Cdc25A* accumulation in the absence of APC/C^{Cdh1}. In addition, stabilization of *Cdc25A* either requires the absence of *GSK3β* and *CHK1* (both of which promote its degradation), or stabilization by *Cdk* activity.
	$\dashv$ P	GSK3	*GSK3β* phosphorylates *Cdc25A*, promoting its proteolysis [387].
	$\dashv$ TR	pRB	Active (hypo-phosphorylated) *pRB* blocks *E2F1*'s ability to drive *Cdc25A* transcription [388, 389].
	$\leftarrow$ TR	E2F1	*E2F1* is a direct transcriptional inducer of *Cdc25A* [388].
	$\leftarrow$ P	CyclinE	*Cdc25A* protein levels are stabilized during S-phase by *CyclinE/Cdk2*-dependent phosphorylation [390].
	$\leftarrow$ TR	FoxM1	*FoxM1* is a direct transcriptional inducer of *Cdc25A* [383].
	$\leftarrow$ P	CyclinA	*Cdc25A* protein levels are stabilized during S and G2 by *Cdk2*-dependent phosphorylation. *Cdk2* first partners with *Cyclin E* [390], then continues to stabilize *Cdc25A* past the point of *Cyclin E* expression by partnering with *Cyclin A* [391].
	$\leftarrow$ P	CyclinB	During mitosis, *Cdc25A* is stabilizes by *Cyclin B/Cdk1* phosphorylation, which protects it from the proteasome [392].
	$\leftarrow$ P	Cdk1	During mitosis, *Cdc25A* is stabilizes by *Cyclin B/Cdk1* phosphorylation, which protects it from the proteasome [392].
	$\dashv$ Deg	Cdh1	The APC/C^{Cdh1} complex degrades *Cdc25A* at mitotic exit [393, 394].
	$\dashv$ P	CHK1	*CHK1* phosphorylates *Cdc25A*, promoting its proteolysis and inhibiting its interaction with *Cyclin B/Cdk1* [395].
CyclinA	**CyclinA** = (**CyclinA_mRNA** and (not **pAPC**)) and ((**Cdc25A** and ((not **Cdh1**) or **Emi1**)) or (**CyclinA** and (((not **Cdh1**) and (**Emi1** or (not **UbcH10**))) or (**Emi1** and (not **UbcH10**)))))		

Table S1p: Phase_SW module

	PC	*Cyclin A* activity requires transcription (*Cyclin A* mRNA) and the absence of degradation by phosphorylated (mitotic) *pAPC*. In addition, turning ON inactive *Cyclin A* requires activation of *Cdk2* by *Cdc25A* [396] and the absence / *Emi1*-mediated inhibition of APC/C^{Cdh1}. Once active, *Cyclin A* maintains its activity in the absence of overpowering influences driving its degradation. Namely, *Cyclin A* relies on either *Emi1* or the absence of *UbcH10* for its ability to keep inactive APC/C^{Cdh1} in check. To overpower active APC/C^{Cdh1}, *Cyclin A* requires both *Emi1* and no *UbcH10*. The precise combinatorial regulation of *Cyclin A* is not known; the above logic is consistent with *Cyclin A* activity pattern during cell cycle progression.	
	← TL	CyclinA _mRNA	Sustained availability of *Cyclin A* requires ongoing translation from *CyclinA* mRNA.
	← Compl	Emi1	*Emi1* binding to *Cdh1* is required to stabilize *Cyclin A* levels at the G1/S transition, allowing *Cyclin A/Cdk2* to block APC/C^{Cdh1} [397, 398, 399].
	← DP	Cdc25A	*Cdc25A* promotes active *Cyclin A/Cdk2* complex formation by removing inhibitory phosphorylation of *Cdk2* [396, 400].
	← Per	CyclinA	We assume that once activated, *Cyclin A/Cdk2,1* complexes can sustain their activity until *Cyclin A* is degraded.
	⊢ Deg	UbcH10	*Cyclin A* degradation by APC/C^{Cdh1} requires *UbcH10* [401].
	⊢ Deg	pAPC	*Cyclin A* is degraded by the APC/C^{Cdc20} in prometaphase (as soon as the APC/C components are phosphorylated by Cdk1) [402, 403], before the full activation of the complex at SAC passage [404]. In our model, this stage of mitotic APC/C^{Cdc20} activation is represented by *Cdk1*-phosphorylated *APC/C* (*pAPC*).
	⊢ Deg	Cdh1	*Cyclin A* is degraded by APC/C^{Cdh1} in the presence of the *UbcH10* protein [405, 401, 307].
Wee1	**Wee1** = (((not **Casp3**) and (**Replication** or **CHK1**)) and (not(**Cdk1** and **CyclinB**))) and (**CHK1** or (not((**Cdk1** and **CyclinA**) and **Plk1**)))		
	K	*Wee1* is active during *Replication*, unless its activity is blocked by *CyclinA/Cdk1* OR *CyclinB/Cdk1* [406].	
	⊢ P	Plk1	*Plk1* phosphorylation at S53 promotes *Wee1* degradation [407]. This event is primed by *Cdk1* phosphorylation of *Wee1* at S123 [407]. As the main partner of *Cdk1* in mitosis is *Cyclin B*, we assume that assistance from *Plk1* to block *Wee1* is more relevant when paired with *Cyclin A/Cdk1* complexes.
	⊢ P	CyclinA	*Cyclin A/Cdk1* is a strong inducer of *Wee1* phosphorylation and deactivation [406].
	⊢ P	CyclinB	*Cyclin B/Cdk1* is a strong inducer of *Wee1* phosphorylation and deactivation [406].

Table S1p: Phase_SW module

	⊢ P	Cdk1	The somatic *Wee1* protein is an order of magnitude more sensitive to *Cdk1* activity than *Cdc25C*. Thus, both *Cyclin A/Cdk1* and *Cyclin B/Cdk1* strongly induce *Wee1* phosphorylation and deactivation [406].
	← ComplProc	Replication	To model the sensitivity of *Wee1* activation to ongoing DNA synthesis even in the absence of damage, our model turns on *Wee1* immediately upon the start of DNA replication and maintains it until both Replication and the checkpoint kinase *Chk1* is OFF [408]. In addition, *Wee1* activity has been implicated in maintaining normal replication fork procession, linking its activity directly to ongoing replication [409].
	← P	CHK1	During DNA replication *Wee1* is activated by the checkpoint kinase *CHK1* [408].
	⊢ Lysis	Casp3	*Caspase 3* cleaves and deactivates *Wee1* [410].
UbcH10	**UbcH10** = (not **Cdh1**) or (**UbcH10** and ((**Cdc20** or **CyclinA**) or **CyclinB**))		
	UbL		The ubiquitin-conjugating enzyme (E2) *UbcH10* is active in the absence of *Cdh1*. Alternatively, active *UbcH10* is maintained in the presence of *Cdh1* when some of its targets are present: *Cdc20* OR *CyclinA* OR *CyclinB* [401].
	← PBind	CyclinA	The presence of APC/C^{Cdh1} substrates, including *Cyclin A*, inhibit the autoubiquitination of *UbcH10* but not its function, thus preserving APC activity [401].
	← PBind	CyclinB	The presence of APC/C^{Cdh1} substrates, including *CyclinB*, inhibit the autoubiquitination of *UbcH10* but not its function, thus preserving APC activity [401].
	← Per	UbcH10	Active *UbcH10* cannot be autoubiquitinated in the presence of APC/C^{Cdh1} substrates and thus remains active [401].
	← PBind	Cdc20	The presence of APC/C^{Cdh1} substrates, including *Cdc20*, inhibit the autoubiquitination of *UbcH10* but not its function, thus preserving APC activity [401].
	⊢ Deg	Cdh1	*UbcH10* is degraded by APC/C^{Cdh1}.
CyclinB	**CyclinB** = (**FoxM1** or (**FoxO3** and **CyclinB**)) and not(**Cdh1** or (**pAPC** and **Cdc20**))		
	PC		*Cyclin B* node is ON when the concentration of *Cyclin B* proteins is high (does not represent the activity of *CyclinB/Cdk1* complexes). This occurs when *Cyclin B* is transcribed by *FoxM1*, maintained by *FoxO3* transcription, and not undergoing *APC*-mediated degradation by APC/C^{Cdc20} or APC/C^{Cdh1}.
	← TR	FoxO3	*FoxO3* is a direct transcriptional regulator of *Cyclin B*; its activation in G2 helps increase/maintain *Cyclin B* levels [411].
	← TR	FoxM1	*FoxM1* is a direct transcriptional regulator of *Cyclin B1* [376, 412].
	← Per	CyclinB	Here we assume that FoxO3 alone can only maintain, but not independently induce *Cyclin B1* expression.

Table S1p: Phase_SW module

	$\dashv$ Deg	pAPC	*Cyclin B* is degraded by APC/C^{Cdc20} [405].
	$\dashv$ Deg	Cdc20	*Cyclin B* is degraded by APC/C^{Cdc20} [405].
	$\dashv$ Deg	Cdh1	*Cyclin B* is degraded by APC/C^{Cdh1} [405].
Cdc25B	**Cdc25B** = **FoxM1** and **f4N_DNA**		
	Ph		*Cdc25B* activation requires transcription by *FoxM1*, centrosomal localization, and activation by *Aurora A* kinase on replicated centrosomes.
	$\leftarrow$ TR	FoxM1	*FoxM1* is an essential inducer or *Cdc25B* [413].
	$\leftarrow$ Loc	f4N_DNA	*Cdc25B* is localized at centrosomes, where it is activated by *Aurora A* kinase [414]. As *Aurora A* itself is only recruited to duplicated, centrosomes before their separation [415], *Cdc25B* activation requires duplicated centrosomes. As our model does not directly account for centrosome dynamics, we account for this by requiring the completion of S-phase (4N DNA).
Plk1	**Plk1** = ((not **Cdh1**) and (**FoxM1** or **Plk1_H**)) and ((**CyclinB** and **Cdk1**) or ((**CyclinA** and (not **Wee1**)) and **Cdc25A**))		
	K		*Plk1* activity requires the absence of APC/C^{Cdh1}, transcription by *FoxM1*, or high *Plk1* levels transcribed earlier by both *FoxM1* and *FoxO3* (see *Plk1_H* below) [411]. In addition, *Plk1* activation requires phosphorylation by either *CyclinB/Cdk1* during mitosis or *Cyclin A/Cdk2* (aided by lack of *Wee1* and *Cdc25A*) at the G2/M boundary [416].
	$\leftarrow$ TR	FoxM1	*Plk1* is a direct transcriptional target of *FoxM1* [385].
	$\leftarrow$ Ind	Cdc25A	As we do not include a separate *Cdk2* node in our model, strong *Cyclin A/Cdk2* activity requires ongoing dephosphorylation of *Cdk2* by *Cdc25A* [417].
	$\dashv$ Ind	Wee1	*Cyclin A*-mediated induction of *Plk1* is blocked by *Wee1* kinase, which specifically inhibits *Cdk2* activity [416].
	$\leftarrow$ P	CyclinA	*Plk1* activation at the G2/M boundary, before *Cdk1/Cuclin B* complexes are activated, requires active *Cyclin A/Cdk* [416].
	$\leftarrow$ P	CyclinB	*Plk1* is activated by *Cyclin B/Cdk1* phosphorylation [418, 419, 420].
	$\leftarrow$ P	Cdk1	*Plk1* is activated by *Cyclin B/Cdk1* phosphorylation [418, 419, 420].
	$\dashv$ Ubiq	Cdh1	The majority of *Plk1* is degraded in anaphase by the APC/C^{Cdh1} complex [421].

Table S1p: Phase_SW module

	← Per	Plk1_H	Our model tracks the accumulauton of high-enough levels of *Plk1* to survive APC/C^{Cdh1} mediated destruction into telophase via the *Plk1_H* node. Its ON state represents strong prior *Plk1* activation. Thus, it sustains the *Plk1* node in the absence of *FoxM1*-mediated transcription until *Plk1_H* itself is lost as *Plk1* levels fall.
Cdc25C	**Cdc25C** = (**f4N_DNA** and **Plk1**) and ((**Cdc25B** and (not **CHK1**)) or (**CyclinB** and **Cdk1**))		
	Ph		In our model, *Cdc25C* is active in cells with replicated DNA (see *f4N_DNA* → *Cdc25C* link). Its activation is initiated by a small, initially cytoplasmic pool of *Cyclin B/Cdk1* activated by *Cdc25B* (not directly represented in our model) and further increased by *Cdc25B* itself, which translocates to the nucleus with the aid of *Plk1*. During mitosis, *Plk1* potentiates the ability of *Cyclin B/Cdk1* to maintain *Cdc25C* activity.
	← Ind	Cdc25B	*CDC25B* starts the cascade leading to mitotic entry by activating a small centrosomal pool of *Cyclin B/Cdk1*, leading to their nuclear translocation where they trigger the activation of *Cdc25C* and eventually the larger nuclear *Cyclin B/Cdk1* pool [422, 423, 424].
	← P	Plk1	In addition, *Plk1* induces nuclear transport of *CDC25B*, where it contributes to the initiation of *Cdk1* activity [425]. During mitosis, *Plk1* helps maintain strong *Cdc25C* activation by phosphorylating it on the same site as *Cyclin B/Cdk1* [426], as indicated by the profound decrease of Cdc25C activity in Plk1-inhibited mitotic cells [419, 427].
	← P	CyclinB	*Cyclin B/Cdk1* complexes are potent activators of *Cdc25C*, creating positive feedback that causes switch-like mitotic entry [428, 429].
	← P	Cdk1	*Cyclin B/Cdk1* complexes are potent activators of *Cdc25C*, creating positive feedback that causes switch-like mitotic entry [428, 429].
	⊢ P	CHK1	*CHK1* phosphorylates *Cdc25C*, leading to its nuclear exclusion, loss of access to its main target, *Cdk1* [430]. In addition, *CHK1* blocks the ability of *Cdc25B* to activate *Cdc25C* at the centrosomes by phosphorylating it and blocking its *Cdk1* activity [431, 432].
	← Ind	f4N_DNA	The nature and localization of the signals responsible for the onset and maintenance of *Cdc25C* activity require replicated DNA (*f4N_DNA*) [425, 424]. Namely, *Cdc25C* is initially activated by a small pool of *Cyclin B/Cdk1* (below the ON-threshold of *Cdk1* in our model) which starts our at the replicated centrosome. Moreover, the pool of mitotic *Cdc25C* co-localized with active *Chk1/Cyclin B* is found on condensed chromosomes, again requiring the presence of *f4N_DNA* [430].
Cdk1	**Cdk1** = (**CyclinB** and **Cdc25C**) and ((not **CHK1**) or ((not **Wee1**) and **Cdk1**))		

Table S1p: Phase_SW module

	K	Full *Cdk1* kinase activation requires its binding partner *Cyclin B* and the *Cdc25C* phosphatase, which maintains *Cdk1* in an active dephosphorylated state. *Cdk1* is inhibited by the checkpoint kinase *CHK1*, unless it is already full active and *Wee1* kinase is inhibited.	
	$\vdash$ P	Wee1	*Wee1* is a nuclear protein that ensures the completion of DNA replication prior to mitosis by blocking nuclear *Cdk1* activation [433].
	$\leftarrow$ DP	Cdc25C	*Cdk1* is subject to inhibitory phosphorylation by *Wee1* or *Myt1*, and its dephosphorylation is carried out by activated *Cdc25C* [428, 434, 429].
	$\leftarrow$ Compl	CyclinB	Full kinase activation of *Cdk1* in our model requires it to complex with *Cyclin B* [434].
	$\leftarrow$ Per	Cdk1	We assume that the presence of fully activated, nuclear *Cdk1* is able to overcome the effect of active *Wee1*, given that *Wee1* is very sensitive to *Cdk1*-mediated inhibitory phosphorylation [406].
	$\vdash$ P	CHK1	In the absence of *CHK1* kinase, a small cytosolic (centrosomal) pool of *Cyclin B/Cdk1* can be activated by *Cdc25B*, the nuclear translocation of which can trigger a positive feedback loop that activates the full *Cdk1* pool (assuming nuclear *Wee1* is also inactive). Thus, *CHK1* can maintain the OFF state of inactive *Cdk1* [416].
pAPC	**pAPC** = (((**CyclinB** and **Cdk1**) and **Plk1**) or ((**CyclinB** and **Cdk1**) and **pAPC**)) or (**pAPC** and **Cdc20**)		
	PC	In line with evidence that *Plk1* can aid full activation of *APC/C*, but *Cdk1* appears to be the more potent inducer, our model requires both *Cyclin B/Cdk1* and *Plk1* to activate *APC/C* from an OFF state, but only *Cdk1* activity to maintain it. In addition, ongoing phosphorylation of the functional *APC/C*^{Cdc20} complex is no longer required.	
	$\leftarrow$ P	Plk1	In addition to *Cyclin B/Cdk1* phosphorylation, full activation of the *APC/C*^{Cdc20} complex also requires the kinase activity of *Plk1* [435].
	$\leftarrow$ P	CyclinB	*CyclinB/Cdk1* activation triggers mitotic entry and promotes *APC/C*^{Cdc20} activity via APC/C subunit phosphorylation [436, 437].
	$\leftarrow$ P	Cdk1	*CyclinB/Cdk1* activation triggers mitotic entry and promotes *APC/C*^{Cdc20} activity via APC/C subunit phosphorylation [436, 437].
	$\leftarrow$ Per	pAPC	Activated *APC/C*^{Cdc20} initiates the Metaphase / Anaphase transition by degrading *Cyclin B* and securin [370]. Once active, *APC/C*^{Cdc20} no longer requires sustained *CyclinB/Cdk1* or *Plk1* phosphorylation.
	$\leftarrow$ Compl	Cdc20	Once active, *APC/C*^{Cdc20} no longer requires sustained *CyclinB/Cdk1* or *Plk1* phosphorylation.

Table S1p: Phase_SW module

Cdc20	**Cdc20** = ((**pAPC**and(not **Emi1**))and(not **Cdh1**))and((not **Mad2**)or((not **CyclinA**)and (not(**CyclinB** and **Cdk1**))))		
	Prot		In our model, APC/C^{Cdc20} complex formation is represented by the joint activity of *Cdc20* and phosphorylated *APC/C* (*pAPC*). *Cdc20* is thus ON in the presence of *pAPC* when both *Emi1* and *Cdh1* are absent (APC/C^{Cdh1} is represented by the *Cdh1* node, see below). In addition, *Cdc20* activity requires either the absence of *Mad2* at unattached kinetochores, or the absence of *Cdc20* phosphorylation by *Cyclin B/Cdk1* or by *Cyclin A/Cdk2* complexes to potentiate the interaction between *Mad2* and *Cdc20*, and *pAPC* is ON (present and phosphorylated) [438].
	⊢ IBind	Emi1	*Emi1* binds *Cdc20* and inhibits the ubiquitin ligase activity of APC/C^{Cdc20} [398].
	⊢ P	CyclinA	*Cyclin A/Cdk2* complexes phosphorylate *Cdc20* and inactivate the APC/C^{Cdc20} complex during S and G2 [439].
	⊢ P	CyclinB	*Cyclin B* partners with *Cdk1* to keep *Cdc20* phosphorylated, increasing its interaction with *Mad2* rather than *APC/C* [440].
	⊢ P	Cdk1	*Cdk1*-phosphorylated *Cdc20* interacts with *Mad2* rather than *APC/C*, resulting in a block on APC/C^{Cdc20} activation until completion of spindle assembly [438].
	← Compl	pAPC	*Cdc20* becomes active in early mitosis by binding to *APC/C*, an event that requires *Cyclin B/Cdk1*-mediated phosphorylation of several core *APC/C* subunits [394, 441].
	⊢ Deg	Cdh1	APC/C^{Cdh1} complexes degrade *Cdc20*, leading to a complete switch from APC/C^{Cdc20} to APC/C^{Cdh1} during mitotic exit [394, 442].
	⊢ IBind	Mad2	Eukaryotic cells do not separate their replicated genome until they pass the Spindle Assembly Checkpoint (SAC). Namely, all their chromosomes need to be are aligned with respect to the metaphase plane and the two copies of each chromosome need to be attached to opposite poles of the mitotic spindle [437]. This physical alignment is monitored via *Mad2*: kinetochores that remain unattached to microtubules catalyze the sequestration of *Cdc20* and thus inhibit APC/C^{Cdc20} [443, 444].
Cdh1	**Cdh1** = (not(**CyclinB** and **Cdk1**)) and (not(**CyclinA** and (**Emi1** or **Cdc25A**)))		
	PC		APC/C^{Cdh1} activity requires the absence of Cyclin Dependent kinase phosphorylation by *Cyclin B/Cdk1*, or *Cyclin A/Cdk2* aided by further inhibition of *Cdh1* by *Emi1*, or ongoing *Cdk2* activation by *Cdc25A* in the absence of *Emi1*.
	⊢ IBind	Emi1	*Emi1* blocks APC/C^{Cdh1} binding to its substrates [399], as well as its ability to add ubiquitin chains to them [445].

Table S1p: Phase_SW module

⊢ Ind	Cdc25A	As we do not include a separate *Cdk2* node in our model, strong *Cyclin A/Cdk2* activity capable of overriding *Cdh1* activity even in the presence of *Emi1* requires ongoing dephosphorylation of *Cdk2* by *Cdc25A* [428].
⊢ P	CyclinA	Active *Cyclin A/Cdk1,2* complexes phosphorylate *Cdh1* during S, G2 and early mitosis, impairing its interaction with *APC/C* until late stages of mitosis when *Cdk1/2* activity falls [394, 405].
⊢ P	CyclinB	*Cyclin B/Cdk1* phosphorylates *Cdh1* during mitosis, impairing its interaction with *APC/C* [394, 405].
⊢ P	Cdk1	*Cyclin B/Cdk1* phosphorylates *Cdh1* during mitosis, impairing its interaction with *APC/C* [394, 405].

Table S1q: Cell_Cycle_Process module

Target Node	Node Gate<br>Node Type<br>Link Type	Node Description<br>Input Node	Link Description
Replication	**Replication** = ((not **CAD**) and **Pre_RC**) and (((**E2F1** and **CyclinE**) and **Cdc25A**) or (((**Replication** and **CyclinA**) and **Cdc25A**) and (**E2F1** or (not **f4N_DNA**))))		
	Proc	The *Replication* node represents ongoing DNA synthesis. This requires a non-apoptotic cell, licensed pre-replication complexes (*Pre_RC*). The start of DNA synthesis requires *E2F1*-meditated transcription of the genes that help execute it, as well as the firing of the first round of replication origins by *Cyclin E/Cdk2*. Once ongoing, replication is sustained by *Cyclin A/Cdk2* and *Cdc25A*, aided by *E2F1* and terminated by completion of a full round of synthesis (*4N_DNA*).	
	← ComplProc	E2F1	In addition to *E2F1* target genes directly included in our model, *E2F1* transcribes an array of critical S-phase genes responsible for carrying out DNA synthesis (e.g, *POLA1*, *POLA2*, *MCM3*, *MCM5*, *MCM6*, *PCNA*, *TOP2A*, *RFC2*, *TK1*) [446, 447].
	← ComplProc	CyclinE	DNA replication is initiated by fully active *Cyclin E/Cdk2* [448].
	← ComplProc	Pre_RC	Ongoing DNA replication requires licensed replication origins, which fire throughout DNA synthesis [449].
	← ComplProc	Cdc25A	Active *Cdc25A* is required for onset as well as progression through S-phase [450, 451].
	← ComplProc	CyclinA	*Cyclin A/Cdk1* complexes regulate the origin firing program in mammalian cells and are required for the completion of DNA replication [451, 400].

Table S1q: Cell_Cycle_Process module

	← Per	Replication	Once ongoing, DNA synthesis continues in the presence of active *Cyclin A/Cdk2*, only ending when DNA content is doubled.
	⊢ ComplProc	f4N_DNA	Complete duplication of a cell's DNA, represented in our model by *f4N_DNA* = ON, marks the end of active *Replication*.
	⊢ ComplProc	CAD	Caspase-activated DNase (*CAD*) destroys DNA, preventing ongoing replication.
ATR	**ATR** = **Replication**		
	K		*ATR* accumulates at replication forks during unperturbed DNA synthesis [452].
	← Loc	Replication	*ATR* accumulates at replication forks during unperturbed DNA synthesis [452].
CHK1	**CHK1** = **ATR**		
	K		*ATR* kinase activates *CHK1* at replication forks, which not only blocks premature mitosis but also regulates the rate of origin firing by keeping *Cdc25* protein levels from increasing above their physiological range [452].
	← P	ATR	*ATR* kinase activates *CHK1* at replication forks (by phosphorylation of serines 317 and 345), which not only blocks premature mitosis but also regulates the rate of origin firing by keeping *Cdc25* protein levels from increasing above their physiological range [452].
f4N_DNA	**f4N_DNA** = (not **CAD**) and ((**Replication** and ((**Pre_RC** and **CyclinA**) or **f4N_DNA**)) or (**f4N_DNA** and (not **Cytokinesis**)))		
	MSt		4N DNA content in our model is reached via the completion of *Replication* (via the firing of the last round of replication origins by *Cyclin A/Cdk* complexes) and maintained in non-apoptotic cells the absence of a contractile ring driving cytokinesis.
	← ComplProc	Pre_RC	*Replication* can only complete DNA synthesis and produce double DNA content if the availability of licensed replication origins is not blocked [449].
	← ComplProc	CyclinA	*Cyclin A/Cdk1* complexes regulate the origin firing program in mammalian cells and are required for the completion of DNA replication [448, 400].
	← ComplProc	Replication	DNA content is doubled by the process of *Replication*.
	← Per	f4N_DNA	Once achieved, a cell's 4N DNA content is sustained up to the point of cytokinesis.
	⊢ ComplProc	Cytokinesis	The process of cytokinesis separates the replicated sister chromatids and resets the DNA content of each daughter cell to a diploid 2N.
	⊢ Deg	CAD	Caspase-activated DNase (*CAD*) destroys DNA, preventing maintenance of a double DNA content.

Table S1q: Cell_Cycle_Process module

U_Kinetochores	**U_Kinetochores** = ((**f4N_DNA** and (not **Cdh1**)) and (not **A_Kinetochores**)) and ((**CyclinB** and **Cdk1**) or **U_Kinetochores**)		
	MSt	The *U_Kinetochores* node in our model is on from the moment the nuclear envelope is dissolved in prometaphase and the mitotic spindle starts to form, until all kinetochores are properly attached. In addition to the presence of unattached kinetochores, *U_Kinetochores* = ON requires attached sister chromatics, the absence of APC/C^{Cdh1} activity. It is turned on my *Cyclin B/Cdk1* and remains on until the spindle is complete (or it is destroyed by APC/C^{Cdh1}).	
	← ComplProc	CyclinB	The start of mitotic spindle assembly is initiated by active *Cyclin B/Cdk1* [453].
	← ComplProc	Cdk1	The start of mitotic spindle assembly is initiated by active *Cyclin B/Cdk1* [453].
	⊢ ComplProc	Cdh1	Premature activation of APC/C^{Cdh1} destroys the incomplete spindle by triggering premature, aberrant anaphase. This occurs due to premature degradation of *APC/C* targets including *Securin* (responsible for keeping sister chromatids attached [454]), *Cyclin B*, *Cdc20*, and Aurora kinase A (*AURKA*) [455].
	← ComplProc	f4N_DNA	Metaphase requires replicated sister chromatids (*f4N_DNA*), held together by their kinetochores, face in opposing directions and can be attached to opposite poles of the mitotic spindle.
	← Per	U_Kinetochores	Once metaphase starts, the mitotic spindle remains incomplete as long as some of the kinetochores remain unattached.
	⊢ ComplProc	A_Kinetochores	In our model, the transition from unattached to all attached kinetochores (*U_Kinetochores* → *A_Kinetochores*) marks the completion of the mitotic spindle and Spindle Assembly Checkpoint (SAC) passage.
Mad2	**Mad2** = **U_Kinetochores** and (not **A_Kinetochores**)		
	Prot	Our model represents the SAC via the *Mad2* kinetochore-binding protein. *Mad2* is active as long as the cell has at least one unattached kinetochore and it is responsible for keeping *Cdc20* sequestered from *APC/C*. By keeping *APC* at bay until the spindle is complete, *Mad2* is required for the proper timing of anaphase [444].	
	← Compl	U_Kinetochores	The *Mad2* SAC protein is active and potent in the presence of even a single unattached kinetochore [444].
	⊢ ComplProc	A_Kinetochores	*Mad2* is inhibited by SAC passage, marked by the completion of the spindle and proper attachment of all kinetochore [444].
A_Kinetochores	**A_Kinetochores** = ((**f4N_DNA** and (not **Cdh1**)) and (not(**pAPC** and **Cdc20**))) and (**A_Kinetochores** or ((((**U_Kinetochores** and **Src**) and **Plk1**) and **CyclinB**) and **Cdk1**))		

Table S1q: Cell_Cycle_Process module

	MSt		The completed spindle, represented by the *A_Kinetochores* node, requires replicated and attached sister chromatids (*f4N_DNA*) and the absence of *APC/C* activity. It turns on when the process of spindle assembly (*U_Kinetochores*) is completed by active *Src*, active *Plk1* localized to unattached kinetochores in the presence of ongoing *Cyclin B/Cdk1* activity, and it remains on until anaphase (*APC/C* activation).
	← ComplProc	Src	*Src* promotes correct spindle orientation [456]. Moreover, absence of *c-Src* leads to severely reduced astral microtubules [457]. Finally, *Src*-mediated phosphorylation of the *Eg5* motor domain is required for the formation of a bipolar spindle and correct chromosome segregation [458].
	← ComplProc	Plk1	*Plk1* activity at unattached kinetochores is required for promoting their attachment [459]. In its absence, kinetochores remain unattached and cells eventually undergo mitotic catastrophe and apoptosis [460].
	← ComplProc	CyclinB	Ongoing *Cyclin B/Cdk1* at unattached kinetochores is necessary to keep *Plk1* active and allow the completion of mitosis [461].
	← ComplProc	Cdk1	Ongoing *Cyclin B/Cdk1* at unattached kinetochores is necessary to keep *Plk1* active and allow the completion of mitosis [461].
	⊣ Deg	pAPC	During normal mitosis, the completed spindle is pulled apart in response to APC/C^{Cdc20}-mediated degradation of *Securin*, which normally blocks *Separate* from severing the *Cohesin* rings keeping sister chromatids attached [454].
	⊣ Deg	Cdc20	During normal mitosis, the completed spindle is pulled apart in response to APC/C^{Cdc20}-mediated degradation of *Securin*, which normally blocks *Separate* from severing the *Cohesin* rings keeping sister chromatids attached [454].
	⊣ Deg	Cdh1	APC/C^{Cdh1} destroys the spindle by triggering anaphase via the degradation of *APC/C* targets, including *Securin* [455].
	← ComplProc	f4N_DNA	Completion of the mitotic spindle requires replicated and attached sister chromatids (*f4N_DNA*).
	← ComplProc	U_Kinetochores	The mitotic spindle is assembled gradually, as the number of unattached kinetochores gradually decreased by the formation of microtubule attachments.
	← Per	A_Kinetochores	Once assembled, separation of the mitotic spindle requires *APC/C* activity to promote the destruction of sister chromatid cohesion [454].
Plk1_H	**Plk1_H** = (**Plk1** and **FoxM1**) and ((**Plk1_H** or **FoxO3**) or **FoxO1**)		
	K		The ON state of *Plk1_H* encodes the short-lived memory of a sufficiently large active *Plk1* pool to temporarily survive *Plk1* destruction by APC/C^{Cdh1} [421], recruit *Ect2* to the central spindle, and thus aid the completion of cytokinesis [462]. Thus, *Plk1_H* requires ongoing *Plk1* activation and transcription by *FoxM1*, and either induction by *FoxO3* or *FoxO1*, or prior accumulation.

Table S1q: Cell_Cycle_Process module

	← TR	FoxO3	*Plk1* is a direct transcriptional target of *FoxO3*, but *Plk1* appears to be sufficiently induced in the absence of *FoxO* preteens to aid its G2/M and mitotic functions. In contrast, accumulation if a large enough *Plk1* pool to briefly outlast APC/C^{Cdh1} activation (modeled by the *Plk1_H* node), requires *FoxO* activity in G2 [411].
	← TR	FoxO1	In addition to *FoxO3*, *FoxO1* also binds the *Plk1* promoter, potentially aiding its accumulation during G2 [463].
	← TR	FoxM1	*Plk1* is a direct transcriptional target of *FoxM1*; loss of *FoxM1* severely reduces *Plk1* protein levels [375, 385].
	← Per	Plk1	Active mitotic *Plk1* is a prerequisite for the accumulation of the larger active *Plk1* pool denoted by *Plk1_H*.
	← Per	Plk1_H	Once accumulated, we assume that the *Plk1_H* pool of active *Plk1* remains stable in the absence of *FoxO*-mediated transcription. This is supported by negative feedback regulation of *FoxO* proteins by *Plk1* [78], indicating that ongoing high FoxO activity is likely not required for the maintenance of *Plk1_H*.
Ect2	**Ect2** = (((**f4N_DNA** and **Plk1_H**) and **Cdh1**) and (not **U_Kinetochores**)) and (not **A_Kinetochores**)		
	GEF		*Ect2* activaiton at the spindle midzone represents the step of cytokinesis in our model. Thus, *Ect2* requires *f4N_DNA*, high *Plk1* activity, as well as *Cdh1* for the assembly of a normal spindle midzone. Finally, *Ect2* cannot be recruited to the mid zone before anaphase is completed.
	← Ind	Cdh1	APC/C^{Cdh1}-mediated destruction of Aurora kinase is required for the assembly of a robust spindle midzone at anaphase and for the normal timing of cytokinesis [464].
	← Ind	f4N_DNA	Formation of a spindle midzone, where *Ect2* accumulates in preparation of cytokinesis requires recently separated sister chromatids (4N DNA content).
	⊢ ComplProc	U _Kinetochores	Formation of a spindle midzone requires the separation of sister chromatids; thus it cannot occur before anaphase.
	⊢ ComplProc	A _Kinetochores	Formation of a spindle midzone requires the separation of sister chromatids; thus it cannot occur before anaphase.
	← Ind	Plk1_H	*Plk1* activity in telophase (*Plk1_H*) is required for the recruitment of *Ect2* to the central spindle [421, 465].
Cytokinesis	**Cytokinesis** = (**Ect2** and **FAK**) and **Src**		
	Proc		In contrast to our previous model in [108] where *Ect2* recruitment to the central spindle marked the start of cytokinesis, in [109] we introduced a separate *Cytokinesis* node to mark cytokinesis and the subsequent resetting of daughter cell DNA content to 2N by a separate node. In addition to *Ect2* recruitment, completion of cytokinesis also requires ECM attachments able to activate *FAK* and *Src* kinases.

Table S1q: Cell_Cycle_Process module

	← ComplProc	FAK	Integrin-activated *FAK* and *Src* control cytokinetic abscission by decelerating *PLK1* degradation at aiding *CEP55* in recruiting abscission process proteins to the midbody [136, 466].
	← ComplProc	Src	Integrin-activated *FAK* and *Src* control cytokinetic abscission by decelerating *PLK1* degradation at aiding *CEP55* in recruiting abscission process proteins to the midbody [136, 466].
	← Loc	Ect2	At the start of cytokinesis, the *Ect2 RhoGEF* is recruited to the central spindle [462]. *Ect2* aids the accumulation of GTP-bound *RhoA* [467, 462] and the formation of the contractile ring.

Table S1r: TRAIL module

Target Node	Node Gate<br>Node Type<br>Link Type	Node Description<br>Input Node	Link Description
Trail	**Trail** = **Trail**		
	Env	The *Trail* node represents environmental availability of the *Trail* protein outside the cell.	
	← Env	Trail	The *Trail* input node remains on/off if set ON/OFF in the absence of *in silico* perturbation.

Table S1s: Apoptotic_SW module

Target Node	Node Gate<br>Node Type<br>Link Type	Node Description<br>Input Node	Link Description
DR4_5	**DR4_5** = **Trail**		
	Rec	The *DR4* and *DR5* death receptors, represented by the *DR4_5* node, are activated by extracellular *Trail* [468].	
	← Ligand	Trail	*DR4* and *DR5* death receptors are activated by extracellular *Trail* [468].
Casp8	**Casp8** = **DR4_5** or **Casp3**		
	PTase	*Pro-Caspase 8* may be cleaved independently by *DISC* (not directly reprsented; an adaptor protein for *DR4_5*) or *Caspase 3*.	
	← Compl	DR4_5	*Trail*-bound (active) *DR4* and *DR5* receptors trigger the assembly of the pro-apoptotic death-inducing signaling complex (*DISC*), which binds a cluster of *pro-Caspase 8* proteins and initiates their cleavage into active *Caspase 8* [469].

Table S1s: Apoptotic_SW module

	← Ind	Casp3	*Caspase 3* indirectly activates *Caspase 8* by cleaving *Caspase 6* [470], which, in turn, cleaves *Caspase 8* [471].
Casp2	**Casp2** = **Casp3** or ((**U_Kinetochores** and **Mad2**) and (not(**CyclinB** and **Cdk1**)))		
	PTase	*Pro-caspase 2* is cleaved and activated by *Caspase 3*, or by failed cytokinesis marked by the presence of unattached kinetochores, an active SAC, and the absence of active *Cyclin B/Cdk1* complexes to phosphorylate and inhibit *Caspase 2*.	
	⊢ P	CyclinB	*Cyclin B1/Cdk1* phosphorylate *caspase-2* at Ser 340, preventing its activation [472].
	⊢ P	Cdk1	*Cyclin B1/Cdk1* phosphorylate *caspase-2* at Ser 340, preventing its activation [472].
	← Ind	Mad2	A functional spindle assembly checkpoint is required for mitotic cell death upon prolonged mitotic arrest [473] or spindle damage [474].
	← Ind	U _Kinetochores	Although the precise molecular mechanism by which *Caspase 2* is activated during prolonged or stalled mitosis is unclear, its activation platform, the *PIDDosome*, has been localized to unattached kinetochores [475]. Even though a checkpoint protein keeps the *PIDDosome* unresponsive to DNA damage signals, the loss of protective *Cyclin B/Cdk1* phosphorylation only leads to *Caspase 2* activation in the presence of a partially assembled mitotic spindle, and requires active SAC.
	← Lysis	Casp3	*Caspase 2* is a target of *Caspase 3*, as its inhibition severely limits *Caspase 2* cleavage during apoptosis [476, 477].
MCL_1	**MCL_1** = (((not **Casp3**) and (not **Casp2**)) and ((not **GSK3**) or (**AKT_B** and (**ERK** or (not **E2F1**))))) and (not((**Cdk1** and **CyclinB**) and **U_Kinetochores**))		
	Prot	*Caspase 3* or *2*-mediated destruction of *MCL-1* must be absent for *MCL-1* to be ON. Avoiding degradation via textitGSK3 requires the *GSK3*-weakenjng presence of basal *AKT* activity (*AKT_B*) [478] and either *ERK*-mediated stabilization, or the absence of its repressor *E2F1*. Finally, during mitotic arrest (*U_Kinetochores*), *MCL-1* is deactivated by *Cyclin B/Cdk1* phosphorylation, which shields it from the *PPA2*-mediated dephosphorylation of its degradation-targeting sites [479].	
	← P	ERK	*ERK* phosphorylates *MCL-1*, promoting its interaction with *Pin1*, which stabilizes it [480, 481].
	← Ind	AKT_B	In order to account for the loss of *MCL-1* in the complete absence of growth factors versus its presence in low growth factor environments, we required basal *AKT* to modulate the strength of *GSK3* inhibition [478].
	⊢ P	GSK3	*MCL-1* is phosphorylated by *GSK3*, leading to ubiquitinylation and degradation of Phosphorylation [478].
	⊢ TR	E2F1	*E2F1* is a direct transcriptional repressor of *MCL-1* [482].

Table S1s: Apoptotic_SW module

	$\dashv$ P	CyclinB	In cells arrested in mitosis, phosphorylation by *Cyclin B/Cdk1* on T92 initiates *MCL-1* degradation [483].
	$\dashv$ P	Cdk1	Phosphorylation by *Cyclin B/Cdk1* in cells arrested in mitosis initiates *MCL-1* degradation [483].
	$\dashv$ Ind	U _Kinetochores	During prolonged mitotic arrest (*U_kinetochores*), *MCL-1* levels drop steadily due to phosphorylation by *JNK*, *p38* and/or *CKII* and its subsequent degradation by the E3 ubiquitin ligase *SCF* (*FBW7*) [479].
	$\dashv$ Lysis	Casp2	*Caspase 2* activation destabilizes the *MCL-1* protein [484].
	$\dashv$ Lysis	Casp3	*Caspase 3* cleaves and deactivated *MCL-1* [485].
BCLXL	**BCLXL** = ((not **Casp3**) and ((**BCL2** and (not **SMAD2_3_4**)) and (not **BAD**))) and (((not **U_Kinetochores**) or (**Plk1** and ((not(**CyclinB** and **Cdk1**)) or (**BCL2** and **MCL_1**)))) or ((**BCL2** and **MCL_1**) and (not(**CyclinB** and **Cdk1**))))		
	Prot		*Bcl-x_L* activity requires the absence of *Caspase 3*. In addition, *BAD* can block *Bcl-x_L*, as it preferentially binds to it rather than *BCL2* (meaning in the absence of the latter *Bcl-x_L* is more likely to be sequestered by basal levels of *BAD*) [486]. Similarly, *TFGβ* can also repress *Bcl-x_L* [487]. Lastly, mitotic *Bcl-x_L* can be inhibited by *Cdk1* activity if either *BCL2*, *MCL-1*, or *Plk1* are OFF. In the absence of *Plk1*, loss of either *BCL2* or *MCL-1* can result in *Bcl-x_L* inhibition (even without *Cdk1* phosphorylation), as we assume its targets are no longer competitively bound by its family members.
	$\dashv$ Ind	SMAD2_3 _4	*BCL-X_L* is repressed by *TFGβ* signaling, an event that mediates *TFGβ*-induced apoptosis [487].
	$\leftarrow$ Ind	Plk1	In addition to other effects of prolonged mitotic arrest on *BCL-2* proteins, *Plk1* inhibition synergistically enhances the inhibitory phosphorylation of *BCL-2* and *BCL-xL*, as well as downregulation of *MCL-1* [488].
	$\dashv$ P	CyclinB	During normal mitosis, *Cyclin B/Cdk1* only transiently phosphorylates part of the *BCL-xL* pool. Prolonged mitosis, however, results in high levels of *BCL-xL* (and *Bcl-2*) phosphorylation, priming the system for *Caspase 2*-mediated apoptosis [489, 490].
	$\dashv$ P	Cdk1	During normal mitosis, *Cyclin B/Cdk1* only transiently phosphorylates part of the *BCL-xL* pool. Prolonged mitosis, however, results in high levels of *BCL-xL* (and *Bcl-2*) phosphorylation, priming the system for *Caspase 2*-mediated apoptosis [489, 490].
	$\dashv$ Ind	U _Kinetochores	Prolonged mitosis is required for the accumulation of *BCL-xL* phosphorylation, weakening its interaction with *Bax* [491].
	$\leftarrow$ Ind	MCL_1	*MCL-1* competes with *BCL-xL* for *BAK* binding; the presence of *MCL-1* can keep part of the *BCL-xL* pool active [492].

Table S1s: Apoptotic_SW module

	← Ind	BCL2	*BCL2* competes with *BCL-xL* for *BAD* binding. As *BCL-xL* is a stronger binding partner of *BAD* [486], here we assume that loss of *BCL2* or *BAD* can both result in low *BCL-xL* activity.
	⊢ IBind	BAD	*Bad* can bind *BCL-xL* and displace it from *BAX*, thus deactivating it [486].
	⊢ Lysis	Casp3	*BCL2* is cleaved and deactivated by *Caspase 3* [493].
BCL2	**BCL2** = (not(((**Casp3** or **BAD**) or **BIM**) or **BIK**)) and (((not **U_Kinetochores**) or (**MCL_1**and**BCLXL**))or(**Plk1**and((**BCLXL**or**MCL_1**)or(not(**Cdk1**and**CyclinB**)))))		
	Prot	While the precise combinatorial logic governing *BCL2* activity is not clear from literature, we modeled *BCL2* as ON in the absence of *Caspase 3*, *BAD*, *BIM* or *BIK*. This choice makes *BCL2* the most sensitive of the three family members to activation of its three inhibitors. In addition, mitotic *BCL2* is blocked by *Cdk1* if both *BCL-xL* and *MCL-1* are OFF. In the absence of *Plk1*, loss of either *BCL2* or *MCL-1* can result in *BCL-2* inhibition (even without *Cdk1* phosphorylation), as we assume its targets are no longer competitively bound by its family members.	
	← Ind	Plk1	In addition to other effects of prolonged mitotic arrest on *BCL2* proteins, *Plk1* inhibition synergistically enhances the inhibitory phosphorylation of *BCL2* and *BCL-xL*, as well as downregulation of *MCL-1* [488].
	⊢ P	CyclinB	*Cyclin B/Cdk1* phosphorylates *BCL2* (and *BCL-xL*) during mitosis [489, 490, 493].
	⊢ P	Cdk1	Prolonged mitosis results in high levels of *BCL-xL* and *BCL2* phosphorylation, priming the system for *Caspase 2*-mediated apoptosis [489, 490, 493].
	⊢ Ind	U _Kinetochores	Prolonged mitosis is required for the accumulation of *BCL2* phosphorylation [489, 490, 493].
	← Ind	MCL_1	*MCL-1* competes with *BCL-xL* for binding most of their apoptotic partners, including *BIK*, *BIM*, *BID*, *BAX* and *BAK*.
	← Ind	BCLXL	*BCL2* competes with *BCL-xL* for binding most of their apoptotic partners, including *BIK*, *BIM*, *BID*, *BAX* and *BAK*.
	⊢ IBind	BAD	*BCL2* competes with *BCL-xL* for *BAD* binding. *BAD* displaces *BCL2* from its inhibitory binding of *Bax/Bak*. Although *BCL-xL* is a stronger binding partner, we assume that *BAD* alone cannot fully block *BCL-xL* in the presence of *BCL2* [486].
	⊢ IBind	BIK	*BIK* binds *BCL2* and they mutually inhibit each other's activity [494].
	⊢ IBind	BIM	*BIM* binds *BCL2* and they mutually inhibit each other's ability to activate further targets [495].
	⊢ Lysis	Casp3	*BCL2* is cleaved and deactivated by *Caspase 3* [493].

Table S1s: Apoptotic_SW module

BAD	**BAD** = (**Casp3** or (not(((**AKT_H** or **AKT_B**) or **ERK**) or **S6K**))) or (**Casp8** and ((not((**AKT_B** and **ERK**) and **S6K**)) and (not(**AKT_H** and (**AKT_B** or **ERK**)))))		
	Prot	*BAD* in our model is ON when cleaved by *Caspase 3*, or in the complete absence of survival signals (*AKT*, *ERK* or *S6K*). Alternatively, *BAD* can be cleaved and activated by *Caspase 8* in the absence of strong survival signaling. We modeled this inhibitory survival signal as either the combined activity of *ERK*, *S6K* and (at least) basal *AKT*, or high *AKT* in the joint presence of ERK and basal *AKT* (indicating that *AKT_H* will not drop by the next time-step).	
	⊢ P	ERK	*ERK* phosphorylates *BAD* at Ser-112, inducing its sequestration away from the mitochondrial membrane where its BCL-2 family targets are located (e.g., *BCL-2*, *BCL-xL*)[496].
	⊢ P	AKT_B	*Akt* phosphorylates *BAD* at Ser-136, inducing its sequestration away from the mitochondrial membrane where its BCL-2 family targets are located (e.g., *BCL2*, *BCL-xL*) [497].
	⊢ P	AKT_H	*Akt* phosphorylates *BAD* at Ser-136, inducing its sequestration away from the mitochondrial membrane where its BCL-2 family targets are located (e.g., *BCL2*, *BCL-xL*) [497].
	⊢ P	S6K	*S6K1* phosphorylates *BAD* at Ser-155, directly blocking its binding to *BCL-xL* [498].
	← Lysis	Casp8	*Caspase 8* is also able to cleave *BAD*, generating a more potently apoptotic fragment [499]. In addition, *TRAIL*-mediated apoptosis results in *BAD* cleavage by a Caspase upstream of MOMP, creating a potent apoptotic inducer before full *Caspase 3* activation [500].
	← Lysis	Casp3	*Caspase 3* cleaves *BAD*, generating a more potently apoptotic fragment [499].
BIK	**BIK** = (not((**MCL_1** or **BCLXL**) or **BCL2**)) and (**SMAD2_3_4** or (not **AKT_H**))		
	Prot	*BIK* is free to activate its target, *BAX*, only when it is not sequestered by any of the three *BCL-2* family proteins [492] and it is either induced by *SMADs* [487] or protected by no more than basal *AKT* activity, which lowers the level of both *BCL2* and *BCL-*x_L [501].	
	⊢ Ind	AKT_H	*AKT_H* elevates the level of both *BCL2* [502] and *BCL-*x_L [501], thus making it easier for these inhibitors to sequester *BIK*.
	← TR	SMAD2_3_4	*Smad2/3/4* are direct transciprional inducers of *BIK* [487].
	⊢ IBind	MCL_1	*MCL-1* binds *BIK*; they mutually inhibit each other [503].
	⊢ IBind	BCLXL	*BCL-xL* binds *BIK*; they mutually inhibit each other [504].
	⊢ IBind	BCL2	*BCL2* binds *BIK*; they mutually inhibit each other [494].

Table S1s: Apoptotic_SW module

BIM	**BIM** = **FoxO3** and ((**GSK3** and not(**ERK** or **MCL_1** or **ZEB1** or **BCLXL** or **BCL2**)) or ((**Runx1** and not **ERK** and not **NfkB**) and (not((**MCL_1** and **BCLXL**) and **BCL2**))))		
	Prot	*BIM*'s pro-apoptotic activity requires expression driven by *FoxO3* and aided by *GSK3*, as well as the absence of *ERK* or any of the three inhibitory *BCL2* family proteins. Alternatively, *Runx1* can induce BIM to apoptotic levels in the absence of *ERK* and *NF-κB*, assuming the three inhibitory *BCL2* family proteins are not all fully active.	
	⊣ Ind	ERK	The *MEK/ERK* pathway represses *BIM* protein levels, likely via transcriptional repression [505].
	← TR	FoxO3	*FoxO3* is a transcriptional activator of *BIM* [506].
	← Ind	GSK3	*GSK3* kinase is likely required for the *AP1*-dependent expression of *BIM* [507].
	⊣ TR	NfkB	*NF-κB* is a transcriptional repressor of *BIM* [508, 509].
	← TR	Runx1	*TGF-β* stimulation leads to increased protein levels of pro-apoptotic *BIM* [510] via *Runx1* induction and subsequent *Foxo3/Runx1* driven transcription [511].
	⊣ TR	ZEB1	*ZEB1* is a direct transcriptional repressor of the *BIM* promoter [512].
	⊣ IBind	MCL_1	*MCL-1* binds *BIM* and inhibits its apoptotic activity [513].
	⊣ IBind	BCLXL	*BCL-xL* binds *BIM* and inhibits its apoptotic activity [495].
	⊣ IBind	BCL2	*BLC2* binds *BIM* and inhibits its apoptotic activity [495].
BID	**BID** = **Casp8** or (**Casp2** and (not((**BCL2** or **BCLXL**) or **MCL_1**)))		
	Prot	*BID* is truncated in response to *Caspase 8* activation. In addition, *Caspase 2* can also promote *BID* activation once all three pro-apoptotic *BCL2* family proteins are blocked.	
	← Lysis	Casp8	In response to *TRAIL* (or *FAS* ligand), the initiator *Caspase 8* cleaves *BID* to its active truncated form [514, 515, 516].
	← Lysis	Casp2	*Caspase 2* cleaves *BID* to its active truncated form [517].
	⊣ IBind	MCL_1	All three anti-apoptotic BCL2 proteins (*BCL2*, *BCL-xL* and *MCL-1*) sequesters *BID* into stable complexes, preventing them from activating *BAX* or *BAK* [518].
	⊣ IBind	BCLXL	All three anti-apoptotic BCL2 proteins (*BCL2*, *BCL-xL* and *MCL-1*) sequesters *BID* into stable complexes, preventing them from activating *BAX* or *BAK* [518].
	⊣ IBind	BCL2	All three anti-apoptotic BCL2 proteins (*BCL2*, *BCL-xL* and *MCL-1*) sequesters *BID* into stable complexes, preventing them from activating *BAX* or *BAK* [518].

Table S1s: Apoptotic_SW module

BAK	**BAK** = (**BID** and((**BIM** or **BIK**)or(not((**BCL2** and **BCLXL**)and **MCL_1**))))or((**BIM** or **BIK**) and (not(**BCLXL** or **MCL_1**)))		
	Prot	Given that *BAK* is preferentially activated by *BID* compared to *BIM* [519] and that it is less responsive to sequestration by *BCL2* than the other two anti-apoptotic *BCL2* family proteins [520, 521], *BAK* in our model turns on when stimulated by *BID* if one or more *BCL2* family proteins are absent, or if *BIM* or *BIK* are also present. In contrast, *BIM* or *BIK* only activate *BAK* if *BCL-xL* and *MCL-1* are absent (*BCL-2* alone cannot block them).	
	⊢ IBind	MCL_1	*MCL-1* binds *BAK* and prevent its oligomerization in the mitochondrial membrane [520, 521].
	⊢ IBind	BCLXL	*BCL-xL* binds *BAK* and prevent its oligomerization in the mitochondrial membrane [522, 520, 521].
	⊢ IBind	BCL2	*BCL2* can also bind *BAK* to prevent its oligomerization, but it does so less potently than the other two BCL-2 family members [520, 521, 523].
	← Compl	BIK	*BIK* can aid the activation of both *BAK* and *BAX* by triggering *BAK* oligomerization on the ER membrane and promoting a Ca^{2+} efflux required for the fragmentation of hyper fused mitochondrial tubules, aiding *BAK* and *BAX* activation [524].
	← Compl	BIM	*BAK* is preferentially activated by *BID* compared to *BIM*, but *BIM* can also promote BAK oligomerization [519].
	← Compl	BID	Activated (truncated) *BID* binds to mitochondrial *BAK*, resulting in its activation and oligomerization in the mitochondrial membrane, followed by *cytochrome c* release [525].
BAX	**BAX** = (**BIM** and((**BID** or **BIK**)or(not((**BCL2** and **BCLXL**)and **MCL_1**))))or((**BID** or **BIK**) and (not(**BCL2** or **BCLXL**)))		
	Prot	In contrast to *BAK*, *BAX* is preferentially activated by *BIM* compared to *BID* [519] and it is less responsive to sequestration by *MCL-1* than the other two anti-apoptotic BCL2 family proteins [520, 521]. *BAX* in our model turns on when stimulated by *BIM* if one or more *BCL2* family proteins are absent, or if *BID* or *BIK* are also present. In contrast, *BID* or *BIK* only activate *BAK* if *BCL2* and *BCL-xL* are both absent (*MCL-1* alone cannot block them).	
	⊢ IBind	MCL_1	*MCL-1* can also bind *BAK* to prevent its oligomerization, but it does so less potently than the other two BCL-2 family members [520, 521, 526].
	⊢ IBind	BCLXL	*BCL-xL* binds *BAX* and prevent its oligomerization in the mitochondrial membrane [520, 521].
	⊢ IBind	BCL2	*BCL2* binds *BAX* and prevent its oligomerization in the mitochondrial membrane [520, 521].
	← Compl	BIK	*BIK* can aid the activation of both *BAK* and *BAX* by triggering *BAK* oligomerization on the ER membrane and promoting a Ca^{2+} efflux required for the fragmentation of hyper fused mitochondrial tubules, aiding *BAK* and *BAX* activation [524].

Table S1s: Apoptotic_SW module

	← Compl	BIM	Activated *BIM* binds to mitochondrial *BAX*, resulting in its allosteric activation and oligomerization in the mitochondrial membrane, leading to *cytochrome c* release [519].
	← Compl	BID	*BAX* is preferentially activated by *BIM* compared to *BID*, but *BID* can also promote *BAK* oligomerization [519].
Cyto_C	**Cyto_C** = **BAX** or **BAK**		
	Prot	*Cytochrome C* release from mitochondria requires the oligomerization of either *BAK* or *BAX* [527].	
	← Loc	BAK	*BAK* oligomerization at the mitochondrial membrane triggers MOMP, which results in the release of *cytochrome C* from mitochondria [527].
	← Loc	BAX	*BAX* oligomerization at the mitochondrial membrane triggers MOMP, which results in the release of *cytochrome C* from mitochondria [528, 529].
SMAC	**SMAC** = **BAX** or **BAK**		
	Prot	*SMAC/Diablo* release from mitochondria requires the oligomerization of either *BAK* or *BAX* [527, 530].	
	← Loc	BAK	*BAK* oligomerization at the mitochondrial membrane triggers MOMP, which results in the release of *SMAC/Diablo* from mitochondria [527, 530].
	← Loc	BAX	*BAX* oligomerization at the mitochondrial membrane triggers MOMP, which results in the release of *SMAC/Diablo* from mitochondria [527, 530].
IAPs	**IAPs** = (not **SMAC**) or **AKT_H** or (**Hypoxia** and **AKT_B**)		
	Prot	Inhibitor of Apoptosis Proteins (*IAP*s) are active in the absence of *SMAC* inhibition, or following *AKT_H* mediated upregulation (this protection from *SMAC* requires peak or oncogenic *AKT* activity). Under hypoxia, *IAP-2* levels are increased by a *Hif-1α* independent mechanism [531]; a process we assume requires at least basal levels of *AKT*.	
	← Ind	AKT_H	*cIAP-2* and *XIAP* are both transcriptionally up-regulated in response to strong *PI3K/AKT1* activation [532].
	⊣ IBind	SMAC	*SMAC/Diablo* binds tightly to *IAP* proteins and blocks their ability to inhibit *Caspase 3* [533].
	← Ind	Hypoxia	Increased levels of *IAP-2* are seen under hypoxic conditions, induced by *Hif-1α* independent mechanisms [531].
	← Ind	AKT_B	*cIAP-2* and *XIAP* are both transcriptionally up-regulated in response to *PI3K/AKT1* activation and is necessary for hypoxic prevention of apoptosis through IAPS. [532].
Casp9	**Casp9** = **Casp3** or ((not **IAPs**) and **Cyto_C**)		
	PTase	*Procaspase 9* is cleaved into active *Caspase 9* by *Caspase 3*, or by the apoptosome (which relies on *cytochrome C* for its assembly) in the absence of *IAP* proteins.	

Table S1s: Apoptotic_SW module

	← Compl	Cyto_C	*Cytochrome c* binds to *APAF-1* proteins, promoting their assembly into the apoptosome, a platform for *procaspase 9* binding and cleavage into its active form [534].
	⊢ IBind	IAPs	*XIAP*, *cIAP1* and *cIAP2* inhibit the *cytochrome c*-induced activation of *procaspase-9* [535].
	← Lysis	Casp3	*Procaspase 9* is a direct cleavage target of *Caspase 3* [477].
Casp3	**Casp3** = ((**Casp9** and **Casp8**) or (**Casp3** and (**Casp9** or **Casp8**))) or ((not **IAPs**) and ((**Casp9** or **Casp8**) or **Casp3**))		
	PTase	Activation of *Caspase 3* requires proteolytic cleavage of *procaspase-3* by initiator caspases such as *Caspase 9* or *Caspase 8*. In our model, cooperation of two of the three caspases (*Casp9*, *Casp8*, *Casp3*) is required in the presence of *IAP*s, which inhibit the proteolytic activity of *Caspase 3* by bind tightly to its active site. In the absence of *IAP*s, either of the three caspases can cleave and activate *Caspase 3*.	
	← Lysis	Casp8	*Caspase 8* can cleave *Caspase 3* [536], but full *Caspase 3* activation also requires MOMP (potentially due to a need for *IAP* inhibition) [537].
	⊢ IBind	IAPs	*IAP*s bind tightly to the active site of *Caspase 3*, keeping its activity in check [535, 538].
	← Lysis	Casp9	Active *Caspase 9* cleaves *procaspase 3* [539].
	← Per	Casp3	Once activated, *Caspase 3* helps sustain its own activation by cleaving *procaspase 8* and *6*. *Caspase 6*, in turn, generates additional active *caspase 8* and *9*. Together they all sustains a continuing active pool of Caspase 3.

Table S1t: DNA_Fragmentation module

Target Node	Node Gate<br>Node Type<br>Link Type	Node Description<br>Input Node	Link Description
CAD	**CAD** = **Casp3** and **Casp9**		
	DNase	Caspase-activated DNase (*CAD*) is activated when its inhibition is released via the cleavage of *ICAD* (inhibitor of caspase-activated DNase). While *Capsase 3* and *7* (a direct target of *Caspase 9*) can inhibit *ICAD* [540], in our model they are both required, as *CAD* = ON is represents terminal, irreversible apoptotic commitment, which is fully locked in when both *Caspase 3* and *9* are on.	
	← Ind	Casp9	In addition of *Caspase 3*, *CAD* inhibition can also be relieved by *ICAD* cleavage by *Caspase 7*, which is a direct target of *Caspase 9* [540].

Table S1t: DNA_Fragmentation module

	$\underset{\text{Ind}}{\leftarrow}$	Casp3	*Caspase 3* relives *CAD* inhibition by cleaving its inhibitor *ICAD* [540].

Table S2a: Key to Node Type Symbols

Symbol	Node Type	Description
Cell	Cell	Node type used when a node's state is used to represent discrete phenotypes of an entire cell; appropriate for multi models.
DM	DM_Switch	Multi-stable regulatory module (Dynamically Modular Switch) in a higher-level model layer.
Conn	Connector	Grouping of nodes that are either mono-stable, or do not form a switch-like circuit that controls discrete phenotype transitions. For example, signaling input layers without feedback, or multi-step links between distinct switches can be represented as connector nodes in coarse-grained (switch-level) models.
Env	Environment	Nodes or modules that represent the extracellular environment of a single cell or cell collective. These are self-sustaining nodes or node groups that maintain their initial states and receive no feedback from the rest of the network (they act as inputs).
Proc	Process	Nodes or modules that stand in for complex cellular processes not modeled in detail (e.g., DNA replication or the process of aligning chromosomes at the metaphase plane during mitosis).
MSt	Macro_Structure	Nodes or modules that represent the state of large, complex cellular structures such as DNA content, cytoskeletal features, junctions or mitochondria.
Met	Metabolite	Regulatory node representing a metabolite (not protein, gene product or complex structure).
mRNA	MRNA	mRNA.
miR	MicroRNA	microRNA.
PC	Protein_Complex	Protein complex represented by a single node or via a key member of the complex.
Rec	Receptor	Cell surface receptor protein or complex.
Adap	Adaptor_Protein	Protein that helps scaffold a signaling complex or other large assembly of proteins.
Secr	Secreted_Protein	Protein secreted into the extracellular environment, such that the state of the node tagged with this type represents the availability fo this protein outside the cell.
TF	TF_Protein	Transcription factor.
K	Kinase	Kinase (enzyme that catalyzes the phosphorylation of its target).
Ph	Phosphatase	Phosphatase (enzyme that catalyzes the removal of phosphorylation from its target).
UbL	Ubiquitin_Ligase	Ubiquitin ligase (protein that recruits an ubiquitin-conjugating enzyme that has been loaded with ubiquitin to a target protein and assists or directly catalyzes the transfer of ubiquitin from the ubiquitin-conjugating enzyme to the target).
PTase	Protease	Protease (enzyme that catalyzes the breakdown of proteins into smaller fragments).
DNase	DNase	Deoxyribonuclease (DNase, for short); endonuclease that catalyzes the hydrolytic cleavage of the DNA backbone.

Table S2a: Key to Node Type Symbols

CAM	CAM	Cell adhesion proteins located on the cell surface.
CDK	CDK	Cyclin-dependent kinase.
CDKI	CDKI	Cyclin-dependent kinase inhibitor.
GEF	GEF	Guanine nucleotide exchange factor.
GAP	GAP	GTPase-activating protein (also called GTPase-accelerating protein).
GTPa	GTPase	GTPase enzymes that hydrolyze ATP to ADP.
Enz	Enzyme	Enzyme that does not fit the more specific enzyme categories listed above.
Prot	Protein	Regulatory protein that does not fit any of the more specific classifications listed above.
MP	Membrane_Potential	A relative measure of membrane potential across a biological membrane, generally indicating whether this potential is within the normal range, or abnormally low / high in a way that affects other regulatory processes.
lncRNA	LncRNA	Long intervening noncoding RNA
SLig	Cell_Surgace_Ligand	Membrane-bound signaling molecule that serves as a ligand to receptors on neighboring cells.

Table S2b: Key to Link Type Symbols

Symbol	Link Type	Description
Env	Enforced_Env	This link type represents self-loops on Environment nodes, which guarantee that these nodes maintain their initial state throughout a time-course simulation unless they are explicitly altered by the simulation's settings.
Ind	Indirect	Regulatory influence that does not involve direct binding, processing, or enzyme activity.
ComplProc	Complex_Process	Regulatory influence that is not modeled in detail, but involves more than one molecule or a macrostructure. For example, the physical need for kinetochores on replicated sister chromatids for the assembly of certain protein complexes can be represented as a link from the node representing kinetochores to the regulatory proteins, with a Complex_Process link type.
Per	Persistence	This link type represents self-loops that alter the ability of a node to stay in a particular state depending on its own current state. For example, if transcription of a protein is easier to maintain than to induce de novo, this may be encoded by a logic gate that includes the node itself and creates a self-loop. The link type of this loop is "Persistence".
TR	Transcription	Action of a transcription factor to alter the expression of the target node (mRNA or protein). Link type should be used for induction as well as repression (the link effect contains this information).

Table S2b: Key to Link Type Symbols

TL	Translation	Regulatory influence that controls the translation of mRNA into protein; should be used for induction as well as repression of translation.
Ligand	Ligand_Binding	Binding of extracellular ligand to its receptor.
Compl	Complex_Formation	Binding even that leads to a regulatory protein complex.
IBind	Inhibitory_Binding	Binding even that represses the target node's level or activity.
Loc	Localization	Regulatory influence that alters the localization of a molecule.
BLoc	Binding_Localization	Binding even that alters the localization of a molecule.
PBind	Protective_Binding	Binding even that increases / protects the target node's activity.
Unbind	Unbinding	A regulatory influence that causes the target node to be released from a protein complex and change its activity (increase or decrease) as a result.
P	Phosphorylation	Phosphorylation.
DP	Dephosphorylation	Dehosphorylation.
PLoc	Phosphorylation_Localization	Phosphorylation resulting in altered protein localization.
Ubiq	Ubiquitination	Ubiquitination, usually leading to protein degradation.
Deg	Degradation	Regulatory influence leading to the degradation of the target molecule (more general than Ubiquitination; the latter link type should be used when appropriate).
GEF	GEF_Activity	Action of a Guanine nucleotide exchange factor (GEF) leading to GTP loading onto (and usually the activation of) a GTPase.
GAP	GAP_Activity	Actions of a GTPase-activating protein (GAP) leading to the hydrolysis of GTP by (and usually de-activation of) a GTPase.
Lysis	Proteolysis	Protein cleavage.
Cat	Catalysis	Increasing the rate of metabolite production by an enzyme.
Epi	Epigenetic	Process that alters gene expression via modifying chromatin condensation or altering DNA methylation.
TrConf	Transcription_Conflict	
Secr	Secretion	Secretion or shedding of a protein or other regulatory molecule to the extracellular environment.
RNAi	RNAi	This process represents inhibitory binding of cytoplasmic mRNAs by RISC-bound microRNAs that block translation and/or enhance mRNA degradation.
Acet	Acetylation	Acetylation
Deacet	Deacetylation	Deacetylation
-OH	Hydroxylation	Hydroxylation

Table S2c: Key to Link Effect Symbols

Symbol	Link Effect	Description

Table S2c: Key to Link Effect Symbols

←	Activation	Link in which the input node aids the expression, activity, persistence or localization of the target such that the target is easier to turn/keep in an ON state. It can be used for multi-level nodes as long as these levels represent increasing intervals of activity.
⊢	Repression	Link in which the input node hinders the expression, activity, persistence or localization of the target such that the target is easier to turn/keep in an OFF state. It can be used for multi-level nodes as long as these levels represent increasing intervals of activity.
•–	Context_Dependent	Link acting on a node in such a way that it activates it under certain conditions (e.g., when another input is OFF), but represses it when this condition is not met. The XOR gate is a good example of a context-dependent link effect.
⊥	Inapt	This link-type refers to connections between complex regulatory switches (rather than molecules), where categorizing the effect of an input as Activation or Repression, or even context-depended activation or repression does not apply. This is usually the case with multi-state switches, where the 3 or more phenotypes represented by the discrete states of these switches do not have a meaningful ordering. Thus, stating that this switch is "activated" by another one is not appropriate.

References

[1] Zhenyi Ma, Zhe Liu, David P. Myers, and Lance S. Terada. Mechanotransduction and anoikis: Death and the homeless cell. *Cell Cycle (Georgetown, Tex.)*, 7(16):2462–2465, August 2008.

[2] Gregg L. Semenza. Defining the Role of Hypoxia-Inducible Factor 1 in Cancer Biology and Therapeutics. *Oncogene*, 29(5):625–634, February 2010.

[3] Rupert Courtnay, Darleen C. Ngo, Neha Malik, Katherine Ververis, Stephanie M. Tortorella, and Tom C. Karagiannis. Cancer metabolism and the Warburg effect: The role of HIF-1 and PI3K. *Molecular Biology Reports*, 42(4):841–851, April 2015.

[4] Herbert Sizek, Dávid Deritei, Katie Fleig, Marlayna Harris, Peter L. Regan, Kimberly Glass, and Erzsébet Ravasz Regan. Unlocking Mitochondrial Dysfunction-Associated Senescence (MiDAS) with NAD+ – a Boolean Model of Mitochondrial Dynamics and Cell Cycle Control. *Translational Oncology*, (submitted), December 2023.

[5] Kreon Koukoulas, Antonis Giakountis, Angeliki Karagiota, Martina Samiotaki, George Panayotou, George Simos, and Ilias Mylonis. ERK signaling controls productive HIF-1 binding to chromatin and cancer cell adaptation to hypoxia through HIF-1α interaction with NPM1. *Molecular Oncology*, 15(12):3468–3489, December 2021.

[6] Brooke M. Emerling, Frank Weinberg, Juinn-Lin Liu, Tak W. Mak, and Navdeep S. Chandel. PTEN regulates p300-dependent hypoxia-inducible factor 1 transcriptional activity through Forkhead transcription factor 3a (FOXO3a). *Proceedings of the National Academy of Sciences of the United States of America*, 105(7):2622–2627, February 2008.

[7] E. Laughner, P. Taghavi, K. Chiles, P. C. Mahon, and G. L. Semenza. HER2 (neu) signaling increases the rate of hypoxia-inducible factor 1alpha (HIF-1alpha) synthesis: Novel mechanism for HIF-1-mediated vascular endothelial growth factor expression. *Molecular and Cellular Biology*, 21(12):3995–4004, June 2001.

[8] K. M. Dodd, J. Yang, M. H. Shen, J. R. Sampson, and A. R. Tee. mTORC1 drives HIF-1α and VEGF-A signalling via multiple mechanisms involving 4E-BP1, S6K1 and STAT3. *Oncogene*, 34(17):2239–2250, April 2015.

[9] Daniela Flügel, Agnes Görlach, Carine Michiels, and Thomas Kietzmann. Glycogen synthase kinase 3 phosphorylates hypoxia-inducible factor 1alpha and mediates its destabilization in a VHL-independent manner. *Molecular and Cellular Biology*, 27(9):3253–3265, May 2007.

[10] Patrick C. Mahon, Kiichi Hirota, and Gregg L. Semenza. FIH-1: A novel protein that interacts with HIF-1α and VHL to mediate repression of HIF-1 transcriptional activity. *Genes & Development*, 15(20):2675–2686, October 2001.

[11] Jan H. Marxsen, Petra Stengel, Kathrin Doege, Pekka Heikkinen, Terhi Jokilehto, Thomas Wagner, Wolfgang Jelkmann, Panu Jaakkola, and Eric Metzen. Hypoxia-inducible factor-1 (HIF-1) promotes its degradation by induction of HIF-α-prolyl-4-hydroxylases. *Biochemical Journal*, 381(Pt 3):761–767, August 2004.

[12] Rebecca J. Appelhoff, Ya-Min Tian, Raju R. Raval, Helen Turley, Adrian L. Harris, Christopher W. Pugh, Peter J. Ratcliffe, and Jonathan M. Gleadle. Differential Function of the Prolyl Hydroxylases PHD1, PHD2, and PHD3 in the Regulation of Hypoxia-inducible Factor *. *Journal of Biological Chemistry*, 279(37):38458–38465, September 2004.

[13] Ayumu Taguchi, Kiyoshi Yanagisawa, Masaharu Tanaka, Ke Cao, Yasushi Matsuyama, Hidemi Goto, and Takashi Takahashi. Identification of Hypoxia-Inducible Factor-1α as a Novel Target for miR-17-92 MicroRNA Cluster. *Cancer Research*, 68(14):5540–5545, July 2008.

[14] Yuree Byun, Young-Chul Choi, Yunhui Jeong, Gangtae Lee, Sena Yoon, Yongsu Jeong, Jaeseung Yoon, and Kwanghee Baek. MiR-200c downregulates HIF-1α and inhibits migration of lung cancer cells. *Cellular & Molecular Biology Letters*, 24:28, April 2019.

[15] Ho-Youl Lee, Taekyong Lee, Naery Lee, Eun G. Yang, Cheolju Lee, Jonghyun Lee, Eun-Yi Moon, Joohun Ha, and Hyunsung Park. Src activates HIF-1α not through direct phosphorylation of HIF-1α specific prolyl-4 hydroxylase 2 but through activation of the NADPH oxidase/Rac pathway. *Carcinogenesis*, 32(5):703–712, May 2011.

[16] Stephanie McMahon, Martine Charbonneau, Sebastien Grandmont, Darren E. Richard, and Claire M. Dubois. Transforming Growth Factor $B1$ Induces Hypoxia-inducible Factor-1 Stabilization through Selective Inhibition of PHD1_2 Expression. *Journal of Biological Chemistry*, 281(34):24171–24181, August 2006.

[17] Pramod Mallikarjuna, Raviprakash T. Sitaram, Karthik Aripaka, Börje Ljungberg, and Marene Landström. Interactions between TGF-β type I receptor and hypoxia-inducible factor-α mediates a synergistic crosstalk leading to poor prognosis for patients with clear cell renal cell carcinoma. *Cell Cycle*, 18(17):2141–2156, September 2019.

[18] David Lando, Daniel J. Peet, Jeffrey J. Gorman, Dean A. Whelan, Murray L. Whitelaw, and Richard K. Bruick. FIH-1 is an asparaginyl hydroxylase enzyme that regulates the transcriptional activity of hypoxia-inducible factor. *Genes & Development*, 16(12):1466–1471, June 2002.

[19] Robert D. Guzy, Beatrice Hoyos, Emmanuel Robin, Hong Chen, Liping Liu, Kyle D. Mansfield, M. Celeste Simon, Ulrich Hammerling, and Paul T. Schumacker. Mitochondrial complex III is required for hypoxia-induced ROS production and cellular oxygen sensing. *Cell Metabolism*, 1(6):401–408, June 2005.

[20] Joan Seoane and Roger R. Gomis. TGF-β Family Signaling in Tumor Suppression and Cancer Progression. *Cold Spring Harbor Perspectives in Biology*, 9(12):a022277, December 2017.

[21] Philip A. Gregory, Cameron P. Bracken, Eric Smith, Andrew G. Bert, Josephine A. Wright, Suraya Roslan, Melanie Morris, Leila Wyatt, Gelareh Farshid, Yat-Yuen Lim, Geoffrey J. Lindeman, M. Frances Shannon, Paul A. Drew, Yeesim Khew-Goodall, and Gregory J. Goodall. An autocrine TGF-beta/ZEB/miR-200 signaling network regulates establishment and maintenance of epithelial-mesenchymal transition. *Molecular Biology of the Cell*, 22(10):1686–1698, May 2011.

[22] Xiao-Jun Tian, Hang Zhang, and Jianhua Xing. Coupled reversible and irreversible bistable switches underlying TGFβ-induced epithelial to mesenchymal transition. *Biophysical Journal*, 105(4):1079–1089, August 2013.

[23] Pramod Mallikarjuna, Raviprakash T. Sitaram, Maréne Landström, and Börje Ljungberg. VHL status regulates transforming growth factor-β signaling pathways in renal cell carcinoma. *Oncotarget*, 9(23):16297–16310, March 2018.

[24] Xuhua Tan, Yi Zhu, Chuan Chen, Xiaoyun Chen, Yingyan Qin, Bo Qu, Lixia Luo, Haotian Lin, Mingxing Wu, Weirong Chen, and Yizhi Liu. Sprouty2 Suppresses Epithelial-Mesenchymal Transition of Human Lens Epithelial Cells through Blockade of Smad2 and ERK1/2 Pathways. *PloS One*, 11(7):e0159275, 2016.

[25] Flore Nallet-Staub, Xueqian Yin, Cristèle Gilbert, Véronique Marsaud, Saber Ben Mimoun, Delphine Javelaud, Edward B. Leof, and Alain Mauviel. Cell density sensing alters TGF-β signaling in a cell-type-specific manner, independent from Hippo pathway activation. *Developmental Cell*, 32(5):640–651, March 2015.

[26] Stephen G. Szeto, Masahiro Narimatsu, Mingliang Lu, Xiaolin He, Ahmad M. Sidiqi, Monica F. Tolosa, Lauren Chan, Krystale De Freitas, Janne Folke Bialik, Syamantak Majumder, Stellar Boo, Boris Hinz, Qinghong Dan, Andrew Advani, Rohan John, Jeffrey L. Wrana, Andras Kapus, and Darren A. Yuen.

YAP/TAZ Are Mechanoregulators of TGF-β-Smad Signaling and Renal Fibrogenesis. *Journal of the American Society of Nephrology: JASN*, 27(10):3117–3128, October 2016.

[27] Jun Zhou, Yasamin Dabiri, Rodrigo A. Gama-Brambila, Shahrouz Ghafoory, Mukaddes Altinbay, Arianeb Mehrabi, Mohammad Golriz, Biljana Blagojevic, Stefanie Reuter, Kang Han, Anna Seidel, Ivan Đikić, Stefan Wölfl, and Xinlai Cheng. pVHL-mediated SMAD3 degradation suppresses TGF-β signaling. *The Journal of Cell Biology*, 221(1):e202012097, December 2021.

[28] I. Serrano, P. C. McDonald, F. E. Lock, and S. Dedhar. Role of the integrin-linked kinase (ILK)/Rictor complex in TGFβ-1-induced epithelial-mesenchymal transition (EMT). *Oncogene*, 32(1):50–60, January 2013.

[29] Paul C. McDonald, Arusha Oloumi, Julia Mills, Iveta Dobreva, Mykola Maidan, Virginia Gray, Elizabeth D. Wederell, Marcel B. Bally, Leonard J. Foster, and Shoukat Dedhar. Rictor and integrin-linked kinase interact and regulate Akt phosphorylation and cancer cell survival. *Cancer Research*, 68(6):1618–1624, March 2008.

[30] Sylvie Thuault, Ulrich Valcourt, Maj Petersen, Guidalberto Manfioletti, Carl-Henrik Heldin, and Aristidis Moustakas. Transforming growth factor-beta employs HMGA2 to elicit epithelial-mesenchymal transition. *The Journal of Cell Biology*, 174(2):175–183, July 2006.

[31] G. J. Hannon and D. Beach. p15INK4B is a potential effector of TGF-beta-induced cell cycle arrest. *Nature*, 371(6494):257–261, September 1994.

[32] X. H. Feng, X. Lin, and R. Derynck. Smad2, Smad3 and Smad4 cooperate with Sp1 to induce p15(Ink4B) transcription in response to TGF-beta. *The EMBO journal*, 19(19):5178–5193, October 2000.

[33] C Sandhu, J Garbe, N Bhattacharya, J Daksis, C H Pan, P Yaswen, J Koh, J M Slingerland, and M R Stampfer. Transforming growth factor beta stabilizes p15INK4B protein, increases p15INK4B-cdk4 complexes, and inhibits cyclin D1-cdk4 association in human mammary epithelial cells. *Molecular and Cellular Biology*, 17(5):2458–2467, May 1997.

[34] P. Staller, K. Peukert, A. Kiermaier, J. Seoane, J. Lukas, H. Karsunky, T. Möröy, J. Bartek, J. Massagué, F. Hänel, and M. Eilers. Repression of p15INK4b expression by Myc through association with Miz-1. *Nature Cell Biology*, 3(4):392–399, April 2001.

[35] K. Katayama, A. Nakamura, Y. Sugimoto, T. Tsuruo, and N. Fujita. FOXO transcription factor-dependent p15(INK4b) and p19(INK4d) expression. *Oncogene*, 27(12):1677–1686, March 2008.

[36] Sneha Ramesh, Xiao-Jun Qi, Gary M Wildey, Janet Robinson, Jeffery Molkentin, John Letterio, and Philip H Howe. TGFβ-mediated BIM expression and apoptosis are regulated through SMAD3-dependent expression of the MAPK phosphatase MKP2. *EMBO reports*, 9(10):990–997, October 2008.

[37] Gary M. Wildey, Supriya Patil, and Philip H. Howe. Smad3 potentiates transforming growth factor beta (TGFbeta)-induced apoptosis and expression of the BH3-only protein Bim in WEHI 231 B lymphocytes. *The Journal of Biological Chemistry*, 278(20):18069–18077, May 2003.

[38] Gary M. Wildey and Philip H. Howe. Runx1 is a co-activator with FOXO3 to mediate transforming growth factor beta (TGFbeta)-induced Bim transcription in hepatic cells. *The Journal of Biological Chemistry*, 284(30):20227–20239, July 2009.

[39] Mark E. Ebel and Geoffrey S. Kansas. Functions of Smad Transcription Factors in TGF-β1-Induced Selectin Ligand Expression on Murine CD4 Th Cells. *Journal of Immunology (Baltimore, Md.: 1950)*, 197(7):2627–2634, October 2016.

[40] Mark A Lemmon and Joseph Schlessinger. Cell signaling by receptor tyrosine kinases. *Cell*, 141(7):1117–1134, June 2010.

[41] Samrein B. M. Ahmed and Sally A. Prigent. Insights into the Shc Family of Adaptor Proteins. *Journal of Molecular Signaling*, 12:2, May 2017.

[42] Ying E. Zhang. Non-Smad pathways in TGF-beta signaling. *Cell Research*, 19(1):128–139, January 2009.

[43] D. D. Schlaepfer, K. C. Jones, and T. Hunter. Multiple Grb2-mediated integrin-stimulated signaling pathways to ERK2/mitogen-activated protein kinase: Summation of both c-Src- and focal adhesion kinase-initiated tyrosine phosphorylation events. *Molecular and Cellular Biology*, 18(5):2571–2585, May 1998.

[44] A Uzman. Molecular Cell Biology (4th edition) Harvey Lodish, Arnold Berk, S. Lawrence Zipursky, Paul Matsudaira, David Baltimore and James Darnell; Freeman & Co., New York, NY, 2000, 1084 pp., list price 102.25, ISBN 0-7167-3136-3. *Biochemistry and Molecular Biology Education*, 29(3):126–128, 2001.

[45] A P Belsches, M D Haskell, and Sarah J. Parsons. Role of c-Src tyrosine kinase in EGF-induced mitogenesis. *Frontiers in Bioscience*, 2(4):d501–518, 1997.

[46] Oliver Rocks, Anna Peyker, Martin Kahms, Peter J. Verveer, Carolin Koerner, Maria Lumbierres, Jürgen Kuhlmann, Herbert Waldmann, Alfred Wittinghofer, and Philippe I. H. Bastiaens. An Acylation Cycle Regulates Localization and Activity of Palmitoylated Ras Isoforms. *Science*, 307(5716):1746–1752, March 2005.

[47] Rosana D. Meyer, David B. Sacks, and Nader Rahimi. IQGAP1-dependent signaling pathway regulates endothelial cell proliferation and angiogenesis. *PloS One*, 3(12):e3848, 2008.

[48] Suyong Choi and Richard A. Anderson. And Akt-ion! IQGAP1 in control of signaling pathways. *The EMBO journal*, 36(8):967–969, April 2017.

[49] Helen Morrison, Tobias Sperka, Jan Manent, Marco Giovannini, Helmut Ponta, and Peter Herrlich. Merlin/neurofibromatosis type 2 suppresses growth by inhibiting the activation of Ras and Rac. *Cancer Research*, 67(2):520–527, January 2007.

[50] Soung Hoo Jeon, Ju-Yong Yoon, Young-Nyun Park, Woo-Jeong Jeong, Sewoon Kim, Eek-Hoon Jho, Young-Joon Surh, and Kang-Yell Choi. Axin inhibits extracellular signal-regulated kinase pathway by Ras degradation via beta-catenin. *The Journal of Biological Chemistry*, 282(19):14482–14492, May 2007.

[51] Dean E. McNulty, Zhigang Li, Colin D. White, David B. Sacks, and Roland S. Annan. MAPK Scaffold IQGAP1 Binds the EGF Receptor and Modulates Its Activation. *Journal of Biological Chemistry*, 286(17):15010–15021, April 2011.

[52] Mi-Sun Yun, Sung-Eun Kim, Soung Hoo Jeon, Jung-Soo Lee, and Kang-Yell Choi. Both ERK and Wnt/beta-catenin pathways are involved in Wnt3a-induced proliferation. *Journal of Cell Science*, 118(Pt 2):313–322, January 2005.

[53] F Chang, L S Steelman, J T Lee, J G Shelton, P M Navolanic, W L Blalock, R A Franklin, and J A McCubrey. Signal transduction mediated by the Ras/Raf/MEK/ERK pathway from cytokine receptors to transcription factors: Potential targeting for therapeutic intervention. *Leukemia*, 17(7):1263–1293, July 2003.

[54] Permeen Yusoff, Dieu-Hung Lao, Siew Hwa Ong, Esther Sook Miin Wong, Jormay Lim, Ting Ling Lo, Hwei Fen Leong, Chee Wai Fong, and Graeme R. Guy. Sprouty2 inhibits the Ras/MAP kinase pathway by inhibiting the activation of Raf. *The Journal of Biological Chemistry*, 277(5):3195–3201, February 2002.

[55] Suzanne C. Brady, Mathew L. Coleman, June Munro, Stephan M. Feller, Nicolas A. Morrice, and Michael F. Olson. Sprouty2 association with B-Raf is regulated by phosphorylation and kinase conformation. *Cancer Research*, 69(17):6773–6781, September 2009.

[56] C Widmann, S Gibson, and G L Johnson. Caspase-dependent cleavage of signaling proteins during apoptosis. A turn-off mechanism for anti-apoptotic signals. *J Biol Chem*, 273(12):7141–7147, March 1998.

[57] Yohannes A Mebratu, Burton F Dickey, Chris Evans, and Yohannes Tesfaigzi. The BH3-only protein Bik/Blk/Nbk inhibits nuclear translocation of activated ERK1/2 to mediate IFNgamma-induced cell death. *J Cell Biol*, 183(3):429–439, November 2008.

[58] Guoyong Yin, Qinlei Zheng, Chen Yan, and Bradford C. Berk. GIT1 is a scaffold for ERK1/2 activation in focal adhesions. *The Journal of Biological Chemistry*, 280(30):27705–27712, July 2005.

[59] Kenji Ichikawa, Yuji Kubota, Takanori Nakamura, Jane S. Weng, Taichiro Tomida, Haruo Saito, and Mutsuhiro Takekawa. MCRIP1, an ERK substrate, mediates ERK-induced gene silencing during epithelial-mesenchymal transition by regulating the co-repressor CtBP. *Molecular Cell*, 58(1):35–46, April 2015.

[60] Pengda Liu, Wenjian Gan, Y Rebecca Chin, Kohei Ogura, Jianping Guo, Jinfang Zhang, Bin Wang, John Blenis, Lewis C Cantley, Alex Toker, Bing Su, and Wenyi Wei. PtdIns(3,4,5)P3-Dependent Activation of the mTORC2 Kinase Complex. *Cancer discovery*, 5(11):1194–1209, November 2015.

[61] Hui H Zhang, Alex I Lipovsky, Christian C Dibble, Mustafa Sahin, and Brendan D Manning. S6K1 regulates GSK3 under conditions of mTOR-dependent feedback inhibition of Akt. *Mol Cell*, 24(2):185–197, October 2006.

[62] Christian C Dibble, John M Asara, and Brendan D Manning. Characterization of Rictor phosphorylation sites reveals direct regulation of mTOR complex 2 by S6K1. *Molecular and cellular biology*, 29(21):5657–5670, November 2009.

[63] P Rodriguez-Viciana, P H Warne, R Dhand, B Vanhaesebroeck, I Gout, M J Fry, M D Waterfield, and J Downward. Phosphatidylinositol-3-OH kinase as a direct target of Ras. *Nature*, 370(6490):527–532, August 1994.

[64] Surbhi Gupta, Antoine R Ramjaun, Paula Haiko, Yihua Wang, Patricia H Warne, Barbara Nicke, Emma Nye, Gordon Stamp, Kari Alitalo, and Julian Downward. Binding of Ras to phosphoinositide 3-kinase p110alpha is required for Ras-driven tumorigenesis in mice. *Cell*, 129(5):957–968, June 2007.

[65] Long Zhang, Fangfang Zhou, and Peter ten Dijke. Signaling interplay between transforming growth factor-β receptor and PI3K/AKT pathways in cancer. *Trends in Biochemical Sciences*, 38(12):612–620, December 2013.

[66] A Khwaja, P Rodriguez-Viciana, S Wennström, P H Warne, and J Downward. Matrix adhesion and Ras transformation both activate a phosphoinositide 3-OH kinase and protein kinase B/Akt cellular survival pathway. *The EMBO Journal*, 16(10):2783–2793, May 1997.

[67] Yiling Lu, Qinghua Yu, Jue Hui Liu, Jinyi Zhang, Hongwei Wang, Dimpy Koul, John S. McMurray, Xianjun Fang, W.K.Alfred Yung, Kathy A. Siminovitch, and Gordon B. Mills. Src Family Protein-tyrosine Kinases Alter the Function of PTEN to Regulate Phosphatidylinositol 3-Kinase/AKT Cascades. *Journal of Biological Chemistry*, 278(41):40057–40066, October 2003.

[68] A. V. Bakin, A. K. Tomlinson, N. A. Bhowmick, H. L. Moses, and C. L. Arteaga. Phosphatidylinositol 3-kinase function is required for transforming growth factor beta-mediated epithelial to mesenchymal transition and cell migration. *The Journal of Biological Chemistry*, 275(47):36803–36810, November 2000.

[69] Brendan D Manning and Alex Toker. AKT/PKB Signaling: Navigating the Network. *Cell*, 169(3):381–405, April 2017.

[70] Zhiqiang Lin, Pingzhu Zhou, Alexander von Gise, Fei Gu, Qing Ma, Jinghai Chen, Haidong Guo, Pim R. R. van Gorp, Da-Zhi Wang, and William T. Pu. Pi3kcb links Hippo-YAP and PI3K-AKT signaling pathways to promote cardiomyocyte proliferation and survival. *Circulation Research*, 116(1):35–45, January 2015.

[71] Tina L Yuan, Gerburg Wulf, Laura Burga, and Lewis C Cantley. Cell-to-Cell Variability in PI3K Protein Level Regulates PI3K-AKT Pathway Activity in Cell Populations. *Current biology : CB*, 21(3):173–183, February 2011.

[72] Rosaline C-Y Hui, Ana R Gomes, Demetra Constantinidou, Joana R Costa, Christina T Karadedou, Silvia Fernández de Mattos, Matthias P Wymann, Jan J Brosens, Almut Schulze, and Eric W-F Lam. The forkhead transcription factor FOXO3a increases phosphoinositide-3 kinase/Akt activity in drug-resistant leukemic cells through induction of PIK3CA expression. *Molecular and cellular biology*, 28(19):5886–5898, October 2008.

[73] Zixi Wang, Tingting Dang, Tingting Liu, She Chen, Lin Li, Song Huang, and Min Fang. NEDD4L Protein Catalyzes Ubiquitination of PIK3CA Protein and Regulates PI3K-AKT Signaling. *Journal of Biological Chemistry*, 291(33):17467–17477, August 2016.

[74] Nader Chalhoub and Suzanne J. Baker. PTEN and the PI3-Kinase Pathway in Cancer. *Annual Review of Pathology: Mechanisms of Disease*, 4(1):127–150, February 2009.

[75] Maiko Higuchi, Keisuke Onishi, Chikako Kikuchi, and Yukiko Gotoh. Scaffolding function of PAK in the PDK1–Akt pathway. *Nature Cell Biology*, 10(11):1356–1364, November 2008.

[76] Kai Mao, Satoru Kobayashi, Zahara M. Jaffer, Yuan Huang, Paul Volden, Jonathan Chernoff, and Qiangrong Liang. Regulation of Akt/PKB activity by P21-activated kinase in cardiomyocytes. *Journal of Molecular and Cellular Cardiology*, 44(2):429–434, February 2008.

[77] Jer-Yen Yang, Cong S Zong, Weiya Xia, Hirohito Yamaguchi, Qingqing Ding, Xiaoming Xie, Jing-Yu Lang, Chien-Chen Lai, Chun-Ju Chang, Wei-Chien Huang, Hsin Huang, Hsu-Ping Kuo, Dung-Fang Lee, Long-Yuan Li, Huang-Chun Lien, Xiaoyun Cheng, King-Jen Chang, Chwan-Deng Hsiao, Fuu-Jen Tsai, Chang-Hai Tsai, Aysegul A Sahin, William J Muller, Gordon B Mills, Dihua Yu, Gabriel N Hortobagyi, and Mien-Chie Hung. ERK promotes tumorigenesis by inhibiting FOXO3a via MDM2-mediated degradation. *Nat Cell Biol*, 10(2):138–148, February 2008.

[78] Octavian Bucur, Andreea Lucia Stancu, Maria Sinziana Muraru, Armelle Melet, Stefana Maria Petrescu, and Roya Khosravi-Far. PLK1 is a binding partner and a negative regulator of FOXO3 tumor suppressor. *Discoveries (Craiova, Romania)*, 2(2):e16, April 2014.

[79] S Cockcroft and G M Thomas. Inositol-lipid-specific phospholipase C isoenzymes and their differential regulation by receptors. *The Biochemical journal*, 288 (Pt 1)(Pt 1):1–14, November 1992.

[80] H K Kim, J W Kim, A Zilberstein, B Margolis, J G Kim, J Schlessinger, and S G Rhee. PDGF stimulation of inositol phospholipid hydrolysis requires PLC-gamma 1 phosphorylation on tyrosine residues 783 and 1254. *Cell*, 65(3):435–441, May 1991.

[81] W Zhang, R P Trible, M Zhu, S K Liu, C J McGlade, and L E Samelson. Association of Grb2, Gads, and phospholipase C-gamma 1 with phosphorylated LAT tyrosine residues. Effect of LAT tyrosine mutations on T cell angigen receptor-mediated signaling. *J Biol Chem*, 275(30):23355–23361, July 2000.

[82] M Falasca, S K Logan, V P Lehto, G Baccante, M A Lemmon, and J Schlessinger. Activation of phospholipase C gamma by PI 3-kinase-induced PH domain-mediated membrane targeting. *EMBO J*, 17(2):414–422, January 1998.

[83] L E Rameh, S G Rhee, K Spokes, A Kazlauskas, L C Cantley, and L G Cantley. Phosphoinositide 3-kinase regulates phospholipase Cgamma-mediated calcium signaling. *J Biol Chem*, 273(37):23750–23757, September 1998.

[84] Aurelie Gresset, John Sondek, and T Kendall Harden. The phospholipase C isozymes and their regulation. *Sub-cellular biochemistry*, 58(Chapter 3):61–94, 2012.

[85] R H Michell, C J Kirk, L M Jones, C P Downes, and J A Creba. The stimulation of inositol lipid metabolism that accompanies calcium mobilization in stimulated cells: Defined characteristics and unanswered questions. *Philosophical transactions of the Royal Society of London Series B, Biological sciences*, 296(1080):123–138, December 1981.

[86] Albert Escobedo, Tiago Gomes, Eric Aragón, Pau Martín-Malpartida, Lidia Ruiz, and Maria J Macias. Structural basis of the activation and degradation mechanisms of the E3 ubiquitin ligase Nedd4L. *Structure (London, England : 1993)*, 22(10):1446–1457, October 2014.

[87] Robert A. Saxton and David M. Sabatini. mTOR Signaling in Growth, Metabolism, and Disease. *Cell*, 169(2):361–371, April 2017.

[88] Li Ma, Zhenbang Chen, Hediye Erdjument-Bromage, Paul Tempst, and Pier Paolo Pandolfi. Phosphorylation and functional inactivation of TSC2 by Erk implications for tuberous sclerosis and cancer pathogenesis. *Cell*, 121(2):179–193, April 2005.

[89] Philippe P. Roux, Bryan A. Ballif, Rana Anjum, Steven P. Gygi, and John Blenis. Tumor-promoting phorbol esters and activated Ras inactivate the tuberous sclerosis tumor suppressor complex via p90 ribosomal S6 kinase. *Proceedings of the National Academy of Sciences of the United States of America*, 101(37):13489–13494, September 2004.

[90] Ken Inoki, Yong Li, Tianquan Zhu, Jun Wu, and Kun-Liang Guan. TSC2 is phosphorylated and inhibited by Akt and suppresses mTOR signalling. *Nature Cell Biology*, 4(9):648–657, September 2002.

[91] Claudia Wiza, Emmani B. M. Nascimento, and D. Margriet Ouwens. Role of PRAS40 in Akt and mTOR signaling in health and disease. *American Journal of Physiology. Endocrinology and Metabolism*, 302(12):E1453–1460, June 2012.

[92] Emilie Vander Haar, Seong-Il Lee, Sricharan Bandhakavi, Timothy J. Griffin, and Do-Hyung Kim. Insulin signalling to mTOR mediated by the Akt/PKB substrate PRAS40. *Nature Cell Biology*, 9(3):316–323, March 2007.

[93] Bruno D. Fonseca, Ewan M. Smith, Vivian H.-Y. Lee, Carol MacKintosh, and Christopher G. Proud. PRAS40 is a target for mammalian target of rapamycin complex 1 and is required for signaling downstream of this complex. *The Journal of Biological Chemistry*, 282(34):24514–24524, August 2007.

[94] Ken Inoki, Yong Li, Tian Xu, and Kun-Liang Guan. Rheb GTPase is a direct target of TSC2 GAP activity and regulates mTOR signaling. *Genes & Development*, 17(15):1829–1834, August 2003.

[95] Mengling Liu, Christopher J. Clarke, Mohamed F. Salama, Yeon Ja Choi, Lina M. Obeid, and Yusuf A. Hannun. Co-ordinated activation of classical and novel PKC isoforms is required for PMA-induced mTORC1 activation. *PloS One*, 12(9):e0184818, 2017.

[96] Constantinos Demetriades, Monika Plescher, and Aurelio A. Teleman. Lysosomal recruitment of TSC2 is a universal response to cellular stress. *Nature Communications*, 7:10662, February 2016.

[97] Audrey Carriere, Yves Romeo, Hugo A. Acosta-Jaquez, Julie Moreau, Eric Bonneil, Pierre Thibault, Diane C. Fingar, and Philippe P. Roux. ERK1/2 phosphorylate Raptor to promote Ras-dependent activation of mTOR complex 1 (mTORC1). *The Journal of Biological Chemistry*, 286(1):567–577, January 2011.

[98] Sebastian Real, Nathalie Meo-Evoli, Lilia Espada, and Albert Tauler. E2F1 regulates cellular growth by mTORC1 signaling. *PloS One*, 6(1):e16163, January 2011.

[99] Xiaomeng Long, Yenshou Lin, Sara Ortiz-Vega, Kazuyoshi Yonezawa, and Joseph Avruch. Rheb binds and regulates the mTOR kinase. *Current biology: CB*, 15(8):702–713, April 2005.

[100] Francisco Ramírez-Valle, Michelle L. Badura, Steve Braunstein, Manisha Narasimhan, and Robert J. Schneider. Mitotic raptor promotes mTORC1 activity, G(2)/M cell cycle progression, and internal ribosome entry site-mediated mRNA translation. *Molecular and Cellular Biology*, 30(13):3151–3164, July 2010.

[101] Marianne F. James, Sangyeul Han, Carolyn Polizzano, Scott R. Plotkin, Brendan D. Manning, Anat O. Stemmer-Rachamimov, James F. Gusella, and Vijaya Ramesh. NF2/merlin is a novel negative regulator of mTOR complex 1, and activation of mTORC1 is associated with meningioma and schwannoma growth. *Molecular and Cellular Biology*, 29(15):4250–4261, August 2009.

[102] Miguel A. López-Lago, Tomoyo Okada, Miguel M. Murillo, Nick Socci, and Filippo G. Giancotti. Loss of the Tumor Suppressor Gene *NF2* , Encoding Merlin, Constitutively Activates Integrin-Dependent mTORC1 Signaling. *Molecular and Cellular Biology*, 29(15):4235–4249, August 2009.

[103] Ratnal Belapurkar, Maximilian Pfisterer, Jan Dreute, Sebastian Werner, Sven Zukunft, Ingrid Fleming, Michael Kracht, and M. Lienhard Schmitz. A transient increase of HIF-1α during the G1 phase (G1-HIF) ensures cell survival under nutritional stress. *Cell Death & Disease*, 14(7):1–15, July 2023.

[104] Jose M. Orozco, Patrycja A. Krawczyk, Sonia M. Scaria, Andrew L. Cangelosi, Sze Ham Chan, Tenzin Kunchok, Caroline A. Lewis, and David M. Sabatini. Dihydroxyacetone phosphate signals glucose availability to mTORC1. *Nature Metabolism*, 2(9):893–901, September 2020.

[105] R. Martin, C. Desponds, R. O. Eren, M. Quadroni, M. Thome, and N. Fasel. Caspase-mediated cleavage of raptor participates in the inactivation of mTORC1 during cell death. *Cell Death Discovery*, 2:16024, 2016.

[106] Xiaoju Max Ma and John Blenis. Molecular mechanisms of mTOR-mediated translational control. *Nature Reviews. Molecular Cell Biology*, 10(5):307–318, May 2009.

[107] Rohini Dhar, Shalini D. Persaud, Joe R. Mireles, and Alakananda Basu. Proteolytic cleavage of p70 ribosomal S6 kinase by caspase-3 during DNA damage-induced apoptosis. *Biochemistry*, 48(7):1474–1480, February 2009.

[108] Herbert Sizek, Andrew Hamel, Dávid Deritei, Sarah Campbell, and Erzsébet Ravasz Regan. Boolean model of growth signaling, cell cycle and apoptosis predicts the molecular mechanism of aberrant cell cycle progression driven by hyperactive PI3K. *PLoS computational biology*, 15(3):e1006402, March 2019.

[109] Eric Guberman, Hikmet Sherief, and Erzsébet Ravasz Regan. Boolean model of anchorage dependence and contact inhibition points to coordinated inhibition but semi-independent induction of proliferation and migration. *Computational and Structural Biotechnology Journal*, 18:2145–2165, 2020.

[110] Rania Azar, Amandine Alard, Christiane Susini, Corinne Bousquet, and Stéphane Pyronnet. 4E-BP1 is a target of Smad4 essential for TGFbeta-mediated inhibition of cell proliferation. *The EMBO journal*, 28(22):3514–3522, November 2009.

[111] M. Bushell, L. McKendrick, R. U. Jänicke, M. J. Clemens, and S. J. Morley. Caspase-3 is necessary and sufficient for cleavage of protein synthesis eukaryotic initiation factor 4G during apoptosis. *FEBS letters*, 451(3):332–336, May 1999.

[112] Qingqing Ding, Weiya Xia, Jaw-Ching Liu, Jer-Yen Yang, Dung-Fang Lee, Jiahong Xia, Geoffrey Bartholomeusz, Yan Li, Yong Pan, Zheng Li, Ralf C. Bargou, Jun Qin, Chien-Chen Lai, Fuu-Jen Tsai, Chang-Hai Tsai, and Mien-Chie Hung. Erk associates with and primes GSK-3beta for its inactivation resulting in upregulation of beta-catenin. *Molecular Cell*, 19(2):159–170, July 2005.

[113] Laura Saieva, Maria Magdalena Barreca, Chiara Zichittella, Maria Giulia Prado, Marco Tripodi, Riccardo Alessandro, and Alice Conigliaro. Hypoxia-Induced miR-675-5p Supports β-Catenin Nuclear Localization by Regulating GSK3-β Activity in Colorectal Cancer Cell Lines. *International Journal of Molecular Sciences*, 21(11):3832, May 2020.

[114] Chengfu Yuan, Lei Wang, Liang Zhou, and Zheng Fu. The function of FOXO1 in the late phases of the cell cycle is suppressed by PLK1-mediated phosphorylation. *Cell Cycle*, 13(5):807–819, 2014.

[115] Roger R. Gomis, Claudio Alarcón, Wei He, Qiongqing Wang, Joan Seoane, Alex Lash, and Joan Massagué. A FoxO-Smad synexpression group in human keratinocytes. *Proceedings of the National Academy of Sciences of the United States of America*, 103(34):12747–12752, August 2006.

[116] Joan Seoane, Hong-Van Le, Lijian Shen, Stewart A. Anderson, and Joan Massagué. Integration of Smad and forkhead pathways in the control of neuroepithelial and glioblastoma cell proliferation. *Cell*, 117(2):211–223, April 2004.

[117] Fen Hu, Chuan Wang, Jun Du, Wei Sun, Jidong Yan, Dong Mi, Jie Zhang, Yuhuan Qiao, Tianhui Zhu, and Shuang Yang. DeltaEF1 promotes breast cancer cell proliferation through down-regulating p21 expression. *Biochimica Et Biophysica Acta*, 1802(2):301–312, February 2010.

[118] Yang Xi, Sugiko Watanabe, Yuko Hino, Chiyomi Sakamoto, Yuko Nakatsu, Seiji Okada, and Mitsuyoshi Nakao. Hmga1 is differentially expressed and mediates silencing of the CD4/CD8 loci in T cell lineages and leukemic cells. *Cancer Science*, 103(3):439–447, March 2012.

[119] Junko Kamei, Toshihiko Toyofuku, and Masatsugu Hori. Negative regulation of p21 by beta-catenin/TCF signaling: A novel mechanism by which cell adhesion molecules regulate cell proliferation. *Biochemical and Biophysical Research Communications*, 312(2):380–387, December 2003.

[120] Yao Dai and Dietmar Siemann. C-Src is required for hypoxia-induced metastasis-associated functions in prostate cancer cells. *OncoTargets and therapy*, 12:3519–3529, May 2019.

[121] Joan Seoane, Hong-Van Le, and Joan Massagué. Myc suppression of the p21(Cip1) Cdk inhibitor influences the outcome of the p53 response to DNA damage. *Nature*, 419(6908):729–734, October 2002.

[122] Simon Mitchell, Jesse Vargas, and Alexander Hoffmann. Signaling via the NFκB system. *Wiley Interdisciplinary Reviews. Systems Biology and Medicine*, 8(3):227–241, May 2016.

[123] Dong Bai, Lynn Ueno, and Peter K. Vogt. Akt-mediated regulation of NFκB and the essentialness of NFκB for the oncogenicity of PI3K and Akt. *International journal of cancer. Journal international du cancer*, 125(12):2863–2870, December 2009.

[124] Eoin P. Cummins, Edurne Berra, Katrina M. Comerford, Amandine Ginouves, Kathleen T. Fitzgerald, Fergal Seeballuck, Catherine Godson, Jens E. Nielsen, Paul Moynagh, Jacques Pouyssegur, and Cormac T. Taylor. Prolyl hydroxylase-1 negatively regulates IκB kinase-β, giving insight into hypoxia-induced NFκB activity. *Proceedings of the National Academy of Sciences of the United States of America*, 103(48):18154–18159, November 2006.

[125] A. Foryst-Ludwig and M. Naumann. P21-activated kinase 1 activates the nuclear factor kappa B (NF-kappa B)-inducing kinase-Ikappa B kinases NF-kappa B pathway and proinflammatory cytokines in Helicobacter pylori infection. *The Journal of Biological Chemistry*, 275(50):39779–39785, December 2000.

[126] A. Israel. The IKK Complex, a Central Regulator of NF- B Activation. *Cold Spring Harbor Perspectives in Biology*, 2(3):a000158–a000158, March 2010.

[127] Marco Pieraccioli, Francesca Imbastari, Alexey Antonov, Gerry Melino, and Giuseppe Raschellà. Activation of miR200 by c-Myb depends on ZEB1 expression and miR200 promoter methylation. *Cell Cycle (Georgetown, Tex.)*, 12(14):2309–2320, July 2013.

[128] A. Lauder, A. Castellanos, and K. Weston. C-Myb transcription is activated by protein kinase B (PKB) following interleukin 2 stimulation of Tcells and is required for PKB-mediated protection from apoptosis. *Molecular and Cellular Biology*, 21(17):5797–5805, September 2001.

[129] Yoshikazu Takada, Xiaojing Ye, and Scott Simon. The integrins. *Genome Biology*, 8(5):215, 2007.

[130] Martin Alexander Schwartz. Integrins and extracellular matrix in mechanotransduction. *Cold Spring Harbor Perspectives in Biology*, 2(12):a005066, December 2010.

[131] Gregory Hannigan, Armelle A. Troussard, and Shoukat Dedhar. Integrin-linked kinase: A cancer therapeutic target unique among its ILK. *Nature Reviews Cancer*, 5(1):51–63, January 2005.

[132] Satyajit K. Mitra, Daniel A. Hanson, and David D. Schlaepfer. Focal adhesion kinase: In command and control of cell motility. *Nature Reviews Molecular Cell Biology*, 6(1):56–68, January 2005.

[133] François G. Gervais, Nancy A. Thornberry, Salvatore C. Ruffolo, Donald W. Nicholson, and Sophie Roy. Caspases Cleave Focal Adhesion Kinase during Apoptosis to Generate a FRNK-like Polypeptide. *Journal of Biological Chemistry*, 273(27):17102–17108, July 1998.

[134] Y. Yamakita, G. Totsukawa, S. Yamashiro, D. Fry, X. Zhang, S. K. Hanks, and F. Matsumura. Dissociation of FAK/p130(CAS)/c-Src complex during mitosis: Role of mitosis-specific serine phosphorylation of FAK. *The Journal of Cell Biology*, 144(2):315–324, January 1999.

[135] Matthew C. Jones, Janet A. Askari, Jonathan D. Humphries, and Martin J. Humphries. Cell adhesion is regulated by CDK1 during the cell cycle. *Journal of Cell Biology*, 217(9):3203–3218, September 2018.

[136] Satyajit K. Mitra and David D. Schlaepfer. Integrin-regulated FAK-Src signaling in normal and cancer cells. *Current Opinion in Cell Biology*, 18(5):516–523, October 2006.

[137] Paul A. Bromann, Hasan Korkaya, and Sara A. Courtneidge. The interplay between Src family kinases and receptor tyrosine kinases. *Oncogene*, 23(48):7957–7968, October 2004.

[138] Hendrik Ungefroren, Susanne Sebens, Stephanie Groth, Frank Gieseler, and Fred Fändrich. Differential roles of Src in transforming growth factor-ß regulation of growth arrest, epithelial-to-mesenchymal transition and cell migration in pancreatic ductal adenocarcinoma cells. *International Journal of Oncology*, 38(3):797–805, March 2011.

[139] Elisa Giannoni, Francesca Buricchi, Giovanni Raugei, Giampietro Ramponi, and Paola Chiarugi. Intracellular Reactive Oxygen Species Activate Src Tyrosine Kinase during Cell Adhesion and Anchorage-Dependent Cell Growth. *Molecular and Cellular Biology*, 25(15):6391–6403, August 2005.

[140] D. R. Stover, J. Liebetanz, and N. B. Lydon. Cdc2-mediated modulation of pp60c-src activity. *The Journal of Biological Chemistry*, 269(43):26885–26889, October 1994.

[141] Taihei Fukuyama, Hisakazu Ogita, Tomomi Kawakatsu, Tatsuro Fukuhara, Tomohiro Yamada, Tatsuhiro Sato, Kazuya Shimizu, Takeshi Nakamura, Michiyuki Matsuda, and Yoshimi Takai. Involvement of the c-Src-Crk-C3G-Rap1 Signaling in the Nectin-induced Activation of Cdc42 and Formation of Adherens Junctions*. *Journal of Biological Chemistry*, 280(1):815–825, January 2005.

[142] Robert W. McLachlan, Astrid Kraemer, Falak M. Helwani, Eva M. Kovacs, and Alpha S. Yap. E-Cadherin Adhesion Activates c-Src Signaling at Cell–Cell Contacts. *Molecular Biology of the Cell*, 18(8):3214–3223, August 2007.

[143] Yoshimi Takai, Kenji Irie, Kazuya Shimizu, Toshiaki Sakisaka, and Wataru Ikeda. Nectins and nectin-like molecules: Roles in cell adhesion, migration, and polarization. *Cancer Science*, 94(8):655–667, August 2003.

[144] Hisakazu Ogita, Yoshiyuki Rikitake, Jun Miyoshi, and Yoshimi Takai. Cell adhesion molecules nectins and associating proteins: Implications for physiology and pathology. *Proceedings of the Japan Academy, Series B*, 86(6):621–629, 2010.

[145] Yukiko Minami, Wataru Ikeda, Mihoko Kajita, Tsutomu Fujito, Hisayuki Amano, Yoshiyuki Tamaru, Kaori Kuramitsu, Yasuhisa Sakamoto, Morito Monden, and Yoshimi Takai. Necl-5/Poliovirus Receptor Interacts in cis with Integrin $\alpha V\beta 3$ and Regulates Its Clustering and Focal Complex Formation. *Journal of Biological Chemistry*, 282(25):18481–18496, June 2007.

[146] Mihoko Kajita, Wataru Ikeda, Yoshiyuki Tamaru, and Yoshimi Takai. Regulation of platelet-derived growth factor-induced Ras signaling by poliovirus receptor Necl-5 and negative growth regulator Sprouty2. *Genes to Cells: Devoted to Molecular & Cellular Mechanisms*, 12(3):345–357, March 2007.

[147] Jacqueline M. Mason, Debra J. Morrison, M. Albert Basson, and Jonathan D. Licht. Sprouty proteins: Multifaceted negative-feedback regulators of receptor tyrosine kinase signaling. *Trends in Cell Biology*, 16(1):45–54, January 2006.

[148] Ulrike Steinhusen, Jörg Weiske, Volker Badock, Rudolf Tauber, Kurt Bommert, and Otmar Huber. Cleavage and Shedding of E-cadherin after Induction of Apoptosis. *Journal of Biological Chemistry*, 276(7):4972–4980, February 2001.

[149] Xinrui Tian, Zhuola Liu, Bo Niu, Jianlin Zhang, Thian Kui Tan, So Ra Lee, Ye Zhao, David C. H. Harris, and Guoping Zheng. E-Cadherin/ β -Catenin Complex and the Epithelial Barrier. *Journal of Biomedicine and Biotechnology*, 2011:1–6, 2011.

[150] Ulrike Steinhusen, Volker Badock, Andreas Bauer, Jürgen Behrens, Brigitte Wittman-Liebold, Bernd Dörken, and Kurt Bommert. Apoptosis-induced Cleavage of β-Catenin by Caspase-3 Results in Proteolytic Fragments with Reduced Transactivation Potential. *Journal of Biological Chemistry*, 275(21):16345–16353, May 2000.

[151] Benjamin Geiger, Joachim P. Spatz, and Alexander D. Bershadsky. Environmental sensing through focal adhesions. *Nature Reviews Molecular Cell Biology*, 10(1):21–33, January 2009.

[152] Sergey V. Plotnikov, Ana M. Pasapera, Benedikt Sabass, and Clare M. Waterman. Force Fluctuations within Focal Adhesions Mediate ECM-Rigidity Sensing to Guide Directed Cell Migration. *Cell*, 151(7):1513–1527, December 2012.

[153] Elizabeth G Kleinschmidt and David D Schlaepfer. Focal adhesion kinase signaling in unexpected places. *Current Opinion in Cell Biology*, 45:24–30, April 2017.

[154] Ana M. Pasapera, Sergey V. Plotnikov, Robert S. Fischer, Lindsay B. Case, Thomas T. Egelhoff, and Clare M. Waterman. Rac1-dependent phosphorylation and focal adhesion recruitment of myosin IIA regulates migration and mechanosensing. *Current biology: CB*, 25(2):175–186, January 2015.

[155] Inna Kozlova, Aino Ruusala, Oleksandr Voytyuk, Spyros S. Skandalis, and Paraskevi Heldin. IQGAP1 regulates hyaluronan-mediated fibroblast motility and proliferation. *Cellular Signalling*, 24(9):1856–1862, September 2012.

[156] Takashi Kohno, Norifumi Urao, Takashi Ashino, Varadarajan Sudhahar, Hyoe Inomata, Minako Yamaoka-Tojo, Ronald D. McKinney, Tohru Fukai, and Masuko Ushio-Fukai. IQGAP1 links PDGF receptor-β signal to focal adhesions involved in vascular smooth muscle cell migration: Role in neointimal formation after vascular injury. *American Journal of Physiology-Cell Physiology*, 305(6):C591–C600, September 2013.

[157] Sahar Foroutannejad, Nathan Rohner, Michael Reimer, Guim Kwon, and Joseph M. Schober. A novel role for IQGAP1 protein in cell motility through cell retraction. *Biochemical and Biophysical Research Communications*, 448(1):39–44, May 2014.

[158] Giorgia Nardone, Jorge Oliver-De La Cruz, Jan Vrbsky, Cecilia Martini, Jan Pribyl, Petr Skládal, Martin Pešl, Guido Caluori, Stefania Pagliari, Fabiana Martino, Zuzana Maceckova, Marian Hajduch, Andres Sanz-Garcia, Nicola Maria Pugno, Gorazd Bernard Stokin, and Giancarlo Forte. YAP regulates cell mechanics by controlling focal adhesion assembly. *Nature Communications*, 8(1):15321, August 2017.

[159] Mariaceleste Aragona, Tito Panciera, Andrea Manfrin, Stefano Giulitti, Federica Michielin, Nicola Elvassore, Sirio Dupont, and Stefano Piccolo. A mechanical checkpoint controls multicellular growth through YAP/TAZ regulation by actin-processing factors. *Cell*, 154(5):1047–1059, August 2013.

[160] Stacey Lee and Sanjay Kumar. Actomyosin stress fiber mechanosensing in 2D and 3D. *F1000Research*, 5:2261, September 2016.

[161] Ruchan Karaman and Georg Halder. Cell Junctions in Hippo Signaling. *Cold Spring Harbor Perspectives in Biology*, 10(5):a028753, May 2018.

[162] Lily Hoa, Yavuz Kulaberoglu, Ramazan Gundogdu, Dorthe Cook, Merdiye Mavis, Marta Gomez, Valenti Gomez, and Alexander Hergovich. The characterisation of LATS2 kinase regulation in Hippo-YAP signalling. *Cellular Signalling*, 28(5):488–497, May 2016.

[163] Mark R. Silvis, Bridget T. Kreger, Wen-Hui Lien, Olga Klezovitch, G. Marianna Rudakova, Fernando D. Camargo, Dan M. Lantz, John T. Seykora, and Valeri Vasioukhin. α-catenin is a tumor suppressor that controls cell accumulation by regulating the localization and activity of the transcriptional coactivator Yap1. *Science Signaling*, 4(174):ra33, May 2011.

[164] Karin Schlegelmilch, Morvarid Mohseni, Oktay Kirak, Jan Pruszak, J. Renato Rodriguez, Dawang Zhou, Bridget T. Kreger, Valera Vasioukhin, Joseph Avruch, Thijn R. Brummelkamp, and Fernando D. Camargo. Yap1 acts downstream of α-catenin to control epidermal proliferation. *Cell*, 144(5):782–795, March 2011.

[165] Ritu Sarpal, Victoria Yan, Lidia Kazakova, Luka Sheppard, Jessica C. Yu, Rodrigo Fernandez-Gonzalez, and Ulrich Tepass. Role of α-Catenin and its mechanosensing properties in regulating Hippo/YAP-dependent tissue growth. *PLoS genetics*, 15(11):e1008454, November 2019.

[166] Sirio Dupont, Leonardo Morsut, Mariaceleste Aragona, Elena Enzo, Stefano Giulitti, Michelangelo Cordenonsi, Francesca Zanconato, Jimmy Le Digabel, Mattia Forcato, Silvio Bicciato, Nicola Elvassore, and Stefano Piccolo. Role of YAP/TAZ in mechanotransduction. *Nature*, 474(7350):179–183, June 2011.

[167] JinSeok Park, Deok-Ho Kim, Sagar R. Shah, Hong-Nam Kim, null Kshitiz, Peter Kim, Alfredo Quiñones-Hinojosa, and Andre Levchenko. Switch-like enhancement of epithelial-mesenchymal transition by YAP through feedback regulation of WT1 and Rho-family GTPases. *Nature Communications*, 10(1):2797, June 2019.

[168] Yawei Hao, Alex Chun, Kevin Cheung, Babak Rashidi, and Xiaolong Yang. Tumor suppressor LATS1 is a negative regulator of oncogene YAP. *The Journal of Biological Chemistry*, 283(9):5496–5509, February 2008.

[169] Sebastian Mana-Capelli and Dannel McCollum. Angiomotins stimulate LATS kinase autophosphorylation and act as scaffolds that promote Hippo signaling. *Journal of Biological Chemistry*, 293(47):18230–18241, November 2018.

[170] Feng Yin, Jianzhong Yu, Yonggang Zheng, Qian Chen, Nailing Zhang, and Duojia Pan. Spatial Organization of Hippo Signaling at the Plasma Membrane Mediated by the Tumor Suppressor Merlin/NF2. *Cell*, 154(6):1342–1355, September 2013.

[171] Nailing Zhang, Haibo Bai, Karen K. David, Jixin Dong, Yonggang Zheng, Jing Cai, Marco Giovannini, Pentao Liu, Robert A. Anders, and Duojia Pan. The Merlin/NF2 tumor suppressor functions through the YAP oncoprotein to regulate tissue homeostasis in mammals. *Developmental Cell*, 19(1):27–38, July 2010.

[172] Susana Moleirinho, Sany Hoxha, Vinay Mandati, Graziella Curtale, Scott Troutman, Ursula Ehmer, and Joseph L Kissil. Regulation of localization and function of the transcriptional co-activator YAP by angiomotin. *eLife*, 6:e23966, May 2017.

[173] Sagar R. Shah, Nathaniel D. Tippens, JinSeok Park, Ahmed Mohyeldin, Shuyan Wang, Guillermo Vela, Juan C. Martinez-Gutierrez, Seth S. Margolis, Susanne Schmidt, Shuli Xia, Andre Levchenko, and Alfredo Quiñones-Hinojosa. YAP controls cell migration and invasion through a Rho-GTPase switch. Preprint, Cancer Biology, April 2019.

[174] Ishani Dasgupta and Dannel McCollum. Control of cellular responses to mechanical cues through YAP/TAZ regulation. *Journal of Biological Chemistry*, 294(46):17693–17706, November 2019.

[175] L. M. McCaffrey and I. G. Macara. Signaling Pathways in Cell Polarity. *Cold Spring Harbor Perspectives in Biology*, 4(6):a009654–a009654, June 2012.

[176] Mariann Bienz. β-Catenin: A Pivot between Cell Adhesion and Wnt Signalling. *Current Biology*, 15(2):R64–R67, January 2005.

[177] Yong Luo, Mingchuan Li, Xuemei Zuo, Spyridon P. Basourakos, Jiao Zhang, Jiahui Zhao, Yili Han, Yunhua Lin, Yongxing Wang, Yongguang Jiang, and Ling Lan. B-catenin nuclear translocation induced by HIF-1α overexpression leads to the radioresistance of prostate cancer. *International Journal of Oncology*, 52(6):1827–1840, June 2018.

[178] Eunice H Y Chan, Marjaana Nousiainen, Ravindra B Chalamalasetty, Anja Schäfer, Erich A Nigg, and Herman H W Silljé. The Ste20-like kinase Mst2 activates the human large tumor suppressor kinase Lats1. *Oncogene*, 24(12):2076–2086, March 2005.

[179] Maria Praskova, Fan Xia, and Joseph Avruch. MOBKL1A/MOBKL1B Phosphorylation by MST1 and MST2 Inhibits Cell Proliferation. *Current Biology*, 18(5):311–321, March 2008.

[180] Julian Kwan, Anna Sczaniecka, Emad Heidary Arash, Liem Nguyen, Chia-Chun Chen, Srdjana Ratkovic, Olga Klezovitch, Liliana Attisano, Helen McNeill, Andrew Emili, and Valeri Vasioukhin. DLG5 connects cell polarity and Hippo signaling protein networks by linking PAR-1 with MST1/2. *Genes & Development*, 30(24):2696–2709, December 2016.

[181] Yoshikazu Hirate and Hiroshi Sasaki. The role of angiomotin phosphorylation in the Hippo pathway during preimplantation mouse development. *Tissue Barriers*, 2(1):e28127, January 2014.

[182] Chunling Yi, Scott Troutman, Daniela Fera, Anat Stemmer-Rachamimov, Jacqueline L. Avila, Neepa Christian, Nathalie Luna Persson, Akihiko Shimono, David W. Speicher, Ronen Marmorstein, Lars Holmgren, and Joseph L. Kissil. A tight junction-associated Merlin-angiomotin complex mediates Merlin's regulation of mitogenic signaling and tumor suppressive functions. *Cancer Cell*, 19(4):527–540, April 2011.

[183] Xiaoming Dai, Peilu She, Fangtao Chi, Ying Feng, Huan Liu, Daqing Jin, Yiqiang Zhao, Xiaocan Guo, Dandan Jiang, Kun-Liang Guan, Tao P. Zhong, and Bin Zhao. Phosphorylation of Angiomotin by Lats1/2 Kinases Inhibits F-actin Binding, Cell Migration, and Angiogenesis. *Journal of Biological Chemistry*, 288(47):34041–34051, November 2013.

[184] Karen Tumaneng, Karin Schlegelmilch, Ryan C. Russell, Dean Yimlamai, Harihar Basnet, Navin Mahadevan, Julien Fitamant, Nabeel Bardeesy, Fernando D. Camargo, and Kun-Liang Guan. YAP mediates crosstalk between the Hippo and PI(3)K–TOR pathways by suppressing PTEN via miR-29. *Nature Cell Biology*, 14(12):1322–1329, December 2012.

[185] Chien-Yu Chen, Jingyu Chen, Lina He, and Bangyan L. Stiles. PTEN: Tumor Suppressor and Metabolic Regulator. *Frontiers in Endocrinology*, 9:338, July 2018.

[186] J. Y.C. Chow, K. T. Quach, B. L. Cabrera, J. A. Cabral, S. E. Beck, and J. M. Carethers. RAS/ERK modulates TGF -regulated PTEN expression in human pancreatic adenocarcinoma cells. *Carcinogenesis*, 28(11):2321–2327, September 2007.

[187] Stayce E. Beck and John M. Carethers. BMP suppresses PTEN expression via RAS/ERK signaling. *Cancer Biology & Therapy*, 6(8):1319–1323, August 2007.

[188] Yong Wu, Hillary Zhou, Ke Wu, Sangkyu Lee, Ruijin Li, and Xuan Liu. PTEN Phosphorylation and Nuclear Export Mediate Free Fatty Acid-Induced Oxidative Stress. *Antioxidants & Redox Signaling*, 20(9):1382–1395, March 2014.

[189] Helene Maccario, Nevin M. Perera, Lindsay Davidson, C. Peter Downes, and Nick R. Leslie. PTEN is destabilized by phosphorylation on Thr366. *Biochemical Journal*, 405(3):439–444, August 2007.

[190] Xiaoling Tang, Sung-Wuk Jang, Xuerong Wang, Zhixue Liu, Scott M. Bahr, Shi-Yong Sun, Daniel Brat, David H. Gutmann, and Keqiang Ye. Akt phosphorylation regulates the tumour-suppressor merlin through ubiquitination and degradation. *Nature Cell Biology*, 9(10):1199–1207, October 2007.

[191] Isabel Serrano, Paul C. McDonald, Frances Lock, William J. Muller, and Shoukat Dedhar. Inactivation of the Hippo tumour suppressor pathway by integrin-linked kinase. *Nature Communications*, 4:2976, 2013.

[192] Nam-Gyun Kim, Eunjin Koh, Xiao Chen, and Barry M. Gumbiner. E-cadherin mediates contact inhibition of proliferation through Hippo signaling-pathway components. *Proceedings of the National Academy of Sciences of the United States of America*, 108(29):11930–11935, July 2011.

[193] Andrew B. Gladden, Alan M. Hebert, Eveline E. Schneeberger, and Andrea I. McClatchey. The NF2 Tumor Suppressor, Merlin, Regulates Epidermal Development through the Establishment of a Junctional Polarity Complex. *Developmental Cell*, 19(5):727–739, November 2010.

[194] Guang-Hui Xiao, Alexander Beeser, Jonathan Chernoff, and Joseph R. Testa. P21-activated kinase links Rac/Cdc42 signaling to merlin. *The Journal of Biological Chemistry*, 277(2):883–886, January 2002.

[195] Youjun Li, Hao Zhou, Fengzhi Li, Siew Wee Chan, Zhijie Lin, Zhiyi Wei, Zhou Yang, Fusheng Guo, Chun Jye Lim, Wancai Xing, Yuequan Shen, Wanjin Hong, Jiafu Long, and Mingjie Zhang. Angiomotin binding-induced activation of Merlin/NF2 in the Hippo pathway. *Cell Research*, 25(7):801–817, July 2015.

[196] Masaki Fukata, Masato Nakagawa, and Kozo Kaibuchi. Roles of Rho-family GTPases in cell polarisation and directional migration. *Current Opinion in Cell Biology*, 15(5):590–597, October 2003.

[197] Lorena B. Benseñor, Ho-Man Kan, Ningning Wang, Horst Wallrabe, Lance A. Davidson, Ying Cai, Dorothy A. Schafer, and George S. Bloom. IQGAP1 regulates cell motility by linking growth factor signaling to actin assembly. *Journal of Cell Science*, 120(4):658–669, February 2007.

[198] Blagoy Blagoev, Irina Kratchmarova, Shao-En Ong, Mogens Nielsen, Leonard J. Foster, and Matthias Mann. A proteomics strategy to elucidate functional protein-protein interactions applied to EGF signaling. *Nature Biotechnology*, 21(3):315–318, March 2003.

[199] Takashi Watanabe, Shujie Wang, Jun Noritake, Kazumasa Sato, Masaki Fukata, Mikito Takefuji, Masato Nakagawa, Nanae Izumi, Tetsu Akiyama, and Kozo Kaibuchi. Interaction with IQGAP1 Links APC to Rac1, Cdc42, and Actin Filaments during Cell Polarization and Migration. *Developmental Cell*, 7(6):871–883, December 2004.

[200] Takashi Watanabe, Jun Noritake, and Kozo Kaibuchi. Roles of IQGAP1 in cell polarization and migration. *Novartis Foundation Symposium*, 269:92–101; discussion 101–105, 223–230, 2005.

[201] Davide Franco, Mirko Klingauf, Martin Bednarzik, Marco Cecchini, Vartan Kurtcuoglu, Jens Gobrecht, Dimos Poulikakos, and Aldo Ferrari. Control of initial endothelial spreading by topographic activation of focal adhesion kinase. *Soft Matter*, 7(16):7313, 2011.

[202] Huiyi Tang, Xueer Wang, Min Zhang, Yuan Yan, Simin Huang, Jiahao Ji, Jinfu Xu, Yijia Zhang, Yongjie Cai, Bobo Yang, Wenqi Lan, Mianbo Huang, and Lin Zhang. MicroRNA-200b/c-3p regulate epithelial plasticity and inhibit cutaneous wound healing by modulating TGF-β-mediated RAC1 signaling. *Cell Death & Disease*, 11(10):931, October 2020.

[203] Young-Ho Ahn, Don L. Gibbons, Deepavali Chakravarti, Chad J. Creighton, Zain H. Rizvi, Henry P. Adams, Alexander Pertsemlidis, Philip A. Gregory, Josephine A. Wright, Gregory J. Goodall, Elsa R.

Flores, and Jonathan M. Kurie. ZEB1 drives prometastatic actin cytoskeletal remodeling by downregulating miR-34a expression. *The Journal of Clinical Investigation*, 122(9):3170–3183, September 2012.

[204] Khameeka N. Kitt and W. James Nelson. Rapid suppression of activated Rac1 by cadherins and nectins during de novo cell-cell adhesion. *PloS One*, 6(3):e17841, March 2011.

[205] Yong Ho Bae, Keeley L. Mui, Bernadette Y. Hsu, Shu-Lin Liu, Alexandra Cretu, Ziba Razinia, Tina Xu, Ellen Puré, and Richard K. Assoian. A FAK-Cas-Rac-Lamellipodin Signaling Module Transduces Extracellular Matrix Stiffness into Mechanosensitive Cell Cycling. *Science Signaling*, 7(330), June 2014.

[206] G. Zhu, Z. Fan, M. Ding, H. Zhang, L. Mu, Y. Ding, Y. Zhang, B. Jia, L. Chen, Z. Chang, and W. Wu. An EGFR/PI3K/AKT axis promotes accumulation of the Rac1-GEF Tiam1 that is critical in EGFR-driven tumorigenesis. *Oncogene*, 34(49):5971–5982, December 2015.

[207] Wataru Ikeda, Shigeki Kakunaga, Kyoji Takekuni, Tatsushi Shingai, Keiko Satoh, Koji Morimoto, Masakazu Takeuchi, Toshio Imai, and Yoshimi Takai. Nectin-like Molecule-5/Tage4 Enhances Cell Migration in an Integrin-dependent, Nectin-3-independent Manner. *Journal of Biological Chemistry*, 279(17):18015–18025, April 2004.

[208] Aude Cannet, Susanne Schmidt, Bénédicte Delaval, and Anne Debant. Identification of a mitotic Rac-GEF, Trio, that counteracts MgcRacGAP function during cytokinesis. *Molecular Biology of the Cell*, 25(25):4063–4071, December 2014.

[209] C. M. Waterman-Storer, R. A. Worthylake, B. P. Liu, K. Burridge, and E. D. Salmon. Microtubule growth activates Rac1 to promote lamellipodial protrusion in fibroblasts. *Nature Cell Biology*, 1(1):45–50, May 1999.

[210] Baolin Zhang, Yaqin Zhang, and Emily Shacter. Caspase 3-Mediated Inactivation of Rac GTPases Promotes Drug-Induced Apoptosis in Human Lymphoma Cells. *Molecular and Cellular Biology*, 23(16):5716–5725, August 2003.

[211] Meiwan Cao, Yayoi Shikama, Michiko Anzai, and Junko Kimura. Impaired Neutrophil Migration Resulting from Mir-34a and Mir-155 Overexpressed in Neutrophils from Myelodysplastic Syndrome Patients. *Blood*, 126(23):999–999, December 2015.

[212] Dahong Yao, Chenyang Li, Muhammad Shahid Riaz Rajoka, Zhendan He, Jian Huang, Jinhui Wang, and Jin Zhang. P21-Activated Kinase 1: Emerging biological functions and potential therapeutic targets in Cancer. *Theranostics*, 10(21):9741–9766, 2020.

[213] Ulla G. Knaus, Yan Wang, Abina M. Reilly, Dawn Warnock, and Janis H. Jackson. Structural Requirements for PAK Activation by Rac GTPases. *Journal of Biological Chemistry*, 273(34):21512–21518, August 1998.

[214] Chiara De Pascalis and Sandrine Etienne-Manneville. Single and collective cell migration: The mechanics of adhesions. *Molecular Biology of the Cell*, 28(14):1833–1846, July 2017.

[215] Gary M. Bokoch. Biology of the p21-Activated Kinases. *Annual Review of Biochemistry*, 72(1):743–781, June 2003.

[216] Ning Ma, Erqian Xu, Qing Luo, and Guanbin Song. Rac1: A Regulator of Cell Migration and a Potential Target for Cancer Therapy. *Molecules (Basel, Switzerland)*, 28(7):2976, March 2023.

[217] Helge Siemens, Rene Jackstadt, Sabine Hünten, Antje Menssen, Ursula Götz, and Heiko Hermeking. miR-34 and SNAIL form a double-negative feedback loop to regulate epithelial-mesenchymal transitions. *Cell Cycle*, 10(24):4256–4271, December 2011.

[218] Haoxuan Zheng, Wenjing Li, Yadong Wang, Zhizhong Liu, Yidong Cai, Tingting Xie, Meng Shi, Zhiqing Wang, and Bo Jiang. Glycogen synthase kinase-3 beta regulates Snail and β-catenin expression during Fas-induced epithelial-mesenchymal transition in gastrointestinal cancer. *European Journal of Cancer (Oxford, England: 1990)*, 49(12):2734–2746, August 2013.

[219] Chengyin Min, Sean F. Eddy, David H. Sherr, and Gail E. Sonenshein. NF-κB and epithelial to mesenchymal transition of cancer. *Journal of Cellular Biochemistry*, 104(3):733–744, 2008.

[220] Zhibo Yang, Suresh Rayala, Diep Nguyen, Ratna K. Vadlamudi, Shiuan Chen, and Rakesh Kumar. Pak1 phosphorylation of snail, a master regulator of epithelial-to-mesenchyme transition, modulates snail's subcellular localization and functions. *Cancer Research*, 65(8):3179–3184, April 2005.

[221] Xingbo Xu, Xiaoying Tan, Björn Tampe, Elisa Sanchez, Michael Zeisberg, and Elisabeth M. Zeisberg. Snail Is a Direct Target of Hypoxia-inducible Factor 1α (HIF1α) in Hypoxia-induced Endothelial to Mesenchymal Transition of Human Coronary Endothelial Cells. *The Journal of Biological Chemistry*, 290(27):16653–16664, July 2015.

[222] Ying Xi, Ying Wei, Barbara Sennino, Arnau Ulsamer, Irene Kwan, Alexis N. Brumwell, Kevin Tan, Manish K. Aghi, Donald M. McDonald, David M. Jablons, and Harold A. Chapman. Identification of pY654-β-catenin as a critical co-factor in hypoxia-inducible factor-1α signaling and tumor responses to hypoxia. *Oncogene*, 32(42):10.1038/onc.2012.530, October 2013.

[223] Sylvie Thuault, E.-Jean Tan, Hector Peinado, Amparo Cano, Carl-Henrik Heldin, and Aristidis Moustakas. HMGA2 and Smads co-regulate SNAIL1 expression during induction of epithelial-to-mesenchymal transition. *The Journal of Biological Chemistry*, 283(48):33437–33446, November 2008.

[224] Jing Zhong, Chang Liu, Ya-jun Chen, Qing-hai Zhang, Jing Yang, Xuan Kang, Si-Rui Chen, Ge-bo Wen, Xu-yu Zu, and Ren-xian Cao. The association between S100A13 and HMGA1 in the modulation of thyroid cancer proliferation and invasion. *Journal of Translational Medicine*, 14:80, March 2016.

[225] Wei Xu, Hao Liu, Zhi-Gang Liu, Hong-Sheng Wang, Fan Zhang, Hao Wang, Ji Zhang, Jing-Jing Chen, Hong-Jun Huang, Yuan Tan, Meng-Ting Cao, Jun Du, Qiu-Gui Zhang, and Guan-Min Jiang. Histone deacetylase inhibitors upregulate Snail via Smad2/3 phosphorylation and stabilization of Snail to promote metastasis of hepatoma cells. *Cancer Letters*, 420:1–13, April 2018.

[226] Peijing Zhang, Yongkun Wei, Li Wang, Bisrat G. Debeb, Yuan Yuan, Jinsong Zhang, Jingsong Yuan, Min Wang, Dahu Chen, Yutong Sun, Wendy A. Woodward, Yongqing Liu, Douglas C. Dean, Han Liang, Ye Hu, K. Kian Ang, Mien-Chie Hung, Junjie Chen, and Li Ma. ATM-mediated stabilization of ZEB1 promotes DNA damage response and radioresistance through CHK1. *Nature Cell Biology*, 16(9):864–875, September 2014.

[227] Nam Hee Kim, Sang Hyun Song, Yun Hee Choi, Kyu Ho Hwang, Jun Seop Yun, Hyeeun Song, So Young Cha, Sue Bean Cho, Inhan Lee, Hyun Sil Kim, and Jong In Yook. Competing Endogenous RNA of Snail and Zeb1 UTR in Therapeutic Resistance of Colorectal Cancer. *International Journal of Molecular Sciences*, 22(17):9589, September 2021.

[228] Xinghai Yang, Lei Li, Quan Huang, Wei Xu, Xiaopan Cai, Jishen Zhang, Wangjun Yan, Dianwen Song, Tielong Liu, Wang Zhou, Zhenxi Li, Cheng Yang, Yongyan Dang, and Jianru Xiao. Wnt signaling through Snail1 and Zeb1 regulates bone metastasis in lung cancer. *American Journal of Cancer Research*, 5(2):748–755, 2015.

[229] Larion Santiago, Garrett Daniels, Dongwen Wang, Fang-Ming Deng, and Peng Lee. Wnt signaling pathway protein LEF1 in cancer, as a biomarker for prognosis and a target for treatment. *American Journal of Cancer Research*, 7(6):1389–1406, 2017.

[230] Kangsun Yun, Yoo Duk Choi, Jong Hee Nam, Zeeyoung Park, and Sin-Hyeog Im. NF-κB regulates Lef1 gene expression in chondrocytes. *Biochemical and Biophysical Research Communications*, 357(3):589–595, June 2007.

[231] Ali Nawshad and Elizabeth D. Hay. TGFbeta3 signaling activates transcription of the LEF1 gene to induce epithelial mesenchymal transformation during mouse palate development. *The Journal of Cell Biology*, 163(6):1291–1301, December 2003.

[232] Pedro Rosmaninho, Susanne Mükusch, Valerio Piscopo, Vera Teixeira, Alexandre ASF Raposo, Rolf Warta, Romina Bennewitz, Yeman Tang, Christel Herold-Mende, Stefano Stifani, Stefan Momma, and Diogo S Castro. Zeb1 potentiates genome-wide gene transcription with Lef1 to promote glioblastoma cell invasion. *The EMBO Journal*, 37(15), August 2018.

[233] Thad Sharp, Jianbo Wang, Xiao Li, Huojun Cao, Shan Gao, Myriam Moreno, and Brad A. Amendt. A pituitary homeobox 2 (Pitx2):microRNA-200a-3p:β-catenin pathway converts mesenchymal cells to amelogenin-expressing dental epithelial cells. *The Journal of Biological Chemistry*, 289(39):27327–27341, September 2014.

[234] Can G. Pham, Concetta Bubici, Francesca Zazzeroni, James R. Knabb, Salvatore Papa, Christian Kuntzen, and Guido Franzoso. Upregulation of Twist-1 by NF-κB Blocks Cytotoxicity Induced by Chemotherapeutic Drugs. *Molecular and Cellular Biology*, 27(11):3920–3935, June 2007.

[235] E.-Jean Tan, Sylvie Thuault, Laia Caja, Tea Carletti, Carl-Henrik Heldin, and Aristidis Moustakas. Regulation of transcription factor Twist expression by the DNA architectural protein high mobility group A2 during epithelial-to-mesenchymal transition. *The Journal of Biological Chemistry*, 287(10):7134–7145, March 2012.

[236] S. Demontis, C. Rigo, S. Piccinin, M. Mizzau, M. Sonego, M. Fabris, C. Brancolini, and R. Maestro. Twist is substrate for caspase cleavage and proteasome-mediated degradation. *Cell Death & Differentiation*, 13(2):335–345, February 2006.

[237] Yingzhun Chen, Xiaoguang Zhao, Jian Sun, Wei Su, Lu Zhang, Yingnan Li, Yingqi Liu, Lijia Zhang, Yanjie Lu, Hongli Shan, and Haihai Liang. YAP1/Twist promotes fibroblast activation and lung fibrosis that conferred by miR-15a loss in IPF. *Cell Death and Differentiation*, 26(9):1832–1844, September 2019.

[238] Natàlia Dave, Sandra Guaita-Esteruelas, Susana Gutarra, Àlex Frias, Manuel Beltran, Sandra Peiró, and Antonio García de Herreros. Functional cooperation between Snail1 and twist in the regulation of ZEB1 expression during epithelial to mesenchymal transition. *The Journal of Biological Chemistry*, 286(14):12024–12032, April 2011.

[239] Martina Rembold, Lucia Ciglar, J. Omar Yáñez-Cuna, Robert P. Zinzen, Charles Girardot, Ankit Jain, Michael A. Welte, Alexander Stark, Maria Leptin, and Eileen E. M. Furlong. A conserved role for Snail as a potentiator of active transcription. *Genes & Development*, 28(2):167–181, January 2014.

[240] Saber Imani, Chunli Wei, Jingliang Cheng, Md. Asaduzzaman Khan, Shangyi Fu, Luquan Yang, Mousumi Tania, Xianqin Zhang, Xiuli Xiao, Xianning Zhang, and Junjiang Fu. MicroRNA-34a targets epithelial to mesenchymal transition-inducing transcription factors (EMT-TFs) and inhibits breast cancer cell migration and invasion. *Oncotarget*, 8(13):21362–21379, March 2017.

[241] Amy Belton, Alexander Gabrovsky, Young Kyung Bae, Ray Reeves, Christine Iacobuzio-Donahue, David L. Huso, and Linda M. S. Resar. HMGA1 induces intestinal polyposis in transgenic mice and drives tumor progression and stem cell properties in colon cancer cells. *PloS One*, 7(1):e30034, 2012.

[242] Wenhui Zhou, Kayla M. Gross, and Charlotte Kuperwasser. Molecular regulation of Snai2 in development and disease. *Journal of Cell Science*, 132(23):jcs235127, December 2019.

[243] Esmeralda Casas, Jihoon Kim, Andrés Bendesky, Lucila Ohno-Machado, Cecily J. Wolfe, and Jing Yang. Snail2 is an Essential Mediator of Twist1-Induced Epithelial Mesenchymal Transition and Metastasis. *Cancer Research*, 71(1):245–254, January 2011.

[244] Makoto Saegusa, Miki Hashimura, Takeshi Kuwata, and Isao Okayasu. Requirement of the Akt/beta-catenin pathway for uterine carcinosarcoma genesis, modulating E-cadherin expression through the transactivation of slug. *The American Journal of Pathology*, 174(6):2107–2115, June 2009.

[245] Elisabetta Lambertini, Tiziana Franceschetti, Elena Torreggiani, Letizia Penolazzi, Antonio Pastore, Stefano Pelucchi, Roberto Gambari, and Roberta Piva. SLUG: A new target of lymphoid enhancer factor-1 in human osteoblasts. *BMC Molecular Biology*, 11(1):13, December 2010.

[246] Brijesh Kumar, Mallikarjunachari V. N. Uppuladinne, Vinod Jani, Uddhavesh Sonavane, Rajendra R. Joshi, and Sharmila A. Bapat. Auto-regulation of Slug mediates its activity during epithelial to mesenchymal transition. *Biochimica Et Biophysica Acta*, 1849(9):1209–1218, September 2015.

[247] Tsuyoshi Morita, Taira Mayanagi, and Kenji Sobue. Dual roles of myocardin-related transcription factors in epithelial–mesenchymal transition via slug induction and actin remodeling. *Journal of Cell Biology*, 179(5):1027–1042, December 2007.

[248] Shashank Gandhi, Erica J. Hutchins, Krystyna Maruszko, Jong H. Park, Matthew Thomson, and Marianne E. Bronner. Bimodal function of chromatin remodeler Hmga1 in neural crest induction and Wnt-dependent emigration. *eLife*, 9:e57779, September 2020.

[249] Stanislav Drápela, Jan Bouchal, Mohit Kumar Jolly, Zoran Culig, and Karel Souček. ZEB1: A Critical Regulator of Cell Plasticity, DNA Damage Response, and Therapy Resistance. *Frontiers in Molecular Biosciences*, 7:36, March 2020.

[250] Christian Wels, Shripad Joshi, Petra Koefinger, Helmut Bergler, and Helmut Schaider. Transcriptional Activation of ZEB1 by Slug Leads to Cooperative Regulation of the EMT like Phenotype in Melanoma. *The Journal of investigative dermatology*, 131(9):1877–1885, September 2011.

[251] Ester Sánchez-Tilló, Oriol de Barrios, Laura Siles, Miriam Cuatrecasas, Antoni Castells, and Antonio Postigo. β-catenin/TCF4 complex induces the epithelial-to-mesenchymal transition (EMT)-activator ZEB1 to regulate tumor invasiveness. *Proceedings of the National Academy of Sciences of the United States of America*, 108(48):19204–19209, November 2011.

[252] X. Li, S. Roslan, C. N. Johnstone, J. A. Wright, C. P. Bracken, M. Anderson, A. G. Bert, L. A. Selth, R. L. Anderson, G. J. Goodall, P. A. Gregory, and Y. Khew-Goodall. MiR-200 can repress breast cancer metastasis through ZEB1-independent but moesin-dependent pathways. *Oncogene*, 33(31):4077–4088, July 2014.

[253] Alexandra C. Title, Sue-Jean Hong, Nuno D. Pires, Lynn Hasenöhrl, Svenja Godbersen, Nadine Stokar-Regenscheit, David P. Bartel, and Markus Stoffel. Genetic dissection of the miR-200-Zeb1 axis reveals its importance in tumor differentiation and invasion. *Nature Communications*, 9(1):4671, November 2018.

[254] Mohit Kumar Jolly, Marcelo Boareto, Bin Huang, Dongya Jia, Mingyang Lu, Eshel Ben-Jacob, José N. Onuchic, and Herbert Levine. Implications of the Hybrid Epithelial/Mesenchymal Phenotype in Metastasis. *Frontiers in Oncology*, 5:155, 2015.

[255] Mingyang Lu, Mohit Kumar Jolly, Herbert Levine, José N. Onuchic, and Eshel Ben-Jacob. MicroRNA-based regulation of epithelial-hybrid-mesenchymal fate determination. *Proceedings of the National Academy of Sciences of the United States of America*, 110(45):18144–18149, November 2013.

[256] Jingyu Zhang, Xiao-Jun Tian, Hang Zhang, Yue Teng, Ruoyan Li, Fan Bai, Subbiah Elankumaran, and Jianhua Xing. TGF-β-induced epithelial-to-mesenchymal transition proceeds through stepwise activation of multiple feedback loops. *Science Signaling*, 7(345):ra91, September 2014.

[257] Sun-Mi Park, Arti B. Gaur, Ernst Lengyel, and Marcus E. Peter. The miR-200 family determines the epithelial phenotype of cancer cells by targeting the E-cadherin repressors ZEB1 and ZEB2. *Genes & Development*, 22(7):894–907, April 2008.

[258] Wenjing Zhang, Xinpeng Shi, Ying Peng, Meiyan Wu, Pei Zhang, Ruyi Xie, Yao Wu, Qingqing Yan, Side Liu, and Jide Wang. HIF-1α Promotes Epithelial-Mesenchymal Transition and Metastasis through Direct Regulation of ZEB1 in Colorectal Cancer. *PLOS ONE*, 10(6):e0129603, June 2015.

[259] M. Nishita, M. K. Hashimoto, S. Ogata, M. N. Laurent, N. Ueno, H. Shibuya, and K. W. Cho. Interaction between Wnt and TGF-beta signalling pathways during formation of Spemann's organizer. *Nature*, 403(6771):781–785, February 2000.

[260] Ya Chung Tian and Aled Owain Phillips. Interaction between the transforming growth factor-beta type II receptor/Smad pathway and beta-catenin during transforming growth factor-beta1-mediated adherens junction disassembly. *The American Journal of Pathology*, 160(5):1619–1628, May 2002.

[261] Nam Hee Kim, Hyun Sil Kim, Nam-Gyun Kim, Inhan Lee, Hyung-Seok Choi, Xiao-Yan Li, Shi Eun Kang, So Young Cha, Joo Kyung Ryu, Jung Min Na, Changbum Park, Kunhong Kim, Sanghyuk Lee, Barry M. Gumbiner, Jong In Yook, and Stephen J. Weiss. P53 and microRNA-34 are suppressors of canonical Wnt signaling. *Science Signaling*, 4(197):ra71, November 2011.

[262] Chunsheng Kang. MicroRNA-200a suppresses the Wnt/β-catenin signaling pathway by interacting with β-catenin. *International Journal of Oncology*, December 2011.

[263] Chun Shik Park, Sung Il Kim, Mi Su Lee, Cho-Ya Youn, Dae Joong Kim, Eek-Hoon Jho, and Woo Keun Song. Modulation of beta-catenin phosphorylation/degradation by cyclin-dependent kinase 2. *The Journal of Biological Chemistry*, 279(19):19592–19599, May 2004.

[264] Wakako Kobayashi and Masayuki Ozawa. The transcription factor LEF-1 induces an epithelial-mesenchymal transition in MDCK cells independent of β-catenin. *Biochemical and Biophysical Research Communications*, 442(1-2):133–138, December 2013.

[265] Jianguo Ai, Feng Zhao, and Xiaowen Zhou. HMGA1 Aggravates Oxidative Stress Injury and Inflammatory Responses in IL-1β-Induced Primary Chondrocytes through the JMJD3/ZEB1 Axis. *International Archives of Allergy and Immunology*, 184(3):279–290, 2023.

[266] Damian Medici, Elizabeth D. Hay, and Bjorn R. Olsen. Snail and Slug promote epithelial-mesenchymal transition through beta-catenin-T-cell factor-4-dependent expression of transforming growth factor-beta3. *Molecular Biology of the Cell*, 19(11):4875–4887, November 2008.

[267] Wafa Bouaziz, Johanna Sigaux, Dominique Modrowski, Claire-Sophie Devignes, Thomas Funck-Brentano, Pascal Richette, Hang-Korng Ea, Sylvain Provot, Martine Cohen-Solal, and Eric Haÿ. Interaction of HIF1α and β-catenin inhibits matrix metalloproteinase 13 expression and prevents cartilage damage in mice. *Proceedings of the National Academy of Sciences*, 113(19):5453–5458, May 2016.

[268] Masuko Katoh and Masaru Katoh. Integrative genomic analyses of ZEB2: Transcriptional regulation of ZEB2 based on SMADs, ETS1, HIF1alpha, POU/OCT, and NF-kappaB. *International Journal of Oncology*, 34(6):1737–1742, June 2009.

[269] Bethany M. Bush, Ashton T. Brock, Jiayue A. Deng, Ronald A. Nelson, and Takita Felder Sumter. The Wnt/β-catenin/T-cell factor 4 pathway up-regulates high-mobility group A1 expression in colon cancer. *Cell Biochemistry and Function*, 31(3):228–236, April 2013.

[270] Yu Zeng, Tianshi Que, Jie Lin, Zhengming Zhan, Anqi Xu, Zhiyong Wu, Cheng Xie, Jie Luo, Shengfeng Ding, Hao Long, Xian Zhang, and Ye Song. Oncogenic ZEB2/miR-637/HMGA1 signaling axis targeting vimentin promotes the malignant phenotype of glioma. *Molecular Therapy. Nucleic Acids*, 23:769–782, March 2021.

[271] Stephanie McMahon, Francine Grondin, Patrick P. McDonald, Darren E. Richard, and Claire M. Dubois. Hypoxia-enhanced expression of the proprotein convertase furin is mediated by hypoxia-inducible factor-1: Impact on the bioactivation of proproteins. *The Journal of Biological Chemistry*, 280(8):6561–6569, February 2005.

[272] Jianheng Peng, Xiaolin Wang, Liang Ran, Junlong Song, Rong Luo, and Yonghong Wang. Hypoxia-Inducible Factor 1α Regulates the Transforming Growth Factor $B1$/SMAD Family Member 3 Pathway to Promote Breast Cancer Progression. *Journal of Breast Cancer*, 21(3):259–266, September 2018.

[273] Martin Lowe, Jon D. Lane, Philip G. Woodman, and Victoria J. Allan. Caspase-mediated cleavage of syntaxin 5 and giantin accompanies inhibition of secretory traffic during apoptosis. *Journal of Cell Science*, 117(Pt 7):1139–1150, March 2004.

[274] Jung-Yoon Yoo, Bon Jeong Ku, Tae Hoon Kim, Jong Il Ahn, Ji Yeon Ahn, Woo Sub Yang, Jeong Mook Lim, Maketo M. Taketo, Jung-Ho Shin, and Jae-Wook Jeong. β-catenin activates TGF-β-induced epithelial-mesenchymal transition in adenomyosis. *Experimental & Molecular Medicine*, 52(10):1754–1765, October 2020.

[275] Lu Zhang, Yi Liao, and Liling Tang. MicroRNA-34 family: A potential tumor suppressor and therapeutic candidate in cancer. *Journal of Experimental & Clinical Cancer Research*, 38(1):53, December 2019.

[276] Dongsong Nie, Jiewen Fu, Hanchun Chen, Jingliang Cheng, and Junjiang Fu. Roles of MicroRNA-34a in Epithelial to Mesenchymal Transition, Competing Endogenous RNA Sponging and Its Therapeutic Potential. *International Journal of Molecular Sciences*, 20(4):861, February 2019.

[277] Perry S. Mongroo and Anil K. Rustgi. The role of the miR-200 family in epithelial-mesenchymal transition. *Cancer Biology & Therapy*, 10(3):219–222, August 2010.

[278] Xiao Ling Li, Toshifumi Hara, Youngeun Choi, Murugan Subramanian, Princy Francis, Sven Bilke, Robert L. Walker, Marbin Pineda, Yuelin Zhu, Yuan Yang, Ji Luo, Lalage M. Wakefield, Thomas Brabletz, Ben Ho Park, Sudha Sharma, Dipanjan Chowdhury, Paul S. Meltzer, and Ashish Lal. A p21-ZEB1 complex inhibits epithelial-mesenchymal transition through the microRNA 183-96-182 cluster. *Molecular and Cellular Biology*, 34(3):533–550, February 2014.

[279] Ulrike Burk, Jörg Schubert, Ulrich Wellner, Otto Schmalhofer, Elizabeth Vincan, Simone Spaderna, and Thomas Brabletz. A reciprocal repression between ZEB1 and members of the miR-200 family promotes EMT and invasion in cancer cells. *EMBO reports*, 9(6):582–589, June 2008.

[280] Takeshi Haraguchi, Masayuki Kondo, Ryo Uchikawa, Kazuyoshi Kobayashi, Hiroaki Hiramatsu, Kyousuke Kobayashi, Ung Weng Chit, Takanobu Shimizu, and Hideo Iba. Dynamics and plasticity of the epithelial to mesenchymal transition induced by miR-200 family inhibition. *Scientific Reports*, 6(1):21117, February 2016.

[281] Jennifer G Gill, Ellen M Langer, R Coleman Lindsley, Mi Cai, Theresa L Murphy, Michael Kyba, and Kenneth M Murphy. Snail and the microRNA-200 Family Act in Opposition to Regulate Epithelial-to-Mesenchymal Transition and Germ Layer Fate Restriction in Differentiating ESCs. *Stem Cells (Dayton, Ohio)*, 29(5):764–776, May 2011.

[282] Michèle Moes, Antony Le Béchec, Isaac Crespo, Christina Laurini, Aliaksandr Halavatyi, Guillaume Vetter, Antonio Del Sol, and Evelyne Friederich. A novel network integrating a miRNA-203/SNAI1 feedback loop which regulates epithelial to mesenchymal transition. *PloS One*, 7(4):e35440, 2012.

[283] Rui Neves, Christina Scheel, Sandra Weinhold, Ellen Honisch, Katharina M. Iwaniuk, Hans-Ingo Trompeter, Dieter Niederacher, Peter Wernet, Simeon Santourlidis, and Markus Uhrberg. Role of DNA methylation in miR-200c/141 cluster silencing in invasive breast cancer cells. *BMC Research Notes*, 3(1):219, August 2010.

[284] Noriko Funato and Hiromi Yanagisawa. TBX1 targets the miR-200-ZEB2 axis to induce epithelial differentiation and inhibit stem cell properties. *Scientific Reports*, 12(1):20188, November 2022.

[285] Cameron P. Bracken, Philip A. Gregory, Natasha Kolesnikoff, Andrew G. Bert, Jun Wang, M. Frances Shannon, and Gregory J. Goodall. A Double-Negative Feedback Loop between ZEB1-SIP1 and the microRNA-200 Family Regulates Epithelial-Mesenchymal Transition. *Cancer Research*, 68(19):7846–7854, October 2008.

[286] Takuya Shirakihara, Masao Saitoh, and Kohei Miyazono. Differential regulation of epithelial and mesenchymal markers by deltaEF1 proteins in epithelial mesenchymal transition induced by TGF-beta. *Molecular Biology of the Cell*, 18(9):3533–3544, September 2007.

[287] Victoria Bolós, Hector Peinado, Mirna A. Pérez-Moreno, Mario F. Fraga, Manel Esteller, and Amparo Cano. The transcription factor Slug represses E-cadherin expression and induces epithelial to mesenchymal transitions: A comparison with Snail and E47 repressors. *Journal of Cell Science*, 116(Pt 3):499–511, February 2003.

[288] Hector Peinado, Francisco Portillo, and Amparo Cano. Transcriptional regulation of cadherins during development and carcinogenesis. *The International Journal of Developmental Biology*, 48(5-6):365–375, 2004.

[289] Farhad Vesuna, Paul van Diest, Ji Hshiung Chen, and Venu Raman. Twist is a transcriptional repressor of E-cadherin gene expression in breast cancer. *Biochemical and Biophysical Research Communications*, 367(2):235–241, March 2008.

[290] Jane S. Weng, Takanori Nakamura, Hisashi Moriizumi, Hiroshi Takano, Ryoji Yao, and Mutsuhiro Takekawa. MCRIP1 promotes the expression of lung-surfactant proteins in mice by disrupting CtBP-mediated epigenetic gene silencing. *Communications Biology*, 2:227, June 2019.

[291] M. L. Grooteclaes and S. M. Frisch. Evidence for a function of CtBP in epithelial gene regulation and anoikis. *Oncogene*, 19(33):3823–3828, August 2000.

[292] E. Sánchez-Tilló, A. Lázaro, R. Torrent, M. Cuatrecasas, E. C. Vaquero, A. Castells, P. Engel, and A. Postigo. ZEB1 represses E-cadherin and induces an EMT by recruiting the SWI/SNF chromatin-remodeling protein BRG1. *Oncogene*, 29(24):3490–3500, June 2010.

[293] J. Comijn, G. Berx, P. Vermassen, K. Verschueren, L. van Grunsven, E. Bruyneel, M. Mareel, D. Huylebroeck, and F. van Roy. The two-handed E box binding zinc finger protein SIP1 downregulates E-cadherin and induces invasion. *Molecular Cell*, 7(6):1267–1278, June 2001.

[294] K. Wesley Overton, Sabrina L. Spencer, William L. Noderer, Tobias Meyer, and Clifford L. Wang. Basal p21 controls population heterogeneity in cycling and quiescent cell cycle states. *Proceedings of the National Academy of Sciences of the United States of America*, 111(41):E4386–4393, October 2014.

[295] Andrei L. Gartel, Xin Ye, Eugene Goufman, Pavel Shianov, Nissim Hay, Feridoon Najmabadi, and Angela L. Tyner. Myc represses the p21(WAF1/CIP1) promoter and interacts with Sp1/Sp3. *Proceedings of the National Academy of Sciences of the United States of America*, 98(8):4510–4515, April 2001.

[296] Zhimin Lu and Tony Hunter. Ubiquitylation and proteasomal degradation of the p21(Cip1), p27(Kip1) and p57(Kip2) CDK inhibitors. *Cell Cycle (Georgetown, Tex.)*, 9(12):2342–2352, June 2010.

[297] J. L. Gervais, P. Seth, and H. Zhang. Cleavage of CDK inhibitor p21(Cip1/Waf1) by caspases is an early event during DNA damage-induced apoptosis. *The Journal of Biological Chemistry*, 273(30):19207–19212, July 1998.

[298] Olivier Coqueret. New roles for p21 and p27 cell-cycle inhibitors: A function for each cell compartment? *Trends in cell biology*, 13(2):65–70, 2003.

[299] J William Harbour, Robin X Luo, Angeline Dei Santi, Antonio A Postigo, and Douglas C Dean. Cdk phosphorylation triggers sequential intramolecular interactions that progressively block Rb functions as cells move through G1. *Cell*, 98(6):859–869, 1999.

[300] Bela Novak and John J Tyson. A model for restriction point control of the mammalian cell cycle. *Journal of Theoretical Biology*, 230(4):563–579, October 2004.

[301] Yoichi Taya. RB kinases and RB-binding proteins: New points of view. *Trends in biochemical sciences*, 22(1):14–17, 1997.

[302] JY Kato, Hitoshi Matsushime, Scott W Hiebert, Mark E Ewen, and Charles J Sherr. Direct binding of cyclin D to the retinoblastoma gene product (pRb) and pRb phosphorylation by the cyclin D-dependent kinase CDK4. *Genes and Development*, 7:331–331, 1993.

[303] Mark E Ewen, Hayla K Sluss, Charles J Sherr, Hitoshi Matsushime, Jun-ya Kato, and David M Livingston. Functional interactions of the retinoblastoma protein with mammalian D-type cyclins. *Cell*, 73(3):487–497, 1993.

[304] Philip W Hinds, Sibylle Mittnacht, Vjekoslav Dulic, Andrew Arnold, Steven I Reed, and Robert A Weinberg. Regulation of retinoblastoma protein functions by ectopic expression of human cyclins. *Cell*, 70(6):993–1006, 1992.

[305] C. L. Fattman, S. M. Delach, Q. P. Dou, and D. E. Johnson. Sequential two-step cleavage of the retinoblastoma protein by caspase-3/-7 during etoposide-induced apoptosis. *Oncogene*, 20(23):2918–2926, May 2001.

[306] Noriko Ishida, Taichi Hara, Takumi Kamura, Minoru Yoshida, Keiko Nakayama, and Keiichi I. Nakayama. Phosphorylation of p27Kip1 on serine 10 is required for its binding to CRM1 and nuclear export. *The Journal of Biological Chemistry*, 277(17):14355–14358, April 2002.

[307] A. Faure, A. Naldi, C. Chaouiya, and D. Thieffry. Dynamical analysis of a generic Boolean model for the control of the mammalian cell cycle. *Bioinformatics*, 22(14):e124–e131, July 2006.

[308] R. H. Medema, G. J. Kops, J. L. Bos, and B. M. Burgering. AFX-like Forkhead transcription factors mediate cell-cycle regulation by Ras and PKB through p27kip1. *Nature*, 404(6779):782–787, April 2000.

[309] Shigeki Kakunaga, Wataru Ikeda, Tatsushi Shingai, Tsutomu Fujito, Akio Yamada, Yukiko Minami, Toshio Imai, and Yoshimi Takai. Enhancement of Serum- and Platelet-derived Growth Factor-induced Cell Proliferation by Necl-5/Tage4/Poliovirus Receptor/CD155 through the Ras-Raf-MEK-ERK Signaling. *Journal of Biological Chemistry*, 279(35):36419–36425, August 2004.

[310] D Müller, C Bouchard, B Rudolph, P Steiner, I Stuckmann, R Saffrich, W Ansorge, W Huttner, and M Eilers. Cdk2-dependent phosphorylation of p27 facilitates its Myc-induced release from cyclin E/cdk2 complexes. *Oncogene*, 15(21):2561–2576, November 1997.

[311] Robert J Sheaff, Mark Groudine, Matthew Gordon, James M Roberts, and Bruce E Clurman. Cyclin E-CDK2 is a regulator of p27Kip1. *Genes & development*, 11(11):1464–1478, 1997.

[312] Alessia Montagnoli, Francesca Fiore, Esther Eytan, Andrea C Carrano, Giulio F Draetta, Avram Hershko, and Michele Pagano. Ubiquitination of p27 is regulated by Cdk-dependent phosphorylation and trimeric complex formation. *Genes & development*, 13(9):1181–1189, 1999.

[313] B. Eymin, O. Sordet, N. Droin, B. Munsch, M. Haugg, M. Van de Craen, P. Vandenabeele, and E. Solary. Caspase-induced proteolysis of the cyclin-dependent kinase inhibitor p27Kip1 mediates its anti-apoptotic activity. *Oncogene*, 18(34):4839–4847, August 1999.

[314] B. Levkau, H. Koyama, E. W. Raines, B. E. Clurman, B. Herren, K. Orth, J. M. Roberts, and R. Ross. Cleavage of p21Cip1/Waf1 and p27Kip1 mediates apoptosis in endothelial cells through activation of Cdk2: Role of a caspase cascade. *Molecular Cell*, 1(4):553–563, March 1998.

[315] Xiyan Chen, Weiting Gu, Qi Wang, Xucheng Fu, Ying Wang, Xin Xu, and Yong Wen. C-MYC and BCL-2 mediate YAP-regulated tumorigenesis in OSCC. *Oncotarget*, 9(1):668–679, January 2018.

[316] Hui Li, Zhenglan Huang, Miao Gao, Ningshu Huang, Zhenhong Luo, Huawei Shen, Xin Wang, Teng Wang, Jing Hu, and Wenli Feng. Inhibition of YAP suppresses CML cell proliferation and enhances efficacy of imatinib in vitro and in vivo. *Journal of experimental & clinical cancer research: CR*, 35(1):134, September 2016.

[317] C. R. Chen, Y. Kang, and J. Massagué. Defective repression of c-myc in breast cancer cells: A loss at the core of the transforming growth factor beta growth arrest program. *Proceedings of the National Academy of Sciences of the United States of America*, 98(3):992–999, January 2001.

[318] R. Sears, F. Nuckolls, E. Haura, Y. Taya, K. Tamai, and J. R. Nevins. Multiple Ras-dependent phosphorylation pathways regulate Myc protein stability. *Genes & Development*, 14(19):2501–2514, October 2000.

[319] MF Roussel, JN Davis, JL Cleveland, J Ghysdael, and SW Hiebert. Dual control of myc expression through a single DNA binding site targeted by ets family proteins and E2F-1. *Oncogene*, 9(2):405–415, 1994.

[320] Minori Koshiji, Yukio Kageyama, Erin A Pete, Izumi Horikawa, J Carl Barrett, and L Eric Huang. HIF-1α induces cell cycle arrest by functionally counteracting Myc. *The EMBO Journal*, 23(9):1949–1956, May 2004.

[321] B. Lutterbach and S. R. Hann. Hierarchical phosphorylation at N-terminal transformation-sensitive sites in c-Myc protein is regulated by mitogens and in mitosis. *Molecular and Cellular Biology*, 14(8):5510–5522, August 1994.

[322] Chen-Ju Lin, Abba Malina, and Jerry Pelletier. C-Myc and eIF4F constitute a feedforward loop that regulates cell growth: Implications for anticancer therapy. *Cancer Research*, 69(19):7491–7494, October 2009.

[323] Markus Welcker, Amir Orian, Jianping Jin, Jonathan E. Grim, Jonathan A. Grim, J. Wade Harper, Robert N. Eisenman, and Bruce E. Clurman. The Fbw7 tumor suppressor regulates glycogen synthase kinase 3 phosphorylation-dependent c-Myc protein degradation. *Proceedings of the National Academy of Sciences of the United States of America*, 101(24):9085–9090, June 2004.

[324] Olivier Ferrigno, François Lallemand, Franck Verrecchia, Sébastien L'Hoste, Jacques Camonis, Azeddine Atfi, and Alain Mauviel. Yes-associated protein (YAP65) interacts with Smad7 and potentiates its inhibitory activity against TGF-beta/Smad signaling. *Oncogene*, 21(32):4879–4884, July 2002.

[325] Abderrahmane Kaidi, Ann Caroline Williams, and Christos Paraskeva. Interaction between beta-catenin and HIF-1 promotes cellular adaptation to hypoxia. *Nature Cell Biology*, 9(2):210–217, February 2007.

[326] Meera Shah, Sherri A. Rennoll, Wesley M. Raup-Konsavage, and Gregory S. Yochum. A dynamic exchange of TCF3 and TCF4 transcription factors controls MYC expression in colorectal cancer cells. *Cell Cycle (Georgetown, Tex.)*, 14(3):323–332, 2015.

[327] E. Batsché, M. Lipp, and C. Cremisi. Transcriptional repression and activation in the same cell type of the human c-MYC promoter by the retinoblastoma gene protein: Antagonisation of both effects by SV40 T antigen. *Oncogene*, 9(8):2235–2243, August 1994.

[328] F Oswald, H Lovec, T Möröy, and M Lipp. E2F-dependent regulation of human MYC: Trans-activation by cyclins D1 and A overrides tumour suppressor protein functions. *Oncogene*, 9(7):2029–2036, July 1994.

[329] K Thalmeier, H Synovzik, R Mertz, EL Winnacker, and M Lipp. Nuclear factor E2F mediates basic transcription and trans-activation by E1a of the human MYC promoter. *Genes & development*, 3(4):527–536, 1989.

[330] JY Leung, GL Ehmann, PH Giangrande, and JR Nevins. A role for Myc in facilitating transcription activation by E2F1. *Oncogene*, 27(30):4172–4179, 2008.

[331] J. A. Diehl, M. Cheng, M. F. Roussel, and C. J. Sherr. Glycogen synthase kinase-3beta regulates cyclin D1 proteolysis and subcellular localization. *Genes & Development*, 12(22):3499–3511, November 1998.

[332] T. Mizuno, H. Murakami, M. Fujii, F. Ishiguro, I. Tanaka, Y. Kondo, S. Akatsuka, S. Toyokuni, K. Yokoi, H. Osada, and Y. Sekido. YAP induces malignant mesothelioma cell proliferation by upregulating transcription of cell cycle-promoting genes. *Oncogene*, 31(49):5117–5122, December 2012.

[333] J Wade Harper, Stephen J Elledge, Khandan Keyomarsi, Brian Dynlacht, Li-Huei Tsai, Pumin Zhang, Steven Dobrowolski, Connell Bai, Lisa Connell-Crowley, and Eric Swindell. Inhibition of cyclin-dependent kinases by p21. *Molecular biology of the cell*, 6(4):387–400, 1995.

[334] Yue Xiong, Gregory J Hannon, Hui Zhang, David Casso, Ryuji Kobayashi, and David Beach. P21 is a universal inhibitor of cyclin kinases. *nature*, 366(6456):701–704, 1993.

[335] Srikumar P Chellappan, Scott Hiebert, Maria Mudryj, Jonathan M Horowitz, and Joseph R Nevins. The E2F transcription factor is a cellular target for the RB protein. *Cell*, 65(6):1053–1061, 1991.

[336] Masahiro Hitomi and Dennis W Stacey. Cyclin D1 production in cycling cells depends on ras in a cell-cycle-specific manner. *Current biology*, 9(19):1075–S2, 1999.

[337] Huseyin Aktas, Hong Cai, and Geoffrey M Cooper. Ras links growth factor signaling to the cell cycle machinery via regulation of cyclin D1 and the Cdk inhibitor p27KIP1. *Molecular and cellular biology*, 17(7):3850–3857, 1997.

[338] Jasmine I Daksis, Richard Y Lu, Linda M Facchini, Wilson W Marhin, and LJ Penn. Myc induces cyclin D1 expression in the absence of de novo protein synthesis and links mitogen-stimulated signal transduction to the cell cycle. *Oncogene*, 9(12):3635–3645, 1994.

[339] MK Mateyak, AJ Obaya, and JM Sedivy. C-Myc regulates cyclin D-Cdk4 and-Cdk6 activity but affects cell cycle progression at multiple independent points. *Molecular and cellular biology*, 19(7):4672–4683, 1999.

[340] Itaru Matsumura, Hirokazu Tanaka, and Yuzuru Kanakura. E2F1 and c-Myc in cell growth and death. *Cell Cycle*, 2(4):332–335, 2003.

[341] Zhi-yi Guo, Xiao-hui Hao, Fei-Fei Tan, Xin Pei, Li-Mei Shang, Xue-lian Jiang, and Fang Yang. The elements of human cyclin D1 promoter and regulation involved. *Clinical epigenetics*, 2(2):63–76, 2011.

[342] J Fan and J R Bertino. Functional roles of E2F in cell cycle regulation. *Oncogene*, 14(10):1191–1200, March 1997.

[343] Dennis W Stacey. Three Observations That Have Changed Our Understanding of Cyclin D1 and p27 in Cell Cycle Control. *Genes & cancer*, 1(12):1189–1199, December 2010.

[344] J A Diehl, F Zindy, and C J Sherr. Inhibition of cyclin D1 phosphorylation on threonine-286 prevents its rapid degradation via the ubiquitin-proteasome pathway. *Genes & Development*, 11(8):957–972, April 1997.

[345] Wantae Kim, Yong Suk Cho, Xiaohui Wang, Ogyi Park, Xueyan Ma, Hanjun Kim, Wenjian Gan, Eek-Hoon Jho, Boksik Cha, Yun-Ji Jeung, Lei Zhang, Bin Gao, Wenyi Wei, Jin Jiang, Kyung-Sook Chung, and Yingzi Yang. Hippo signaling is intrinsically regulated during cell cycle progression by APC/CCdh1. *Proceedings of the National Academy of Sciences of the United States of America*, 116(19):9423–9432, May 2019.

[346] S J Weintraub, C A Prater, and D C Dean. Retinoblastoma protein switches the E2F site from positive to negative element. *Nature*, 358(6383):259–261, July 1992.

[347] Gustavo Leone, James DeGregori, Rosalie Sears, Laszlo Jakoi, and Joseph R Nevins. Myc and Ras collaborate in inducing accumulation of active cyclin E/Cdk2 and E2F. *Nature*, 387(6631):422–426, 1997.

[348] Hirokazu Tanaka, Itaru Matsumura, Sachiko Ezoe, Yusuke Satoh, Toshiyuki Sakamaki, Chris Albanese, Takashi Machii, Richard G. Pestell, and Yuzuru Kanakura. E2F1 and c-Myc potentiate apoptosis through inhibition of NF-kappaB activity that facilitates MnSOD-mediated ROS elimination. *Molecular Cell*, 9(5):1017–1029, May 2002.

[349] Peng Dong, Manoj V Maddali, Jaydeep K Srimani, François Thélot, Joseph R Nevins, Bernard Mathey-Prevot, and Lingchong You. Division of labour between Myc and G1 cyclins in cell cycle commitment and pace control. *Nature Communications*, 5:4750, 2014.

[350] David G Johnson, Kiyoshi Ohtani, and Joseph R Nevins. Autoregulatory control of E2F1 expression in response to positive and negative regulators of cell cycle progression. *Genes & Development*, 8(13):1514–1525, 1994.

[351] W Krek, M E Ewen, S Shirodkar, Z Arany, W G Kaelin, and D M Livingston. Negative regulation of the growth-promoting transcription factor E2F-1 by a stably bound cyclin A-dependent protein kinase. *Cell*, 78(1):161–172, July 1994.

[352] M Xu, K A Sheppard, C Y Peng, A S Yee, and H Piwnica-Worms. Cyclin A/CDK2 binds directly to E2F-1 and inhibits the DNA-binding activity of E2F-1/DP-1 by phosphorylation. *Molecular and cellular biology*, 14(12):8420–8431, December 1994.

[353] Kristian Helin. Regulation of cell proliferation by the E2F transcription factors. *Current opinion in genetics & development*, 8(1):28–35, 1998.

[354] Kasturi Mitra, Christian Wunder, Badrinath Roysam, Gang Lin, and Jennifer Lippincott-Schwartz. A hyperfused mitochondrial state achieved at G1-S regulates cyclin E buildup and entry into S phase. *Proceedings of the National Academy of Sciences of the United States of America*, 106(29):11960–11965, July 2009.

[355] Kiyoshi Ohtani, James Degregori, and JOSEPH R Nevins. Regulation of the cyclin E gene by transcription factor E2F1. *Proceedings of the National Academy of Sciences*, 92(26):12146–12150, 1995.

[356] Cara L Lunn, John C Chrivia, and Joseph J Baldassare. Activation of Cdk2/Cyclin E complexes is dependent on the origin of replication licensing factor Cdc6 in mammalian cells. *Cell Cycle*, 9(22):4533–4541, November 2010.

[357] Yan Geng, Young-Mi Lee, Markus Welcker, Jherek Swanger, Agnieszka Zagozdzon, Joel D. Winer, James M. Roberts, Philipp Kaldis, Bruce E. Clurman, and Piotr Sicinski. Kinase-independent function of cyclin E. *Molecular Cell*, 25(1):127–139, January 2007.

[358] Claus Storgaard Sørensen, Randi G Syljuåsen, Jacob Falck, Tine Schroeder, Lars Rönnstrand, Kum Kum Khanna, Bin-Bing Zhou, Jiri Bartek, and Jiri Lukas. Chk1 regulates the S phase checkpoint by coupling the physiological turnover and ionizing radiation-induced accelerated proteolysis of Cdc25A. *Cancer cell*, 3(3):247–258, March 2003.

[359] Suparna Mazumder, Bendi Gong, Quan Chen, Judith A. Drazba, Jeffrey C. Buchsbaum, and Alexandru Almasan. Proteolytic cleavage of cyclin E leads to inactivation of associated kinase activity and amplification of apoptosis in hematopoietic cells. *Molecular and Cellular Biology*, 22(7):2398–2409, April 2002.

[360] S. J. Yeung, J. Pan, and M.-H. Lee. Roles of p53, MYC and HIF-1 in regulating glycolysis - the seventh hallmark of cancer. *Cellular and molecular life sciences: CMLS*, 65(24):3981–3999, December 2008.

[361] K. Ohtani, J. DeGregori, G. Leone, D. R. Herendeen, T. J. Kelly, and J. R. Nevins. Expression of the HsOrc1 gene, a human ORC1 homolog, is regulated by cell proliferation via the E2F transcription factor. *Molecular and Cellular Biology*, 16(12):6977–6984, December 1996.

[362] Melvin L. DePamphilis, J. Julian Blow, Soma Ghosh, Tapas Saha, Kohji Noguchi, and Alex Vassilev. Regulating the licensing of DNA replication origins in metazoa. *Current Opinion in Cell Biology*, 18(3):231–239, June 2006.

[363] Z. Yan, J. DeGregori, R. Shohet, G. Leone, B. Stillman, J. R. Nevins, and R. S. Williams. Cdc6 is regulated by E2F and is essential for DNA replication in mammalian cells. *Proceedings of the National Academy of Sciences of the United States of America*, 95(7):3603–3608, March 1998.

[364] Hyungshin Yim and Raymond L. Erikson. Cell division cycle 6, a mitotic substrate of polo-like kinase 1, regulates chromosomal segregation mediated by cyclin-dependent kinase 1 and separase. *Proceedings of the National Academy of Sciences of the United States of America*, 107(46):19742–19747, November 2010.

[365] B. O. Petersen, J. Lukas, C. S. Sørensen, J. Bartek, and K. Helin. Phosphorylation of mammalian CDC6 by cyclin A/CDK2 regulates its subcellular localization. *The EMBO journal*, 18(2):396–410, January 1999.

[366] Cristina Pelizon, Fabrizio d'Adda di Fagagna, Lorena Farrace, and Ronald A. Laskey. Human replication protein Cdc6 is selectively cleaved by caspase 3 during apoptosis. *EMBO reports*, 3(8):780–784, August 2002.

[367] Kenichi Yoshida and Ituro Inoue. Regulation of Geminin and Cdt1 expression by E2F transcription factors. *Oncogene*, 23(21):3802–3812, May 2004.

[368] Taras Valovka, Manuela Schönfeld, Philipp Raffeiner, Kathrin Breuker, Theresia Dunzendorfer-Matt, Markus Hartl, and Klaus Bister. Transcriptional control of DNA replication licensing by Myc. *Scientific Reports*, 3:3444, December 2013.

[369] Ken-ichiro Yanagi, Takeshi Mizuno, Zhiying You, and Fumio Hanaoka. Mouse geminin inhibits not only Cdt1-MCM6 interactions but also a novel intrinsic Cdt1 DNA binding activity. *The Journal of Biological Chemistry*, 277(43):40871–40880, October 2002.

[370] Linda Clijsters, Janneke Ogink, and Rob Wolthuis. The spindle checkpoint, APC/C(Cdc20), and APC/C(Cdh1) play distinct roles in connecting mitosis to S phase. *J Cell Biol*, 201(7):1013–1026, June 2013.

[371] Irene García-Higuera, Eusebio Manchado, Pierre Dubus, Marta Cañamero, Juan Méndez, Sergio Moreno, and Marcos Malumbres. Genomic stability and tumour suppression by the APC/C cofactor Cdh1. *Nature Cell Biology*, 10(7):802–811, July 2008.

[372] K. E. Knudsen, A. F. Fribourg, M. W. Strobeck, J. M. Blanchard, and E. S. Knudsen. Cyclin A is a functional target of retinoblastoma tumor suppressor protein-mediated cell cycle arrest. *The Journal of Biological Chemistry*, 274(39):27632–27641, September 1999.

[373] Il-Man Kim, Timothy Ackerson, Sneha Ramakrishna, Maria Tretiakova, I.-Ching Wang, Tanya V. Kalin, Michael L. Major, Galina A. Gusarova, Helena M. Yoder, Robert H. Costa, and Vladimir V. Kalinichenko. The Forkhead Box m1 transcription factor stimulates the proliferation of tumor cells during development of lung cancer. *Cancer Research*, 66(4):2153–2161, February 2006.

[374] Tanya V. Kalin, I.-Ching Wang, Timothy J. Ackerson, Michael L. Major, Carol J. Detrisac, Vladimir V. Kalinichenko, Alexander Lyubimov, and Robert H. Costa. Increased levels of the FoxM1 transcription factor accelerate development and progression of prostate carcinomas in both TRAMP and LADY transgenic mice. *Cancer Research*, 66(3):1712–1720, February 2006.

[375] Jamila Laoukili, Matthijs R. H. Kooistra, Alexandra Brás, Jos Kauw, Ron M. Kerkhoven, Ashby Morrison, Hans Clevers, and René H. Medema. FoxM1 is required for execution of the mitotic programme and chromosome stability. *Nature Cell Biology*, 7(2):126–136, February 2005.

[376] Mónica Alvarez-Fernández, Vincentius A. Halim, Lenno Krenning, Melinda Aprelia, Shabaz Mohammed, Albert J. Heck, and René H. Medema. Recovery from a DNA-damage-induced G2 arrest requires Cdk-dependent activation of FoxM1. *EMBO reports*, 11(6):452–458, June 2010.

[377] Moe Tategu, Hiroki Nakagawa, Kaori Sasaki, Rieko Yamauchi, Sota Sekimachi, Yuka Suita, Naoko Watanabe, and Kenichi Yoshid. Transcriptional regulation of human polo-like kinases and early mitotic inhibitor. *Journal of Genetics and Genomics = Yi Chuan Xue Bao*, 35(4):215–224, April 2008.

[378] Jerry Y. Hsu, Julie D. R. Reimann, Claus S. Sørensen, Jiri Lukas, and Peter K. Jackson. E2F-dependent accumulation of hEmi1 regulates S phase entry by inhibiting APC(Cdh1). *Nature Cell Biology*, 4(5):358–366, May 2002.

[379] Jinho Lee, Jin Ah Kim, Valerie Barbier, Arun Fotedar, and Rati Fotedar. DNA damage triggers p21WAF1-dependent Emi1 down-regulation that maintains G2 arrest. *Molecular Biology of the Cell*, 20(7):1891–1902, April 2009.

[380] David V. Hansen, Alexander V. Loktev, Kenneth H. Ban, and Peter K. Jackson. Plk1 regulates activation of the anaphase promoting complex by phosphorylating and triggering SCFbetaTrCP-dependent destruction of the APC Inhibitor Emi1. *Molecular Biology of the Cell*, 15(12):5623–5634, December 2004.

[381] Huafeng Pan, Yudi Zhu, Wei Wei, Siliang Shao, and Xin Rui. Transcription factor FoxM1 is the downstream target of c-Myc and contributes to the development of prostate cancer. *World Journal of Surgical Oncology*, 16(1):59, March 2018.

[382] Juliane M. Lüscher-Firzlaff, Richard Lilischkis, and Bernhard Lüscher. Regulation of the transcription factor FOXM1c by Cyclin E/CDK2. *FEBS letters*, 580(7):1716–1722, March 2006.

[383] Con Sullivan, Youhong Liu, Jingjing Shen, Adam Curtis, Christina Newman, Janet M. Hock, and Xiong Li. Novel interactions between FOXM1 and CDC25A regulate the cell cycle. *PloS One*, 7(12):e51277, 2012.

[384] Michael L. Major, Rita Lepe, and Robert H. Costa. Forkhead box M1B transcriptional activity requires binding of Cdk-cyclin complexes for phosphorylation-dependent recruitment of p300/CBP coactivators. *Molecular and Cellular Biology*, 24(7):2649–2661, April 2004.

[385] Zheng Fu, Liviu Malureanu, Jun Huang, Wei Wang, Hao Li, Jan M. van Deursen, Donald J. Tindall, and Junjie Chen. Plk1-dependent phosphorylation of FoxM1 regulates a transcriptional programme required for mitotic progression. *Nature Cell Biology*, 10(9):1076–1082, September 2008.

[386] Jamila Laoukili, Monica Alvarez, Lars A. T. Meijer, Marie Stahl, Shabaz Mohammed, Livio Kleij, Albert J. R. Heck, and René H. Medema. Activation of FoxM1 during G2 requires cyclin A/Cdk-dependent relief of autorepression by the FoxM1 N-terminal domain. *Molecular and Cellular Biology*, 28(9):3076–3087, May 2008.

[387] Tiebang Kang, Yongkun Wei, Yuchi Honaker, Hiroshi Yamaguchi, Ettore Appella, Mien-Chie Hung, and Helen Piwnica-Worms. GSK-3 beta targets Cdc25A for ubiquitin-mediated proteolysis, and GSK-3 beta inactivation correlates with Cdc25A overproduction in human cancers. *Cancer Cell*, 13(1):36–47, January 2008.

[388] E. Vigo, H. Müller, E. Prosperini, G. Hateboer, P. Cartwright, M. C. Moroni, and K. Helin. CDC25A phosphatase is a target of E2F and is required for efficient E2F-induced S phase. *Molecular and Cellular Biology*, 19(9):6379–6395, September 1999.

[389] L. Wu, E. C. Goodwin, L. K. Naeger, E. Vigo, K. Galaktionov, K. Helin, and D. DiMaio. E2F-Rb complexes assemble and inhibit cdc25A transcription in cervical carcinoma cells following repression of human papillomavirus oncogene expression. *Molecular and Cellular Biology*, 20(19):7059–7067, October 2000.

[390] I Hoffmann, G Draetta, and E Karsenti. Activation of the phosphatase activity of human cdc25A by a cdk2-cyclin E dependent phosphorylation at the G1/S transition. *EMBO J*, 13(18):4302–4310, September 1994.

[391] Laurent Mazzolini, Anaïs Broban, Carine Froment, Odile Burlet-Schiltz, Arnaud Besson, Stéphane Manenti, and Christine Dozier. Phosphorylation of CDC25A on SER283 in late S/G2 by CDK/cyclin complexes accelerates mitotic entry. *Cell Cycle (Georgetown, Tex.)*, 15(20):2742–2752, October 2016.

[392] Niels Mailand, Alexandre V. Podtelejnikov, Anja Groth, Matthias Mann, Jiri Bartek, and Jiri Lukas. Regulation of G(2)/M events by Cdc25A through phosphorylation-dependent modulation of its stability. *The EMBO journal*, 21(21):5911–5920, November 2002.

[393] Maddalena Donzelli, Massimo Squatrito, Dvora Ganoth, Avram Hershko, Michele Pagano, and Giulio F Draetta. Dual mode of degradation of Cdc25 A phosphatase. *The EMBO journal*, 21(18):4875–4884, 2002.

[394] Eusebio Manchado, Manuel Eguren, and Marcos Malumbres. The anaphase-promoting complex/cyclosome (APC/C): Cell-cycle-dependent and -independent functions. *Biochem Soc Trans*, 38(Pt 1):65–71, February 2010.

[395] Mei-Shya Chen, Christine E. Ryan, and Helen Piwnica-Worms. Chk1 kinase negatively regulates mitotic function of Cdc25A phosphatase through 14-3-3 binding. *Molecular and Cellular Biology*, 23(21):7488–7497, November 2003.

[396] Ida Blomberg and Ingrid Hoffmann. Ectopic expression of Cdc25A accelerates the G1/S transition and leads to premature activation of cyclin E-and cyclin A-dependent kinases. *Molecular and cellular biology*, 19(9):6183–6194, 1999.

[397] Yuichi J. Machida and Anindya Dutta. The APC/C inhibitor, Emi1, is essential for prevention of rereplication. *Genes & Development*, 21(2):184–194, January 2007.

[398] J. D. Reimann, E. Freed, J. Y. Hsu, E. R. Kramer, J. M. Peters, and P. K. Jackson. Emi1 is a mitotic regulator that interacts with Cdc20 and inhibits the anaphase promoting complex. *Cell*, 105(5):645–655, June 2001.

[399] J. D. Reimann, B. E. Gardner, F. Margottin-Goguet, and P. K. Jackson. Emi1 regulates the anaphase-promoting complex by a different mechanism than Mad2 proteins. *Genes & Development*, 15(24):3278–3285, December 2001.

[400] Yuko Katsuno, Ayumi Suzuki, Kazuto Sugimura, Katsuzumi Okumura, Doaa H Zineldeen, Midori Shimada, Hiroyuki Niida, Takeshi Mizuno, Fumio Hanaoka, and Makoto Nakanishi. Cyclin A-Cdk1 regulates the origin firing program in mammalian cells. *Proceedings of the National Academy of Sciences*, 106(9):3184–3189, March 2009.

[401] Michael Rape and Marc W. Kirschner. Autonomous regulation of the anaphase-promoting complex couples mitosis to S-phase entry. *Nature*, 432(7017):588–595, December 2004.

[402] S. Geley, E. Kramer, C. Gieffers, J. Gannon, J. M. Peters, and T. Hunt. Anaphase-promoting complex/cyclosome-dependent proteolysis of human cyclin A starts at the beginning of mitosis and is not subject to the spindle assembly checkpoint. *The Journal of Cell Biology*, 153(1):137–148, April 2001.

[403] N. den Elzen and J. Pines. Cyclin A is destroyed in prometaphase and can delay chromosome alignment and anaphase. *The Journal of Cell Biology*, 153(1):121–136, April 2001.

[404] Barbara Di Fiore and Jonathon Pines. How cyclin A destruction escapes the spindle assembly checkpoint. *The Journal of Cell Biology*, 190(4):501–509, August 2010.

[405] J W Harper. The anaphase-promoting complex: It's not just for mitosis any more. *Genes & Development*, 16(17):2179–2206, September 2002.

[406] Richard W Deibler and Marc W. Kirschner. Quantitative reconstitution of mitotic CDK1 activation in somatic cell extracts. *Mol Cell*, 37(6):753–767, March 2010.

[407] Nobumoto Watanabe, Harumi Arai, Jun-Ichi Iwasaki, Masaaki Shiina, Kazuhiro Ogata, Tony Hunter, and Hiroyuki Osada. Cyclin-dependent kinase (CDK) phosphorylation destabilizes somatic Wee1 via multiple pathways. *Proceedings of the National Academy of Sciences of the United States of America*, 102(33):11663–11668, August 2005.

[408] Joon Lee, Akiko Kumagai, and William G Dunphy. Positive regulation of Wee1 by Chk1 and 14-3-3 proteins. *Molecular biology of the cell*, 12(3):551–563, 2001.

[409] Raquel Domínguez-Kelly, Yusé Martín, Stephane Koundrioukoff, Marvin E. Tanenbaum, Veronique A. J. Smits, René H. Medema, Michelle Debatisse, and Raimundo Freire. Wee1 controls genomic stability during replication by regulating the Mus81-Eme1 endonuclease. *The Journal of Cell Biology*, 194(4):567–579, August 2011.

[410] B. B. Zhou, H. Li, J. Yuan, and M. W. Kirschner. Caspase-dependent activation of cyclin-dependent kinases during Fas-induced apoptosis in Jurkat cells. *Proceedings of the National Academy of Sciences of the United States of America*, 95(12):6785–6790, June 1998.

[411] B. Alvarez, C. Martínez-A, B. M. Burgering, and A. C. Carrera. Forkhead transcription factors contribute to execution of the mitotic programme in mammals. *Nature*, 413(6857):744–747, October 2001.

[412] T. W. Leung, S. S. Lin, A. C. Tsang, C. S. Tong, J. C. Ching, W. Y. Leung, R. Gimlich, G. G. Wong, and K. M. Yao. Over-expression of FoxM1 stimulates cyclin B1 expression. *FEBS letters*, 507(1):59–66, October 2001.

[413] I.-Ching Wang, Yi-Ju Chen, Douglas Hughes, Vladimir Petrovic, Michael L. Major, Hyung Jung Park, Yongjun Tan, Timothy Ackerson, and Robert H. Costa. Forkhead box M1 regulates the transcriptional network of genes essential for mitotic progression and genes encoding the SCF (Skp2-Cks1) ubiquitin ligase. *Molecular and Cellular Biology*, 25(24):10875–10894, December 2005.

[414] Stéphanie Dutertre, Martine Cazales, Muriel Quaranta, Carine Froment, Valerie Trabut, Christine Dozier, Gladys Mirey, Jean-Pierre Bouché, Nathalie Theis-Febvre, Estelle Schmitt, Bernard Monsarrat, Claude Prigent, and Bernard Ducommun. Phosphorylation of CDC25B by Aurora-A at the centrosome contributes to the G2-M transition. *Journal of Cell Science*, 117(Pt 12):2523–2531, May 2004.

[415] Stéphanie Dutertre, Simon Descamps, and Claude Prigent. On the role of aurora-A in centrosome function. *Oncogene*, 21(40):6175–6183, September 2002.

[416] Lilia Gheghiani, Damarys Loew, Bérangère Lombard, Jörg Mansfeld, and Olivier Gavet. PLK1 Activation in Late G2 Sets Up Commitment to Mitosis. *Cell Reports*, 19(10):2060–2073, June 2017.

[417] Oleg Timofeev, Onur Cizmecioglu, Entan Hu, Thomas Orlik, and Ingrid Hoffmann. Human Cdc25A phosphatase has a non-redundant function in G2 phase by activating Cyclin A-dependent kinases. *FEBS letters*, 583(4):841–847, February 2009.

[418] S. Kotani, S. Tugendreich, M. Fujii, P. M. Jorgensen, N. Watanabe, C. Hoog, P. Hieter, and K. Todokoro. PKA and MPF-activated polo-like kinase regulate anaphase-promoting complex activity and mitosis progression. *Molecular Cell*, 1(3):371–380, February 1998.

[419] Y. W. Qian, E. Erikson, C. Li, and J. L. Maller. Activated polo-like kinase Plx1 is required at multiple points during mitosis in Xenopus laevis. *Molecular and Cellular Biology*, 18(7):4262–4271, July 1998.

[420] Yann Thomas, Luca Cirillo, Costanza Panbianco, Lisa Martino, Nicolas Tavernier, Françoise Schwager, Lucie Van Hove, Nicolas Joly, Anna Santamaria, Lionel Pintard, and Monica Gotta. Cdk1 Phosphorylates SPAT-1/Bora to Promote Plk1 Activation in C. elegans and Human Cells. *Cell Reports*, 15(3):510–518, April 2016.

[421] Catherine Lindon and Jonathon Pines. Ordered proteolysis in anaphase inactivates Plk1 to contribute to proper mitotic exit in human cells. *The Journal of Cell Biology*, 164(2):233–241, January 2004.

[422] C. P. De Souza, K. A. Ellem, and B. G. Gabrielli. Centrosomal and cytoplasmic Cdc2/cyclin B1 activation precedes nuclear mitotic events. *Experimental Cell Research*, 257(1):11–21, May 2000.

[423] Mark Jackman, Catherine Lindon, Erich A. Nigg, and Jonathon Pines. Active cyclin B1-Cdk1 first appears on centrosomes in prophase. *Nature Cell Biology*, 5(2):143–148, February 2003.

[424] Arne Lindqvist, Helena Källström, Andreas Lundgren, Emad Barsoum, and Christina Karlsson Rosenthal. Cdc25B cooperates with Cdc25A to induce mitosis but has a unique role in activating cyclin B1-Cdk1 at the centrosome. *The Journal of Cell Biology*, 171(1):35–45, October 2005.

[425] Valerie Lobjois, Denis Jullien, Jean-Pierre Bouché, and Bernard Ducommun. The polo-like kinase 1 regulates CDC25B-dependent mitosis entry. *Biochimica Et Biophysica Acta*, 1793(3):462–468, March 2009.

[426] B. Ouyang, W. Li, H. Pan, J. Meadows, I. Hoffmann, and W. Dai. The physical association and phosphorylation of Cdc25C protein phosphatase by Prk. *Oncogene*, 18(44):6029–6036, October 1999.

[427] J. P. Cogswell, C. E. Brown, J. E. Bisi, and S. D. Neill. Dominant-negative polo-like kinase 1 induces mitotic catastrophe independent of cdc25C function. *Cell Growth & Differentiation: The Molecular Biology Journal of the American Association for Cancer Research*, 11(12):615–623, December 2000.

[428] I. Hoffmann, P. R. Clarke, M. J. Marcote, E. Karsenti, and G. Draetta. Phosphorylation and activation of human cdc25-C by cdc2–cyclin B and its involvement in the self-amplification of MPF at mitosis. *The EMBO journal*, 12(1):53–63, January 1993.

[429] A Karaskou, X Cayla, O Haccard, C Jessus, and R Ozon. MPF amplification in Xenopus oocyte extracts depends on a two-step activation of cdc25 phosphatase. *Experimental Cell Research*, 244(2):491–500, November 1998.

[430] A. Lopez-Girona, B. Furnari, O. Mondesert, and P. Russell. Nuclear localization of Cdc25 is regulated by DNA damage and a 14-3-3 protein. *Nature*, 397(6715):172–175, January 1999.

[431] Estelle Schmitt, Rose Boutros, Carine Froment, Bernard Monsarrat, Bernard Ducommun, and Christine Dozier. CHK1 phosphorylates CDC25B during the cell cycle in the absence of DNA damage. *Journal of Cell Science*, 119(Pt 20):4269–4275, October 2006.

[432] Alwin Krämer, Niels Mailand, Claudia Lukas, Randi G. Syljuåsen, Christopher J. Wilkinson, Erich A. Nigg, Jiri Bartek, and Jiri Lukas. Centrosome-associated Chk1 prevents premature activation of cyclin-B-Cdk1 kinase. *Nature Cell Biology*, 6(9):884–891, September 2004.

[433] R Heald, M McLoughlin, and F McKeon. Human wee1 maintains mitotic timing by protecting the nucleus from cytoplasmically activated Cdc2 kinase. *Cell*, 74(3):463–474, August 1993.

[434] MR Jackman and JN Pines. Cyclins and the G2/M transition. *Cancer surveys*, 29:47–73, 1996.

[435] Amnon Golan, Yana Yudkovsky, and Avram Hershko. The cyclin-ubiquitin ligase activity of cyclosome/APC is jointly activated by protein kinases Cdk1-cyclin B and Plk. *The Journal of Biological Chemistry*, 277(18):15552–15557, May 2002.

[436] Adam D Rudner and Andrew W Murray. Phosphorylation by Cdc28 activates the Cdc20-dependent activity of the anaphase-promoting complex. *The Journal of cell biology*, 149(7):1377–1390, 2000.

[437] Xinxian Qiao, Liyong Zhang, Armin M Gamper, Takeo Fujita, and Yong Wan. APC/C-Cdh1: From cell cycle to cellular differentiation and genomic integrity. *Cell Cycle*, 9(19):3904–3912, October 2010.

[438] Vincenzo D'Angiolella, Cecilia Mari, Donatella Nocera, Linda Rametti, and Domenico Grieco. The spindle checkpoint requires cyclin-dependent kinase activity. *Genes & Development*, 17(20):2520–2525, October 2003.

[439] Jamin B. Hein and Jakob Nilsson. Interphase APC/C-Cdc20 inhibition by cyclin A2-Cdk2 ensures efficient mitotic entry. *Nature Communications*, 7:10975, March 2016.

[440] Speranta Avram, Maria Mernea, Dan Florin Mihailescu, Corina Duda Seiman, Daniel Duda Seiman, and Mihai Viorel Putz. Mitotic checkpoint proteins Mad1 and Mad2 - structural and functional relationship with implication in genetic diseases. *Current Computer-Aided Drug Design*, 10(2):168–181, 2014.

[441] Brian R Thornton and David P Toczyski. Precise destruction: An emerging picture of the APC. *Genes & development*, 20(22):3069–3078, 2006.

[442] Jan-Michael Peters. The anaphase promoting complex/cyclosome: A machine designed to destroy. *Nature reviews Molecular cell biology*, 7(9):644–656, 2006.

[443] S K Reddy, M Rape, W A Margansky, and M W Kirschner. Ubiquitination by the anaphase-promoting complex drives spindle checkpoint inactivation. *Nature*, 446(7138):921–925, April 2007.

[444] Luigi Nezi and Andrea Musacchio. Sister chromatid tension and the spindle assembly checkpoint. *Current opinion in cell biology*, 21(6):785–795, December 2009.

[445] Weiping Wang and Marc W. Kirschner. Emi1 preferentially inhibits ubiquitin chain elongation by the anaphase-promoting complex. *Nature Cell Biology*, 15(7):797–806, July 2013.

[446] Bing Ren, Hieu Cam, Yasuhiko Takahashi, Thomas Volkert, Jolyon Terragni, Richard A. Young, and Brian David Dynlacht. E2F integrates cell cycle progression with DNA repair, replication, and G(2)/M checkpoints. *Genes & Development*, 16(2):245–256, January 2002.

[447] Hugh Cam and Brian David Dynlacht. Emerging roles for E2F: Beyond the G1/S transition and DNA replication. *Cancer Cell*, 3(4):311–316, April 2003.

[448] Dawn Coverley, Heike Laman, and Ronald A Laskey. Distinct roles for cyclins E and A during DNA replication complex assembly and activation. *Nature Cell Biology*, 4(7):523–528, 2002.

[449] Michalis Fragkos, Olivier Ganier, Philippe Coulombe, and Marcel Méchali. DNA replication origin activation in space and time. *Nature Reviews Molecular Cell Biology*, 16(6):360–374, June 2015.

[450] Niels Mailand, Jacob Falck, Claudia Lukas, Randi G Syljuåsen, Markus Welcker, Jiri Bartek, and Jiri Lukas. Rapid destruction of human Cdc25A in response to DNA damage. *Science*, 288(5470):1425–1429, 2000.

[451] Maddalena Donzelli and Giulio F Draetta. Regulating mammalian checkpoints through Cdc25 inactivation. *EMBO reports*, 4(7):671–677, July 2003.

[452] Eva Petermann, Apolinar Maya-Mendoza, George Zachos, David A F Gillespie, Dean A Jackson, and Keith W Caldecott. Chk1 requirement for high global rates of replication fork progression during normal vertebrate S phase. *Molecular and Cellular Biology*, 26(8):3319–3326, April 2006.

[453] Irma Sánchez and Brian David Dynlacht. New insights into cyclins, CDKs, and cell cycle control. *Seminars in Cell & Developmental Biology*, 16(3):311–321, June 2005.

[454] F. Uhlmann, F. Lottspeich, and K. Nasmyth. Sister-chromatid separation at anaphase onset is promoted by cleavage of the cohesin subunit Scc1. *Nature*, 400(6739):37–42, July 1999.

[455] Tamar Listovsky and Julian E. Sale. Sequestration of CDH1 by MAD2L2 prevents premature APC/C activation prior to anaphase onset. *The Journal of Cell Biology*, 203(1):87–100, October 2013.

[456] Yuji Nakayama, Yuki Matsui, Yumi Takeda, Mai Okamoto, Kohei Abe, Yasunori Fukumoto, and Naoto Yamaguchi. C-Src but not Fyn promotes proper spindle orientation in early prometaphase. *The Journal of Biological Chemistry*, 287(30):24905–24915, July 2012.

[457] Michelle S. Lu and Christopher A. Johnston. Molecular pathways regulating mitotic spindle orientation in animal cells. *Development*, 140(9):1843–1856, May 2013.

[458] Kathleen G. Bickel, Barbara J. Mann, Joshua S. Waitzman, Taylor A. Poor, Sarah E. Rice, and Patricia Wadsworth. Src family kinase phosphorylation of the motor domain of the human kinesin-5, Eg5. *Cytoskeleton*, 74(9):317–330, September 2017.

[459] Mark Petronczki, Péter Lénárt, and Jan-Michael Peters. Polo on the Rise-from Mitotic Entry to Cytokinesis with Plk1. *Developmental Cell*, 14(5):646–659, May 2008.

[460] Travis L Schmit, Weixiong Zhong, Vijayasaradhi Setaluri, Vladimir S Spiegelman, and Nihal Ahmad. Targeted depletion of Polo-like kinase (Plk) 1 through lentiviral shRNA or a small-molecule inhibitor causes mitotic catastrophe and induction of apoptosis in human melanoma cells. *The Journal of investigative dermatology*, 129(12):2843–2853, December 2009.

[461] Andrea Vecchione, Gustavo Baldassarre, Hideshi Ishii, Milena S. Nicoloso, Barbara Belletti, Fabio Petrocca, Nicola Zanesi, Louise Y. Y. Fong, Sabrina Battista, Daniela Guarnieri, Raffaele Baffa, Hansjuerg Alder, John L. Farber, Peter J. Donovan, and Carlo M. Croce. Fez1/Lzts1 absence impairs Cdk1/Cdc25C interaction during mitosis and predisposes mice to cancer development. *Cancer Cell*, 11(3):275–289, March 2007.

[462] Mark Petronczki, Michael Glotzer, Norbert Kraut, and Jan-Michael Peters. Polo-like kinase 1 triggers the initiation of cytokinesis in human cells by promoting recruitment of the RhoGEF Ect2 to the central spindle. *Developmental Cell*, 12(5):713–725, May 2007.

[463] Zengqiang Yuan, Esther B E Becker, Paola Merlo, Tomoko Yamada, Sara DiBacco, Yoshiyuki Konishi, Erik M Schaefer, and Azad Bonni. Activation of FOXO1 by Cdk1 in cycling cells and postmitotic neurons. *Science (New York, NY)*, 319(5870):1665–1668, March 2008.

[464] Suzanne Floyd, Jonathon Pines, and Catherine Lindon. APC/C Cdh1 targets aurora kinase to control reorganization of the mitotic spindle at anaphase. *Current biology: CB*, 18(21):1649–1658, November 2008.

[465] Benjamin A. Wolfe, Tohru Takaki, Mark Petronczki, and Michael Glotzer. Polo-like kinase 1 directs assembly of the HsCyk-4 RhoGAP/Ect2 RhoGEF complex to initiate cleavage furrow formation. *PLoS biology*, 7(5):e1000110, May 2009.

[466] Kousuke Kasahara, Yuji Nakayama, Yoshimi Nakazato, Kikuko Ikeda, Takahisa Kuga, and Naoto Yamaguchi. Src signaling regulates completion of abscission in cytokinesis through ERK/MAPK activation at the midbody. *The Journal of Biological Chemistry*, 282(8):5327–5339, February 2007.

[467] Mark E. Burkard, Catherine L. Randall, Stéphane Larochelle, Chao Zhang, Kevan M. Shokat, Robert P. Fisher, and Prasad V. Jallepalli. Chemical genetics reveals the requirement for Polo-like kinase 1 activity in positioning RhoA and triggering cytokinesis in human cells. *Proceedings of the National Academy of Sciences of the United States of America*, 104(11):4383–4388, March 2007.

[468] Maria Eugenia Guicciardi and Gregory J Gores. Life and death by death receptors. *The FASEB Journal*, 23(6):1625–1637, June 2009.

[469] Bodvaël Pennarun, Annemieke Meijer, Elisabeth G. E. de Vries, Jan H. Kleibeuker, Frank Kruyt, and Steven de Jong. Playing the DISC: Turning on TRAIL death receptor-mediated apoptosis in cancer. *Biochimica Et Biophysica Acta*, 1805(2):123–140, April 2010.

[470] E. A. Slee, M. T. Harte, R. M. Kluck, B. B. Wolf, C. A. Casiano, D. D. Newmeyer, H. G. Wang, J. C. Reed, D. W. Nicholson, E. S. Alnemri, D. R. Green, and S. J. Martin. Ordering the cytochrome c-initiated caspase cascade: Hierarchical activation of caspases-2, -3, -6, -7, -8, and -10 in a caspase-9-dependent manner. *The Journal of Cell Biology*, 144(2):281–292, January 1999.

[471] V. Cowling and J. Downward. Caspase-6 is the direct activator of caspase-8 in the cytochrome c-induced apoptosis pathway: Absolute requirement for removal of caspase-6 prodomain. *Cell Death and Differentiation*, 9(10):1046–1056, October 2002.

[472] Joshua L Andersen, Carrie E Johnson, Christopher D Freel, Amanda B Parrish, Jennifer L Day, Marisa R Buchakjian, Leta K Nutt, J Will Thompson, M Arthur Moseley, and Sally Kornbluth. Restraint of apoptosis during mitosis through interdomain phosphorylation of caspase-2. *The EMBO Journal*, 28(20):3216–3227, October 2009.

[473] Celia Vogel, Anne Kienitz, Rolf Müller, and Holger Bastians. The mitotic spindle checkpoint is a critical determinant for topoisomerase-based chemotherapy. *The Journal of Biological Chemistry*, 280(6):4025–4028, February 2005.

[474] Akira Masuda, Ken Maeno, Taku Nakagawa, Hiroko Saito, and Takashi Takahashi. Association between mitotic spindle checkpoint impairment and susceptibility to the induction of apoptosis by anti-microtubule agents in human lung cancers. *The American Journal of Pathology*, 163(3):1109–1116, September 2003.

[475] Richa B. Shah, Ruth Thompson, and Samuel Sidi. A mitosis-sensing caspase activation platform? New insights into the PIDDosome. *Molecular & Cellular Oncology*, 3(3):e1059921, May 2016.

[476] H. Li, L. Bergeron, V. Cryns, M. S. Pasternack, H. Zhu, L. Shi, A. Greenberg, and J. Yuan. Activation of caspase-2 in apoptosis. *The Journal of Biological Chemistry*, 272(34):21010–21017, August 1997.

[477] E. A. Slee, C. Adrain, and S. J. Martin. Executioner caspase-3, -6, and -7 perform distinct, non-redundant roles during the demolition phase of apoptosis. *The Journal of Biological Chemistry*, 276(10):7320–7326, March 2001.

[478] Ulrich Maurer, Céline Charvet, Allan S Wagman, Emmanuel Dejardin, and Douglas R Green. Glycogen synthase kinase-3 regulates mitochondrial outer membrane permeabilization and apoptosis by destabilization of MCL-1. *Mol Cell*, 21(6):749–760, March 2006.

[479] Ingrid E. Wertz, Saritha Kusam, Cynthia Lam, Toru Okamoto, Wendy Sandoval, Daniel J. Anderson, Elizabeth Helgason, James A. Ernst, Mike Eby, Jinfeng Liu, Lisa D. Belmont, Josh S. Kaminker, Karen M. O'Rourke, Kanan Pujara, Pawan Bir Kohli, Adam R. Johnson, Mark L. Chiu, Jennie R. Lill, Peter K. Jackson, Wayne J. Fairbrother, Somasekar Seshagiri, Mary J. C. Ludlam, Kevin G. Leong, Erin C. Dueber, Heather Maecker, David C. S. Huang, and Vishva M. Dixit. Sensitivity to antitubulin chemotherapeutics is regulated by MCL1 and FBW7. *Nature*, 471(7336):110–114, March 2011.

[480] Qingqing Ding, Longfei Huo, Jer-Yen Yang, Weiya Xia, Yongkun Wei, Yong Liao, Chun-Ju Chang, Yan Yang, Chien-Chen Lai, Dung-Fang Lee, Chia-Jui Yen, Yun-Ju Rita Chen, Jung-Mao Hsu, Hsu-Ping Kuo, Chun-Yi Lin, Fuu-Jen Tsai, Long-Yuan Li, Chang-Hai Tsai, and Mien-Chie Hung. Down-regulation of myeloid cell leukemia-1 through inhibiting Erk/Pin 1 pathway by sorafenib facilitates chemosensitization in breast cancer. *Cancer Research*, 68(15):6109–6117, August 2008.

[481] K. J. Townsend, J. L. Trusty, M. A. Traupman, A. Eastman, and R. W. Craig. Expression of the antiapoptotic MCL1 gene product is regulated by a mitogen activated protein kinase-mediated pathway triggered through microtubule disruption and protein kinase C. *Oncogene*, 17(10):1223–1234, September 1998.

[482] Rhonda Croxton, Yihong Ma, Lanxi Song, Eric B Haura, and W Douglas Cress. Direct repression of the Mcl-1 promoter by E2F1. *Oncogene*, 21(9):1359–1369, February 2002.

[483] Margaret E. Harley, Lindsey A. Allan, Helen S. Sanderson, and Paul R. Clarke. Phosphorylation of Mcl-1 by CDK1-cyclin B1 initiates its Cdc20-dependent destruction during mitotic arrest. *The EMBO journal*, 29(14):2407–2420, July 2010.

[484] Chiou-Feng Lin, Cheng-Chieh Tsai, Wei-Ching Huang, Yu-Chih Wang, Po-Chun Tseng, Tsung-Ting Tsai, and Chia-Ling Chen. Glycogen Synthase Kinase-3β and Caspase-2 Mediate Ceramide- and Etoposide-Induced Apoptosis by Regulating the Lysosomal-Mitochondrial Axis. *PloS One*, 11(1):e0145460, 2016.

[485] Eun-Sil Sung, Kyung-Jin Park, Hye-Ji Choi, Chul-Ho Kim, and Yong-Sung Kim. The proteasome inhibitor MG132 potentiates TRAIL receptor agonist-induced apoptosis by stabilizing tBid and Bik in human head and neck squamous cell carcinoma cells. *Experimental Cell Research*, 318(13):1564–1576, August 2012.

[486] E. Yang, J. Zha, J. Jockel, L. H. Boise, C. B. Thompson, and S. J. Korsmeyer. Bad, a heterodimeric partner for Bcl-XL and Bcl-2, displaces Bax and promotes cell death. *Cell*, 80(2):285–291, January 1995.

[487] L. C. Spender, D. I. O'Brien, D. Simpson, D. Dutt, C. D. Gregory, M. J. Allday, L. J. Clark, and G. J. Inman. TGF-beta induces apoptosis in human B cells by transcriptional regulation of BIK and BCL-XL. *Cell Death and Differentiation*, 16(4):593–602, April 2009.

[488] Lilly Magdalena Weiß, Manuela Hugle, Sarah Romero, and Simone Fulda. Synergistic induction of apoptosis by a polo-like kinase 1 inhibitor and microtubule-interfering drugs in Ewing sarcoma cells. *International Journal of Cancer*, 138(2):497–506, January 2016.

[489] David T. Terrano, Meenakshi Upreti, and Timothy C. Chambers. Cyclin-dependent kinase 1-mediated Bcl-xL/Bcl-2 phosphorylation acts as a functional link coupling mitotic arrest and apoptosis. *Molecular and Cellular Biology*, 30(3):640–656, February 2010.

[490] Lingli Zhou, Xiaoling Cai, Xueyao Han, Naihan Xu, and Donald C. Chang. CDK1 switches mitotic arrest to apoptosis by phosphorylating Bcl-2/Bax family proteins during treatment with microtubule interfering agents. *Cell Biology International*, 38(6):737–746, June 2014.

[491] N. Bah, L. Maillet, J. Ryan, S. Dubreil, F. Gautier, A. Letai, P. Juin, and S. Barillé-Nion. Bcl-xL controls a switch between cell death modes during mitotic arrest. *Cell Death & Disease*, 5:e1291, June 2014.

[492] Céline Gélinas and Eileen White. BH3-only proteins in control: Specificity regulates MCL-1 and BAK-mediated apoptosis. *Genes & Development*, 19(11):1263–1268, June 2005.

[493] D. G. Kirsch, A. Doseff, B. N. Chau, D. S. Lim, N. C. de Souza-Pinto, R. Hansford, M. B. Kastan, Y. A. Lazebnik, and J. M. Hardwick. Caspase-3-dependent cleavage of Bcl-2 promotes release of cytochrome c. *The Journal of Biological Chemistry*, 274(30):21155–21161, July 1999.

[494] B. Elangovan and G. Chinnadurai. Functional dissection of the pro-apoptotic protein Bik. Heterodimerization with anti-apoptosis proteins is insufficient for induction of cell death. *The Journal of Biological Chemistry*, 272(39):24494–24498, September 1997.

[495] L. O'Connor, A. Strasser, L. A. O'Reilly, G. Hausmann, J. M. Adams, S. Cory, and D. C. Huang. Bim: A novel member of the Bcl-2 family that promotes apoptosis. *The EMBO journal*, 17(2):384–395, January 1998.

[496] X. Fang, S. Yu, A. Eder, M. Mao, R. C. Bast, D. Boyd, and G. B. Mills. Regulation of BAD phosphorylation at serine 112 by the Ras-mitogen-activated protein kinase pathway. *Oncogene*, 18(48):6635–6640, November 1999.

[497] S R Datta, H Dudek, X Tao, S Masters, H Fu, Y Gotoh, and M E Greenberg. Akt phosphorylation of BAD couples survival signals to the cell-intrinsic death machinery. *Cell*, 91(2):231–241, October 1997.

[498] Y. Tan, M. R. Demeter, H. Ruan, and M. J. Comb. BAD Ser-155 phosphorylation regulates BAD/Bcl-XL interaction and cell survival. *The Journal of Biological Chemistry*, 275(33):25865–25869, August 2000.

[499] F. Condorelli, P. Salomoni, S. Cotteret, V. Cesi, S. M. Srinivasula, E. S. Alnemri, and B. Calabretta. Caspase cleavage enhances the apoptosis-inducing effects of BAD. *Molecular and Cellular Biology*, 21(9):3025–3036, May 2001.

[500] Agshin F. Taghiyev, Natalya V. Guseva, Hisashi Harada, C. Michael Knudson, Oskar W. Rokhlin, and Michael B. Cohen. Overexpression of BAD potentiates sensitivity to tumor necrosis factor-related apoptosis-inducing ligand treatment in the prostatic carcinoma cell line LNCaP. *Molecular cancer research: MCR*, 1(7):500–507, May 2003.

[501] M. A. Jacquin, J. Chiche, B. Zunino, M. Bénéteau, O. Meynet, L. A. Pradelli, S. Marchetti, A. Cornille, M. Carles, and J.-E. Ricci. GAPDH binds to active Akt, leading to Bcl-xL increase and escape from caspase-independent cell death. *Cell Death and Differentiation*, 20(8):1043–1054, August 2013.

[502] S. Pugazhenthi, A. Nesterova, C. Sable, K. A. Heidenreich, L. M. Boxer, L. E. Heasley, and J. E. Reusch. Akt/protein kinase B up-regulates Bcl-2 expression through cAMP-response element-binding protein. *The Journal of Biological Chemistry*, 275(15):10761–10766, April 2000.

[503] Bernhard Gillissen, Frank Essmann, Philipp G. Hemmati, Antje Richter, Anja Richter, Ilker Oztop, Govindaswamy Chinnadurai, Bernd Dörken, and Peter T. Daniel. Mcl-1 determines the Bax dependency of Nbk/Bik-induced apoptosis. *The Journal of Cell Biology*, 179(4):701–715, November 2007.

[504] J. M. Boyd, G. J. Gallo, B. Elangovan, A. B. Houghton, S. Malstrom, B. J. Avery, R. G. Ebb, T. Subramanian, T. Chittenden, and R. J. Lutz. Bik, a novel death-inducing protein shares a distinct sequence motif with Bcl-2 family proteins and interacts with viral and cellular survival-promoting proteins. *Oncogene*, 11(9):1921–1928, November 1995.

[505] Rosie Hughes, Jonathan Gilley, Mark Kristiansen, and Jonathan Ham. The MEK-ERK pathway negatively regulates bim expression through the 3' UTR in sympathetic neurons. *BMC neuroscience*, 12:69, July 2011.

[506] P. F. Dijkers, R. H. Medema, J. W. Lammers, L. Koenderman, and P. J. Coffer. Expression of the pro-apoptotic Bcl-2 family member Bim is regulated by the forkhead transcription factor FKHR-L1. *Current biology: CB*, 10(19):1201–1204, October 2000.

[507] Vesa Hongisto, Nina Smeds, Stephan Brecht, Thomas Herdegen, Michael J. Courtney, and Eleanor T. Coffey. Lithium blocks the c-Jun stress response and protects neurons via its action on glycogen synthase kinase 3. *Molecular and Cellular Biology*, 23(17):6027–6036, September 2003.

[508] Veena Potluri, Sunil K. Noothi, Subrahmanya D. Vallabhapurapu, Sang-Oh Yoon, James J. Driscoll, Charles H. Lawrie, and Sivakumar Vallabhapurapu. Transcriptional repression of Bim by a novel YY1-RelA complex is essential for the survival and growth of Multiple Myeloma. *PloS One*, 8(7):e66121, 2013.

[509] Zhe Wang, Baochun Zhang, Liqun Yang, Jane Ding, and Han-Fei Ding. Constitutive production of NF-kappaB2 p52 is not tumorigenic but predisposes mice to inflammatory autoimmune disease by repressing Bim expression. *The Journal of Biological Chemistry*, 283(16):10698–10706, April 2008.

[510] Zoltán Wiener, Arja M. Band, Pauliina Kallio, Jenny Högström, Ville Hyvönen, Seppo Kaijalainen, Olli Ritvos, Caj Haglund, Olli Kruuna, Sylvie Robine, Daniel Louvard, Yinon Ben-Neriah, and Kari Alitalo. Oncogenic mutations in intestinal adenomas regulate Bim-mediated apoptosis induced by TGF-β. *Proceedings of the National Academy of Sciences of the United States of America*, 111(21):E2229–2236, May 2014.

[511] Sneha Ramesh, Gary M. Wildey, and Philip H. Howe. Transforming growth factor beta (TGFbeta)-induced apoptosis: The rise & fall of Bim. *Cell Cycle (Georgetown, Tex.)*, 8(1):11–17, January 2009.

[512] Akane Inoue-Yamauchi and Hideaki Oda. EMT-inducing transcription factor ZEB1-associated resistance to the BCL-2/BCL-XL inhibitor is overcome by BIM upregulation in ovarian clear cell carcinoma cells. *Biochemical and Biophysical Research Communications*, 526(3):612–617, June 2020.

[513] Patricia Gomez-Bougie, Régis Bataille, and Martine Amiot. The imbalance between Bim and Mcl-1 expression controls the survival of human myeloma cells. *European Journal of Immunology*, 34(11):3156–3164, November 2004.

[514] H. Yamada, S. Tada-Oikawa, A. Uchida, and S. Kawanishi. TRAIL causes cleavage of bid by caspase-8 and loss of mitochondrial membrane potential resulting in apoptosis in BJAB cells. *Biochemical and Biophysical Research Communications*, 265(1):130–133, November 1999.

[515] Kai Huang, Jingjing Zhang, Katelyn L. O'Neill, Channabasavaiah B. Gurumurthy, Rolen M. Quadros, Yaping Tu, and Xu Luo. Cleavage by Caspase 8 and Mitochondrial Membrane Association Activate the BH3-only Protein Bid during TRAIL-induced Apoptosis. *The Journal of Biological Chemistry*, 291(22):11843–11851, May 2016.

[516] H Li, H Zhu, C J Xu, and J Yuan. Cleavage of BID by caspase 8 mediates the mitochondrial damage in the Fas pathway of apoptosis. *Cell*, 94(4):491–501, August 1998.

[517] John-Paul Upton, Kathryn Austgen, Mari Nishino, Kristen M. Coakley, Andrew Hagen, Dan Han, Feroz R. Papa, and Scott A. Oakes. Caspase-2 cleavage of BID is a critical apoptotic signal downstream of endoplasmic reticulum stress. *Molecular and Cellular Biology*, 28(12):3943–3951, June 2008.

[518] Hyungjin Kim, Mubina Rafiuddin-Shah, Ho-Chou Tu, John R. Jeffers, Gerard P. Zambetti, James J.-D. Hsieh, and Emily H.-Y. Cheng. Hierarchical regulation of mitochondrion-dependent apoptosis by BCL-2 subfamilies. *Nature Cell Biology*, 8(12):1348–1358, December 2006.

[519] Kristopher A. Sarosiek, Xiaoke Chi, John A. Bachman, Joshua J. Sims, Joan Montero, Luv Patel, Annabelle Flanagan, David W. Andrews, Peter Sorger, and Anthony Letai. BID preferentially activates BAK while BIM preferentially activates BAX, affecting chemotherapy response. *Molecular Cell*, 51(6):751–765, September 2013.

[520] Dayong Zhai, Chaofang Jin, Ziwei Huang, Arnold C. Satterthwait, and John C. Reed. Differential regulation of Bax and Bak by anti-apoptotic Bcl-2 family proteins Bcl-B and Mcl-1. *The Journal of Biological Chemistry*, 283(15):9580–9586, April 2008.

[521] Simon N. Willis, Lin Chen, Grant Dewson, Andrew Wei, Edwina Naik, Jamie I. Fletcher, Jerry M. Adams, and David C. S. Huang. Proapoptotic Bak is sequestered by Mcl-1 and Bcl-xL, but not Bcl-2, until displaced by BH3-only proteins. *Genes & Development*, 19(11):1294–1305, June 2005.

[522] Erinna F. Lee, Stephanie Grabow, Stephane Chappaz, Grant Dewson, Colin Hockings, Ruth M. Kluck, Marlyse A. Debrincat, Daniel H. Gray, Matthew T. Witkowski, Marco Evangelista, Anne Pettikiriarachchi, Philippe Bouillet, Rachael M. Lane, Peter E. Czabotar, Peter M. Colman, Brian J. Smith, Benjamin T. Kile, and W. Douglas Fairlie. Physiological restraint of Bak by Bcl-xL is essential for cell survival. *Genes & Development*, 30(10):1240–1250, May 2016.

[523] Haiming Dai, X. Wei Meng, Sun-Hee Lee, Paula A. Schneider, and Scott H. Kaufmann. Context-dependent Bcl-2/Bak interactions regulate lymphoid cell apoptosis. *The Journal of Biological Chemistry*, 284(27):18311–18322, July 2009.

[524] Jaigi P. Mathai, Marc Germain, and Gordon C. Shore. BH3-only BIK regulates BAX,BAK-dependent release of Ca2+ from endoplasmic reticulum stores and mitochondrial apoptosis during stress-induced cell death. *The Journal of Biological Chemistry*, 280(25):23829–23836, June 2005.

[525] M. C. Wei, T. Lindsten, V. K. Mootha, S. Weiler, A. Gross, M. Ashiya, C. B. Thompson, and S. J. Korsmeyer. tBID, a membrane-targeted death ligand, oligomerizes BAK to release cytochrome c. *Genes & Development*, 14(16):2060–2071, August 2000.

[526] Marc Germain, Jocelyn Milburn, and Vincent Duronio. MCL-1 inhibits BAX in the absence of MCL-1/BAX Interaction. *The Journal of Biological Chemistry*, 283(10):6384–6392, March 2008.

[527] M. C. Wei, W. X. Zong, E. H. Cheng, T. Lindsten, V. Panoutsakopoulou, A. J. Ross, K. A. Roth, G. R. MacGregor, C. B. Thompson, and S. J. Korsmeyer. Proapoptotic BAX and BAK: A requisite gateway to mitochondrial dysfunction and death. *Science (New York, N.Y.)*, 292(5517):727–730, April 2001.

[528] Z. N. Oltvai, C. L. Milliman, and S. J. Korsmeyer. Bcl-2 heterodimerizes in vivo with a conserved homolog, Bax, that accelerates programmed cell death. *Cell*, 74(4):609–619, August 1993.

[529] S. Manon, B. Chaudhuri, and M. Guérin. Release of cytochrome c and decrease of cytochrome c oxidase in Bax-expressing yeast cells, and prevention of these effects by coexpression of Bcl-xL. *FEBS letters*, 415(1):29–32, September 1997.

[530] Liying Zhou and Donald C. Chang. Dynamics and structure of the Bax-Bak complex responsible for releasing mitochondrial proteins during apoptosis. *Journal of Cell Science*, 121(Pt 13):2186–2196, July 2008.

[531] Zheng Dong, Manjeri A. Venkatachalam, Jinzhao Wang, Yogendra Patel, Pothana Saikumar, Gregg L. Semenza, Thomas Force, and Junichiro Nishiyama. Up-regulation of Apoptosis Inhibitory Protein IAP-2 by Hypoxia: HIF-1-INDEPENDENT MECHANISMS*. *Journal of Biological Chemistry*, 276(22):18702–18709, June 2001.

[532] Ping Hu, Zhang Han, Anthony D. Couvillon, and John H. Exton. Critical role of endogenous Akt/IAPs and MEK1/ERK pathways in counteracting endoplasmic reticulum stress-induced cell death. *The Journal of Biological Chemistry*, 279(47):49420–49429, November 2004.

[533] C. Du, M. Fang, Y. Li, L. Li, and X. Wang. Smac, a mitochondrial protein that promotes cytochrome c-dependent caspase activation by eliminating IAP inhibition. *Cell*, 102(1):33–42, July 2000.

[534] S. M. Srinivasula, M. Ahmad, T. Fernandes-Alnemri, and E. S. Alnemri. Autoactivation of procaspase-9 by Apaf-1-mediated oligomerization. *Molecular Cell*, 1(7):949–957, June 1998.

[535] Q. L. Deveraux, N. Roy, H. R. Stennicke, T. Van Arsdale, Q. Zhou, S. M. Srinivasula, E. S. Alnemri, G. S. Salvesen, and J. C. Reed. IAPs block apoptotic events induced by caspase-8 and cytochrome c by direct inhibition of distinct caspases. *The EMBO journal*, 17(8):2215–2223, April 1998.

[536] H. R. Stennicke, J. M. Jürgensmeier, H. Shin, Q. Deveraux, B. B. Wolf, X. Yang, Q. Zhou, H. M. Ellerby, L. M. Ellerby, D. Bredesen, D. R. Green, J. C. Reed, C. J. Froelich, and G. S. Salvesen. Pro-caspase-3 is a major physiologic target of caspase-8. *The Journal of Biological Chemistry*, 273(42):27084–27090, October 1998.

[537] Sabrina L. Spencer, Suzanne Gaudet, John G. Albeck, John M. Burke, and Peter K. Sorger. Non-genetic origins of cell-to-cell variability in TRAIL-induced apoptosis. *Nature*, 459(7245):428–432, May 2009.

[538] S. J. Riedl, M. Renatus, R. Schwarzenbacher, Q. Zhou, C. Sun, S. W. Fesik, R. C. Liddington, and G. S. Salvesen. Structural basis for the inhibition of caspase-3 by XIAP. *Cell*, 104(5):791–800, March 2001.

[539] P. Li, D. Nijhawan, I. Budihardjo, S. M. Srinivasula, M. Ahmad, E. S. Alnemri, and X. Wang. Cytochrome c and dATP-dependent formation of Apaf-1/caspase-9 complex initiates an apoptotic protease cascade. *Cell*, 91(4):479–489, November 1997.

[540] B. B. Wolf, M. Schuler, F. Echeverri, and D. R. Green. Caspase-3 is the primary activator of apoptotic DNA fragmentation via DNA fragmentation factor-45/inhibitor of caspase-activated DNase inactivation. *The Journal of Biological Chemistry*, 274(43):30651–30656, October 1999.
