# Supplementary material for: A Boolean network model of hypoxia, mechanosensing and TGF-β signaling captures the role of phenotypic plasticity and mutations in tumor metastasis
Source: PLoS Comput Biol. 2025 Apr 16;21(4):e1012735. doi: 10.1371/journal.pcbi.1012735 (PMC12061430; doi:10.1371/journal.pcbi.1012735)
Supplement: S1 Fig — (PDF) [file pcbi.1012735.s001.pdf]

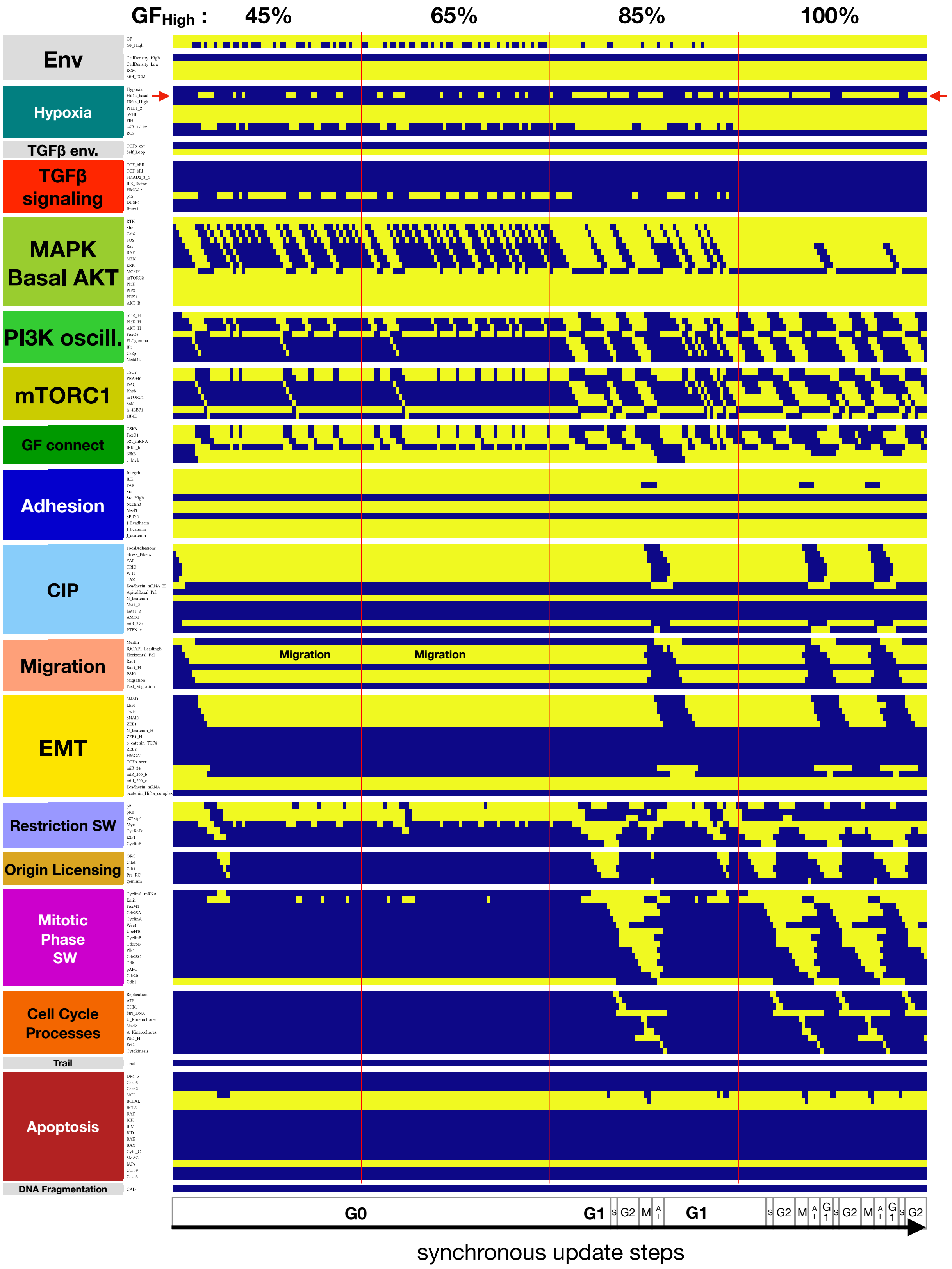

S1 Fig. Hif-1α is required for proliferation under normoxia (full version of Fig. 2A). A) Dynamics of relevant regulatory molecule expression/activity during exposure of a quiescent cell to increasing mitogen signaling (60 update-steps of 45%, 65%, 85% and 100% high GF; full network dynamics in SM Fig. 1A). *X-axis:* update steps annotated by cell cycle phase (G0: quiescence; G1: start of cell cycle entry; S: DNA synthesis; G2: growth phase 2; M: metaphase; A T: anaphase, telophase, cytokinesis); *y-axis*: nodes organized by regulatory module; *yellow/ blue*: ON/OFF; *red arrow:* Hif1a_basal node.
