# Supplementary material for: A Boolean network model of hypoxia, mechanosensing and TGF-β signaling captures the role of phenotypic plasticity and mutations in tumor metastasis
Source: PLoS Comput Biol. 2025 Apr 16;21(4):e1012735. doi: 10.1371/journal.pcbi.1012735 (PMC12061430; doi:10.1371/journal.pcbi.1012735)
Supplement: Attachment — Submitted filename: Hypoxia _ response to Reviewers.pdf [file pcbi.1012735.s019.pdf]

Response to Reviewer Comments

Ref.: **PCOMPBIOL-D-24-02209**
A Boolean network model of hypoxia, mechanosensing and TGF-β signaling captures the role of phenotypic plasticity and mutations in tumor metastasis
PLOS Computational Biology

Dear Dr. Ravasz Regan,

Thank you for submitting your manuscript to PLOS Computational Biology. After careful consideration, we feel that it has merit but does not fully meet PLOS Computational Biology's publication criteria as it currently stands. Therefore, we invite you to submit a revised version of the manuscript that addresses the points raised during the review process.

[…]

We look forward to receiving your revised manuscript.

Kind regards,

Sylvain Soliman
Academic Editor
PLOS Computational Biology

Stacey Finley
Section Editor
PLOS Computational Biology

We would like to thank Drs. Soliman and Finley for the opportunity to revise and resubmit our manuscript. Moreover, we thank our three reviewers for the time and effort they dedicated to providing us with encouraging and valuable feedback. I especially wish to thank you for the rare confidence boost our undergraduate student authors received upon reading your positive reviews. Below we have included a summary of our revisions, followed by a **point by point response** to all reviewer comments, as well as journal requirements raised by our editors.

Summary of Revisions:

Text:

— In *Results*, we clarified the text referencing our simulation setup for Fig. 6 so that the reasons for a lack of apoptosis in step 3 are more clear.

— We added a substantial paragraph to our *Discussion* to consider the applicability of our model to specific types of cancer, placing some of our supplementary figure results in this context.

— We completed the truncated sentence in our *Introduction,* and proof-red the manuscript once more.

Figures:

— We corrected “% time” to “fraction of time” on all figures.

— We added the originally “not shown” data to SM Figure 7C.

— We replaced the images on Fig. 6, SM Figs 9-11 with a new series generated from a Wikipedia commons image and added attribution to each figure caption.

— We fixed axis-label cropping issues.

Reviewers' comments:

Reviewer's Responses to Questions

Reviewer #1: This manuscript proposes Boolean model that integrates hypoxic signaling with a mechanosensitive model of EMT. The model is impressively comprehensive, it which includes the EMT-promoting crosstalk of mitogens and biomechanical signals, cell cycle control, and apoptosis. The model is validated by showing that it reproduces multiple phenotypes and processes, for example hypoxic protection from anoikis.

The model has high explanatory power, as well as predictive power. For example, the model yields experimentally testable predictions about the effect of loss of the Von Hippel Landau protein (VHL) on cancer hallmarks, with or without secondary oncogene activation.

This model is a key advance, integrating multiple cancer hallmarks. It can be further extended to cover DNA damage response and senescence, which would then cover all single-cell cancer hallmarks.

The methods and results are well-documented and the writing is clear. In addition to its clear biological/medical implications, this manuscript is a very good example of successful modeling.

We would like to thank Reviewer #1 for their recognition of our work.

Reviewer #2: In this work, the authors built a Boolean network model that extends their previously published model to integrate a hypoxic response module and study the dynamics of signaling pathways associated with the epithelial-mesenchymal transition.

Their model successfully reproduces opposite outputs that depend on multiple signals and integrates the influence of biomechanical features of the environment, such as the stiffness of the extracellular matrix. The links and nodes in the Boolean network can be traced to experimental evidence in the literature, and the authors validated the results of their simulations by matching them to experimental data.

The article is well-structured and well-written. The introduction is thorough and provides the reader with enough context to understand the results, particularly the paradoxical hypoxia responses. Every claim in the results can be easily traced to a figure, and the computational methods and tools that were used are well-described and referenced. Additionally, the supplementary files include all the data needed to run the model and ensure reproducible results. The Discussion section, although somewhat lengthy, recapitulates the results, comments on their relationship with the hallmarks of cancer, and discusses the limitations of the model. Overall, I must admit that I really enjoyed reading and reviewing this article. It is a great example of the relevance of Boolean networks and their application in cancer systems biology, and I hope the authors continue expanding the use of this model.

We would like to thank Reviewer #2 for their recognition of our work, and it is great to hear that the manuscript was fun to read. We will certainly continue extending the model, and put it to use in several disease contexts, including specific types of cancer.

Reviewer #3: The manuscript presents a single-cell Boolean network model that incorporates hypoxia signaling in a previously published mechanosensitive epithelial to mesenchymal transition (EMT) model. The authors aim to model the signaling crosstalk between environmental factors, such as extracellular matrix stiffness, cell density, hypoxia, and growth signals, that are central for tumor metastasis, and they also aim to model the effect of these factors on the cell cycle, cell death, and EMT.

The integration of these processes in a single model is a significant advancement; in the literature, these processes are mostly studied in isolation. The authors used their in-house simulation software dynmod and provided sufficient detail for the reproducibility of their work. The model underwent extensive validation and could reproduce key findings from the literature. The authors provide predictions for interactions, between environmental factors and mutations, that affect cancer hallmarks. Original experimental validations for the model and predictions were not included.

Overall, this manuscript is written well for the most part and its results provide significant insight. However, it has a few issues, which the authors should address so that it can be recommended for publication.

We would like to thank Reviewer #3 for their encouraging assessment of our work, and hope to have addressed all their concerns below.

Major issues:

1. The authors include a detailed discussion about the limitations of the model; however, an extra discussion should be added about the applicability of the predictions of a general model to a specific type of cancer cell, which can have numerous cell-type specific interactions that are not captured by the general model.

We thank Reviewer #3 for this suggestion, as we agree that making clear connection from our model to understanding and predicting the behavior of specific cancers better highlights the potential impact of our work. To address this, we added the following paragraph to our *Discussion*:

> "The challenges of applying a cell-type agnostic model of hypoxia-induced EMT to specific carcinomas are at least three-fold. First, the most prevalent driver mutations and pathway alterations driving carcinoma vary substantially by tissue (e.g., VHL loss in ccRCC vs. $BRAF^{V600E}$ mutations in melanoma). Moreover, our current model does not cover pathways that regulate genomic instability or immune evasion (150). Second, there is substantial genetic diversity within tumors of the same type; not only among patients but also among cells from a single tumor (151,152). Third, tumor growth is a cell population-level phenomenon, where cell-cell interactions go beyond contact inhibition or EMT-promoting signaling (modeled here), extending to signals from tumor-associated fibroblasts and immune cells (not modeled). Given these challenges, it is important to address: *i)* which, if any, tumor cell type do we expect our predictions to apply to, and *ii)* how can our model answer questions about specific cancers? *i)* Our predictions address mechano-sensitive responses such as EMT or proliferation under hypoxia (b,d,e) or the effects of specific mutations (a,c) in controlled conditions. Thus, we expect our predicted trends to hold *in vitro* in several carcinoma cell lines, with substantial variation in effect size and/or the environments the effects are strongest in (note that most mutation combinations only change a subset of model behaviors, if any; **SM Fig. 7**). *ii)* Given the challenges of predicting cancer cell behaviors *in vivo*, we expect our model to be of use in predicting the *range* of cell behaviors that mutations associated with a specific carcinoma can generate, in combination, in different microenvironments (e.g., **SM Fig. 7** for ccRCC), in response to chemotherapy /targeted therapy. These predicted responses can then be compared to single-cell RNA sequencing from tumors (153), analyzed with a focus on cell-behavior signatures rather than individual genes (e.g., EMT, cell cycle, apoptosis). This can help us pinpoint enriched behaviors compared to healthy tissues and predict mutation combinations that co-occur in single cells to drive tissue behavior (testable with DNA sequencing).
>
> Single-cell transcriptomics can also identify key tissue-specific cancer cell signaling pathways missing from our model, pointing to cell-cell interactions we most need to account for. As an example, our in silico metastatic cascade is a simplification of the highly complex tumor and blood microenvironments experienced by circulating tumor cells. […]"

2. In line 511, "apoptosis of a neighbor" is mentioned as an example of how an epithelial cell can gain the room necessary for EMT. However, on Figure 6, no apoptosis can be observed in the corresponding pulse, Pulse 3. This may lead to confusion, and the authors should clarify how cell density can decrease at this pulse.

We agree that there is an apparent contradiction here, which could benefit from a

better explanation. The key to it is that our Boolean model tracks the fate of single cells experiencing their microenvironment *independently* from each other, as dictated by the input settings of our simulation (e.g., high vs. low density). Thus we do not capture population-level phenomena such as apoptosis in a subset of our simulated cells leading to an overall decrease of density, experienced in turn by other simulated cells. Instead, we ask: what do *live* cells do if their neighborhood becomes less dense (steps 2 → 3 → 4 in **Fig. 6**)? As a result, we do not assume that these live cells are subject to pro-apoptotic signals / environments; just a density change. This means that there is no apoptotic response in steps 2-4; not until cells loose all ECM attachment inside blood vessels in step 5.

This logic is not apparent in our original submission. To clarify the process we do model and better tie it to changes in tumor structure, we edited the paragraph to read as follows (<u>new text underlined</u>):

> "To do this, we started with an epithelial cell at high cell density on a stiff ECM, such as the basement membrane (120) (**Fig. 6A,** pulse 1). In a growing solid tumor, this cell is likely to experience hypoxia (**Fig. 6A,** pulse 2). Yet, due to density-dependent contact inhibition of proliferation as well as migration, <u>this epithelial cell is protected from hypoxia-driven EMT. For EMT to start, a decrease in local density must occur (**Fig. 6A**, pulse 3). In a tumor setting this may be due to cell death in the neighborhood, potentially due to nearby anoxic areas of necrosis (not modeled)(79) or changes to constraining structures such as the basement membrane (121).</u> Migrating away from the solid tumor, a mesenchymal cell can now reach areas of lower density and adequate oxygen, while maintaining its mesenchymal state (**Fig. 6A**, pulse 4)."

<u>*Minor issues:*</u>

1. On page 8, the sentence: "Under moderate hypoxia, localization of Hif-1α to the mitochondria prevents oxidative-stress induced apoptosis through…" seems to be unfinished.

Fixed and reference added:

> "Under moderate hypoxia, localization of Hif-1α to the mitochondria prevents oxidative-stress induced apoptosis <u>by reducing mitochondrial transcription and ROS production (80).</u>"

2. Figures 2, 3, 4, 6: y axis is labelled "% time in phenotype", but values are 0–1 and not in percentage.

Fixed on all main and supplementary figures.

Figure 4B: values along the y axis are partially obstructed.

Fixed.

On SM Fig. 5B-C the x axis label is missing.

Fixed.

3. In line 300, authors should introduce the abbreviation SAC for those unfamiliar.

Replaced with "Spindle Assembly Checkpoint (SAC)".

4. On page 28, the line “We predict that the loss of TGF-β signaling can… slow down hypoxia-induced EMT in cells with weak mitogenic signaling (SM Fig. 3).” seems to incorrectly refer to SM Fig. 3 based on the line on page 15: “…TGF-β receptor I knockdown does not block hypoxia-induced EMT – though it slows it in mild hypoxia (Fig. 3B-C).”

Thank you; we corrected it to "(Fig. 3B-C)”.

5. Authors should proofread the manuscript for typos and omissions.

We did a thorough proofread and grammar check; corrected mistakes throughout.

Journal Requirements:

1) We noticed that you used the phrase 'not shown' in the manuscript. We do not allow these references, as the PLOS data access policy requires that all data be either published with the manuscript or made available in a publicly accessible database. Please amend the supplementary material to include the referenced data or remove the references.

We expanded Supplementary Figure 7C to include the data previously marked as “not shown”, and referenced it in the text.

2) Please upload all main figures as separate Figure files in .tif or .eps format. For more information about how to convert and format your figure files please see our guidelines: https://journals.plos.org/ploscompbiol/s/figures

We converted all main figures in .TIFF format, processed them with PACE, and uploaded the .TIF files generated by PACE.

3) Some material included in your submission may be copyrighted. According to PLOSu2019s copyright policy, authors who use figures or other material (e.g., graphics, clipart, maps) from another author or copyright holder must demonstrate or obtain permission to publish this material under the Creative Commons Attribution 4.0 International (CC BY 4.0) License used by PLOS journals. Please closely review the details of PLOSu2019s copyright requirements here: PLOS Licenses and Copyright. If you need to request permissions from a copyright holder, you may use PLOS's Copyright Content Permission form.
Please respond directly to this email and provide any known details concerning your material's license terms and permissions required for reuse, even if you have not yet obtained copyright permissions or are unsure of your material's copyright compatibility. Once you have responded and addressed all other outstanding technical requirements, you may resubmit your manuscript within Editorial Manager.

Potential Copyright Issues:
i) Figures 6A, Supplementary Figures(9A, 10, and 11). Please confirm whether you drew the images / clip-art within the figure panels by hand. If you did not draw the images, please provide (a) a link to the source of the images or icons and their license / terms of use; or (b) written permission from the copyright holder to publish the images or icons under our CC BY 4.0 license. Alternatively, you may replace the images with open source alternatives. See these open source resources you may use to replace images / clip-art:
- https://commons.wikimedia.org
- https://openclipart.org/.

The images in Figures 6A and SM Figures 9-11 were created with BioRender, but we do not currently have a license to secure a copyright. We thus created an image series with similar content by using a Wikipedia Commons image, then added appropriate *Image credits* sentences to each figure. Moreover, for SM Figure 11 we also included credits for the small mitotic spindle image used in some of the figure icons, as follows:

> "*Image credits:* metastatic cascade adapted from *https://commons.wikimedia.org/wiki/File:Contribution_of_EMT_to_cancer_progression.jpg*; mitotic spindle image in cycling cell icons: *https://commons.wikimedia.org/wiki/File:Kinetochore.jpg."*

4) Please amend your detailed Financial Disclosure statement. This is published with the article. It must therefore be completed in full sentences and contain the exact wording you wish to be published.

1) State what role the funders took in the study. If the funders had no role in your study, please state: "The funders had no role in study design, data collection and analysis, decision to publish, or preparation of the manuscript."

We added this statement to the end of the *funding details* box inside Financial Disclosure (see full text below).

5) Please ensure that the funders and grant numbers match between the Financial Disclosure field and the Funding Information tab in your submission form. Note that the funders must be provided in the same order in both places as well. Currently, "the Faculty Development Funds, and Biochemistry and Molecular Biology Program" are missing from the Funding Information tab.

The *funding details* box inside **Financial Disclosure** now reads:

> "All funding for this study was provided by The College of Wooster (https://wooster.edu): a) E.R. would like to thank the Henry Luce III Fund for Distinguished Scholarship, b) the Faculty Development Funds and c) the Life Sciences Endowed Funds for research support; d) I.Z thanks the Henry J. Copeland Independent Study Fund for research travel support; e) G.G. thanks Wooster's Biochemistry and Molecular Biology Program for support to present his work. The funders had no role in study design, data collection and analysis, decision to publish, or preparation of the manuscript."

We have also updated the Funding Information to include all 5 items, in the order lister above.

6) Please include a completed 'Competing Interests' statement in the text box when submitting your production task, including any COIs declared by your co-authors, written in full sentences.
If you have no competing interests to declare, please state "The authors have declared that no competing interests exist". You may also provide an updated statement via email.

We updated the field to "*The authors have declared that no competing interests exist.*"

7) Please ensure that the Supplementary Files (1-4) are uploaded in a correct numerical order in the online submission form.

As the online submission portal reorders these whenever Supplementary File 4 is a pdf but the others are .zip files, we have zipped up Supplementary File 4 and placed it last.

Figure resubmission:

While revising your submission, please upload your figure files to the Preflight Analysis and Conversion Engine (PACE) digital diagnostic tool, https://pacev2.apexcovantage.com/. PACE helps ensure that figures meet PLOS requirements. To use PACE, you must first register as a user. Registration is free. Then, login and navigate to the UPLOAD tab, where you will find detailed instructions on how to use the tool. If you encounter any issues or have any questions when using PACE, please email PLOS at figures@plos.org. Please note that Supporting Information files do not need this step. If there are other versions of figure files still present in your submission file inventory at resubmission, please replace them with the PACE-processed versions.

We converted all main figures in .TIFF format, processed them with PACE, and uploaded the .TIF files generated by PACE.
